# Rational design of phenyl 2,4,5-trichlorobenzenesulfonate based thiosemicarbazones as α-glucosidase and α-amylase inhibitors: integrating enzymatic evaluation and molecular modeling

**DOI:** 10.1039/d5ra08761a

**Published:** 2026-01-06

**Authors:** Faiqa Noreen, Magdi E. A. Zaki, Nastaran Sadeghian, Feyzi Sinan Tokali, Parham Taslimi, Rima D. Alharthy, Halil Şenol, Xianliang Zhao, Furkan Çakır, Sobhi M. Gomha, Zahid Shafiq

**Affiliations:** a Institute of Chemical Sciences, Bahauddin Zakariya University 60800 Multan Pakistan zahidshafiq@bzu.edu.pk; b Department of Chemistry, Faculty of Science, Imam Mohammad Ibn Saud Islamic University (IMSIU) Riyadh 11623 Saudi Arabia; c Department of Biotechnology, Faculty of Science, Bartin University 74110 Bartin Türkiye; d Department of Material and Material Processing Technologies, Kars Vocational School, Kafkas University 36100 Kars Türkiye; e Department of Chemistry, Science & Arts College, Rabigh Branch, King Abdulaziz University Rabigh 21911 Saudi Arabia; f Department of Pharmaceutical Chemistry, Faculty of Pharmacy, Bezmialem Vakif University 34093 Fatih Ístanbul; g School of Biological and Chemical Engineering, Zhejiang University of Science and Technology Hangzhou 310023 Zhejiang Province China; h Department of Chemistry, Faculty of Science, Islamic University of Madinah Madinah 42351 Saudi Arabia smgomha@iu.edu.sa

## Abstract

The present study aimed to investigate the antidiabetic potential of a new series of thiosemicarbazone derivatives through integrated *in vitro* enzymatic assays and *in silico* molecular modeling. The synthesized compounds were evaluated for their inhibitory activities against α-glucosidase (α-Glu) and α-amylase (α-Amy) enzymes. Among the tested derivatives, compound 16 (2-chlorophenyl-substituted) demonstrated the most potent dual inhibition with IC_50_ values of 14.58 nM (α-Glu) and 88.37 nM (α-Amy), surpassing the reference drug acarbose in potency. Molecular docking analyses revealed that compound 16 formed stable interactions with Asn-214, Glu-276, Phe-157, and Tyr-71 in the α-Glu and Asp-197, Glu-233, and Lys-200 in α-Amy's active site. These key interactions were further supported by 250 ns molecular dynamics simulations, confirming the conformational stability of both complexes with average RMSD values below 2.0 Å and minimal ligand fluctuations. Energy decomposition analysis indicated that van der Waals and electrostatic interactions were the major contributors to the overall binding free energy. *In silico* ADME profiling predicted favorable pharmacokinetic properties, including high gastrointestinal absorption, good oral bioavailability, and compliance with Lipinski's rule of five, while no significant blood–brain barrier penetration was observed. The combined *in vitro* and *in silico* findings highlight compound 16 as a promising lead candidate for further optimization and development as a dual α-Glu and α-Amy inhibitor for the management of type 2 diabetes mellitus.

## Abstract 2

A new series of thiosemicarbazone derivatives were synthesized and evaluated as dual α-Glu/α-Amy inhibitors.

## Graphical abstract

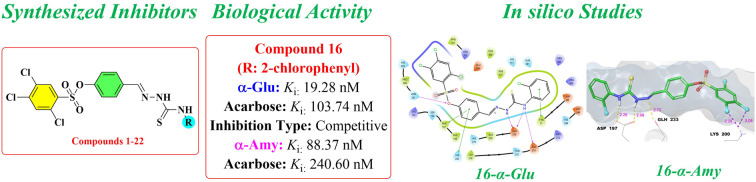

## 1. Introduction

Diabetes mellitus is a chronic metabolic disorder characterized by impaired carbohydrate, lipid, and protein metabolism resulting from defects in insulin secretion, insulin action, or both. The persistent hyperglycemia associated with diabetes leads to severe long-term complications, including cardiovascular diseases, nephropathy, neuropathy, and retinopathy.^1,2^ In recent decades, the global prevalence of diabetes has increased dramatically, making it one of the major health concerns worldwide. One of the most effective therapeutic strategies to control postprandial hyperglycemia involves the inhibition of carbohydrate-hydrolyzing enzymes such as α-amylase and α-glucosidase, which are responsible for the breakdown of complex carbohydrates into glucose.^3,4^ Thus, α-Amy and α-Glu inhibitors play a pivotal role in delaying glucose absorption and maintaining normal blood glucose levels, providing a rational approach for the management of type 2 diabetes mellitus (T2DM).

The inhibition of α-Amy and α-Glu is an established therapeutic approach for the management of type 2 diabetes mellitus. These enzymes catalyze the hydrolysis of dietary polysaccharides and disaccharides into absorbable monosaccharides, primarily glucose, leading to a rapid increase in postprandial blood sugar levels.^4,5^ By inhibiting these enzymes, the digestion and absorption of carbohydrates are delayed, resulting in a gradual release of glucose into the bloodstream. This mechanism not only reduces postprandial hyperglycemia but also minimizes the overall glycemic load and insulin demand.^6,7^

Currently, several α-Glu and α-Amy inhibitors, such as acarbose, miglitol, and voglibose, are clinically available and widely prescribed. However, their clinical utility is often limited due to undesirable gastrointestinal side effects, including flatulence, abdominal discomfort, and diarrhea, which result from the excessive fermentation of undigested carbohydrates in the colon. Furthermore, issues related to poor selectivity, low bioavailability, and suboptimal pharmacokinetic profiles further restrict their long-term use.^8–10^ Therefore, the discovery and development of novel α-glucosidase and α-amylase inhibitors with improved potency, selectivity, and safety profiles remain a crucial goal in antidiabetic drug research.

**Scheme 1:**
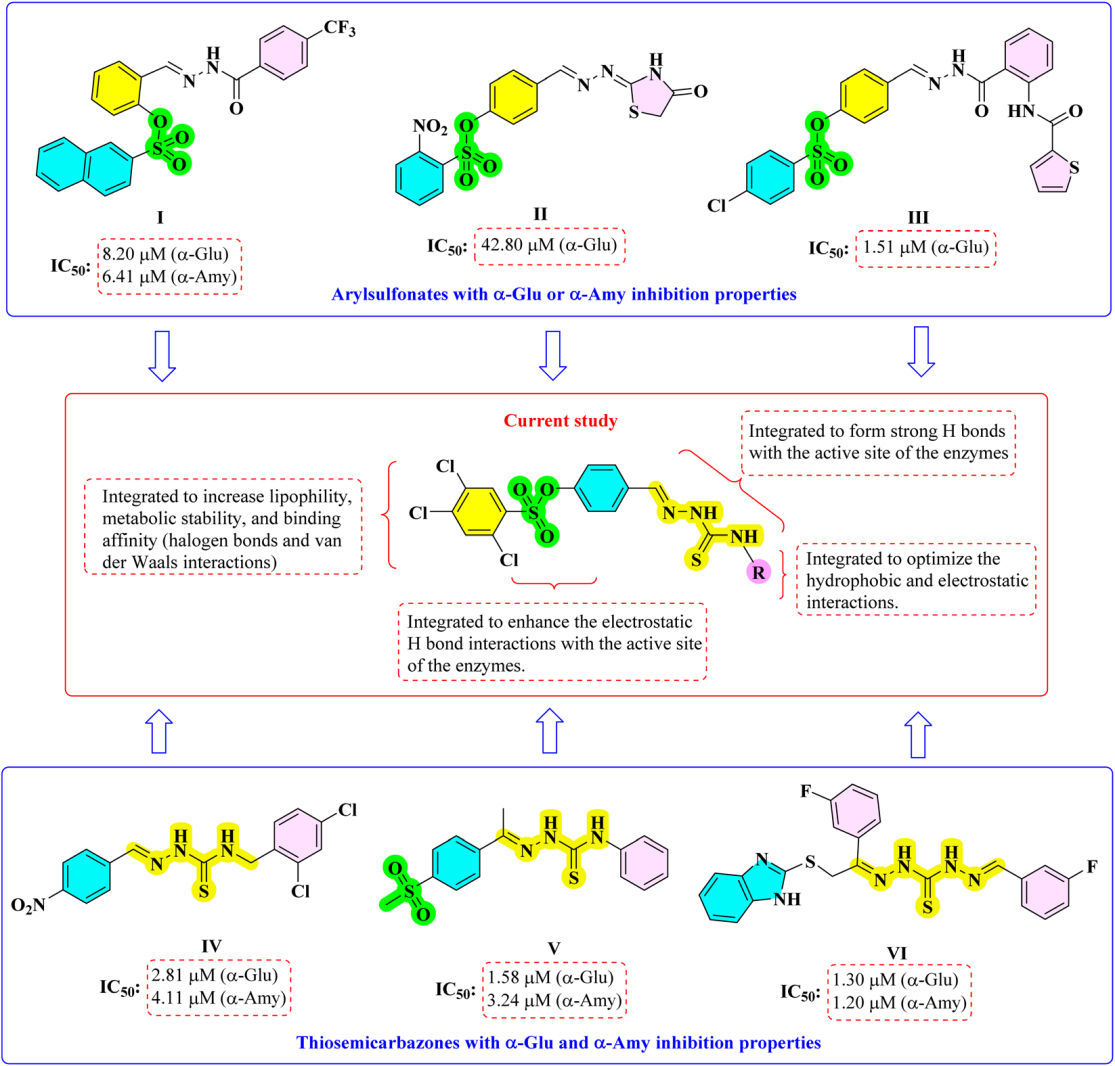
The rational design strategy of synthesized compounds.

Aryl sulfonates constitute a structurally diverse class of compounds widely explored in medicinal chemistry due to their broad pharmacological potential. They have been reported to exhibit anticancer,^11^ antimicrobial,^12^ antiviral,^13^ antiobesity,^14^ and antidiabetic (I,^15^ II,^16^ III^17^) activities (Scheme 1). The sulfonyl moiety plays a crucial role in modulating physicochemical and pharmacokinetic properties, often enhancing molecular stability, lipophilicity, and membrane permeability. Moreover, the strong electron-withdrawing nature of the sulfonyl group enables effective interactions with various biological targets, particularly enzymes and receptors involved in metabolic and inflammatory pathways.^18,19^ Similarly, thiosemicarbazones represent another versatile pharmacophore class with wide-ranging biological activities. They have been extensively studied for their anticancer,^20^ antimicrobial,^21^ antiviral,^22^ antioxidant^23^ and antidiabetic (IV,^24^ V,^25^ VI^26^) properties (Scheme 1). Structurally, thiosemicarbazones possess both imine and thione functionalities, which serve as key pharmacophoric centers capable of establishing strong electronic or hydrophobic interactions with active site residues of enzymes. These features endow thiosemicarbazones with remarkable potential to inhibit enzymatic activity or modulate redox processes at the molecular level. Due to their structural flexibility and binding diversity, thiosemicarbazones continue to attract interest as lead structures for the design of novel therapeutic agents across multiple disease categories, including metabolic disorders such as diabetes.^27,28^

Although numerous thiosemicarbazone-based derivatives have been reported as effective α-Glu and α-Amy inhibitors, most of these studies primarily focus on single-enzyme inhibition or limited substituent variation. Moreover, systematic investigations addressing the influence of diverse aliphatic and aromatic substituents on dual α-Glu and α-Amy inhibition remain relatively scarce. In particular, the integration of sulfonyl-containing moieties within thiosemicarbazone frameworks and their impact on enzyme inhibition profiles have not been comprehensively explored. Therefore, there is still a need for structurally diversified thiosemicarbazone derivatives that enable a clearer understanding of structure–activity relationships and provide improved inhibitory potency against both enzymes.

Guided by the well-documented pharmacological relevance of both aryl sulfonates and thiosemicarbazones, the present study was designed to integrate these two bioactive moieties within a single molecular framework to achieve enhanced enzyme inhibitory potential. The design strategy involved the introduction of a sulfonyl group onto the phenolic ring of 4-hydroxybenzaldehyde using 2,4,5-trichlorobenzenesulfonyl chloride, generating a reactive sulfonated aldehyde intermediate. The sulfonyl functionality was incorporated to improve the molecular lipophilicity, metabolic stability, and binding affinity through possible electrostatic and hydrogen-bonding interactions within the enzyme active site. Additionally, the presence of chlorine substituents on the aromatic ring was expected to modulate the electronic distribution further and enhance hydrophobic interactions with nonpolar residues of the enzyme pocket, thereby strengthening the overall enzyme–ligand binding. Halogen atoms, particularly chlorine, are known to participate in halogen bonding and van der Waals interactions, which can significantly contribute to molecular recognition and potency enhancement in enzyme inhibition. Subsequently, condensation of this aldehyde with various thiosemicarbazides furnished a series of thiosemicarbazone derivatives, wherein the azomethine and thioamide functionalities were expected to contribute additional binding interactions, such as hydrogen bonding and hydrophobic interactions, enhancing overall inhibitory efficiency. The substituents were selected to provide systematic variation in steric bulk, electronic properties, and aromatic character at the R position. This design strategy was intended to enable a comparative evaluation of aliphatic *versus* aromatic substituents, as well as the influence of substituent size and position on dual α-Glu and α-Amy inhibition, thereby facilitating meaningful structure–activity relationship analysis (Scheme 1).

In light of these considerations, the present study aims to design, synthesize, and biologically evaluate a new series of sulfonated thiosemicarbazone derivatives as potential dual inhibitors of α-Glu and α-Amy, with a particular focus on elucidating the effects of substituent variation on enzyme inhibitory activity.

## 2. Results and discussion

### 2.1. Chemistry

**Scheme 2:**
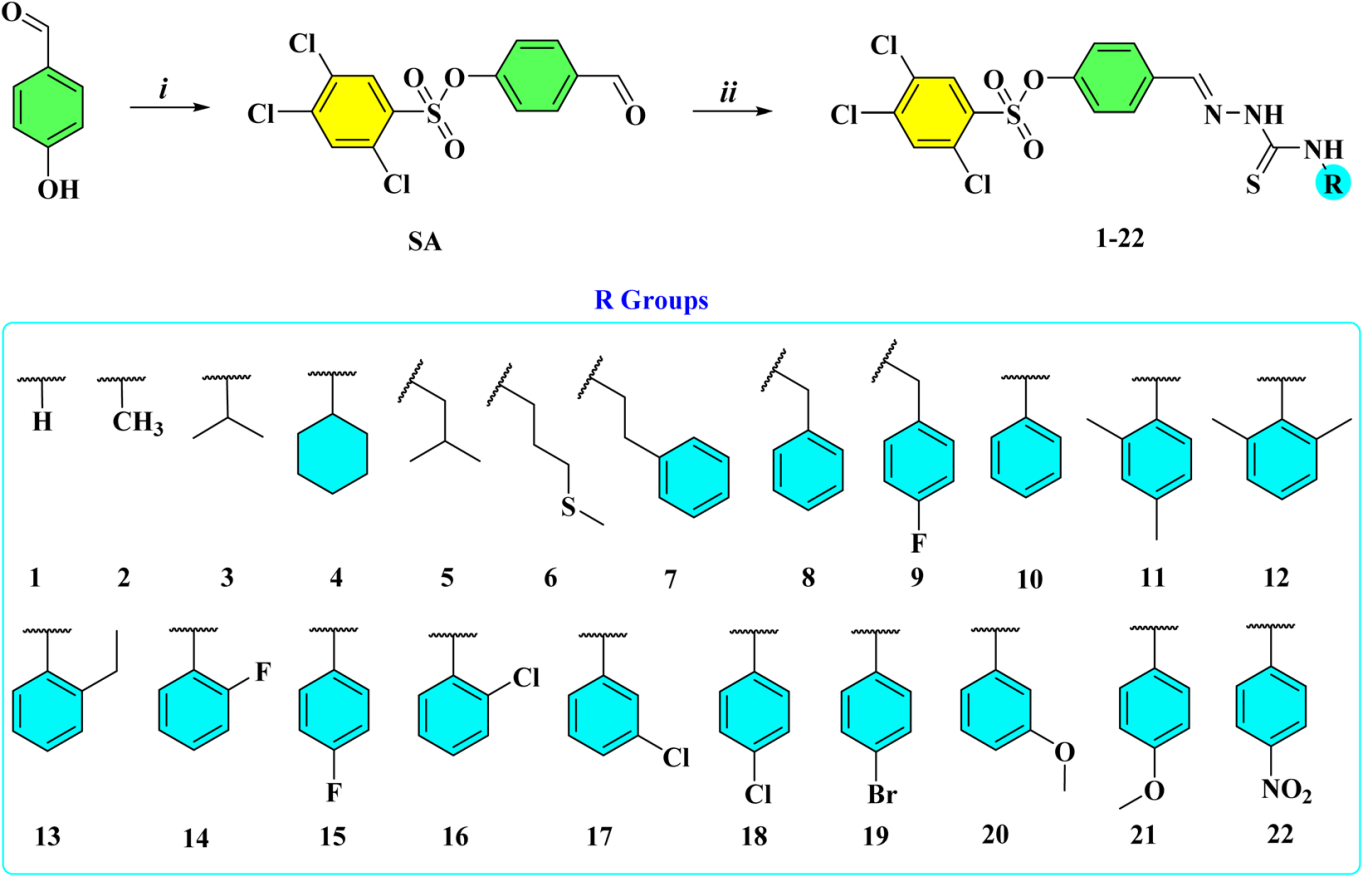
Synthesis of compounds 1–22. Reaction conditions: (i) 2,4,5-trichlorobenzenesulfonyl chloride (1 eq.), triethylamine (1.1 eq.), DMF, 0–5 °C, 1 hour; (ii) thiosemicarbazides (1 eq.), MeOH, AcOH (2–3 drops), reflux, 3–6 hours.

In the present work, a series of twenty-two novel thiosemicarbazone derivatives were designed, synthesized, and evaluated for their α-Glu and α-Amy inhibitory activities. The synthetic route commenced with the sulfonation of 4-hydroxybenzaldehyde using 2,4,5-trichlorobenzenesulfonyl chloride under basic conditions to furnish the corresponding sulfonylated aldehyde intermediate (SA).^29^ This key intermediate was subsequently subjected to condensation with a variety of thiosemicarbazides, affording the desired thiosemicarbazone derivatives (1–22) in satisfactory yields (Scheme 2).^30^ The chemical structures of all synthesized compounds were unambiguously confirmed by spectroscopic techniques, including ^1^H, ^13^C, and ^19^F NMR, FTIR, and HRMS analyses.

Analysis of the ^1^H NMR spectra exhibited characteristic resonance patterns consistent with the thiosemicarbazone framework. The hydrazinic NH protons appeared as distinct singlets within the *δ* 12.24–11.47 ppm range. The thioamide NH proton signals were observed as singlets at *δ* 10.44–9.91 ppm for the aromatic-substituted derivatives, whereas they appeared as triplets at *δ* 9.14–8.55 ppm for the aliphatic analogues.^31^ In addition, the azomethine protons resonated as singlets in the *δ* 8.38–8.35 ppm region, confirming the successful formation of the imine linkage in all synthesized compounds.^30^ The ^13^C NMR spectra further supported these findings, displaying characteristic signals of C=S carbons in the *δ* 178.7–176.0 ppm region, indicative of the thioamide functionality.^32^ The N=CH carbons appeared at *δ* 142.7–140.7 ppm, consistent with the expected chemical environment of the imine carbon.^33^ Furthermore, for fluorine-containing derivatives, the aromatic carbon resonances appeared as doublets due to C–F coupling, providing additional evidence for the incorporation of fluorinated substituents within the aromatic framework.^34^ The FTIR spectra of the synthesized compounds exhibited characteristic absorption bands consistent with the proposed structures. The N–H stretching vibrations were observed in the ranges of 3358–3234 and 3188–3119 cm^−1^. The characteristic N=CH stretching vibration appeared in the region of 1606–1585 cm^−1^, confirming the formation of the imine moiety. The asymmetric and symmetric SO_2_ bending vibrations were detected at 1392–1354 and 1153–1134 cm^−1^, respectively. In addition, the C=S stretching vibration was observed in the range of 1069–1061 cm^−1^.

### 2.2. Biological activity

#### 2.2.1. *In vitro* α-Glu activity and SAR analysis

The synthesized thiosemicarbazone derivatives were evaluated for their *in vitro* α-Glu inhibitory activity to assess their potential as enzyme inhibitors relevant to antidiabetic research. The experiments were carried out using *Saccharomyces cerevisiae* α-Glu, with acarbose serving as the reference inhibitor.^35^ The obtained results demonstrated that several compounds exhibited potent enzyme inhibition, in certain cases comparable or superior to acarbose. The corresponding inhibition data for all tested derivatives are listed in Table 1.

**Table 1:** *In vitro* biological activity results for α-Glu and α-Amy inhibition activity

Compounds	α-Glu			α-Amy	
	IC_50_ (nM)	*R* ^2^	*K*_*i*_ (nM)	IC_50_ (nM)	*R* ^2^
1	31.90	0.933	38.55 ± 4.86	172.46	0.920
2	10.17	0.913	18.48 ± 2.57	149.17	0.982
3	25.87	0.906	31.04 ± 4.17	113.07	0.901
4	29.56	0.947	40.33 ± 5.51	170.36	0.921
5	53.86	0.917	71.27 ± 6.02	117.64	0.946
6	20.16	0.974	35.17 ± 4.23	164.50	0.948
7	15.43	0.918	17.74 ± 3.06	151.70	0.919
8	17.27	0.935	22.41 ± 3.17	163.60	0.969
9	45.32	0.971	62.28 ± 7.13	98.21	0.993
10	67.23	0.962	81.54 ± 4.64	163.18	0.980
11	56.54	0.984	79.90 ± 6.23	106.15	0.944
12	35.22	0.968	44.48 ± 3.42	163.21	0.973
13	52.19	0.974	66.13 ± 4.72	124.24	0.988
14	38.08	0.990	47.98 ± 5.01	174.18	0.985
15	25.23	0.938	26.16 ± 2.31	126.12	0.918
16	14.58	0.988	19.28 ± 3.55	88.37	0.957
17	40.82	0.989	44.21 ± 3.91	100.92	0.980
18	64.21	0.925	87.13 ± 7.52	155.14	0.919
19	17.18	0.918	21.86 ± 3.28	152.48	0.912
20	32.01	0.976	40.74 ± 3.03	126.72	0.927
21	40.73	0.984	53.70 ± 4.60	161.46	0.989
22	24.51	0.960	34.70 ± 4.28	166.14	0.932
Acarbose	92.75	0.962	103.74 ± 6.52	240.60	0.950

As shown in Table 1, all synthesized thiosemicarbazone derivatives demonstrated significant inhibitory activity against α-Glu, with IC_50_ values ranging from 10.17 to 67.23 nM. Most of compounds exhibited stronger inhibition than the standard drug acarbose (IC_50_ = 92.75 nM), highlighting the efficiency of this scaffold in targeting α-Glu. Among the tested series, compounds 2 (IC_50_ = 10.17 nM, *K*_i_ = 18.48 nM) and 16 (IC_50_ = 14.58 nM, *K*_i_ = 19.28 nM) emerged as the most potent inhibitors, showing nearly 9-fold higher activity compared to acarbose. Other derivatives such as 7 (IC_50_ = 15.43 nM), 8 (17.27 nM), and 19 (17.18 nM) also displayed substantial inhibitory potency, indicating a consistent trend of high α-Glu inhibition within this series. Conversely, compounds like 10 (67.23 nM) and 18 (64.21 nM) exhibited relatively weaker activity, though still outperforming the reference compound. These findings emphasize that the designed thiosemicarbazone framework provides a robust basis for α-Glu inhibition, with certain substitutions leading to remarkable enhancement in activity.

According to the kinetic evaluation, compounds 2, 7, 16, and 19 were determined to act as competitive inhibitors of α-Glu, whereas the other tested analogs and the reference drug acarbose exhibited non-competitive inhibition (see SI). In competitive inhibition, the inhibitor binds directly to the enzyme's active site, thereby competing with the substrate for occupancy of the catalytic pocket. This interaction blocks substrate access and consequently decreases the catalytic efficiency of the enzyme. A hallmark of this inhibition mode is its reversibility, as enzyme activity can be restored either upon removal of the inhibitor or by increasing the substrate concentration enough to displace it.^36^ Conversely, in non-competitive inhibition, the inhibitor attaches to a distinct allosteric site, independent of substrate binding. This interaction induces conformational changes in the enzyme that diminish its catalytic function regardless of substrate levels. Because it modifies enzyme structure rather than substrate accessibility, this type of inhibition is generally less reversible.^37^

The α-glucosidase inhibitory activity was markedly affected by both the size and the substitution pattern of the R moiety. Small aliphatic substituents led to enhanced activity, with the methyl-substituted compound 2 exhibiting the highest potency (IC_50_ = 10.17 nM), indicating that limited steric hindrance at this position is favorable for enzyme inhibition. Increasing aliphatic bulk, as observed for isopropyl (3), cyclohexyl (4), and isobutyl (5) substituents, resulted in reduced activity, suggesting steric congestion within the enzyme binding region. The introduction of flexible aromatic-containing substituents such as phenethyl (7) and benzyl (8) improved inhibitory potency compared to simple phenyl substitution (10), emphasizing the positive contribution of aromaticity combined with conformational flexibility. Among aromatic derivatives, *ortho*-substituted phenyl groups significantly enhanced activity, particularly halogenated analogs. The 2-chlorophenyl derivative (16) showed one of the strongest inhibitory effects (IC_50_ = 14.58 nM), whereas *para*-substituted analogs such as 4-chlorophenyl (18) and 4-nitrophenyl (22) displayed diminished activity. The SAR analysis indicates that moderate steric bulk combined with aromaticity at the R position is favorable for α-Glu inhibition, whereas excessive bulk or strongly electron-withdrawing substituents reduce activity. These findings suggest that future optimization should focus on *ortho*-substituted aromatic groups with balanced steric and electronic properties to further enhance inhibitory potency.

#### 2.2.2. *In vitro* α-Amy activity and SAR analysis

The α-Amy-catalyzed reactions using polymeric substrates are known to involve multiple binding subsites and non-ideal kinetic behavior, including substrate heterogeneity and partial diffusion limitation. As a result, Lineweaver–Burk-based kinetic analyses may not yield reliable *K*_i_ values. For this reason, α-Amy inhibition was assessed using IC_50_ values, as commonly reported in the literature.^38–40^ The *in vitro* α-Amy inhibition data (Table 1) indicate that all synthesized thiosemicarbazone derivatives exhibit notable inhibitory activity, surpassing the reference drug acarbose (IC_50_ = 240.60 nM). The most potent compounds were 16 (2-chlorophenyl, IC_50_ = 88.37 nM), 9 (4-fluorobenzyl, IC_50_ = 98.21 nM), and 17 (3-chlorophenyl, IC_50_ = 100.92 nM), demonstrating a marked enhancement in activity compared to acarbose. A group of derivatives, including 7 (phenethyl, 151.70 nM), 8 (benzyl, 163.60 nM), and 19 (4-bromophenyl, 152.48 nM), showed moderate inhibition, while compounds such as 14 (2-fluorophenyl, 174.18 nM) and 6 (2-methylthioethyl, 164.50 nM) displayed the lowest activity in the series. Even the less active derivatives showed higher α-amylase inhibitory activity than acarbose, highlighting the relevance of the thiosemicarbazone scaffold for further enzyme inhibition studies.

Compared to α-Glu, α-Amy inhibition exhibited a narrower activity range; however, clear structure–activity trends were still observed. Compounds bearing aromatic substituents generally showed improved α-Amy inhibition relative to aliphatic analogs. In particular, *ortho*-substituted halogenated phenyl derivatives again demonstrated superior potency, with compound 16 (2-chlorophenyl) emerging as the most active inhibitor (IC_50_ = 88.37 nM). *Meta*- and *para*-substituted halogenated analogs (17–19) showed reduced activity, suggesting that substituent orientation plays a key role in effective enzyme interaction. Electron-donating methoxy-substituted derivatives (20 and 21) exhibited moderate inhibition, while the strongly electron-withdrawing nitro group (22) led to a notable decrease in activity. The α-Amy inhibition appears to favor aromatic substituents with appropriate steric orientation rather than purely electronic effects, and excessive steric bulk or unfavorable substitution patterns adversely impact inhibitory potency. The results highlight the importance of substituent orientation rather than purely electronic effects, with *ortho*-substituted aromatic derivatives showing superior activity compared to *meta*- and *para*-substituted analogs. This trend provides a clear direction for further optimization, favoring fine-tuning of substituent position and steric fit to improve enzyme inhibition.

#### 2.2.3. Dual α-Glu/α-Amy inhibitor profile and selectivity

The synthesized thiosemicarbazone derivatives were evaluated for their dual inhibitory activity against α-Glu and α-Amy to assess both potency and selectivity. The results (Table 1) revealed that several compounds exhibited strong inhibition of both enzymes, with IC_50_ values significantly lower than the reference drug acarbose. Some derivatives, such as 2, 16, and 19, showed comparable potency against both enzymes, indicating a balanced dual inhibitory profile. Other compounds displayed moderate selectivity, favoring either α-Glu or α-Amy, suggesting that substituent type and position can fine-tune enzyme selectivity. These findings suggest that the designed thiosemicarbazone scaffold may serve as a versatile framework for exploring dual α-glucosidase and α-amylase inhibition with tunable selectivity.

### 2.3. Molecular docking

Molecular docking is a fundamental computational tool in rational drug design, offering predictive insights into how small-molecule ligands interact within the active site of their target enzymes. This approach not only identifies the most favorable binding poses but also highlights crucial noncovalent interactions such as hydrogen bonding, π–π stacking, and hydrophobic contacts that stabilize the complex.^41^ To achieve a more realistic representation of the dynamic binding process, the Induced Fit Docking (IFD) protocol was employed, enabling flexibility of both the ligand and the active site residues during docking refinement. Subsequently, Molecular Mechanics-Generalized Born Surface Area (MM-GBSA) calculations were performed to estimate the binding free energies (Δ*G*_bind_) of the resulting complexes, providing thermodynamic evidence supporting the docking results and ranking ligand affinity toward the enzyme.^42^ The IFD scores and MM-GBSA binding free energy calculations indicate that all synthesized compounds exhibit strong binding affinities toward both α-Glu and α-Amy (Table 2).

**Table 2:** IFD scores and MM-GBSA Δ*G* binding free energies of the compounds against α-Glu and α-Amy

Compounds	IFD score (kcal mol^−1^)		MMGBSA Δ*G*_bind._ (kcal mol^−1^)	
	α-Glu^a^ (PDB ID: 3A4A)	α-Amy (PDB ID: 4W93)	α-Glu	α-Amy
1	−8.071	−6.744	−38.91	−56.11
2	−7.617	−6.419	−40.58	−57.79
3	−7.384	−5.825	−56.80	−60.56
4	−6.734	−6.977	−51.23	−43.55
5	−7.718	−5.798	−55.04	−61.40
6	−8.071	−6.744	−38.91	−56.11
7	−7.730	−5.997	−51.46	−53.91
8	−8.950	−7.269	−55.22	−46.95
9	−7.775	−6.214	−54.36	−59.60
10	−6.889	−7.428	−54.73	−53.65
11	−7.585	−7.615	−49.94	−55.09
12	−7.761	−5.818	−57.21	−25.95
13	−7.912	−6.189	−59.72	−53.62
14	−8.429	−8.110	−51.49	−59.28
15	−9.015	−7.088	−54.54	−57.94
16	−9.980	−8.851	−69.67	−66.02
17	−7.348	−7.277	−54.13	−64.10
18	−8.532	−7.019	−51.76	−62.09
19	−9.336	−6.389	−64.13	−37.81
20	−8.879	−7.582	−61.90	−64.47
21	−8.690	−7.383	−56.35	−66.81
22	−8.643	−7.538	−65.84	−65.12

a Homology model based on *S. cerevisiae* isomaltose (PDB ID: 3A4A).

The molecular docking and MM-GBSA results provided valuable insights into the binding preferences of the synthesized thiosemicarbazone derivatives toward both α-Glu and α-Amy. As summarized in Table 2, the IFD scores and binding free energies (Δ*G*_bind_) varied considerably depending on the nature and position of the substituents on the thioamide moiety.

For α-Glu, most compounds exhibited favorable binding affinities, with IFD scores ranging between −6.7 and −9.9 kcal mol^−1^ and Δ*G*_bind_ values spanning from −38.9 to −69.7 kcal mol^−1^. Among them, compound 16 demonstrated the strongest interaction (IFD: −9.980 kcal mol^−1^; Δ*G*_bind_: −69.67 kcal mol^−1^), followed closely by 19 and 22, suggesting that electron-withdrawing groups significantly enhance α-Glu affinity. Compounds 15, 20, and 21 also displayed notable stabilization energies (−55 to −65 kcal mol^−1^), implying that both electron-donating and withdrawing substituents contribute favorably when properly oriented within the enzyme pocket.

In the case of α-Amy, the binding profiles were consistent yet slightly less intense, with IFD scores ranging from −5.8 to −8.9 kcal mol^−1^ and Δ*G*_bind_ values between −25.9 and −66.8 kcal mol^−1^. The most potent interaction was again observed for compound 16 (IFD: −8.851 kcal mol^−1^; Δ*G*_bind_: −66.02 kcal mol^−1^), followed closely by 21 and 22, indicating that polarizable and moderately bulky substituents facilitate stronger binding through hydrophobic and hydrogen-bonding contacts.

**Fig. 1:**
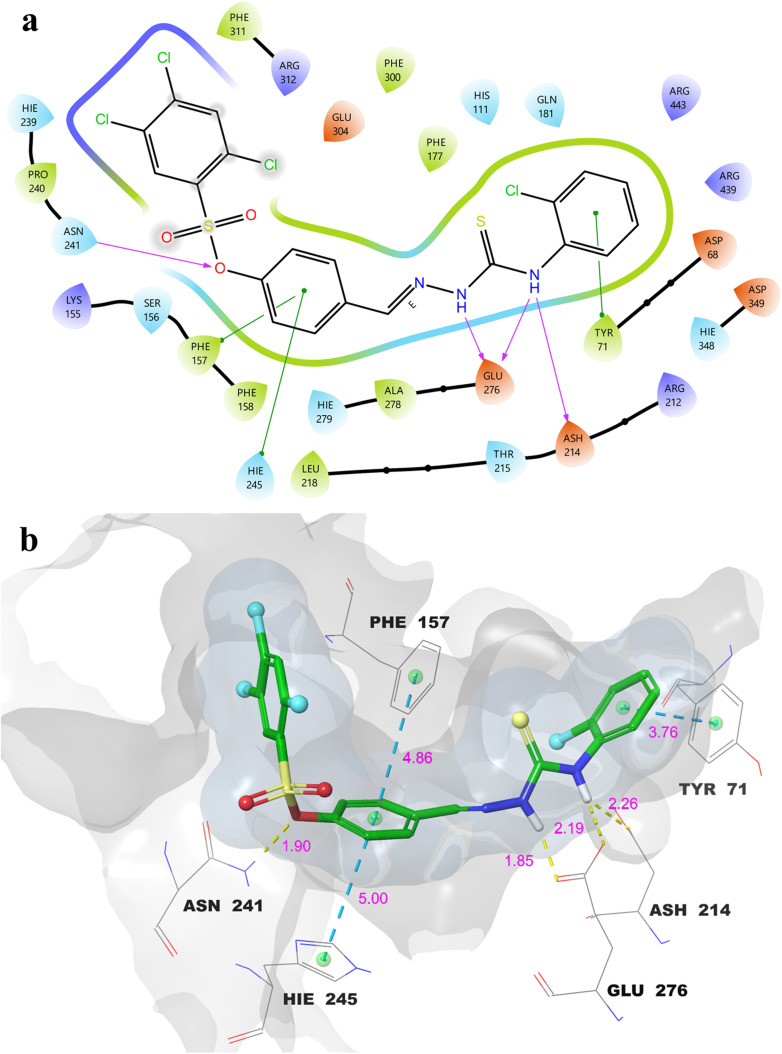
Molecular docking 2D (a) and 3D (b) ligand–protein interactions of 16-α-Glu complex.

In light of the *in vitro* inhibition data and the results obtained from the IFD and MM-GBSA analyses, the most active compound, 16, was subjected to detailed molecular docking visualization. Both 2D and 3D interaction profiles were examined to elucidate and rationalize its possible binding orientations and key molecular interactions within the active sites of α-Glu and α-Amy. The 2D and 3D ligand–protein interaction maps of compound 16 within the α-Glu active site are depicted in Fig. 1.

As illustrated in Fig. 1, the thioamide nitrogen atoms form three strong hydrogen bonds with the catalytic residues Glu-276 and Asp-214 (1.85, 2.19, and 2.26 Å) which play crucial roles in substrate recognition and catalytic proton transfer.^43,44^ An additional hydrogen bond is established between the sulfonate oxygen and Asn-241 (1.90 Å), further stabilizing the ligand within the active pocket.^45^ Beyond these polar contacts, the benzylidene ring engages in π–π stacking interactions with Phe-157 (4.86 Å) and His-245 (5 Å), residues commonly associated with hydrophobic stabilization of aromatic substrates.^46,47^ Moreover, the 2-chlorophenyl moiety exhibits an additional π–π stacking interaction with Tyr-71 (3.76 Å), reinforcing the overall binding affinity through π-surface complementarity. These multiple hydrogen bonding and π–π stacking interactions underscore the strong and specific binding orientation of compound 16 within the α-Glu active site.

**Fig. 2:**
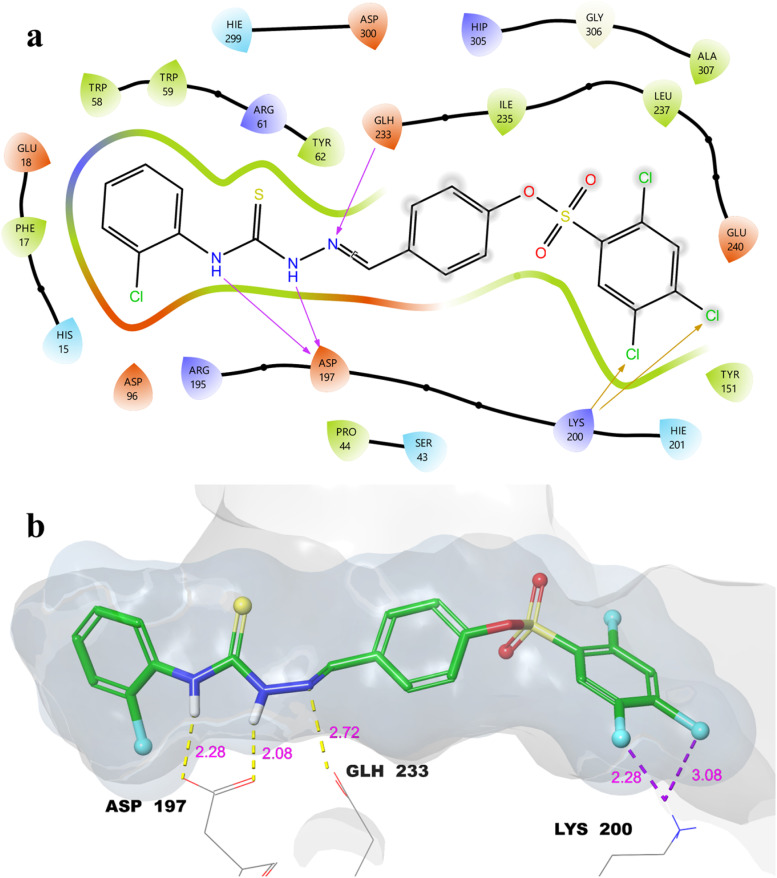
Molecular docking 2D (a) and 3D (b) ligand–protein interactions of 16-α-Amy complex.

The 2D and 3D ligand–protein interaction maps of compound 16 within the α-Amy active site are presented in Fig. 2. In this complex, the thioamide nitrogen atoms form two hydrogen bonds with the catalytic residue Asp-197 (2.08 and 2.28 Å), which is critical for stabilizing the substrate and facilitating enzymatic cleavage.^48,49^ An additional hydrogen bond is observed between the thiosemicarbazone nitrogen and Glu-233 (2.72 Å), further anchoring the ligand within the binding pocket.^50^ The 4- and 5-position chlorines on the 2,4,5-trichlorobenzene moiety engage in two halogen bonds with Lys-200 (2.28 and 3.08 Å), a residue implicated in substrate recognition and stabilization.^51,52^ These combined hydrogen bonding and halogen interactions highlight the high specificity and strength of binding of compound 16 to α-Amy, providing a structural rationale for its observed dual inhibitory potency *in vitro*.

Although detailed 2D/3D interaction analyses were performed for compound 16 due to its superior dual inhibitory potency, comparative docking scores and MM-GBSA binding energies provide valuable insight into the interaction patterns of other representative derivatives. Compounds bearing small alkyl substituents, such as compound 2, showed favorable binding energies mainly driven by hydrophobic interactions but lacked the extensive π–π stacking and halogen-mediated contacts observed for aromatic derivatives. Electron-donating aromatic substituents (20 and 21) contributed additional π–π interactions; however, their *para*-substitution pattern resulted in suboptimal orientation within the enzyme pocket, leading to slightly reduced binding affinity. In contrast, *ortho*-halogenated derivatives (16 and 17) exhibited enhanced stabilization through improved steric complementarity, π–π stacking, and halogen interactions with key active-site residues. These comparative trends support the SAR findings and underscore the importance of substituent type and position in modulating enzyme–ligand interactions.

As a result of the molecular docking studies, compound 16 demonstrated strong and specific binding interactions within the active sites of both α-Glu and α-Amy, consistent with the *in vitro* inhibition data. Key hydrogen bonding, π–π stacking, and halogen interactions observed in the docking poses provide a structural explanation for its high dual inhibitory potency, confirming that the designed thiosemicarbazone scaffold effectively engages critical residues in both enzymes.

### 2.4. Molecular dynamics simulations

**Fig. 3:**
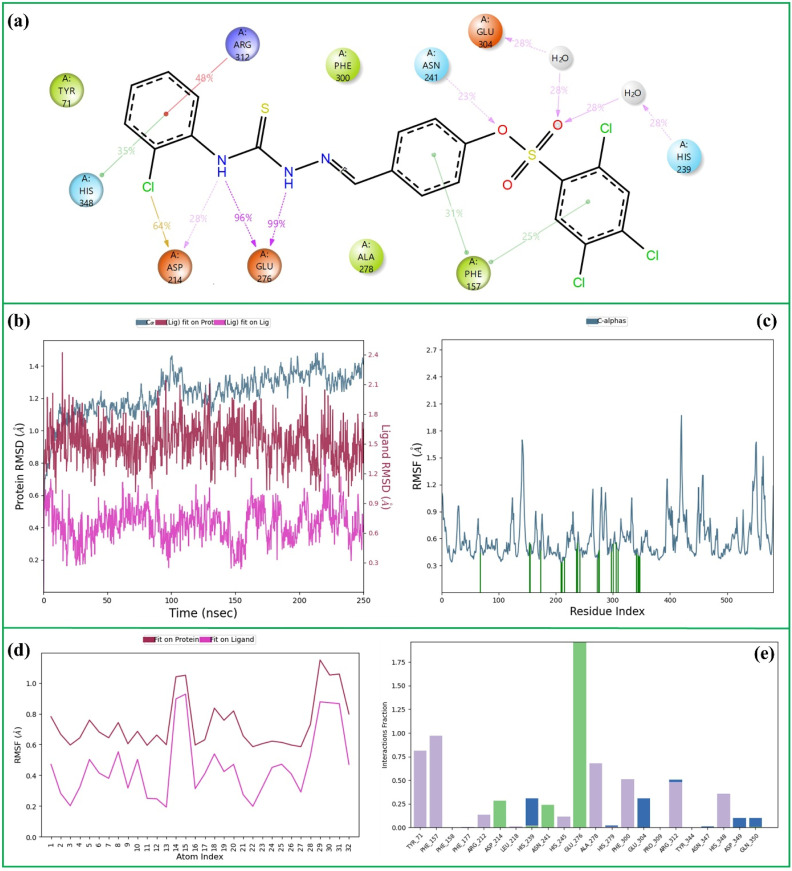
The 250 ns MD simulation analysis of 16-α-Glu complex. (a) 2D key ligand–protein interactions, (b) RMSD of ligand and protein atoms, (c) RMSF of protein atoms, (d) RMSF of ligand atoms, (e) fractional interaction histogram.

**Fig. 4:**
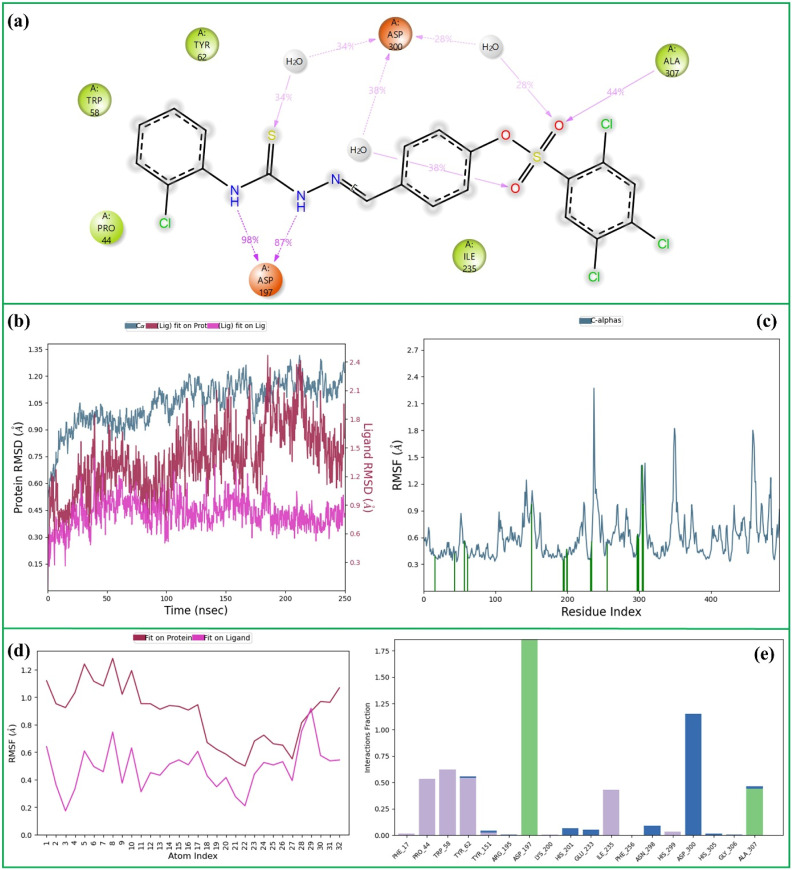
The 250 ns MD simulation analysis of 16-α-Amy complex. (a) 2D key ligand–protein interactions, (b) RMSD of ligand and protein atoms, (c) RMSF of protein atoms, (d) RMSF of ligand atoms, (e) fractional interaction histogram.

Molecular dynamics (MD) simulations provide detailed, time-resolved insights into the stability and conformational behavior of protein–ligand complexes at the atomic level, making them an essential tool in rational drug design. Key metrics such as Root Mean Square Deviation (RMSD) and Root Mean Square Fluctuation (RMSF) are commonly used to assess the overall structural stability and the flexibility of individual residues, respectively. While RMSD tracks the global deviations of the complex during the simulation, RMSF identifies highly mobile regions that could influence ligand binding and recognition.^53,54^ Building on the findings from *in vitro* inhibition assays and molecular docking studies, MD simulations were conducted for the most potent compound, 16, over a 250 ns trajectory. The resulting data, presented in Fig. 3 and 4, reveal the dynamic stability of the complex and provide further insight into the key interactions sustaining their binding.

During the 250 ns molecular dynamics simulation of the 16-α-Glu complex, a stable and extensive interaction network was maintained within the active site (Fig. 3a). The thioamide NH groups formed two highly persistent hydrogen bonds with Glu-276 (occupancies of 99% and 96%) and an additional hydrogen bond with Asp-214 (28%), stabilizing the ligand through strong polar interactions. The sulfonate oxygen contributed an additional hydrogen bond with Asn-241 (23%), while the sulfoxide oxygen engaged in water-mediated hydrogen bonds with His-239 and Glu-304 (each 28%), further reinforcing complex stability. Moreover, significant π–π stacking interactions were observed between the benzylidene and 2,4,5-trichlorophenyl rings with Phe-157 (31% and 25%), along with additional π–π stacking with His-348 (35%) and a cation–π interaction with Arg-312 (48%). These interactions demonstrate the strong binding affinity and conformational stability of compound 16 within the α-Glu active site, corroborating its potent inhibitory profile observed experimentally.

Throughout the simulation, the Cα atoms of the protein (pale blue) maintained an average RMSD of 1.2 Å, indicating a well-equilibrated and structurally stable backbone with negligible deviation over time. The ligand (red) also displayed remarkable stability, with an average RMSD of 1.5 Å, closely following the conformational behavior of the active-site residues. Moreover, the internal RMSD of the ligand (pink) remained consistently low at 0.6 Å, suggesting that its binding orientation was firmly retained with only minor thermal fluctuations (Fig. 3b). These observations collectively confirm that both the enzyme and compound 16 preserved their structural integrity and maintained a stable binding conformation throughout the MD trajectory.

Fig. 3c presents the RMSF profiles of the protein Cα atoms over the simulation period. The protein exhibited an average RMSF value of approximately 0.8 Å, reflecting a high degree of structural rigidity and limited residue-level fluctuations. These low fluctuation amplitudes suggest that the enzyme maintained a compact and conformationally stable backbone throughout the trajectory. The green vertical markers in Fig. 3c highlight nearly twenty persistent protein–ligand contacts, reinforcing the notion that 16 remained tightly bound within the active site during the entire course of the simulation.

Fig. 3d displays the RMSF profiles obtained from both fit-on-protein and fit-on-ligand alignments. The ligand exhibited an average RMSF of 0.8 Å when aligned to the protein (red), indicating that it moved in concert with the enzyme backbone and maintained a stable association within the binding pocket. Conversely, the lower RMSF value of 0.4 Å for the ligand aligned to itself (pink) suggests minimal internal flexibility, signifying that its conformation remained largely rigid during the simulation. Collectively, these results confirm the tight binding and conformational stability of 16 within the active site of α-Glu throughout the MD trajectory.

Fig. 3e presents the interaction histogram, illustrating the range and persistence of contacts between 16 and key residues within the enzyme's active site throughout the simulation. Hydrophobic interactions are represented in grey, conventional hydrogen bonds in green, and water-mediated hydrogen bonds in blue. The interactions are organized according to the ligand's functional domains, demonstrating that each structural moiety engages with multiple residues, while several amino acids interact with more than one ligand fragment. This comprehensive mapping highlights the dominant interaction network within the complex. Residues Tyr-71, Phe-157, Glu-276, Phe-300, and Arg-312 exhibited the highest interaction frequencies, underscoring their critical role in preserving the ligand's stable orientation and strong binding within the α-Glu active site.

Compound 16 also maintained a stable interaction network within the α-Amy active site throughout the simulation (Fig. 4a). The thioamide NH groups formed two strong hydrogen bonds with Asp-197 (98% and 87%), representing the most persistent contacts and suggesting a key anchoring role in the catalytic region. Additionally, the sulfoxide oxygen engaged in a hydrogen bond with Ala-307 (44%), reinforcing the ligand's orientation. Water-mediated hydrogen bonds with Asp-300, involving the thioamide sulfur (34%) and sulfoxide oxygens (38% and 28%), further contributed to the overall stability of the complex.

The Cα RMSD of 1.05 Å indicates that the overall backbone of α-Amy remained highly stable throughout the simulation, showing minimal structural deviation from its initial conformation. The ligand RMSD of 1.4 Å suggests that compound 16 maintained a steady binding pose within the active site, with only minor positional adjustments during the trajectory. Moreover, the low internal RMSD value of 0.9 Å reflects that the ligand itself preserved a rigid conformation without notable intramolecular distortion (Fig. 4b).

The protein displayed an average RMSF of 0.6 Å, indicating very limited residue-level mobility and a largely rigid backbone throughout the simulation (Fig. 4c). In addition, nearly twenty protein–ligand contacts were maintained over the trajectory, underscoring a dense and stable interaction network that likely contributes to the prolonged residence and high affinity of 16 within the binding pocket.

The ligand showed an average RMSF of 1.0 Å when aligned to the protein, suggesting that it moved in concert with the overall protein dynamics while remaining stably associated within the binding site. When aligned to itself, the RMSF decreased to 0.6 Å, reflecting minimal internal flexibility and a well-preserved conformation (Fig. 4d).

Fig. 4e displays the interaction histogram for 16 within the α-Amy active site over the course of the simulation. Among the interacting residues, Asp-197 and Asp-300 showed the highest contact frequencies, highlighting their essential role in anchoring the ligand and maintaining its stable orientation. This persistent interaction network supports the strong binding affinity and conformational stability of 16 within the enzyme's catalytic pocket.

To gain further insight into the contributions of different interaction types to the binding of compound 16, energy decomposition analysis was performed using MM-GBSA calculations. This analysis allows the dissection of the total binding free energy into individual components, highlighting the roles of van der Waals, electrostatic, hydrogen bonding, lipophilic, covalent, and solvation interactions in stabilizing the ligand within the enzyme active sites. The results were given in Table 3.

**Table 3:** Energy decomposition analysis of α-Glu-16 and α-Amy-16 complexes

Energy decomposition (kcal mol^−1^)	α-Glu-16	α-Amy-16
MM-GBSA Δ*G* bind. Coulomb	−20.53	−8.58
MM-GBSA Δ*G* bind. covalent	3.62	6.45
MM-GBSA Δ*G* bind. Hbond	−3.04	−2.12
MM-GBSA Δ*G* bind. Lipo	−28.86	−25.19
MM-GBSA Δ*G* bind. solv. GB	57.50	37.61
MM-GBSA Δ*G* bind. vdW	−61.49	−51.90

For the 16-α-Glu complex, the binding was primarily driven by van der Waals (−61.49 kcal mol^−1^) and lipophilic interactions (−28.86 kcal mol^−1^), with additional contributions from coulombic interactions (−20.53 kcal mol^−1^) and hydrogen bonds (−3.04 kcal mol^−1^). The polar solvation energy (57.50 kcal mol^−1^) partially offset these favorable interactions, while the covalent component was slightly unfavorable (3.62 kcal mol^−1^). Similarly, in the 16-α-Amy complex, strong van der Waals (−51.90 kcal mol^−1^) and lipophilic (−25.19 kcal mol^−1^) contributions dominated, supported by coulombic (−8.58 kcal mol^−1^) and hydrogen bonding (−2.12 kcal mol^−1^) interactions, with solvation effects (37.61 kcal mol^−1^) opposing binding. These results indicate that nonpolar interactions, particularly van der Waals and lipophilic forces, are the main contributors to the stable binding of compound 16, providing a mechanistic explanation for its high dual inhibitory potency observed *in vitro*.

As a result of the molecular dynamics simulations, compound 16 maintained stable binding conformations within both α-Glu and α-Amy active sites, supported by persistent hydrogen bonding, π–π, and halogen interactions. The low RMSD and RMSF values for both the protein backbones and the ligand indicate high structural stability, corroborating the strong inhibitory activity observed *in vitro*. Energy decomposition analysis further revealed that van der Waals and lipophilic interactions were the major contributors to binding, with additional support from coulombic and hydrogen bonding interactions, providing a mechanistic explanation for the compound's dual inhibitory potency.

### 2.5. ADME predictions

ADME profiling constitutes an essential aspect of contemporary drug discovery, providing early predictions of the pharmacokinetic properties of potential therapeutic agents. These *in silico* evaluations allow estimation of key parameters, including gastrointestinal absorption, systemic distribution, metabolic stability, and routes of excretion, before experimental testing. Additionally, such analyses can assess drug-likeness, oral bioavailability, blood–brain barrier penetration, and possible toxicity, aiding in the prioritization of promising candidates. Overall, ADME predictions offer valuable guidance for optimizing pharmacokinetic profiles and supporting rational drug design strategies.^55,56^ The predicted ADME properties of the synthesized compounds are summarized in Table 4.

**Table 4:** ADME prediction of the compounds 1–22 as weel as reference values^a^

Comp.	Ro5	Ro3	%HOA	QPPCaco	QPPMDCK	QPlogBB	QPlogPo/w	QPlogS	aHB	dHB	Mol MW
1	0	0	86	265	2770	−0.949	2.684	−5.459	9	2	438.73
2	0	1	100	822	9340	−0.421	3.561	−5.981	9	2	452.75
3	0	1	100	885	10 000	−0.461	4.177	−6.615	9	2	480.81
4	2	1	87	1302	10 000	−0.309	5.222	−7.902	9	2	520.87
5	0	1	100	935	9363	−0.590	4.641	−7.205	9	2	494.83
6	1	1	92	721	10 000	−0.669	4.590	−7.317	9	2	512.87
7	2	1	88	1087	10 000	−0.556	5.725	−8.114	9	2	542.88
8	2	1	83	770	9625	−0.676	5.194	−7.796	9	2	528.85
9	2	1	84	785	10 000	−0.559	5.425	−8.131	9	2	546.84
10	1	1	94	812	7711	−0.642	4.872	−7.560	9	2	514.82
11	2	1	83	782	7786	−0.652	5.299	−8.187	9	2	542.88
12	2	1	84	910	8374	−0.559	5.259	−7.903	9	2	542.88
13	2	1	85	902	9087	−0.613	5.374	−7.869	9	2	542.88
14	1	1	93	686	10 000	−0.597	4.932	−7.634	9	2	532.81
15	2	1	83	848	10 000	−0.500	5.120	−7.878	9	2	532.81
16	2	1	87	1084	10 000	−0.334	5.476	−8.246	9	2	549.27
17	2	1	86	921	10 000	−0.415	5.483	−8.450	9	2	549.27
18	2	1	84	812	10 000	−0.491	5.357	−8.283	9	2	549.27
19	2	1	84	812	10 000	−0.482	5.432	−8.394	9	2	593.72
20	1	1	93	746	6354	−0.775	4.866	−7.580	9	2	544.85
21	1	1	94	774	7093	−0.742	4.897	−7.620	9	2	544.85
22	1	1	73	91	698	−1.953	4.121	−7.643	10	2	559.82

a MW 130 to 725 (molecular weight); dHB 0 to 6 (H-bond donors); aHB 2 to 20 (H-bond acceptors); QPlogPo/w −2 to 6.5 (octanol/water partition coefficient); QPlogS −6.5 to 0.5 (aqueous solubility, log *S*); QPPCaco <25 poor, >500 great (intestinal permeability); QPlogBB −3 to 1.2 (brain/blood partition); QPPMDCK <25 poor, >500 great (BBB permeability); %HOA >80 high, <25 poor (oral absorption); Ro5 ≤4 (Lipinski); Ro3 ≤3 (Jorgensen).

The predicted ADME profiles of the synthesized compounds indicate generally favorable pharmacokinetic properties. Most compounds complied with Lipinski's rule of five (Ro5 ≤ 4) and Jorgensen's rule of three (Ro3 ≤ 3), suggesting good drug-likeness. Lipinski's Ro5 is a widely used guideline to evaluate the drug-likeness of small molecules, predicting oral bioavailability based on molecular weight, lipophilicity (log *P*), and hydrogen bond donors and acceptors. Compounds violating more than one of these criteria may exhibit reduced oral absorption. Jorgensen's Ro3 provides additional criteria for lead-likeness, particularly in early-stage drug discovery, focusing on solubility, permeability, and metabolic stability.

Oral absorption (%HOA) was high (>80%) for the majority of compounds, with compounds 2, 3, 5–7, 10, 14, 16, 21, and 22 showing particularly strong predicted absorption. Intestinal permeability (QPPCaco) values were favorable for most derivatives (>500 nm s^−1^), indicating potential for efficient gastrointestinal uptake, whereas compound 22 showed notably poor permeability (91 nm s^−1^). The blood–brain barrier (QPlogBB) predictions were generally negative (−0.95 to −0.33), consistent with limited CNS penetration, which may reduce central side effects. Lipophilicity (QPlogPo/w) ranged from 2.68 to 5.48, suggesting adequate membrane permeability, while aqueous solubility (QPlogS) values were within acceptable limits (−5.45 to −8.45), indicating moderate to low solubility. These *in silico* ADME results suggest that most of the synthesized thiosemicarbazone derivatives possess drug-like characteristics suitable for further development.

## 3. Conclusion

In this study, a novel series of twenty-two thiosemicarbazone derivatives featuring a 2,4,5-trichlorobenzenesulfonate scaffold were synthesized and fully characterized by ^1^H, ^13^C, and ^19^F NMR, FTIR, and HRMS spectroscopies. The *in vitro* evaluation demonstrated that several compounds possessed potent dual inhibitory activity against α-Glu and α-Amy, with compound 16 emerging as the most active, surpassing the reference drug acarbose in both enzyme assays. The SAR analysis indicated that substituents at the thioamide moiety critically influenced activity, with aromatic halogenated groups and hydrophobic moieties enhancing enzyme binding.

Molecular docking studies revealed that compound 16 formed multiple stabilizing interactions, including hydrogen bonds, π–π stacking, cation–π, and halogen bonds, with key active site residues in both α-Glu and α-Amy. The IFD and MM-GBSA analyses further supported strong binding affinities, while MD simulations over 250 ns confirmed the stability of these interactions, as evidenced by low RMSD and RMSF values and persistent contacts throughout the trajectories. Energy decomposition analysis highlighted that van der Waals and lipophilic interactions were the predominant contributors to binding, with additional stabilization provided by coulombic and hydrogen bonding interactions.

*In silico* ADME predictions indicated that several synthesized derivatives exhibit acceptable pharmacokinetic properties, including good intestinal permeability, moderate lipophilicity, and reasonable solubility, although deviations from Lipinski's rule were observed for some bulky analogs. Limited blood–brain barrier penetration was predicted for most compounds, which may reduce the risk of central nervous system-related side effects. When considered together with the *in vitro* enzyme inhibition data, these findings suggest that the synthesized thiosemicarbazone derivatives represent promising lead structures for dual α-Glu/α-Amy inhibition. In particular, compound 16 demonstrated the highest inhibitory potency, while compound 2 combined strong α-Glu inhibition with full compliance to Lipinski's criteria, highlighting complementary profiles for further optimization and investigation.

While the present study provides an initial *in vitro* and *in silico* assessment of dual α-glucosidase and α-amylase inhibition, it is limited to enzymatic assays and computational analyses. Therefore, the findings should be interpreted as preliminary. Building on the observed inhibitory profiles and computational insights, future studies should focus on *in vivo* evaluation of the most active derivatives, along with structural optimization to improve selectivity and pharmacokinetic behavior. Further investigation is also required to assess safety, metabolic stability, and efficacy in more biologically relevant systems. In addition, advanced computational approaches, such as free energy perturbation and enhanced sampling molecular dynamics simulations, may offer deeper insight into ligand–enzyme interactions and support the rational design of next-generation inhibitors.

## 4. Material and methods

### 4.1. Chemistry

All chemicals used in this study were obtained from various commercial suppliers. Melting points of the synthesized compounds were measured using a WRS-2A Microprocessor Melting-point Apparatus and are reported without correction. ^1^H NMR spectra were acquired using a Bruker 400 MHz spectrometer, while ^13^C NMR spectra were obtained on a Bruker 101 MHz instrument. Chemical shifts are reported in *δ* (ppm) relative to tetramethylsilane (TMS, *δ* 0.00 singlet) using deuterated dimethyl sulfoxide (DMSO-*d*_6_) as solvents.

#### 4.1.1. Synthesis of sulfonylated aldehyde (SA)

To a solution of 4-hydroxybenzaldehyde (10 mmol) in anhydrous DMF (10 mL) 2,4,5-trichlorobenzenesulfonyl chloride (10 mmol) was added and the mixture was put in an ice bath. Triethylamine (11 mmol) was added to this solution dropwise and the final mixture was stirred at 0–5 °C for 1 hour.^57^ Then the mixture was poured onto water (100 mL) and the formed precipitate was filtered off. The crude product (SA) was recrystallized from ethanol (Scheme 2).

#### 4.1.2. Synthesis of compounds 1–22

Sulfonylated aldehyde (SA) (10 mmol) was dissolved in absolute ethanol (20 mL) and glacial acetic acid (3–4 drops) was added to this solution as a catalyst. The thiosemicarbazide derivative (10 mmol) was added to this solution and the mixture was refluxed for 3–6 hours.^58^ Then the mixture was cooled to room temperature and the formed crude product was filtered off and recrystallized from ethanol (Scheme 2).

##### 4.1.2.1 (*E*)-4-((2-Carbamothioylhydrazineylidene)methyl)phenyl 2,4,5-trichlorobenzenesulfonate (1)

White solid, yield: 89%, mp: 220–222 °C. FTIR (cm^−1^) 3409, 3234, 3119, 2988, 1596, 1387, 1146, 1063. ^1^H NMR (400 MHz, DMSO) *δ* 11.50 (s, 1H), 8.36 (s, 1H), 8.24 (s, 1H), 8.08 (s, 1H), 8.05 (s, 1H), 8.02 (s, 1H), 7.87 (d, *J* = 8.8 Hz, 2H), 7.18 (d, *J* = 8.8 Hz, 2H). ^13^C NMR (101 MHz, DMSO) *δ* 178.7, 149.6, 140.8, 139.7, 134.6, 134.5, 133.2, 132.8, 131.8, 131.8, 129.6, 122.5. HRMS-ESI (*m*/*z*): chemical formula: C_14_H_10_Cl_3_N_3_O_3_S_2_, calculated [M + H]^+^: 437.9307, found [M + H]^+^: 437.9296.

##### 4.1.2.2 (*E*)-4-((2-(Methylcarbamothioyl)hydrazineylidene)methyl)phenyl 2,4,5-trichlorobenzenesulfonate (2)

Off-white solid, yield: 88%, mp: 249–251 °C. FTIR (cm^−1^) 3347, 3148, 3081, 2987, 1599, 1358, 1138, 1064. ^1^H NMR (400 MHz, DMSO) *δ* 11.55 (s, 1H), 8.55 (d, *J* = 4.5 Hz, 1H), 8.36 (s, 1H), 8.08 (s, 1H), 8.01 (s, 1H), 7.86 (d, *J* = 8.8 Hz, 2H), 7.19 (d, *J* = 8.8 Hz, 2H), 3.01 (d, *J* = 4.5 Hz, 3H). ^13^C NMR (101 MHz, DMSO) *δ* 178.4, 149.6, 140.2, 139.7, 134.6, 133.2, 132.8, 131.8, 131.8, 129.5, 122.5, 31.3. HRMS-ESI (*m*/*z*): chemical formula: C_15_H_13_Cl_3_N_3_O_3_S_2_, calculated [M + H]^+^: 451.9464, found [M + H]^+^: 451.9454.

##### 4.1.2.3 (*E*)-4-((2-(Isopropylcarbamothioyl)hydrazineylidene)methyl)phenyl 2,4,5-trichlorobenzenesulfonate (3)

Yellow solid, yield: 91%, mp: 193–195 °C. FTIR (cm^−1^) 3343, 3151, 3092, 2971, 1599, 1371, 1139, 1065. ^1^H NMR (400 MHz, DMSO) *δ* 11.47 (s, 1H), 8.36 (s, 1H), 8.09 (s, 1H), 8.07 (s, 1H), 8.03 (s, 1H), 7.86 (d, *J* = 8.8 Hz, 2H), 7.20 (d, *J* = 8.7 Hz, 2H), 4.53 (sext, *J* = 6.6 Hz, 1H), 1.22 (d, *J* = 6.6 Hz, 6H). ^13^C NMR (101 MHz, DMSO) *δ* 176.4, 149.6, 140.7, 139.7, 134.6, 134.4, 133.2, 132.8, 131.8, 129.6, 122.5, 46.1, 22.3. HRMS-ESI (*m*/*z*): chemical formula: C_17_H_17_Cl_3_N_3_O_3_S_2_, calculated [M + H]^+^: 479.9777, found [M + H]^+^: 479.9769.

##### 4.1.2.4 (*E*)-4-((2-(Cyclohexylcarbamothioyl)hydrazineylidene)methyl)phenyl 2,4,5-trichlorobenzenesulfonate (4)

White solid, yield: 85%, mp: 203–205 °C. FTIR (cm^−1^) 3318, 3143, 3092, 2987, 1532, 1385, 1142, 1061. ^1^H NMR (400 MHz, DMSO) *δ* 11.47 (s, 1H), 8.36 (s, 1H), 8.07–8.03 (m, 3H), 7.85 (d, *J* = 8.8 Hz, 2H), 7.19 (d, *J* = 8.7 Hz, 2H), 4.23–4.14 (m, 1H), 1.87 (d, *J* = 9.7 Hz, 2H), 1.73 (d, *J* = 12.8 Hz, 2H), 1.61 (d, *J* = 12.4 Hz, 1H), 1.42 (dd, *J* = 26.4, 12.1 Hz, 2H), 1.28 (dd, *J* = 25.2, 12.5 Hz, 2H), 1.14 (t, *J* = 12.4 Hz, 1H). ^13^C NMR (101 MHz, DMSO) *δ* 176.3, 149.6, 140.7, 139.7, 134.6, 134.4, 133.2, 132.8, 131.8, 129.6, 122.5, 53.2, 32.2, 25.6, 25.4. HRMS-ESI (*m*/*z*): chemical formula: C_20_H_21_Cl_3_N_3_O_3_S_2_, calculated [M + H]^+^: 520.0090, found [M + H]^+^: 520.0081.

##### 4.1.2.5 (*E*)-4-((2-(Isobutylcarbamothioyl)hydrazineylidene)methyl)phenyl 2,4,5-trichlorobenzenesulfonate (5)

White solid, yield: 92%, mp: 178–180 °C. FTIR (cm^−1^) 3358, 3137, 3087, 2953, 1532, 1392, 1136, 1066. ^1^H NMR (400 MHz, DMSO) *δ* 11.50 (s, 1H), 8.54 (t, *J* = 5.9 Hz, 1H), 8.36 (s, 1H), 8.08 (s, 1H), 8.03 (s, 1H), 7.86 (d, *J* = 8.8 Hz, 2H), 7.19 (d, *J* = 8.7 Hz, 2H), 3.39 (t, *J* = 6.5 Hz, 2H), 2.01 (sept, *J* = 6.8 Hz, 1H), 0.88 (d, *J* = 6.8 Hz, 6H). ^13^C NMR (101 MHz, DMSO) *δ* 177.9, 149.6, 140.4, 139.7, 134.6, 134.5, 133.2, 132.8, 131.8, 131.8, 129.5, 122.5, 51.3, 28.3, 20.6. HRMS-ESI (*m*/*z*): chemical formula: C_18_H_19_Cl_3_N_3_O_3_S_2_, calculated [M + H]^+^: 493.9933, found [M + H]^+^: 493.9924.

##### 4.1.2.6 (*E*)-4-((2-((2-(Methylthio)propyl)carbamothioyl)hydrazineylidene)methyl)phenyl 2,4,5-trichlorobenzenesulfonate (6)

Off-white solid, yield: 92%, mp: 158–160 °C. FTIR (cm^−1^) 3345, 3137, 2994, 1538, 1354, 1136, 1066. ^1^H NMR (400 MHz, DMSO) *δ* 11.53 (s, 1H), 8.62 (t, *J* = 5.8 Hz, 1H), 8.36 (s, 1H), 8.08 (s, 1H), 8.02 (s, 1H), 7.86 (d, *J* = 8.8 Hz, 2H), 7.19 (d, *J* = 8.8 Hz, 2H), 3.63 (dd, *J* = 13.8, 6.5 Hz, 2H), 2.06 (s, 3H), 1.90–1.83 (m, 2H). ^13^C NMR (101 MHz, DMSO) *δ* 177.7, 149.6, 140.5, 139.7, 134.6, 134.5, 133.2, 132.8, 131.8, 131.8, 129.5, 122.5, 43.2, 31.2, 28.7, 15.1. HRMS-ESI (*m*/*z*): chemical formula: C_18_H_18_Cl_3_N_3_O_3_S_3_, calculated [M + H]^+^: 525.9654, found [M + H]^+^: 525.9646.

##### 4.1.2.7 (*E*)-4-((2-(Phenethylcarbamothioyl)hydrazineylidene)methyl)phenyl 2,4,5-trichlorobenzenesulfonate (7)

Off-white solid, yield: 91%, mp: 197–199 °C. FTIR (cm^−1^) 3332, 3188, 3089, 3000, 1600, 1379, 1139, 1066. ^1^H NMR (400 MHz, DMSO) *δ* 11.59 (s, 1H), 8.60 (t, *J* = 5.8 Hz, 1H), 8.38 (s, 1H), 8.09 (s, 1H), 8.02 (s, 1H), 7.83 (d, *J* = 8.8 Hz, 2H), 7.33–7.22 (m, 5H), 7.19 (d, *J* = 8.8 Hz, 2H), 3.75 (dd, *J* = 15.5, 6.0 Hz, 2H), 2.91 (t, *J* = 6.0 Hz, 2H). ^13^C NMR (101 MHz, DMSO) *δ* 177.6, 149.7, 140.5, 139.7, 139.7, 134.6, 134.5, 133.2, 132.8, 131.8, 131.8, 129.5, 129.1, 128.9, 126.7, 122.5, 45.5, 35.3. HRMS-ESI (*m*/*z*): chemical formula: C_22_H_19_Cl_3_N_3_O_3_S_2_, calculated [M + H]^+^: 541.9933, found [M + H]^+^: 541.9923.

##### 4.1.2.8 (*E*)-4-((2-(Benzylcarbamothioyl)hydrazineylidene)methyl)phenyl 2,4,5-trichlorobenzenesulfonate (8)

Light yellow solid, yield: 93%, mp: 176–178 °C. FTIR (cm^−1^) 3356, 3130, 3086, 2988, 1600, 1385, 1145, 1064. ^1^H NMR (400 MHz, DMSO) *δ* 11.67 (s, 1H), 9.13 (t, *J* = 6.2 Hz, 1H), 8.35 (s, 1H), 8.06 (d, *J* = 5.2 Hz, 2H), 7.88 (d, *J* = 8.7 Hz, 2H), 7.35–7.30 (m, 4H), 7.25–7.22 (m, 1H), 7.18 (d, *J* = 8.7 Hz, 2H), 4.84 (d, *J* = 6.2 Hz, 2H). ^13^C NMR (101 MHz, DMSO) *δ* 178.2, 149.7, 140.8, 139.8, 139.7, 134.6, 134.5, 133.2, 132.8, 131.8, 129.6, 128.6, 127.7, 127.2, 122.5, 47.1. HRMS-ESI (*m*/*z*): chemical formula: C_21_H_17_Cl_3_N_3_O_3_S_2_, calculated [M + H]^+^: 527.9777, found [M + H]^+^: 527.9766.

##### 4.1.2.9 (*E*)-4-((2-((4-Fluorobenzyl)carbamothioyl)hydrazineylidene)methyl)phenyl 2,4,5-trichlorobenzenesulfonate (9)

White solid, yield: 86%, mp: 209–211 °C. FTIR (cm^−1^) 3355, 3134, 3094, 2989, 1601, 1387, 1149, 1067. ^1^H NMR (400 MHz, DMSO) *δ* 11.68 (s, 1H), 9.14 (t, *J* = 6.2 Hz, 1H), 8.35 (s, 1H), 8.07 (s, 1H), 8.05 (s, 1H), 7.88 (d, *J* = 8.8 Hz, 2H), 7.39 (dd, *J* = 8.5, 5.7 Hz, 2H), 7.19–7.13 (m, 4H), 4.81 (d, *J* = 6.2 Hz, 2H). ^13^C NMR (101 MHz, DMSO) *δ* 178.2, 161.6 (d, *J* = 242 Hz), 149.7, 140.9, 139.7, 136.0 (d, *J* = 2.9 Hz), 134.6, 134.5, 133.2, 132.8, 131.79 (d, *J* = 2.1 Hz), 129.7, 129.6, 129.6, 122.5, 115.3 (d, *J* = 21.3 Hz), 46.3. ^19^F NMR (471 MHz, DMSO) *δ* −116.20. HRMS-ESI (*m*/*z*): chemical formula: C_21_H_16_Cl_3_FN_3_O_3_S_2_, calculated [M + H]^+^: 545.9683, found [M + H]^+^: 545.9670.

##### 4.1.2.10 (*E*)-4-((2-(Phenylcarbamothioyl)hydrazineylidene)methyl)phenyl 2,4,5-trichlorobenzenesulfonate (10)

White solid, yield: 89%, mp: 236–238 °C. FTIR (cm^−1^) 3277, 3155, 3086, 2980, 1594, 1391, 1136, 1065. ^1^H NMR (400 MHz, DMSO) *δ* 11.89 (s, 1H), 10.13 (s, 1H), 8.36 (s, 1H), 8.13 (s, 1H), 8.08 (s, 1H), 7.97 (d, *J* = 8.8 Hz, 2H), 7.55 (d, *J* = 7.5 Hz, 2H), 7.37 (t, *J* = 7.8 Hz, 2H), 7.23–7.18 (m, 3H). ^13^C NMR (101 MHz, DMSO) *δ* 176.7, 149.8, 141.4, 139.7, 139.5, 134.6, 134.3, 133.2, 132.8, 131.8, 131.8, 130.0, 128.5, 126.3, 125.9, 122.5. HRMS-ESI (*m*/*z*): chemical formula: C_20_H_15_Cl_3_N_3_O_3_S_2_, calculated [M + H]^+^: 513.9620, found [M + H]^+^: 513.9612.

##### 4.1.2.11 (*E*)-4-((2-((2,4-Dimethylphenyl)carbamothioyl)hydrazineylidene)methyl)phenyl 2,4,5-trichlorobenzenesulfonate (11)

Light yellow solid, yield: 88%, mp: 226–228 °C. FTIR (cm^−1^) 3216, 3089, 2976, 1595, 1361, 1135, 1065. ^1^H NMR (400 MHz, DMSO) *δ* 11.80 (s, 1H), 9.93 (s, 1H), 8.36 (s, 1H), 8.09 (s, 1H), 8.08 (s, 1H), 7.96 (d, *J* = 8.8 Hz, 2H), 7.17 (d, *J* = 8.8 Hz, 2H), 7.12–7.08 (m, 2H), 7.02 (d, *J* = 7.9 Hz, 1H), 2.29 (s, 3H), 2.17 (s, 3H). ^13^C NMR (101 MHz, DMSO) *δ* 177.6, 149.7, 140.9, 139.7, 136.3, 135.9, 135.6, 134.6, 134.5, 133.2, 132.8, 131.8, 131.1, 129.8, 129.0, 126.9, 122.4, 21.1, 18.2. HRMS-ESI (*m*/*z*): chemical formula: C_22_H_19_Cl_3_N_3_O_3_S_2_, calculated [M + H]^+^: 541.9933, found [M + H]^+^: 541.9923.

##### 4.1.2.12 (*E*)-4-((2-((2,6-Dimethylphenyl)carbamothioyl)hydrazineylidene)methyl)phenyl 2,4,5-trichlorobenzenesulfonate (12)

Off-white solid, yield: 87%, mp: 228–230 °C. FTIR (cm^−1^) 3330, 3146, 3092, 2989, 1601, 1379, 1148, 1066. ^1^H NMR (400 MHz, DMSO) *δ* 11.81 (s, 1H), 9.91 (s, 1H), 8.36 (s, 1H), 8.09 (s, 2H), 7.98 (d, *J* = 8.8 Hz, 2H), 7.17 (d, *J* = 8.8 Hz, 2H), 7.12–7.09 (m, 3H), 2.18 (s, 6H). ^13^C NMR (101 MHz, DMSO) *δ* 177.4, 149.7, 140.8, 139.7, 137.5, 136.9, 134.6, 133.2, 132.8, 131.8, 129.8, 128.0, 127.4, 122.4, 18.4. HRMS-ESI (*m*/*z*): chemical formula: C_22_H_19_Cl_3_N_3_O_3_S_2_, calculated [M + H]^+^: 541.9933, found [M + H]^+^: 541.9933.

##### 4.1.2.13 (*E*)-4-((2-((2-Ethylphenyl)carbamothioyl)hydrazineylidene)methyl)phenyl 2,4,5-trichlorobenzenesulfonate (13)

Off-white solid, yield: 92%, mp: 210–212 °C. FTIR (cm^−1^) 3319, 3128, 3092, 2969, 1602, 1385, 1143, 1065. ^1^H NMR (400 MHz, DMSO) *δ* 11.85 (s, 1H), 10.01 (s, 1H), 8.36 (s, 1H), 8.10 (s, 1H), 8.09 (s, 1H), 7.97 (d, *J* = 8.8 Hz, 2H), 7.30–7.24 (m, 4H), 7.18 (d, *J* = 8.8 Hz, 2H), 2.59 (q, *J* = 7.6 Hz, 2H), 1.14 (t, *J* = 7.6 Hz, 3H). ^13^C NMR (101 MHz, DMSO) *δ* 177.9, 149.7, 141.6, 141.0, 139.7, 138.0, 134.6, 134.5, 133.2, 132.8, 131.8, 131.8, 129.8, 129.7, 128.7, 127.5, 126.3, 122.4, 24.5, 14.6. HRMS-ESI (*m*/*z*): chemical formula: C_22_H_19_Cl_3_N_3_O_3_S_2_, calculated [M + H]^+^: 541.9933, found [M + H]^+^: 541.9923.

##### 4.1.2.14 (*E*)-4-((2-((2-Fluorophenyl)carbamothioyl)hydrazineylidene)methyl)phenyl 2,4,5-trichlorobenzenesulfonate (14)

Orange solid, yield: 88%, mp: 218–220 °C. FTIR (cm^−1^) 3269, 3155, 3084, 2962, 1595, 1392, 1138, 1065. ^1^H NMR (400 MHz, DMSO) *δ* 12.03 (s, 1H), 10.01 (s, 1H), 8.36 (s, 1H), 8.12 (s, 1H), 8.09 (s, 1H), 7.96 (d, *J* = 8.8 Hz, 2H), 7.48 (t, *J* = 7.8 Hz, 1H), 7.35–7.22 (m, 3H), 7.20 (d, *J* = 8.7 Hz, 2H). ^13^C NMR (101 MHz, DMSO) *δ* 178.0, 157.9 (d, *J* = 247 Hz), 149.8, 141.6, 139.7, 134.6, 133.8 (d, *J* = 2.8 Hz), 132.8, 131.8, 131.8, 130.8, 129.9, 128.8, 128.4 (d, *J* = 8 Hz), 127.5, 124.5, 122.5, 116.2 (d, *J* = 19.9 Hz). ^19^F NMR (471 MHz, DMSO) *δ* −120.76. HRMS-ESI (*m*/*z*): chemical formula: C_20_H_14_Cl_3_FN_3_O_3_S_2_, calculated [M + H]^+^: 531.9526, found [M + H]^+^: 531.9517.

##### 4.1.2.15 (*E*)-4-((2-((4-Fluorophenyl)carbamothioyl)hydrazineylidene)methyl)phenyl 2,4,5-trichlorobenzenesulfonate (15)

Orange solid, yield: 87%, mp: 246–248 °C. FTIR (cm^−1^) 3277, 3161, 3087, 2983, 1606, 1355, 1136, 1068. ^1^H NMR (400 MHz, DMSO) *δ* 11.92 (s, 1H), 10.14 (s, 1H), 8.37 (s, 1H), 8.12 (s, 1H), 8.08 (s, 1H), 7.97 (d, *J* = 8.8 Hz, 2H), 7.53 (dd, *J* = 8.9, 5.1 Hz, 2H), 7.23–7.18 (m, 4H). ^13^C NMR (101 MHz, DMSO) *δ* 177.0, 160.2 (d, *J* = 242.3 Hz), 149.8, 141.5, 139.7, 135.8 (d, *J* = 2.8 Hz), 134.6, 134.3, 133.2, 132.8, 131.8, 131.8, 130.0, 128.6 (d, *J* = 8.6 Hz), 122.5, 115.2 (d, *J* = 22.3 Hz). ^19^F NMR (471 MHz, DMSO) *δ* −117.00. HRMS-ESI (*m*/*z*): chemical formula: C_20_H_14_Cl_3_FN_3_O_3_S_2_, calculated [M + H]^+^: 531.9526, found [M + H]^+^: 531.9518.

##### 4.1.2.16 (*E*)-4-((2-((2-Chlorophenyl)carbamothioyl)hydrazineylidene)methyl)phenyl 2,4,5-trichlorobenzenesulfonate (16)

Off-white solid, yield: 89%, mp: 208–210 °C. FTIR (cm^−1^) 3319, 3121, 3091, 2970, 1598, 1384, 1144, 1065. ^1^H NMR (400 MHz, DMSO) *δ* 12.05 (s, 1H), 10.12 (s, 1H), 8.36 (s, 1H), 8.13 (s, 1H), 8.09 (s, 1H), 7.95 (d, *J* = 8.7 Hz, 2H), 7.65 (d, *J* = 7.7 Hz, 1H), 7.55 (d, *J* = 7.9 Hz, 1H), 7.39 (t, *J* = 7.0 Hz, 1H), 7.32 (t, *J* = 7.6 Hz, 1H), 7.21 (d, *J* = 8.7 Hz, 2H). ^13^C NMR (101 MHz, DMSO) *δ* 177.4, 149.8, 141.6, 139.7, 137.0, 134.6, 134.3, 133.2, 132.8, 131.8, 131.3, 130.5, 129.9, 129.8, 128.4, 127.6, 122.6. HRMS-ESI (*m*/*z*): chemical formula: C_20_H_14_Cl_4_N_3_O_3_S_2_, calculated [M + H]^+^: 547.9231, found [M + H]^+^: 547.9227.

##### 4.1.2.17 (*E*)-4-((2-((3-Chlorophenyl)carbamothioyl)hydrazineylidene)methyl)phenyl 2,4,5-trichlorobenzenesulfonate (17)

Off-white solid, yield: 94%, mp: 223–225 °C. FTIR (cm^−1^) 3276, 3142, 3093, 2978, 1585, 1388, 1134, 1065. ^1^H NMR (400 MHz, DMSO) *δ* 12.02 (s, 1H), 10.19 (s, 1H), 8.37 (s, 1H), 8.14 (s, 1H), 8.08 (s, 1H), 7.98 (d, *J* = 8.8 Hz, 2H), 7.74 (s, 1H), 7.60 (d, *J* = 8.2 Hz, 1H), 7.40 (t, *J* = 8.1 Hz, 1H), 7.27 (d, *J* = 9.9 Hz, 1H), 7.21 (d, *J* = 8.8 Hz, 2H). ^13^C NMR (101 MHz, DMSO) *δ* 176.5, 149.9, 142.0, 140.9, 139.7, 134.6, 134.2, 133.2, 132.8, 132.6, 131.8, 131.8, 130.1, 125.6, 125.5, 124.6, 122.5. HRMS-ESI (*m*/*z*): chemical formula: C_20_H_14_Cl_4_N_3_O_3_S_2_, calculated [M + H]^+^: 547.9231, found [M + H]^+^: 547.9226.

##### 4.1.2.18 (*E*)-4-((2-((4-Chlorophenyl)carbamothioyl)hydrazineylidene)methyl)phenyl 2,4,5-trichlorobenzenesulfonate (18)

Off-white solid, yield: 90%, mp: 241–243 °C. FTIR (cm^−1^) 3283, 3142, 3090, 2981, 1592, 1358, 1137, 1068. ^1^H NMR (400 MHz, DMSO) *δ* 11.97 (s, 1H), 10.16 (s, 1H), 8.37 (s, 1H), 8.13 (s, 1H), 8.08 (s, 1H), 7.97 (d, *J* = 8.8 Hz, 2H), 7.60 (d, *J* = 8.8 Hz, 2H), 7.42 (d, *J* = 8.7 Hz, 2H), 7.20 (d, *J* = 8.8 Hz, 2H). ^13^C NMR (101 MHz, DMSO) *δ* 176.6, 149.9, 141.8, 139.7, 138.5, 134.6, 134.2, 133.2, 132.8, 131.8, 130.0, 129.9, 128.4, 127.9, 122.5. HRMS-ESI (*m*/*z*): chemical formula: C_20_H_14_Cl_4_N_3_O_3_S_2_, calculated [M + H]^+^: 547.9231, found [M + H]^+^: 547.9220.

##### 4.1.2.19 (*E*)-4-((2-((4-Bromophenyl)carbamothioyl)hydrazineylidene)methyl)phenyl 2,4,5-trichlorobenzenesulfonate (19)

Off-white solid, yield: 89%, mp: 235–237 °C. FTIR (cm^−1^) 3284, 3129, 2978, 1589, 1359, 1138, 1067. ^1^H NMR (400 MHz, DMSO) *δ* 11.98 (s, 1H), 10.16 (s, 1H), 8.37 (s, 1H), 8.13 (s, 1H), 8.08 (s, 1H), 7.97 (d, *J* = 8.8 Hz, 2H), 7.55 (s, 4H), 7.20 (d, *J* = 8.8 Hz, 2H). ^13^C NMR (101 MHz, DMSO) *δ* 176.6, 149.9, 141.8, 139.7, 138.9, 134.6, 134.2, 133.2, 132.8, 131.8, 131.4, 130.0, 128.2, 122.5, 118.1. HRMS-ESI (*m*/*z*): chemical formula: C_20_H_14_Cl_3_BrN_3_O_3_S_2_, calculated [M + H]^+^: 591.8726, found [M + H]^+^: 591.8553.

##### 4.1.2.20 (*E*)-4-((2-((3-Methoxyphenyl)carbamothioyl)hydrazineylidene)methyl)phenyl 2,4,5-trichlorobenzenesulfonate (20)

White solid, yield: 87%, mp: 181–183 °C. FTIR (cm^−1^) 3273, 3091, 2942, 1593, 1356, 1153, 1068. ^1^H NMR (400 MHz, DMSO) *δ* 11.90 (s, 1H), 10.08 (s, 1H), 8.37 (s, 1H), 8.12 (s, 1H), 8.08 (s, 1H), 7.97 (d, *J* = 8.8 Hz, 2H), 7.29–7.25 (m, 2H), 7.21–7.16 (m, 3H), 6.79 (dd, *J* = 7.8, 2.2 Hz, 1H), 3.77 (s, 3H). ^13^C NMR (101 MHz, DMSO) *δ* 176.4, 159.5, 149.8, 141.5, 140.5, 139.7, 134.6, 134.3, 133.2, 132.8, 131.8, 130.0, 129.2, 122.5, 118.3, 111.8, 111.3, 55.6. HRMS-ESI (*m*/*z*): chemical formula: C_21_H_17_Cl_3_N_3_O_4_S_2_, calculated [M + H]^+^: 543.9726, found [M + H]^+^: 543.9716.

##### 4.1.2.21 (*E*)-4-((2-((4-Methoxyphenyl)carbamothioyl)hydrazineylidene)methyl)phenyl 2,4,5-trichlorobenzenesulfonate (21)

White solid, yield: 91%, mp: 208–210 °C. FTIR (cm^−1^) 3293, 3130, 2984, 1594, 1359, 1140, 1064. ^1^H NMR (400 MHz, DMSO) *δ* 11.81 (s, 1H), 10.04 (s, 1H), 8.37 (s, 1H), 8.10 (s, 1H), 8.08 (s, 1H), 7.97 (d, *J* = 8.6 Hz, 2H), 7.38 (d, *J* = 8.7 Hz, 2H), 7.19 (d, *J* = 8.6 Hz, 2H), 6.93 (d, *J* = 8.8 Hz, 2H), 3.77 (s, 3H). ^13^C NMR (101 MHz, DMSO) *δ* 177.0, 157.5, 149.7, 141.1, 139.7, 134.6, 134.4, 133.2, 132.8, 132.3, 131.8, 129.9, 128.0, 122.4, 113.7, 55.7. HRMS-ESI (*m*/*z*): chemical formula: C_21_H_17_Cl_3_N_3_O_4_S_2_, calculated [M + H]^+^: 543.9726, found [M + H]^+^: 543.9717.

##### 4.1.2.22 (*E*)-4-((2-((4-Nitrophenyl)carbamothioyl)hydrazineylidene)methyl)phenyl 2,4,5-trichlorobenzenesulfonate (22)

Yellow solid, yield: 85%, mp: 247–249 °C. FTIR (cm^−1^) 3270, 3150, 3087, 2980, 1597, 1555, 1360, 1330, 1136, 1069. ^1^H NMR (400 MHz, DMSO) *δ* 12.24 (s, 1H), 10.44 (s, 1H), 8.37 (s, 1H), 8.25 (d, *J* = 9.2 Hz, 2H), 8.18 (s, 1H), 8.08 (s, 1H), 8.06 (d, *J* = 9.2 Hz, 2H), 7.99 (d, *J* = 8.8 Hz, 2H), 7.23 (d, *J* = 8.8 Hz, 2H). ^13^C NMR (101 MHz, DMSO) *δ* 176.0, 150.0, 145.8, 144.0, 142.7, 139.7, 134.6, 134.0, 133.2, 132.7, 131.8, 130.2, 124.9, 124.2, 122.5. HRMS-ESI (*m*/*z*): chemical formula: C_20_H_14_Cl_3_N_4_O_5_S_2_, calculated [M + H]^+^: 558.9471, found [M + H]^+^: 558.9297.

### 4.2. *In vitro* enzyme inhibition

#### 4.2.1. α-Glucosidase inhibition

The α-Glu inhibitory activity was evaluated following the procedure reported by Tao *et al.*^59^ The assay employed *p*-nitrophenyl-α-d-glucopyranoside (*p*-NPG) as the substrate, using α-Glu derived from *Saccharomyces cerevisiae* (G5003, Sigma Aldrich). The reaction mixtures were prepared in phosphate buffer (pH 7.4) by adding 75 µL of buffer, 20 µL of α-Glu solution (0.15 U mL^−1^), and the test compound. The enzymatic reaction was initiated by introducing *p*-NPG (5 mM, prepared in phosphate buffer, pH 7.4), followed by incubation at 40 °C. After the reaction period, the absorbance was recorded at 405 nm to determine enzymatic activity. The inhibitory effects of synthesized compounds were assessed using a minimum of five distinct inhibitor concentrations. The kinds of inhibition and *K*_i_ constants were determined using Lineweaver and Burk curves.^60^

#### 4.2.2. α-Amylase inhibition

The inhibitory activity of compounds against α-Amy from human saliva (A1031, Sigma-Aldrich) was evaluated following the method described by Xiao *et al.*^61^ For the preparation of the starch solution, 6 g of starch was dissolved in 240 mL of 0.4 M NaOH and heated at 70 °C for 25 min. The mixture was then cooled in an ice bath, and the pH was adjusted to 6.9 using 2.0 M HCl. Finally, the volume was brought up to 300 mL with distilled water. Various concentrations of sample solutions were obtained by dilution with phosphate buffer (PB, pH 6.9) to ensure complete enzyme inhibition. In the assay, 50 µL of the substrate, 100 µL of PB, and 5–200 µL of the sample solution were mixed and pre-incubated at 37 °C for 30 min. Subsequently, 10 µL of α-Amy solution (50 µg mL^−1^) was added, and the reaction mixture was further incubated for 30 min. The absorbance was measured spectrophotometrically at 580 nm. One unit of α-Amy activity was defined as the amount of enzyme required to release 1.0 mg of maltose from starch in 3 min at pH 6.9 and 20 °C.

### 4.3. Computational studies

Molecular docking and molecular dynamics simulations were carried out using the Schrödinger Molecular Modeling Suite (release 2024-1), employing the Maestro interface (v13.9) and Desmond (D. E. Shaw Research). Protein and ligand preparations were performed following standard protocols previously described in the literature. Since the three-dimensional crystal structure of *Saccharomyces cerevisiae* α-Glu is not available, a homology model of the enzyme (Uniprot ID: P53341) was constructed based on the closely related *Saccharomyces cerevisiae* isomaltase (PDB ID: 3 A4A). The modeled structure was subsequently refined and optimized using Schrödinger's Protein Preparation Wizard to ensure geometric accuracy and suitability for docking studies.^62^ The crystal structure of α-amylase (PDB ID: 4W93) was used for molecular docking study of α-Amy.^52^ Molecular docking was performed using Glide in Extra Precision (XP) mode, generating 20 docking poses for each ligand. To account for receptor flexibility, the Induced Fit Docking (IFD) protocol was also applied, allowing both the ligand and active site residues to adjust during docking.^63^ The most favorable poses were selected based on their IFD docking scores, which reflect both binding affinity and conformational stability within the active site. Furthermore, prime MM-GBSA calculations were employed to estimate the binding free energies of the optimized complexes, using the VSGB solvation model to obtain accurate thermodynamic insights into protein–ligand interactions.

MD simulations were performed using Desmond to evaluate the dynamic stability of the protein–ligand complex under near-physiological conditions. The system was solvated in an explicit TIP3P water model and neutralized by adding appropriate counterions to maintain electrostatic balance. The simulation was run for 250 ns under NPT ensemble conditions at 300 K and 1 atm to ensure thermodynamic equilibrium. Throughout the simulation, the root mean square deviation (RMSD) of the protein backbone and ligand atoms was monitored to assess structural stability and conformational integrity. Furthermore, key non-covalent interactions-including hydrogen bonds, hydrophobic contacts, and salt bridges-were continuously analyzed to elucidate the persistence and strength of binding throughout the trajectory.^64,65^

The pharmacokinetic behavior of the synthesized compounds was evaluated using the QikProp module (Schrödinger, 2024), which predicts key ADME (Absorption, Distribution, Metabolism, and Excretion) properties. This computational approach enables the estimation of drug-likeness and oral bioavailability based on experimentally derived predictive models, thereby providing reliable insight into the compounds' pharmacokinetic suitability and structural optimization potential.^66^

## Author contributions

Faiqa Noreen, Furkan Çakır: investigation, formal analysis. Feyzi Sinan Tokali: writing – original draft, data curation. Rima D. Alharthy, Xianliang Zhao: formal analysis, validation, data curation. Nastaran Sadeghian, Parham Taslimi: biological activity, data curation, software. Magdi E. A. Zaki, Sobhi M. Gomha: data curation, resources, funding acquisition. Halil Şenol: data curation, software, writing – review & editing. Zahid Shafiq: writing – original draft, supervision, conceptualization.

## Conflicts of interest

The authors declare that they have no known competing financial interests or personal relationships that could have appeared to influence the work reported in this paper.

## Supplementary Material

RA-016-D5RA08761A-s001

## Data Availability

All data generated or analyzed during this study are included in this published article [and its supplementary information (SI) files]. Supplementary information: ^1^H, ^13^C, and ^19^F NMR, HRMS, FTIR spectra and Lineweaver–Burk graphs of the compounds. See DOI: https://doi.org/10.1039/d5ra08761a.
