# Supplementary material for: Rational design of phenyl 2,4,5-trichlorobenzenesulfonate based thiosemicarbazones as α-glucosidase and α-amylase inhibitors: integrating enzymatic evaluation and molecular modeling
Source: RSC Adv. 2026 Jan 6;16(2):1662–81. doi: 10.1039/d5ra08761a (PMC12771563; doi:10.1039/d5ra08761a)
Supplement: RA-016-D5RA08761A-s001 [file RA-016-D5RA08761A-s001.pdf]

Supplementary Information (SI) for RSC Advances.
This journal is © The Royal Society of Chemistry 2025

SUPPLEMENTARY MATERIAL

CONTENTS **Page**

^{1}H NMR Spectrum of Compound 1 3
^{13}C NMR Spectrum of Compound 1 3
HRMS Spectrum of Compound 1 4
FTIR Spectrum of Compound 1 4
^{1}H NMR Spectrum of Compound 2 5
^{13}C NMR Spectrum of Compound 2 5
HRMS Spectrum of Compound 2 6
FTIR Spectrum of Compound 2 6
^{1}H NMR Spectrum of Compound 3 7
^{13}C NMR Spectrum of Compound 3 7
HRMS Spectrum of Compound 3 8
FTIR Spectrum of Compound 3 8
^{1}H NMR Spectrum of Compound 4 9
^{13}C NMR Spectrum of Compound 4 9
HRMS Spectrum of Compound 4 10
FTIR Spectrum of Compound 4 10
^{1}H NMR Spectrum of Compound 5 11
^{13}C NMR Spectrum of Compound 5 11
HRMS Spectrum of Compound 5 12
FTIR Spectrum of Compound 5 12
^{1}H NMR Spectrum of Compound 6 13
^{13}C NMR Spectrum of Compound 6 13
HRMS Spectrum of Compound 6 14
FTIR Spectrum of Compound 6 14
^{1}H NMR Spectrum of Compound 7 15
^{13}C NMR Spectrum of Compound 7 15
HRMS Spectrum of Compound 7 16
FTIR Spectrum of Compound 7 16
^{1}H NMR Spectrum of Compound 8 17
^{13}C NMR Spectrum of Compound 8 17
HRMS Spectrum of Compound 8 18
FTIR Spectrum of Compound 8 18
^{1}H NMR Spectrum of Compound 9 19
^{13}C NMR Spectrum of Compound 9 19
^{19}F NMR Spectrum of Compound 9 20
HRMS Spectrum of Compound 9 20
FTIR Spectrum of Compound 9 21
^{1}H NMR Spectrum of Compound 10 21
^{13}C NMR Spectrum of Compound 10 22
HRMS Spectrum of Compound 10 22
FTIR Spectrum of Compound 10 23
^{1}H NMR Spectrum of Compound 11 23
^{13}C NMR Spectrum of Compound 11 24
HRMS Spectrum of Compound 11 24
FTIR Spectrum of Compound 11 25
^{1}H NMR Spectrum of Compound 12 25
^{13}C NMR Spectrum of Compound 12 26
HRMS Spectrum of Compound 12 26
FTIR Spectrum of Compound 12 27
^{1}H NMR Spectrum of Compound 13 27
^{13}C NMR Spectrum of Compound 13 28

HRMS Spectrum of Compound 13………………………………………………………………. **28**
FTIR Spectrum of Compound 13…………………………………………………………….... **29**
^{1}H NMR Spectrum of Compound 14……………………………………………………….... **29**
^{13}C NMR Spectrum of Compound 14……………………………………………………….. **30**
^{19}F NMR Spectrum of Compound 14……………………………………………………….... **30**
HRMS Spectrum of Compound 14………………………………………………………………. **31**
FTIR Spectrum of Compound 14…………………………………………………………….... **31**
^{1}H NMR Spectrum of Compound 15……………………………………………………….... **32**
^{13}C NMR Spectrum of Compound 15……………………………………………………….. **32**
^{19}F NMR Spectrum of Compound 15……………………………………………………….... **33**
HRMS Spectrum of Compound 15………………………………………………………………. **33**
FTIR Spectrum of Compound 15…………………………………………………………….... **34**
^{1}H NMR Spectrum of Compound 16……………………………………………………….... **34**
^{13}C NMR Spectrum of Compound 16……………………………………………………….. **35**
HRMS Spectrum of Compound 16………………………………………………………………. **35**
FTIR Spectrum of Compound 16…………………………………………………………….... **36**
^{1}H NMR Spectrum of Compound 17……………………………………………………….... **36**
^{13}C NMR Spectrum of Compound 17……………………………………………………….. **37**
HRMS Spectrum of Compound 17………………………………………………………………. **37**
FTIR Spectrum of Compound 17…………………………………………………………….... **38**
^{1}H NMR Spectrum of Compound 18……………………………………………………….... **38**
^{13}C NMR Spectrum of Compound 18……………………………………………………….. **39**
HRMS Spectrum of Compound 18………………………………………………………………. **39**
FTIR Spectrum of Compound 18…………………………………………………………….... **40**
^{1}H NMR Spectrum of Compound 19……………………………………………………….... **40**
^{13}C NMR Spectrum of Compound 19……………………………………………………….. **41**
HRMS Spectrum of Compound 19………………………………………………………………. **41**
FTIR Spectrum of Compound 19…………………………………………………………….... **42**
^{1}H NMR Spectrum of Compound 20……………………………………………………….... **42**
^{13}C NMR Spectrum of Compound 20……………………………………………………….. **43**
HRMS Spectrum of Compound 20………………………………………………………………. **43**
FTIR Spectrum of Compound 20…………………………………………………………….... **44**
^{1}H NMR Spectrum of Compound 21……………………………………………………….... **44**
^{13}C NMR Spectrum of Compound 21……………………………………………………….. **45**
HRMS Spectrum of Compound 21………………………………………………………………. **45**
FTIR Spectrum of Compound 21…………………………………………………………….... **46**
^{1}H NMR Spectrum of Compound 22……………………………………………………….... **46**
^{13}C NMR Spectrum of Compound 22……………………………………………………….. **47**
HRMS Spectrum of Compound 22………………………………………………………………. **47**
FTIR Spectrum of Compound 22…………………………………………………………….... **48**
Lineweaver-Burk Graphs of Compounds 1-22 and Acarbose..………………………………… **49**
The interactions of compound 16 with α-Glu and α-Amy……………………………………... **52**

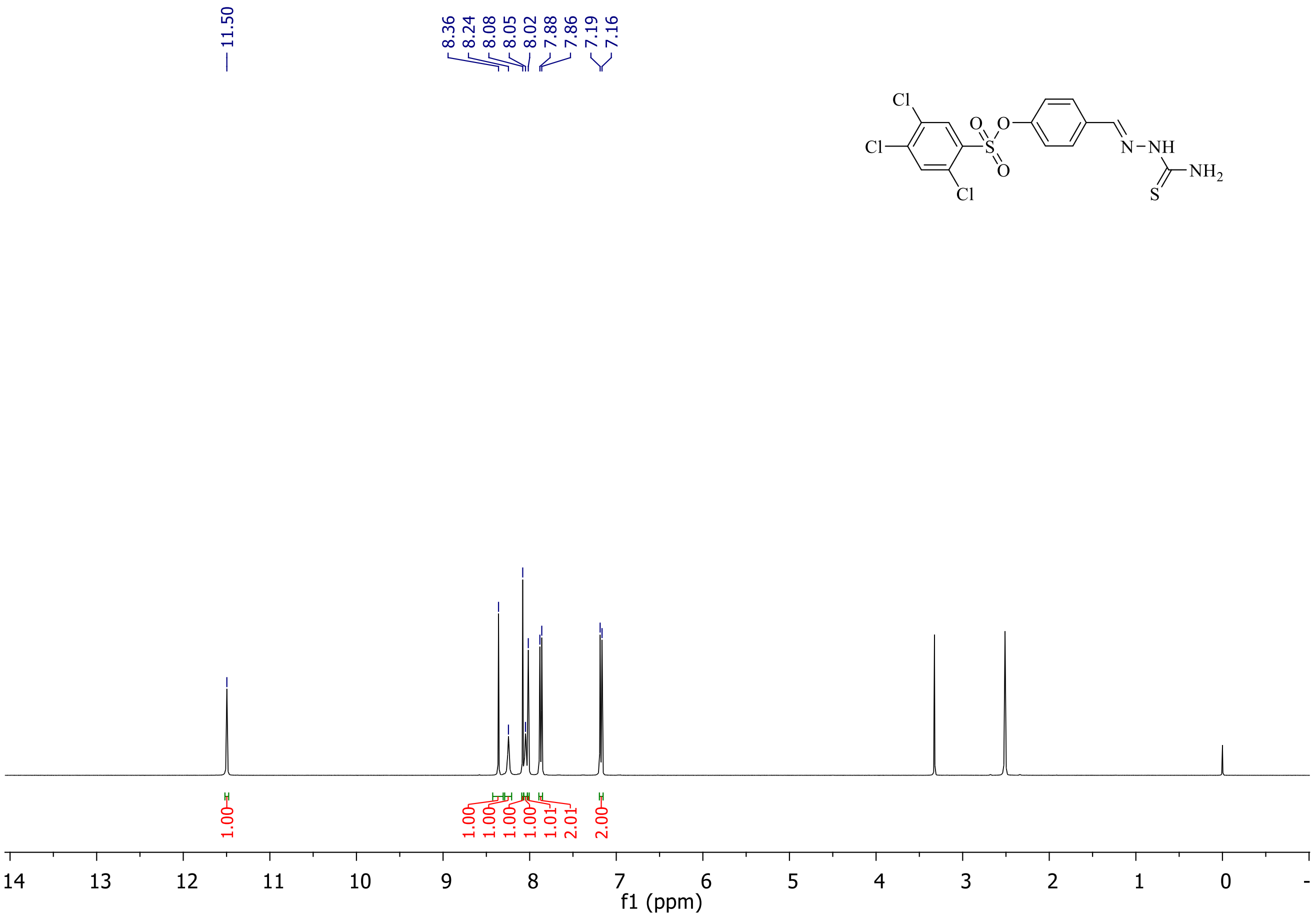

^{1}H NMR Spectrum of Compound 1

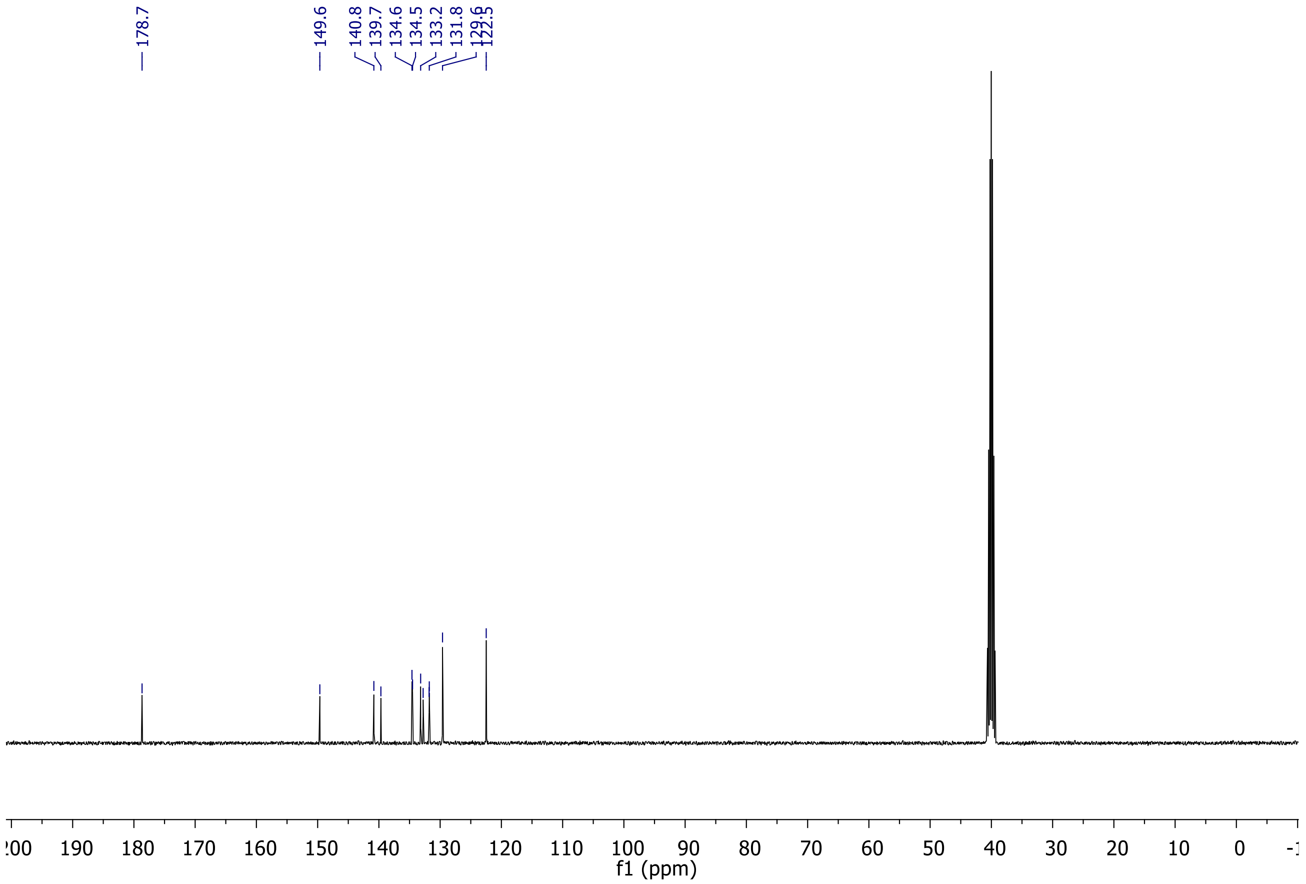

^{13}C NMR Spectrum of Compound 1

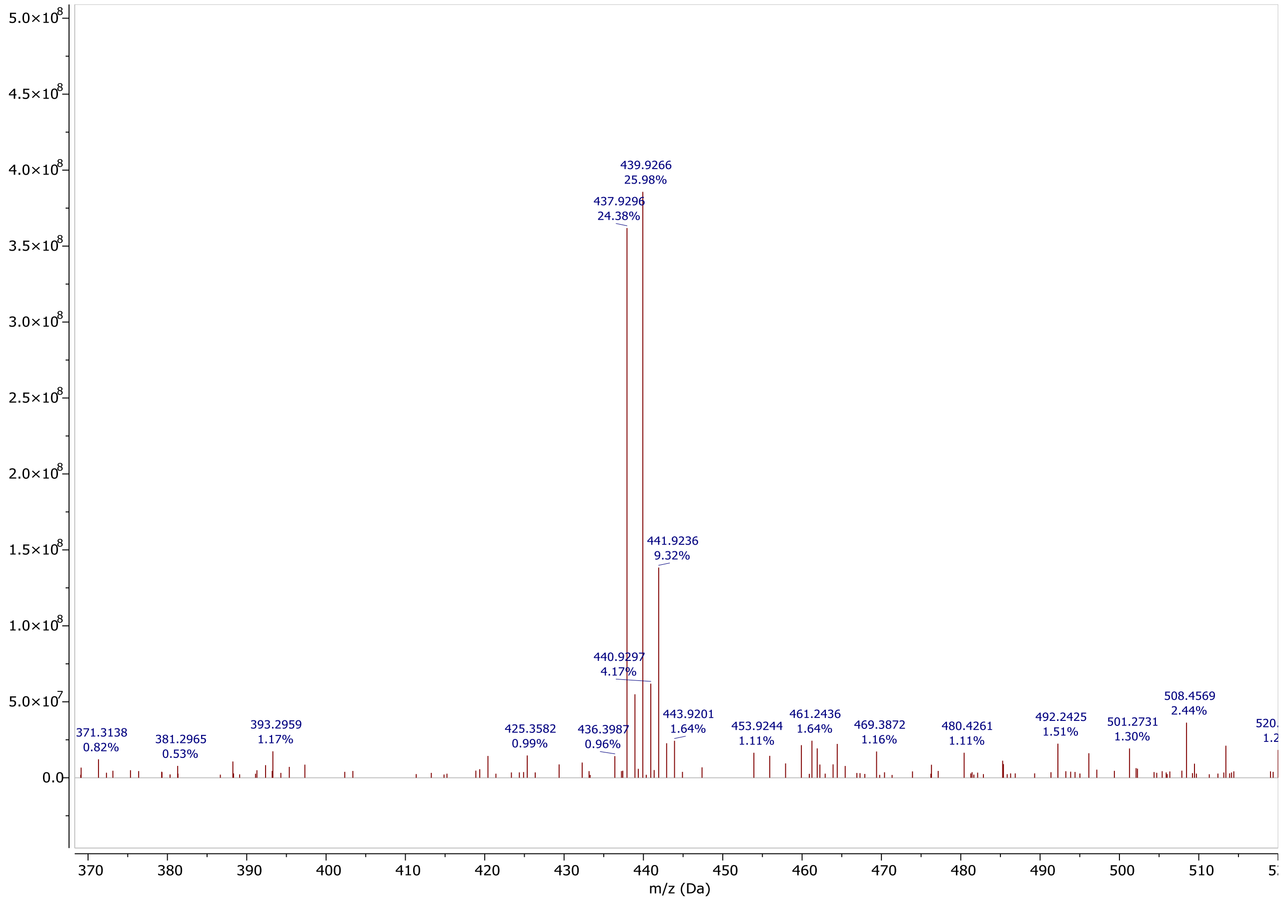

HRMS Spectrum of Compound 1

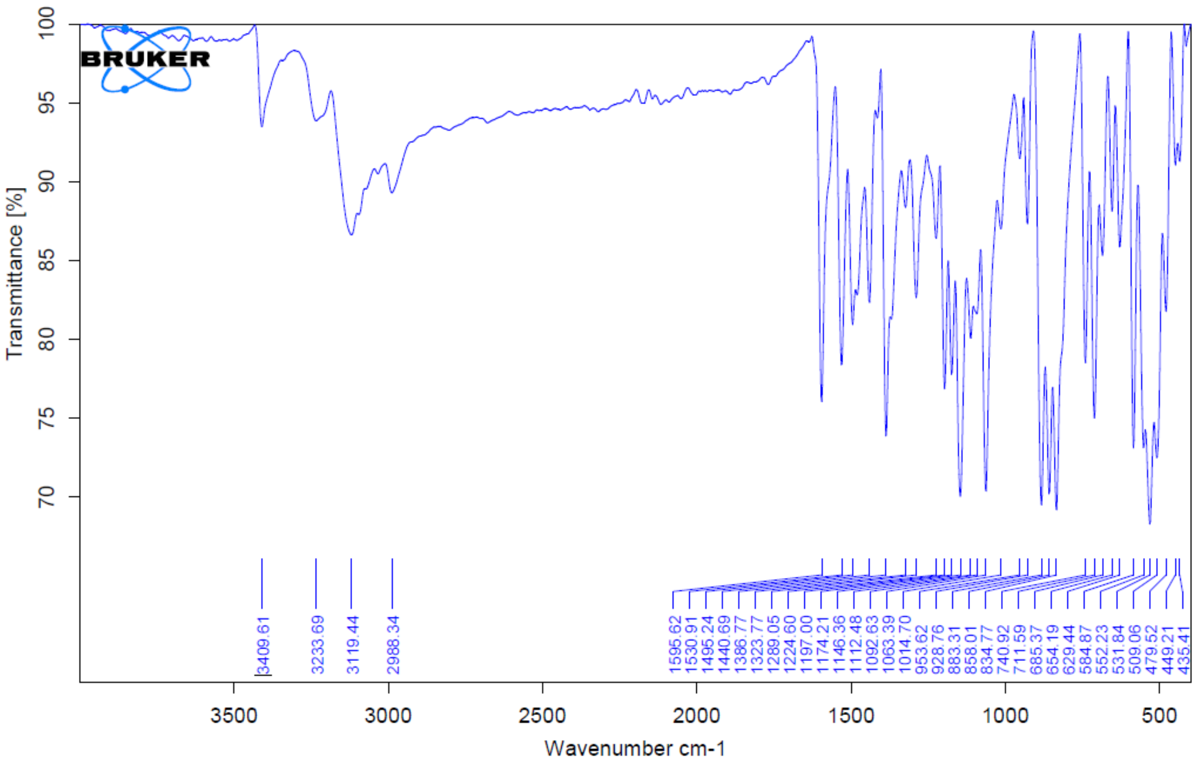

FTIR Spectrum of Compound 1

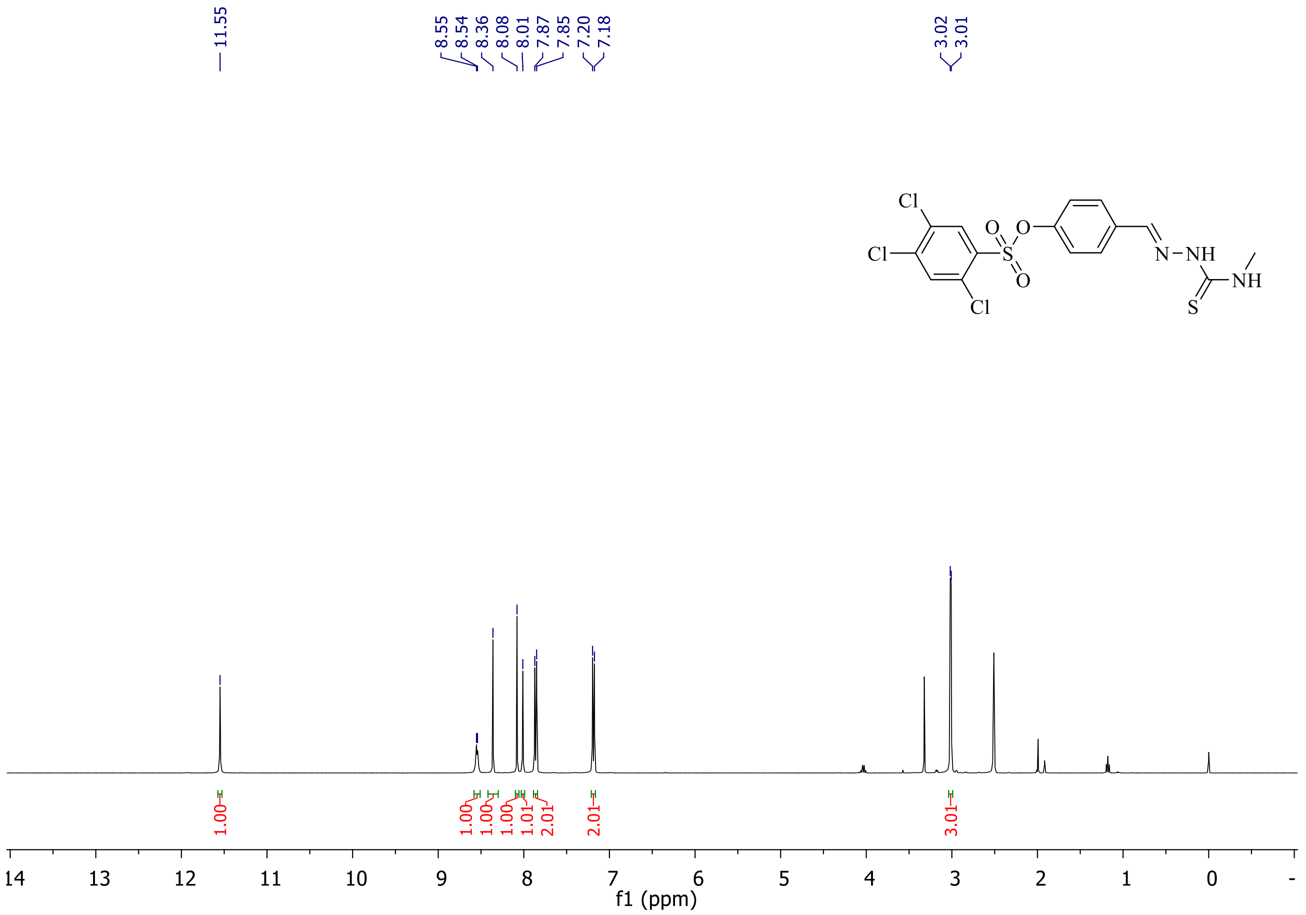

^{1}H NMR Spectrum of Compound 2

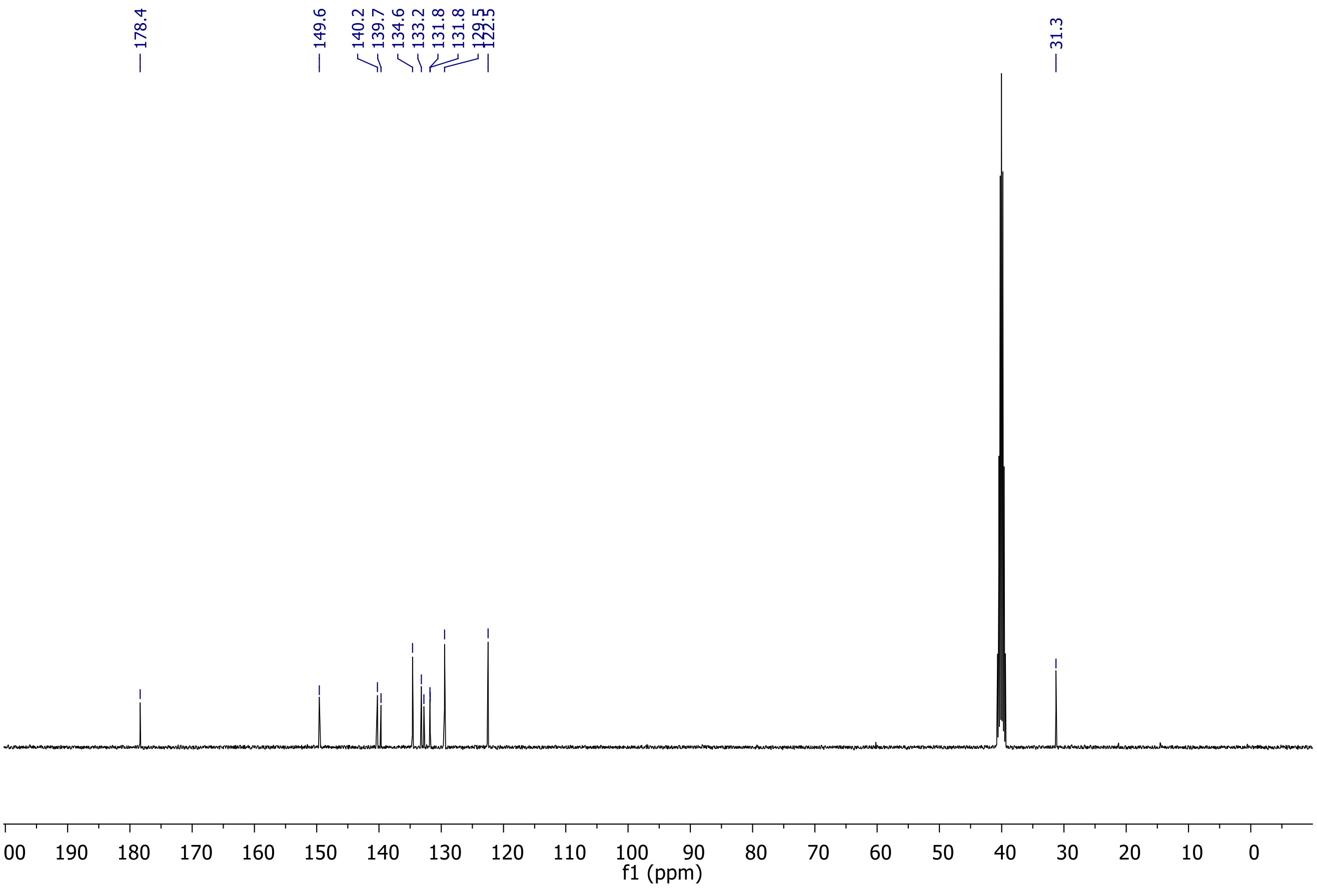

^{13}C NMR Spectrum of Compound 2

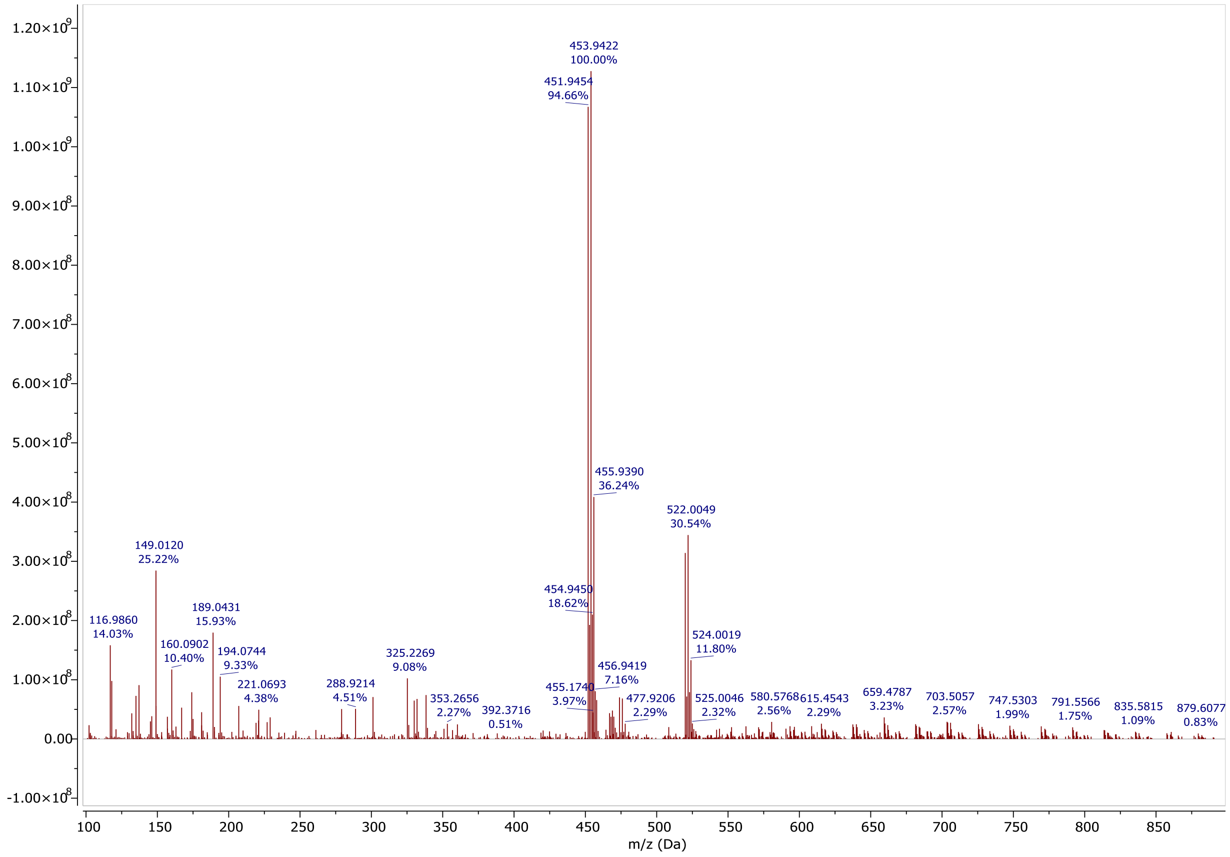

HRMS Spectrum of Compound 2

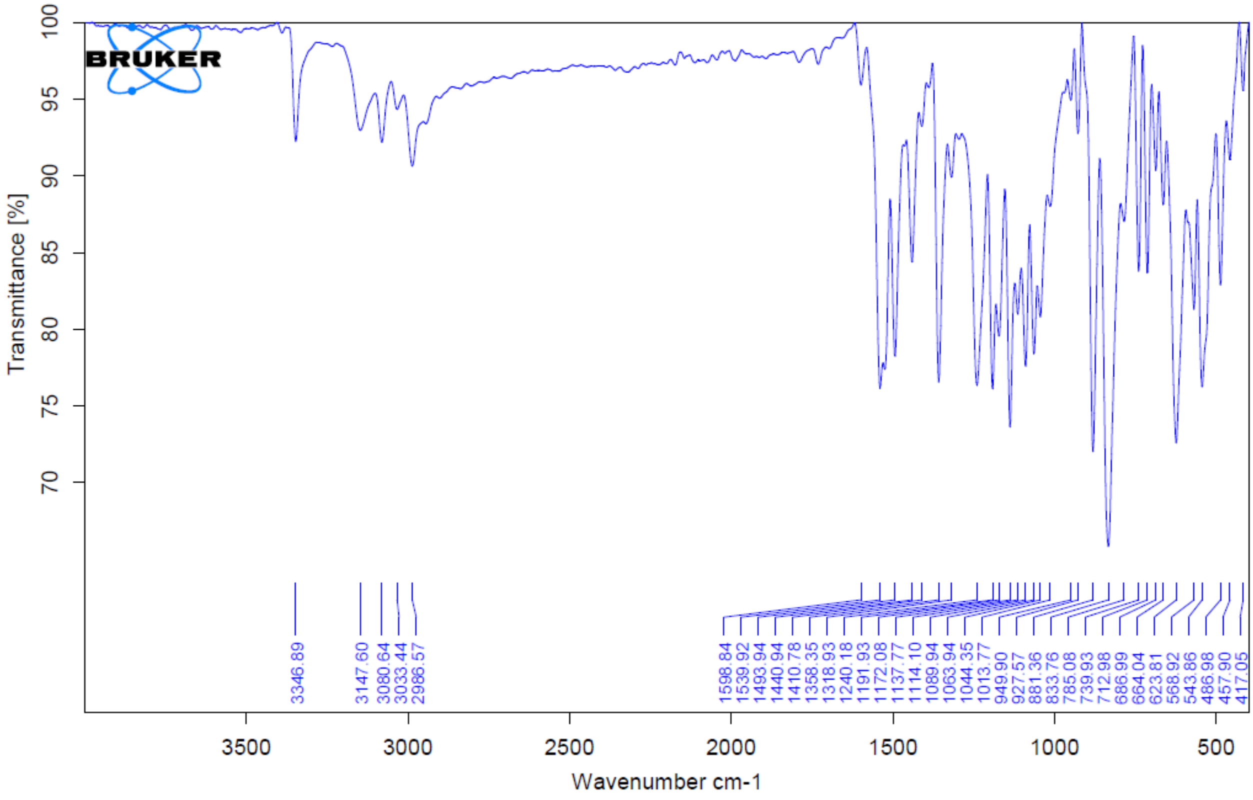

FTIR Spectrum of Compound 2

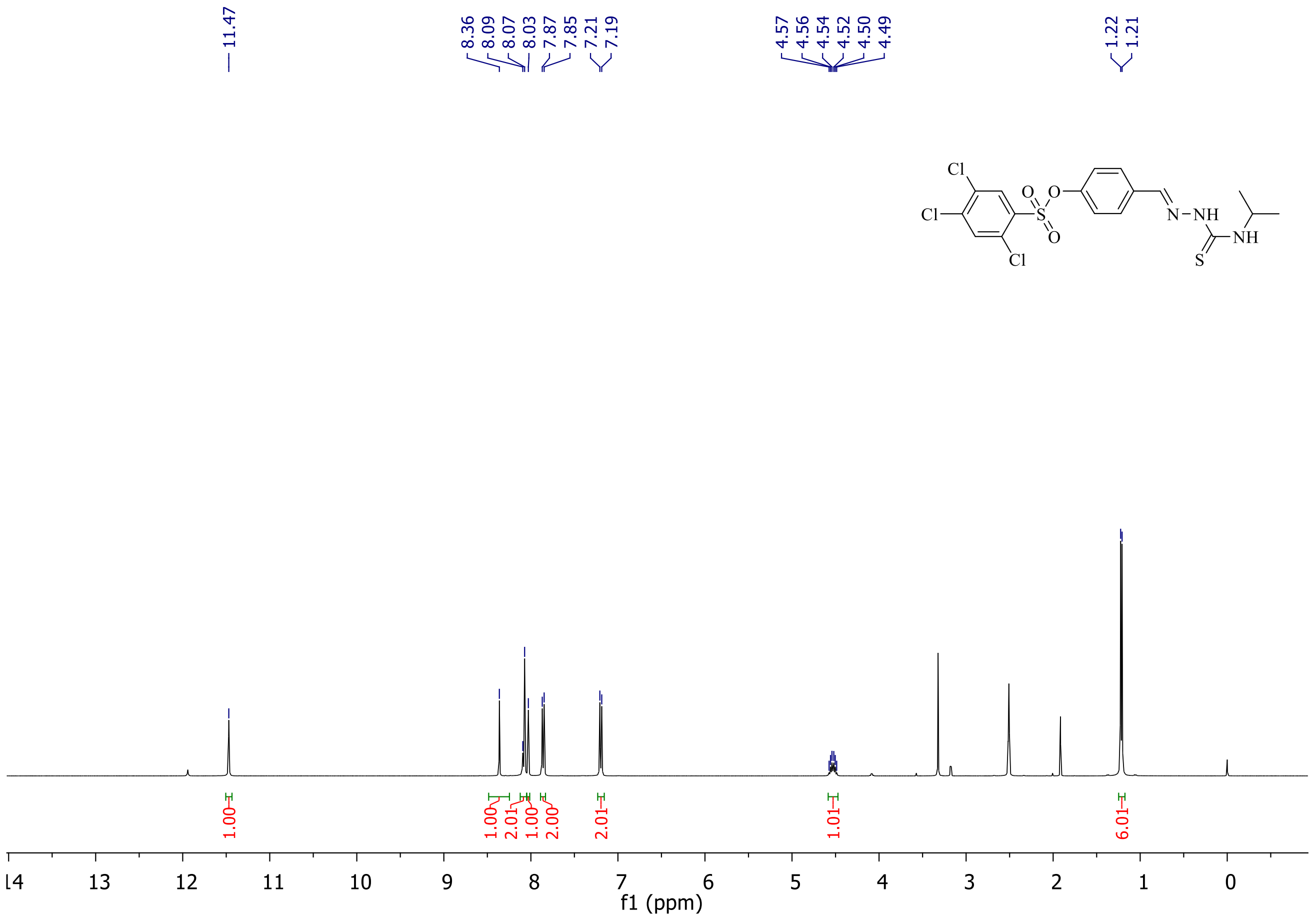

^{1}H NMR Spectrum of Compound 3

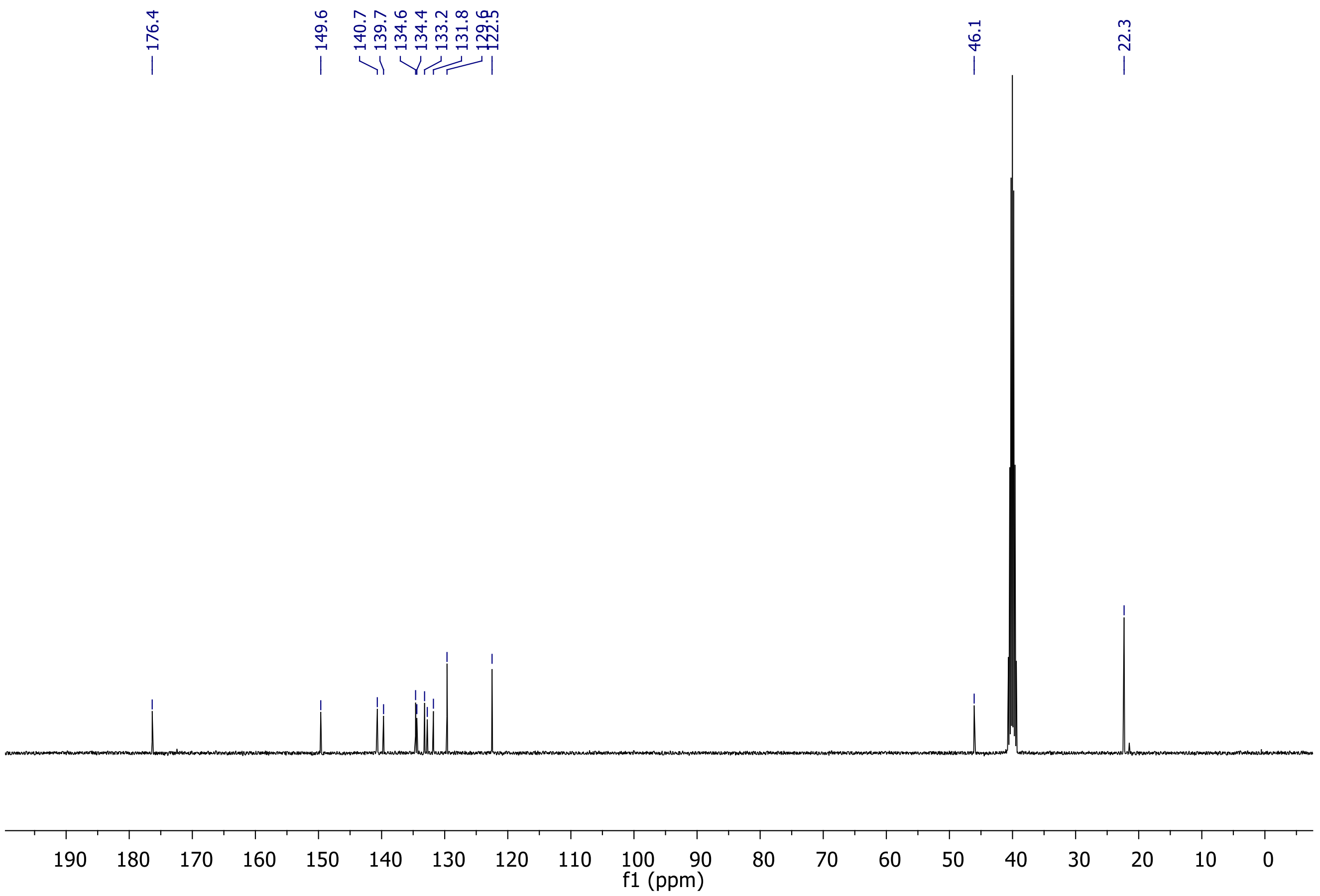

^{13}C NMR Spectrum of Compound 3

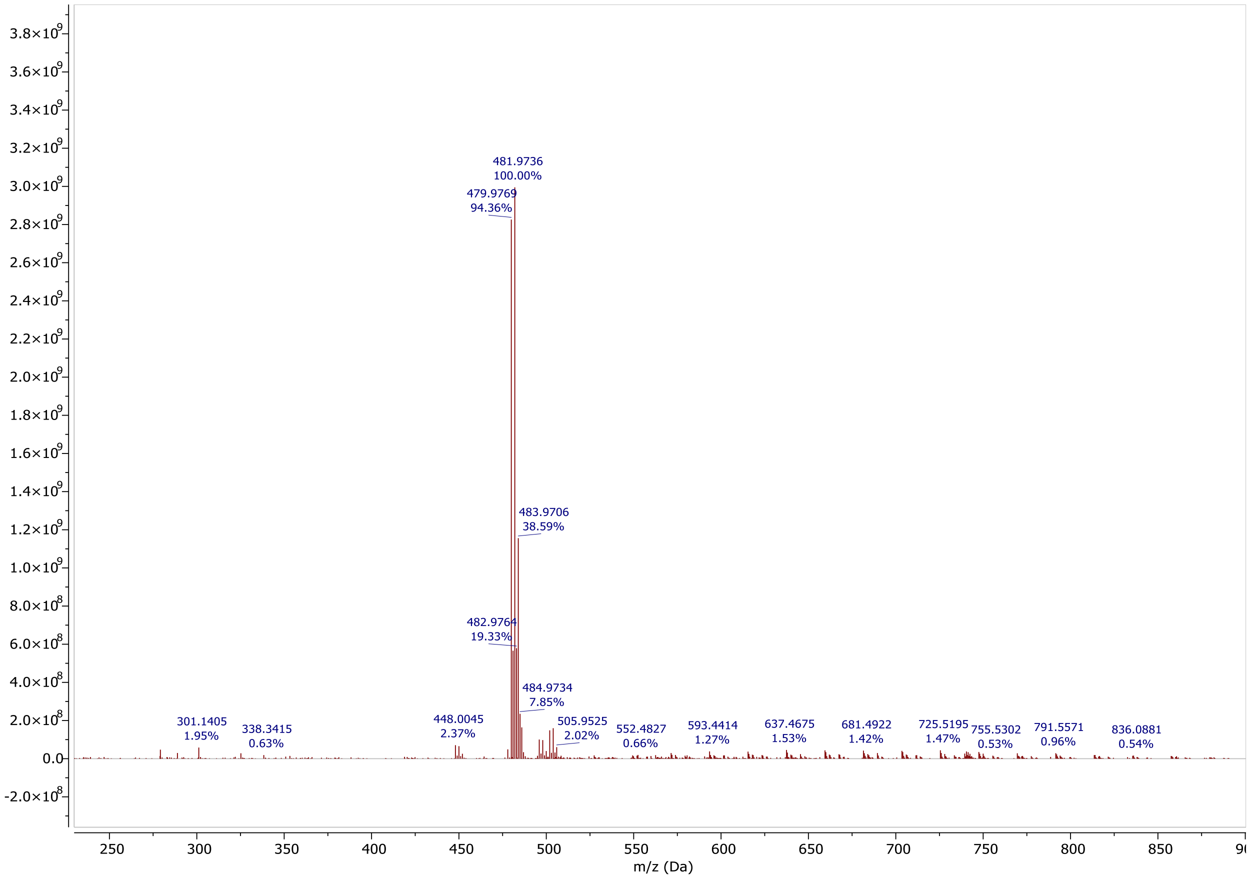

HRMS Spectrum of Compound 3

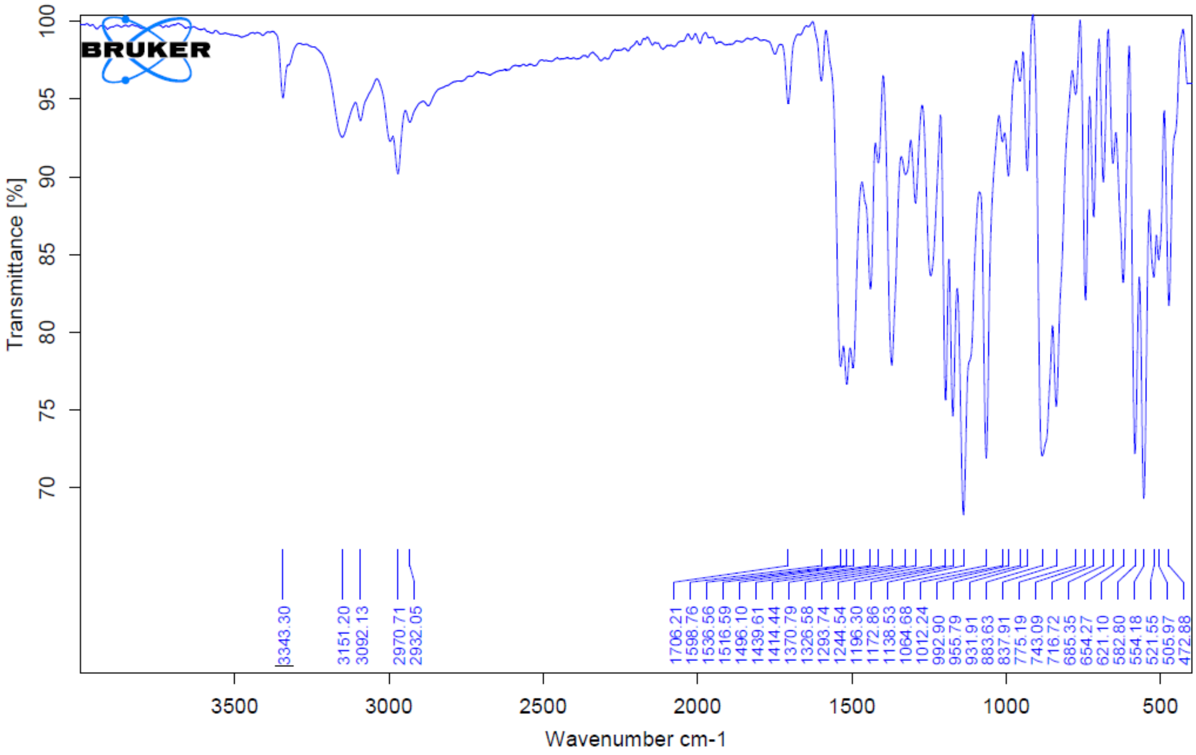

FTIR Spectrum of Compound 3

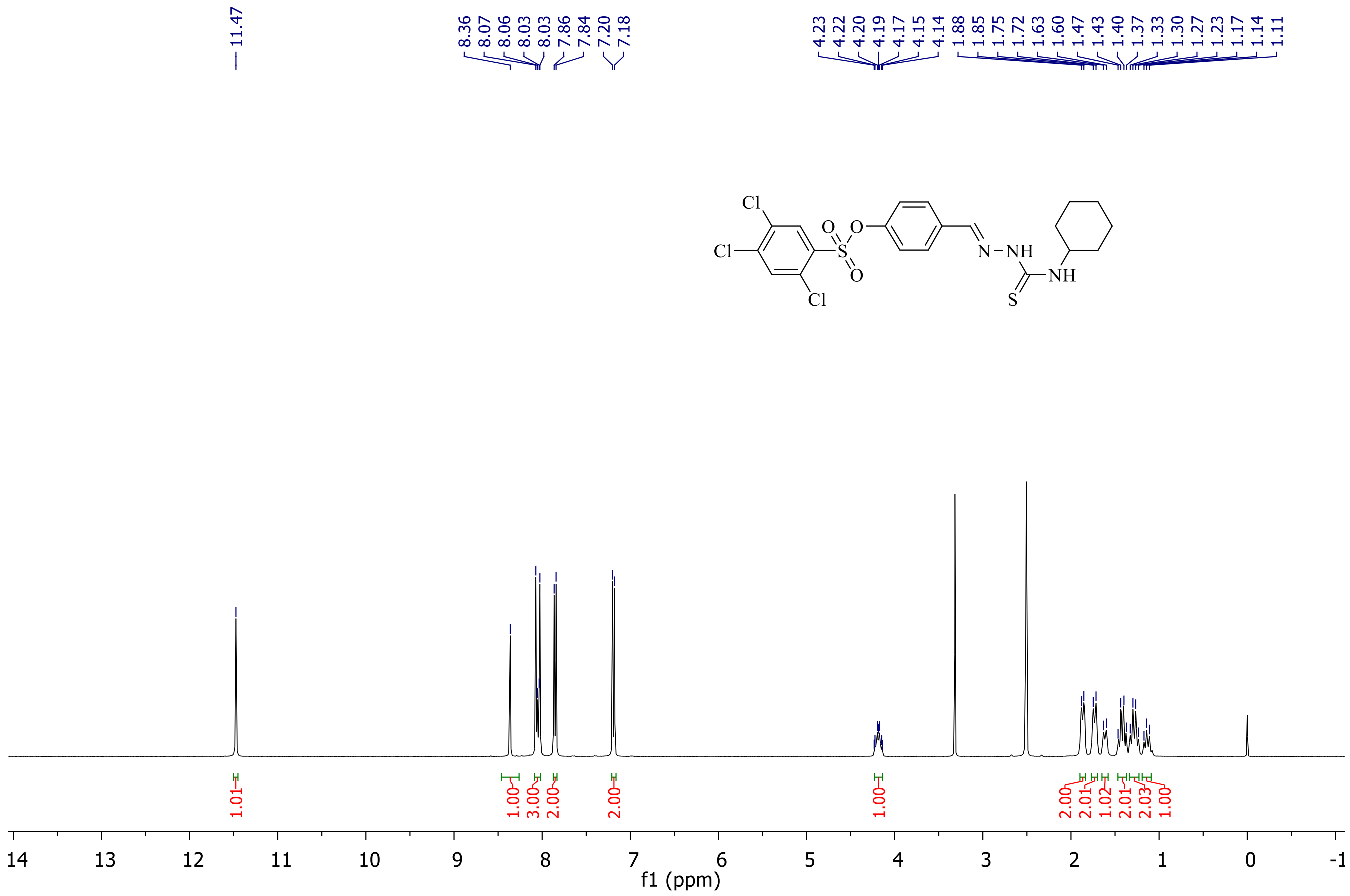

^{1}H NMR Spectrum of Compound 4

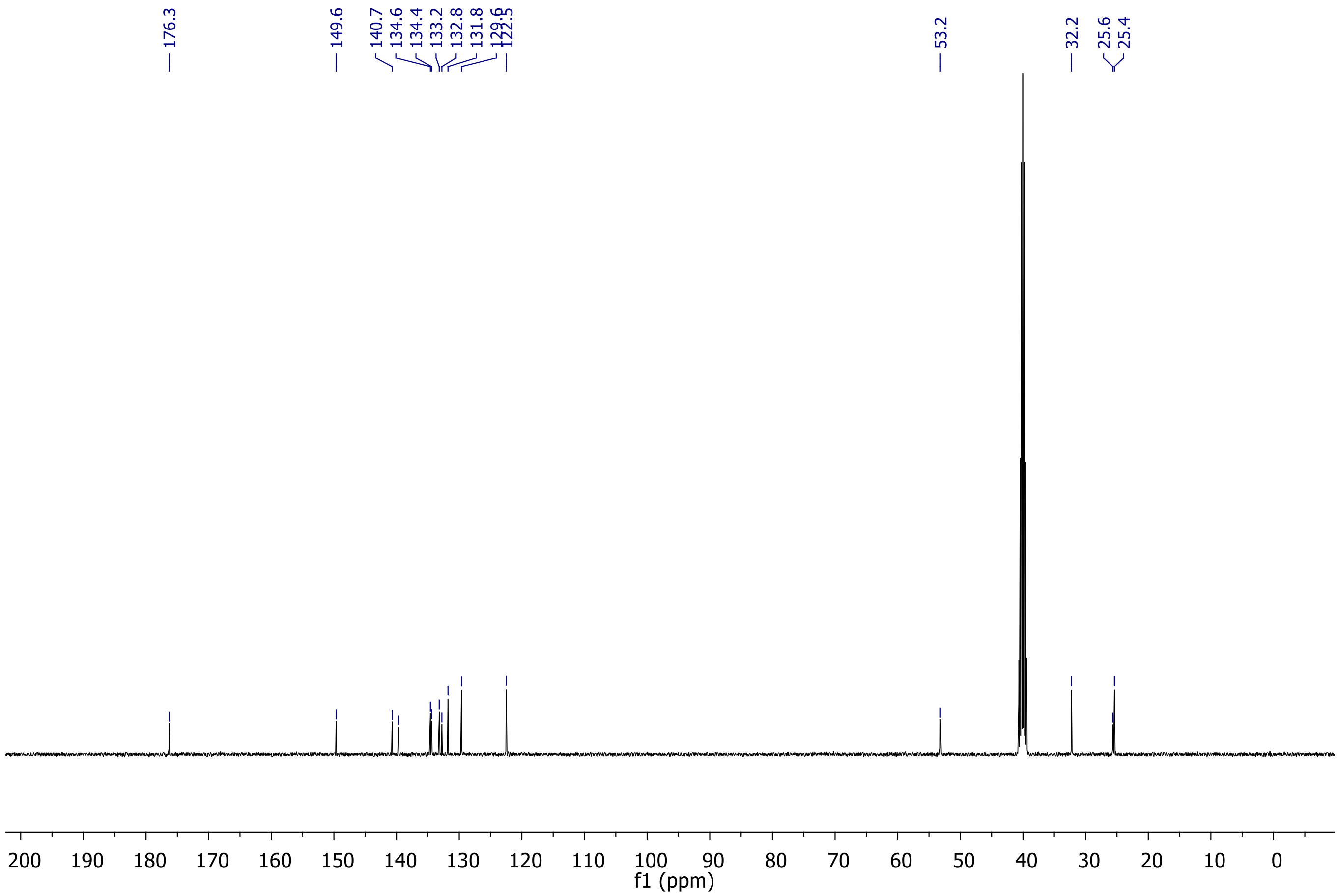

^{13}C NMR Spectrum of Compound 4

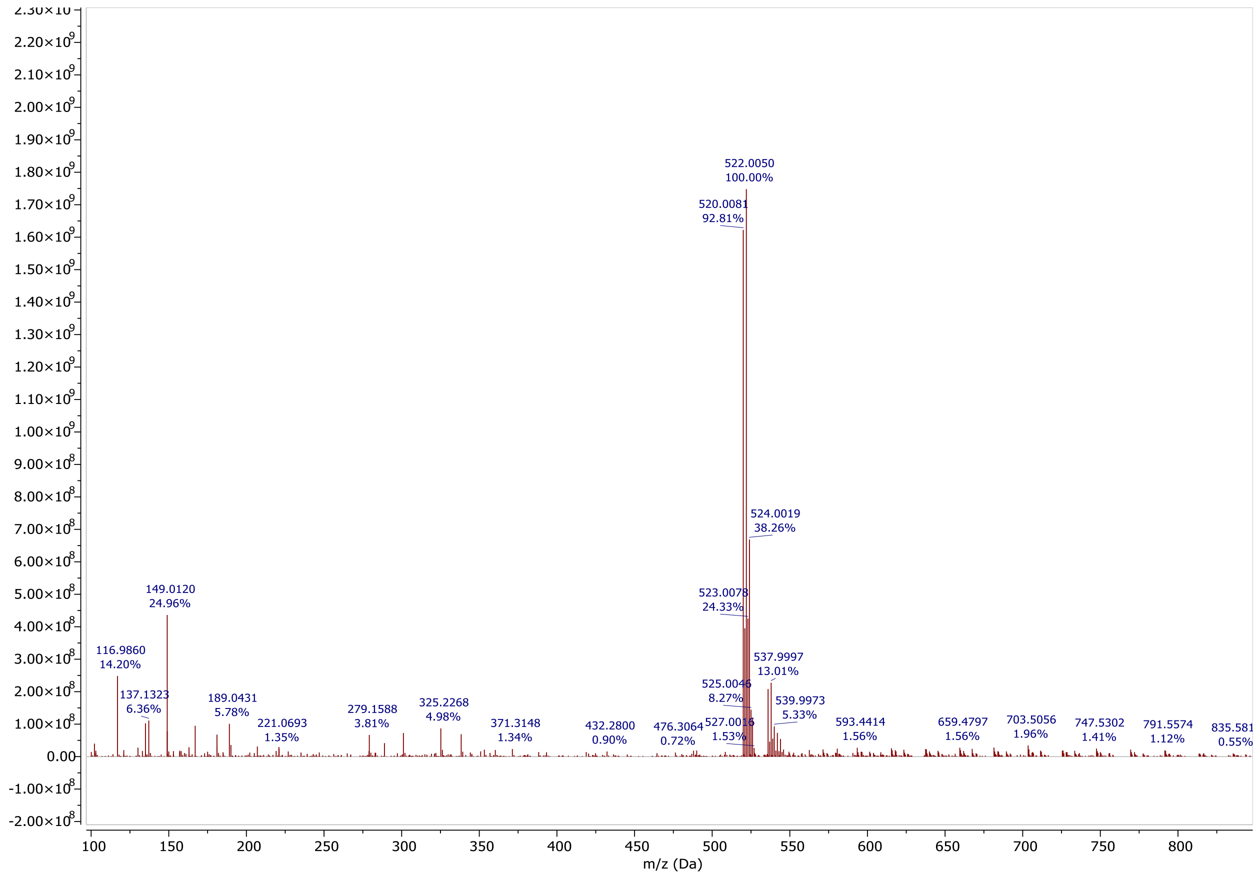

HRMS Spectrum of Compound 4

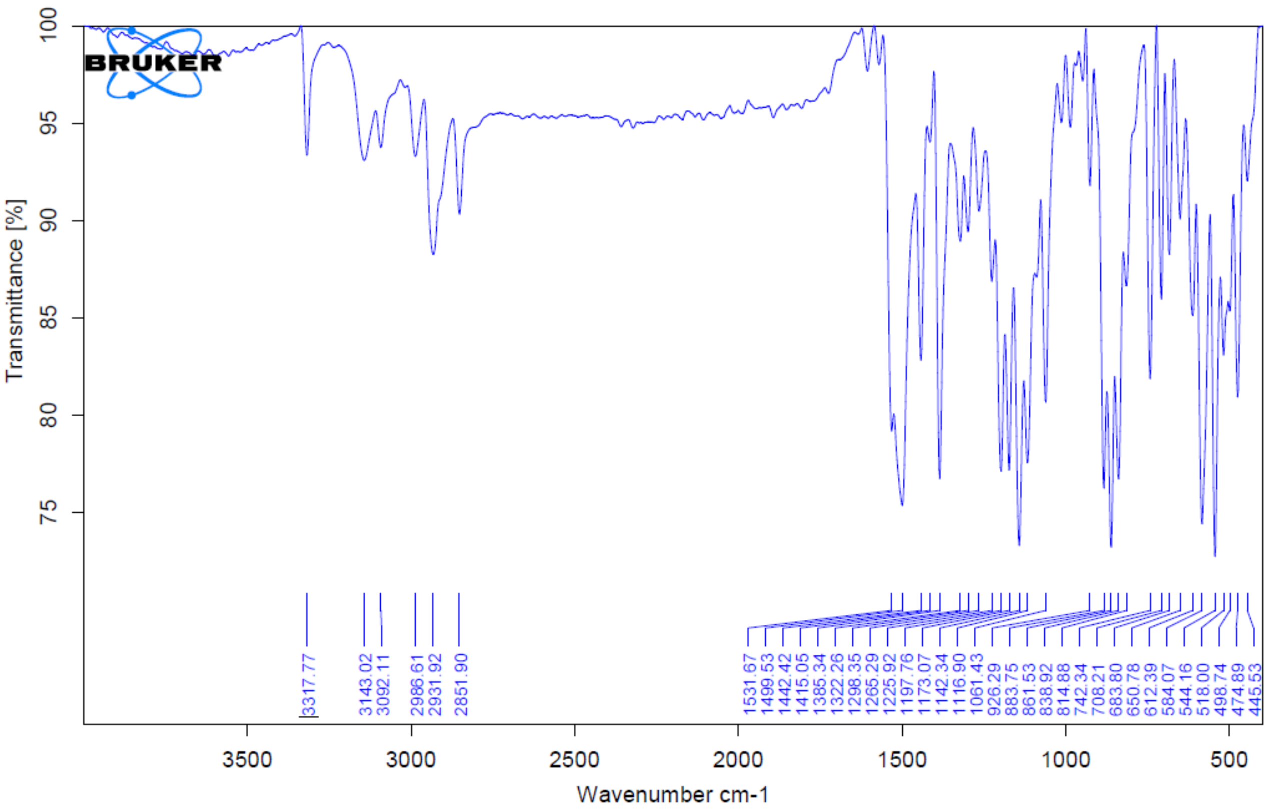

FTIR Spectrum of Compound 4

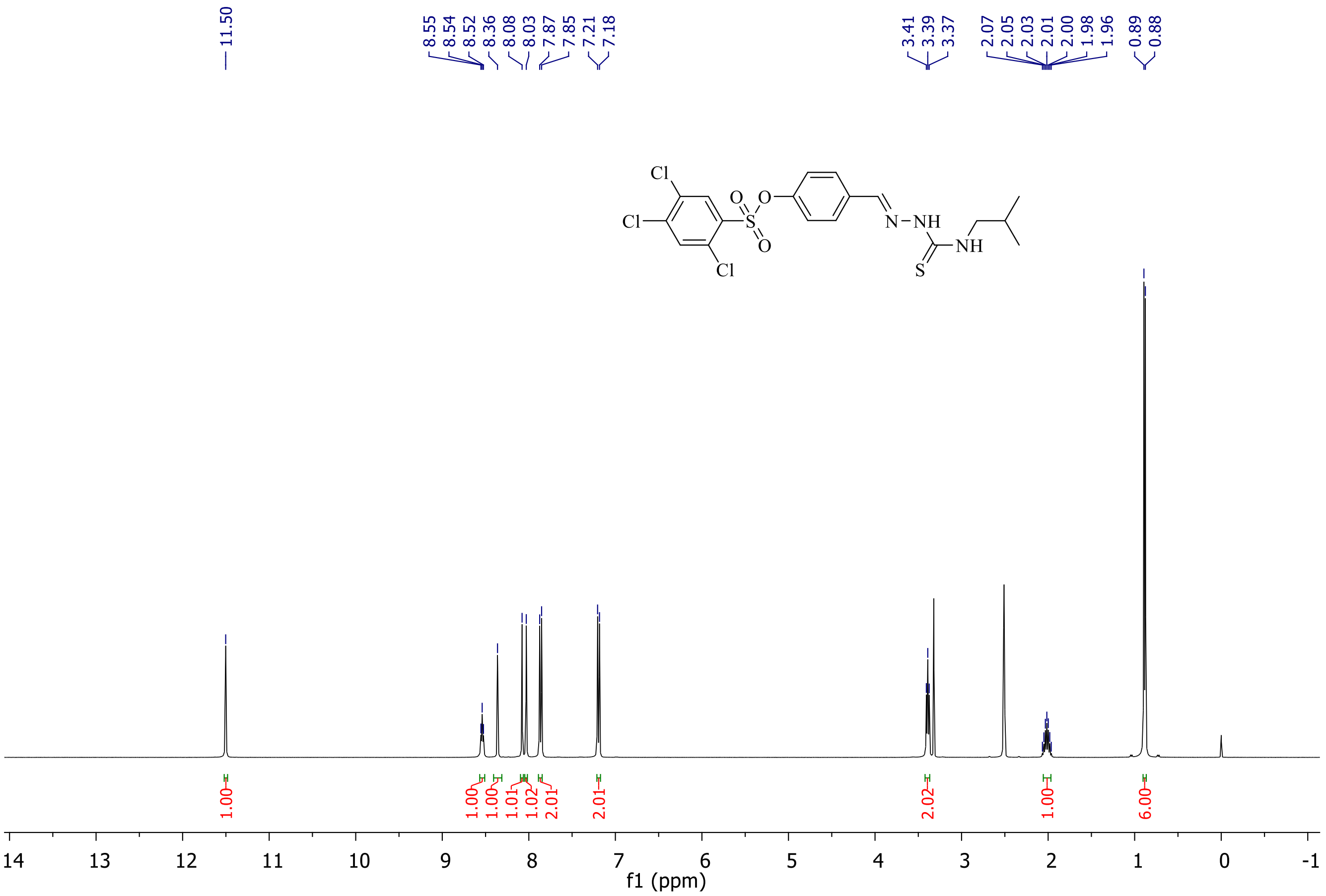

^{1}H NMR Spectrum of Compound 5

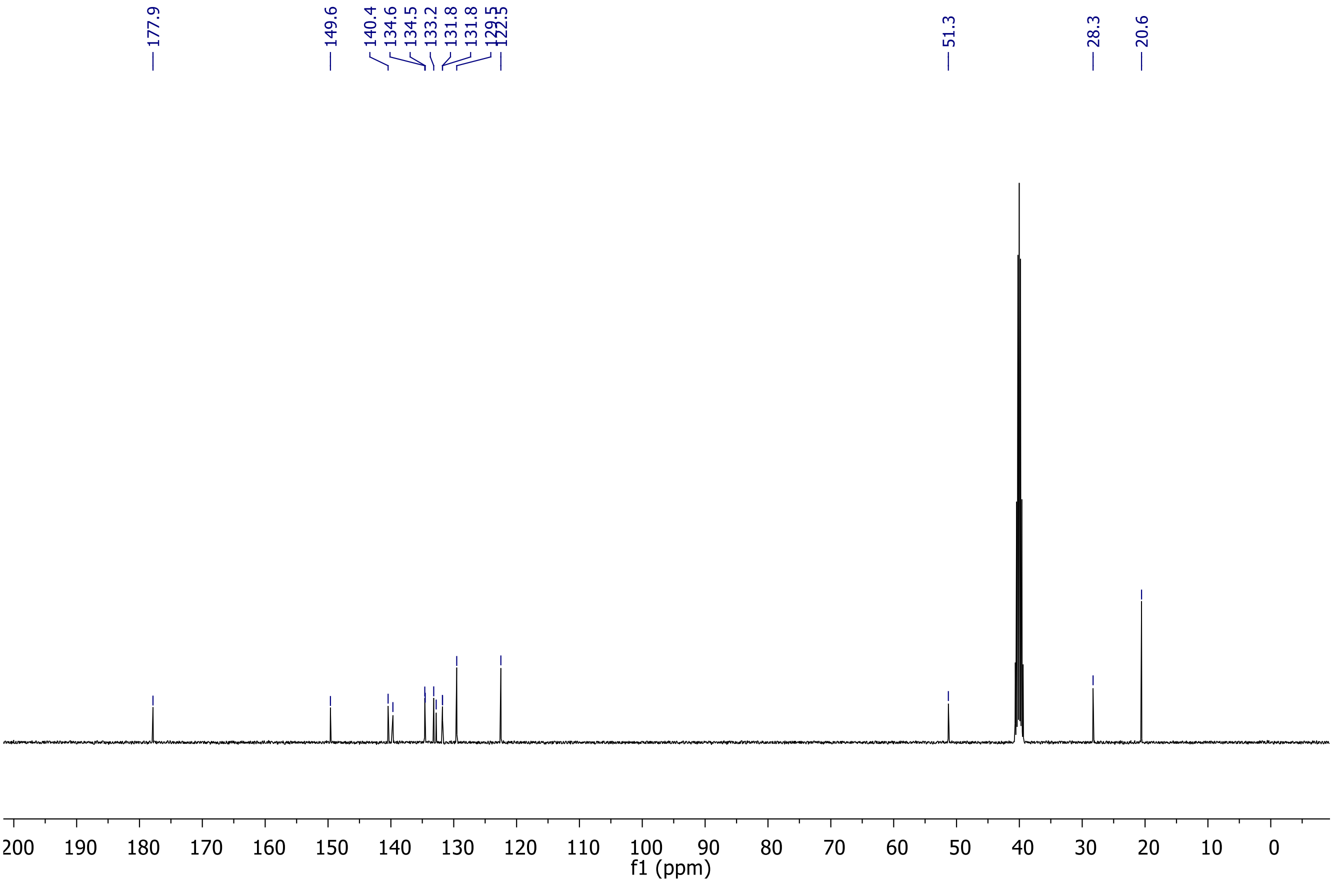

^{13}C NMR Spectrum of Compound 5

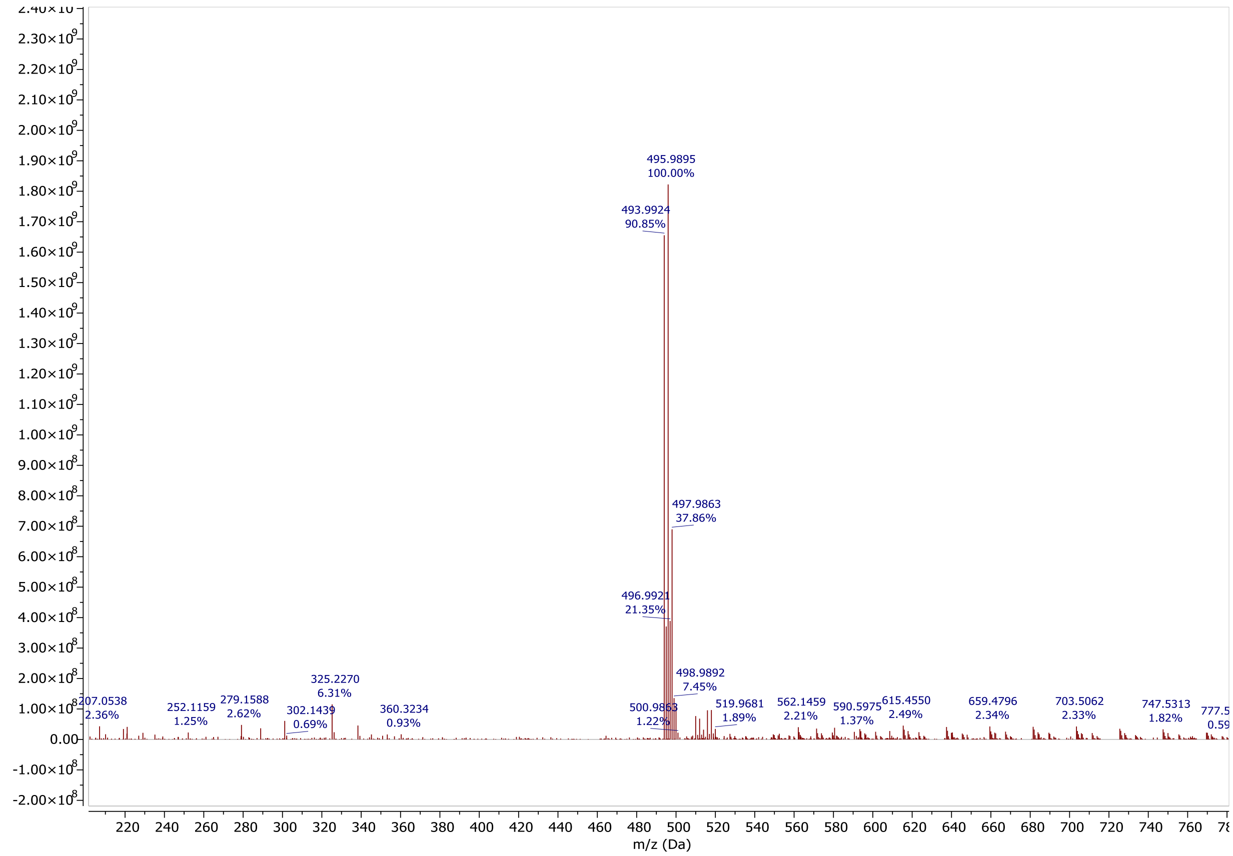

HRMS Spectrum of Compound 5

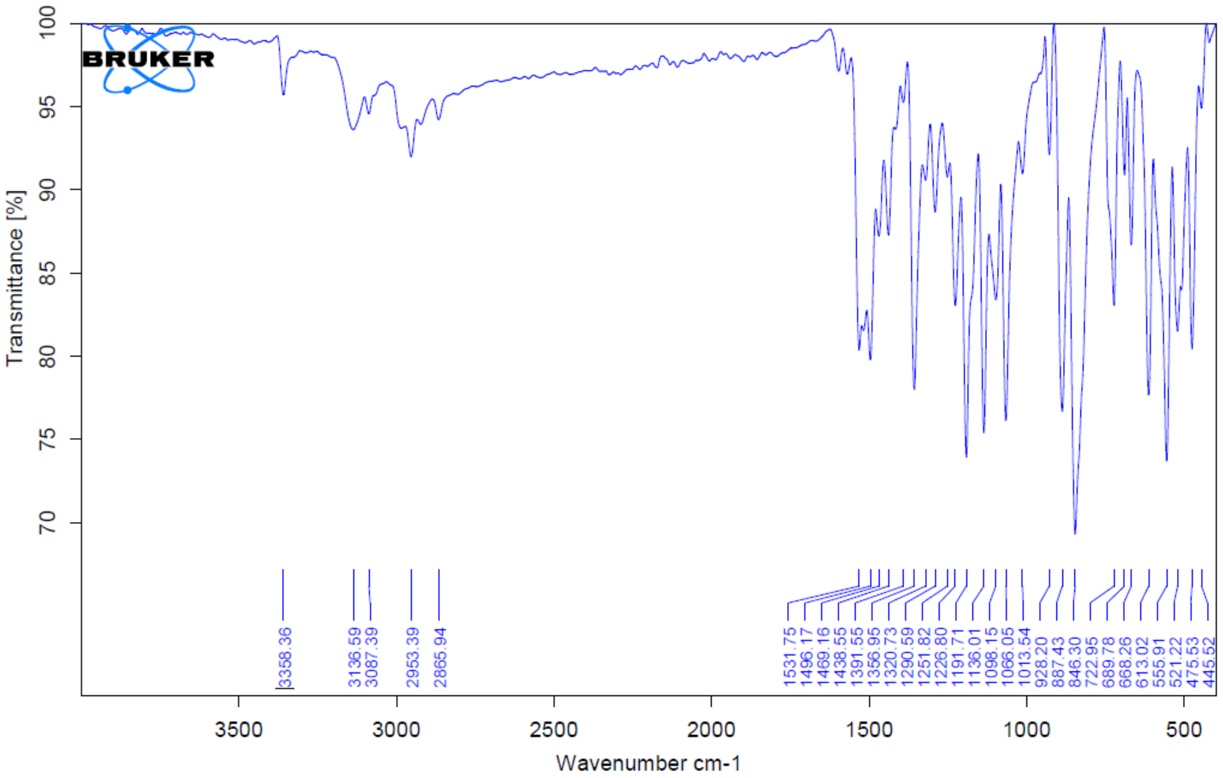

FTIR Spectrum of Compound 5

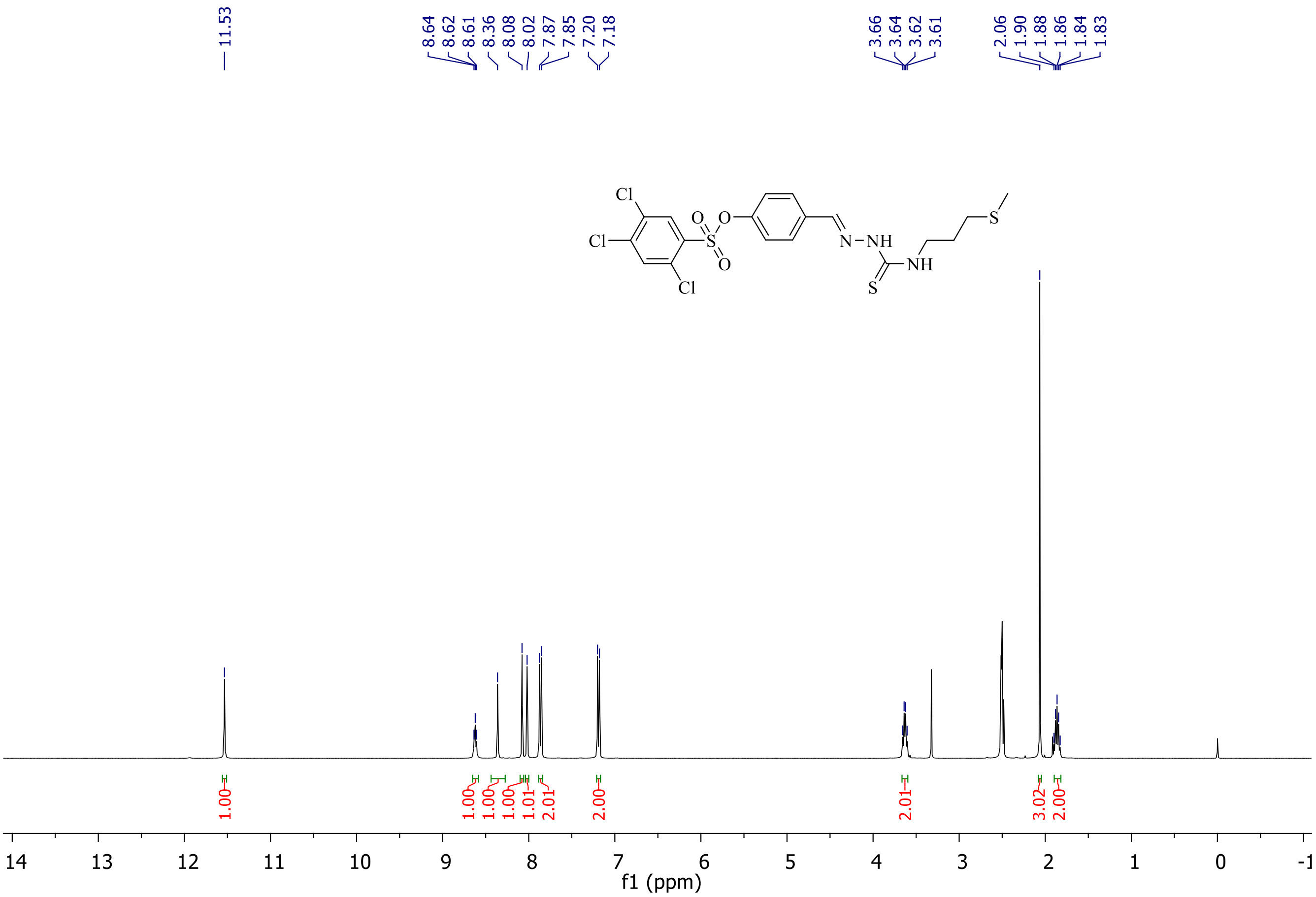

^{1}H NMR Spectrum of Compound 6

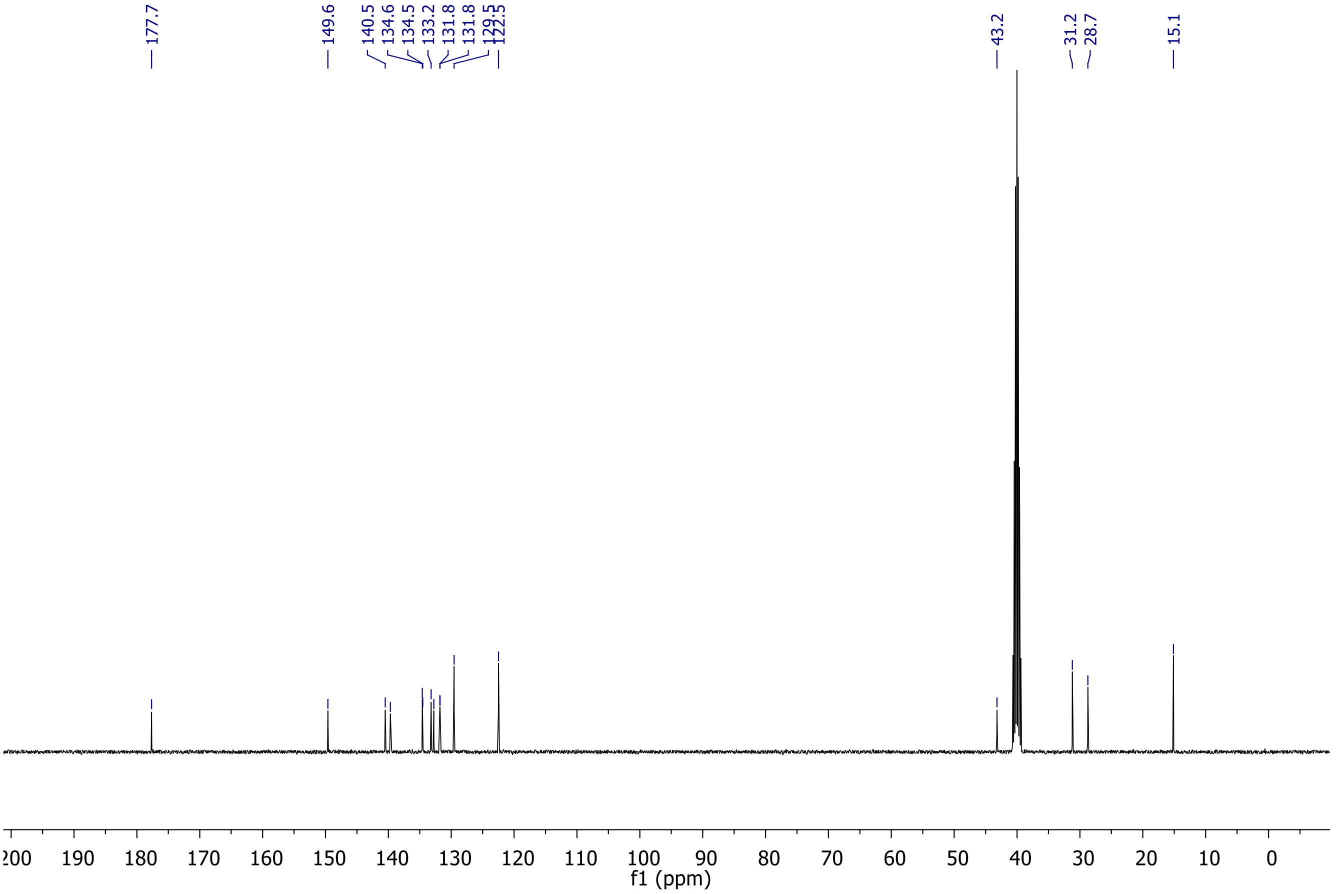

^{13}C NMR Spectrum of Compound 6

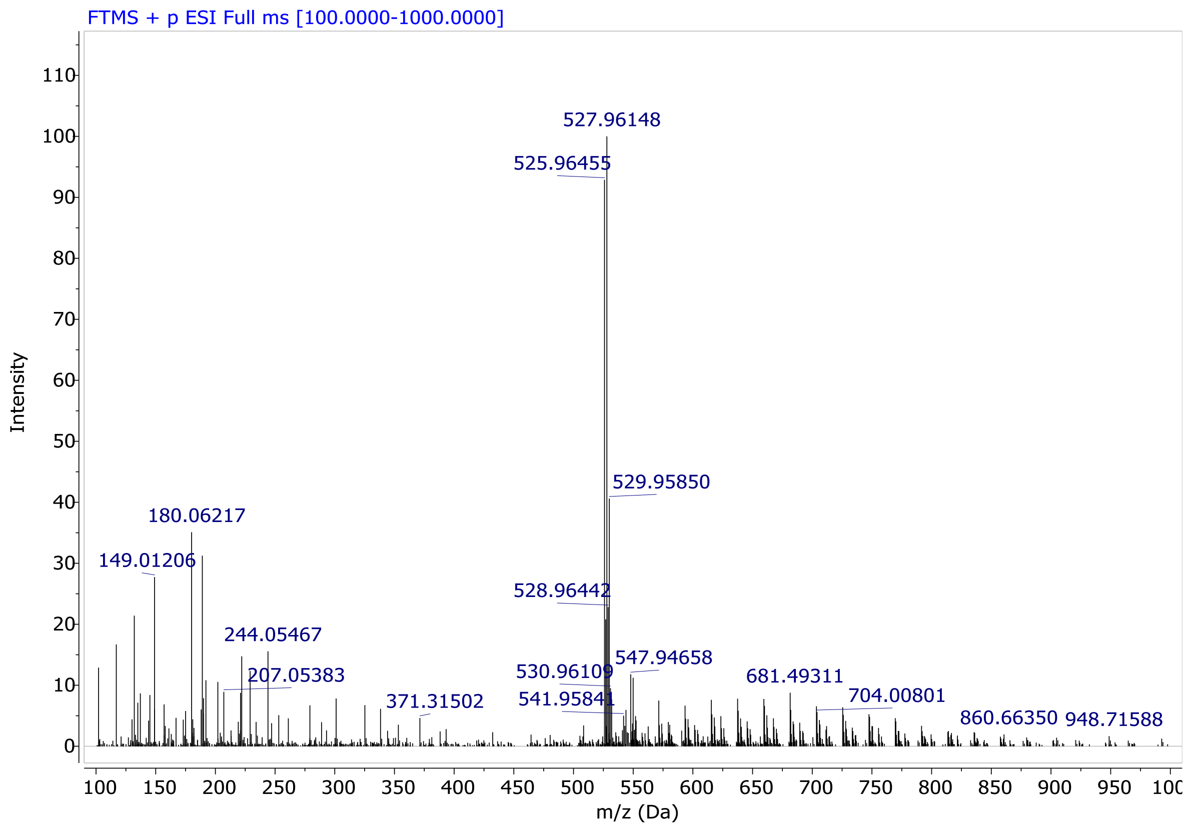

HRMS Spectrum of Compound 6

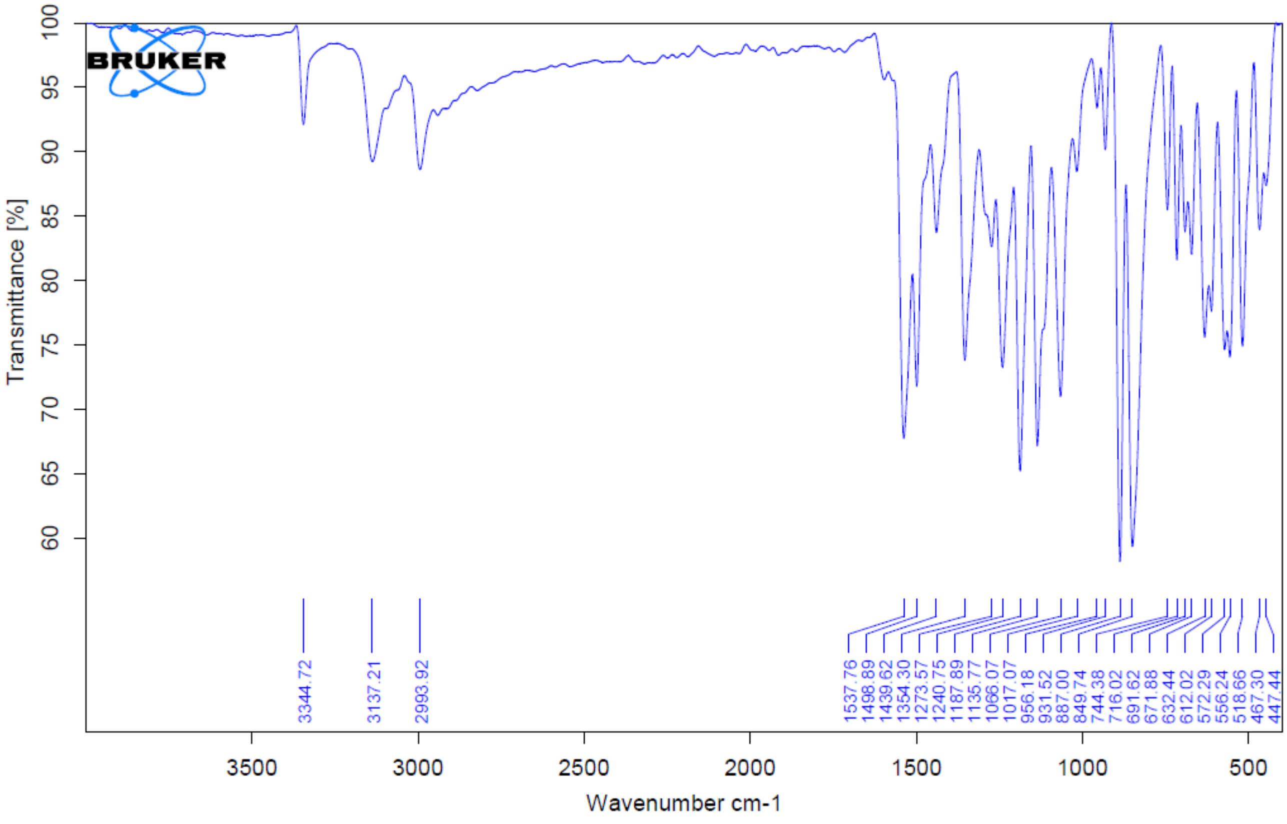

FTIR Spectrum of Compound 6

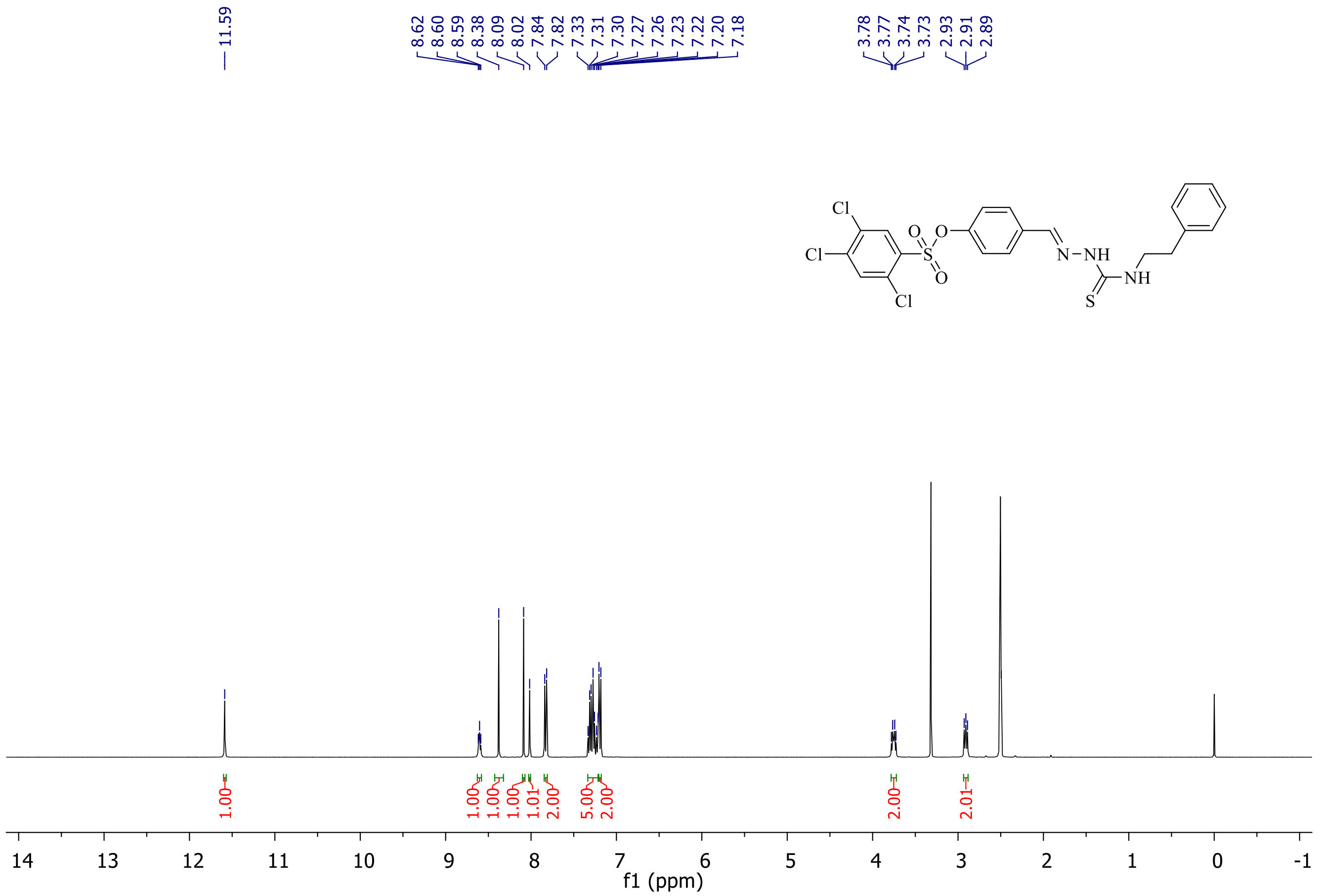

^{1}H NMR Spectrum of Compound 7

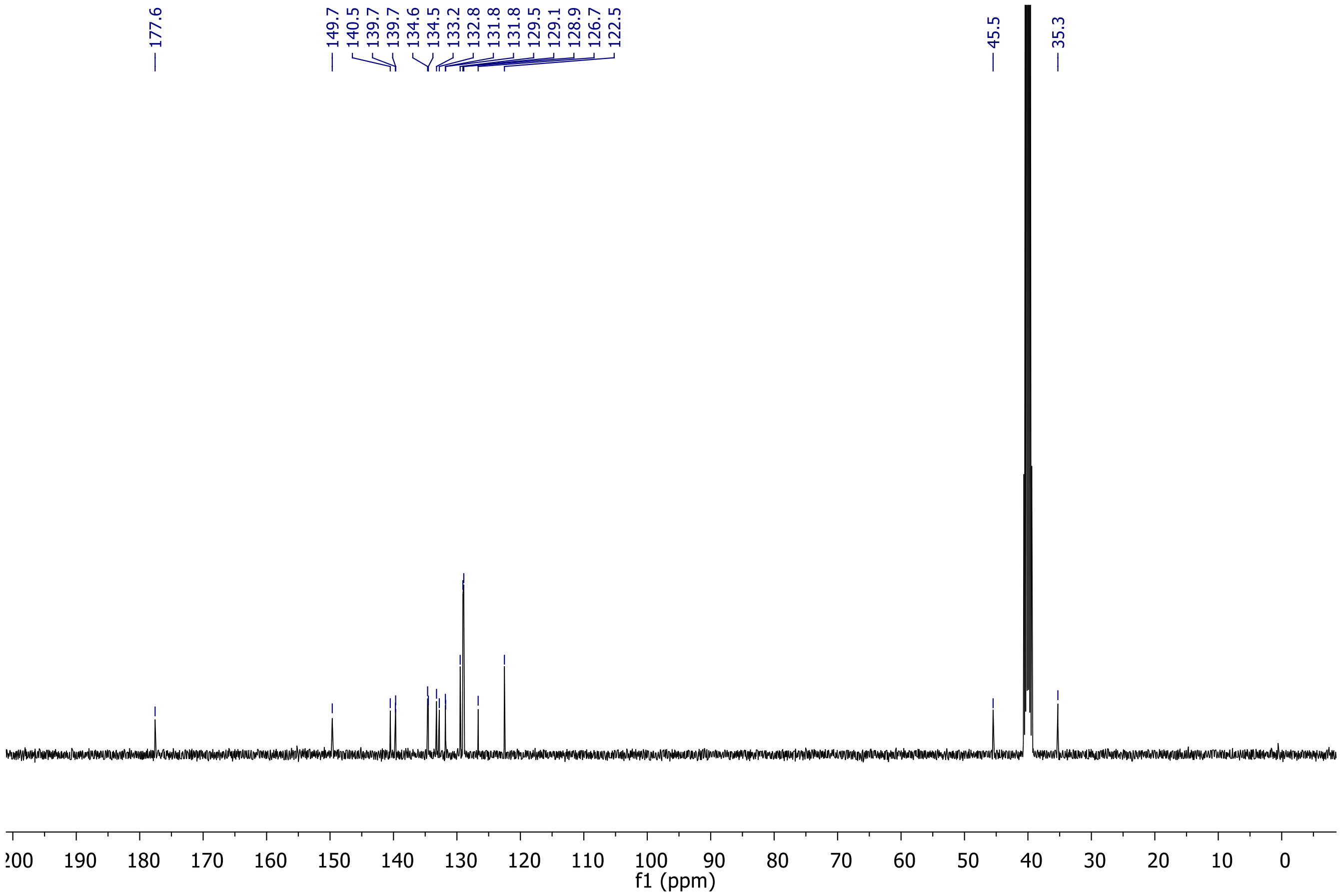

^{13}C NMR Spectrum of Compound 7

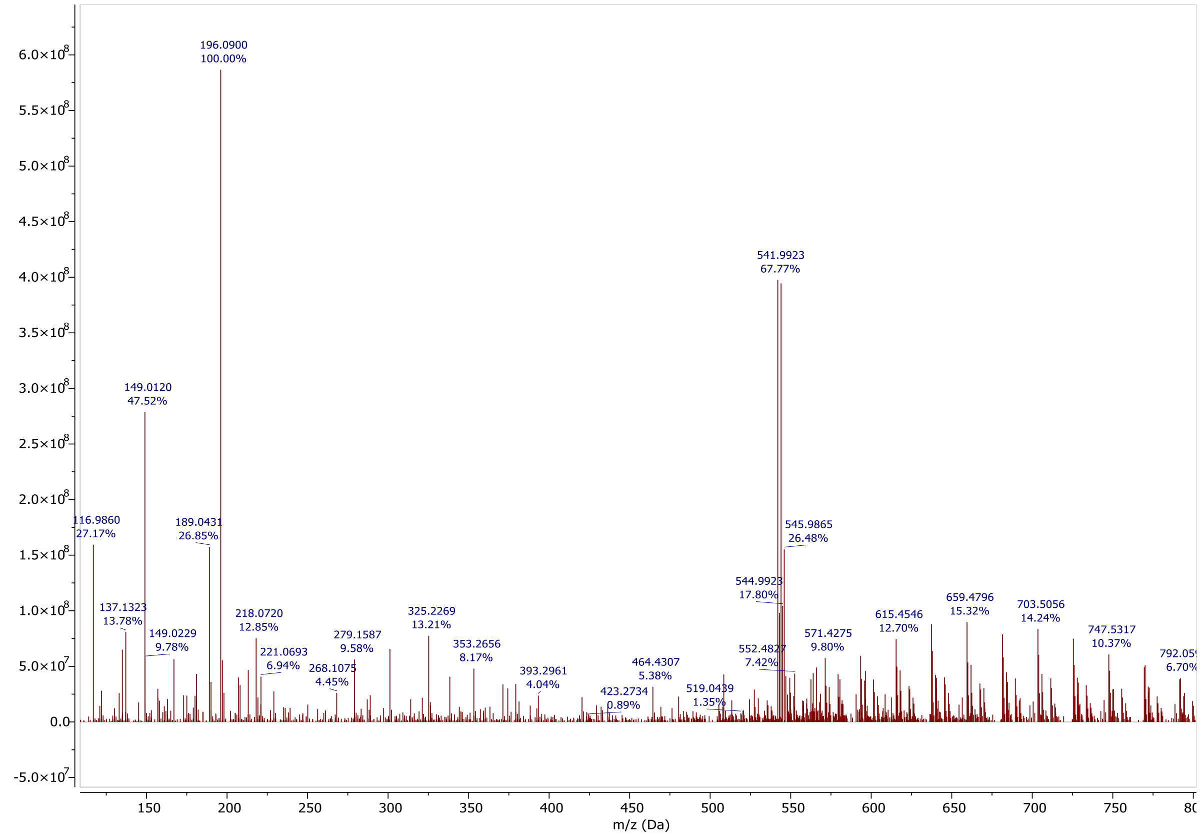

HRMS Spectrum of Compound 7

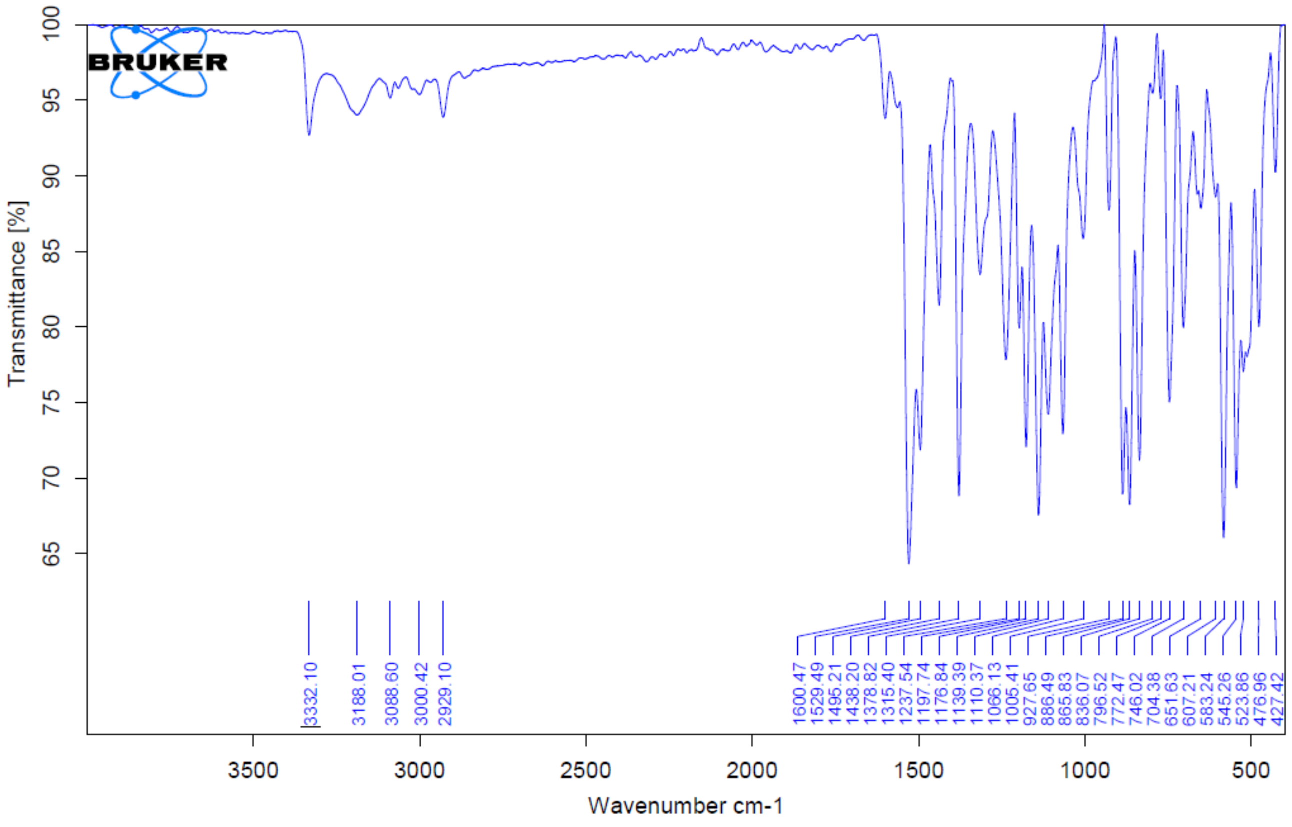

FTIR Spectrum of Compound 7

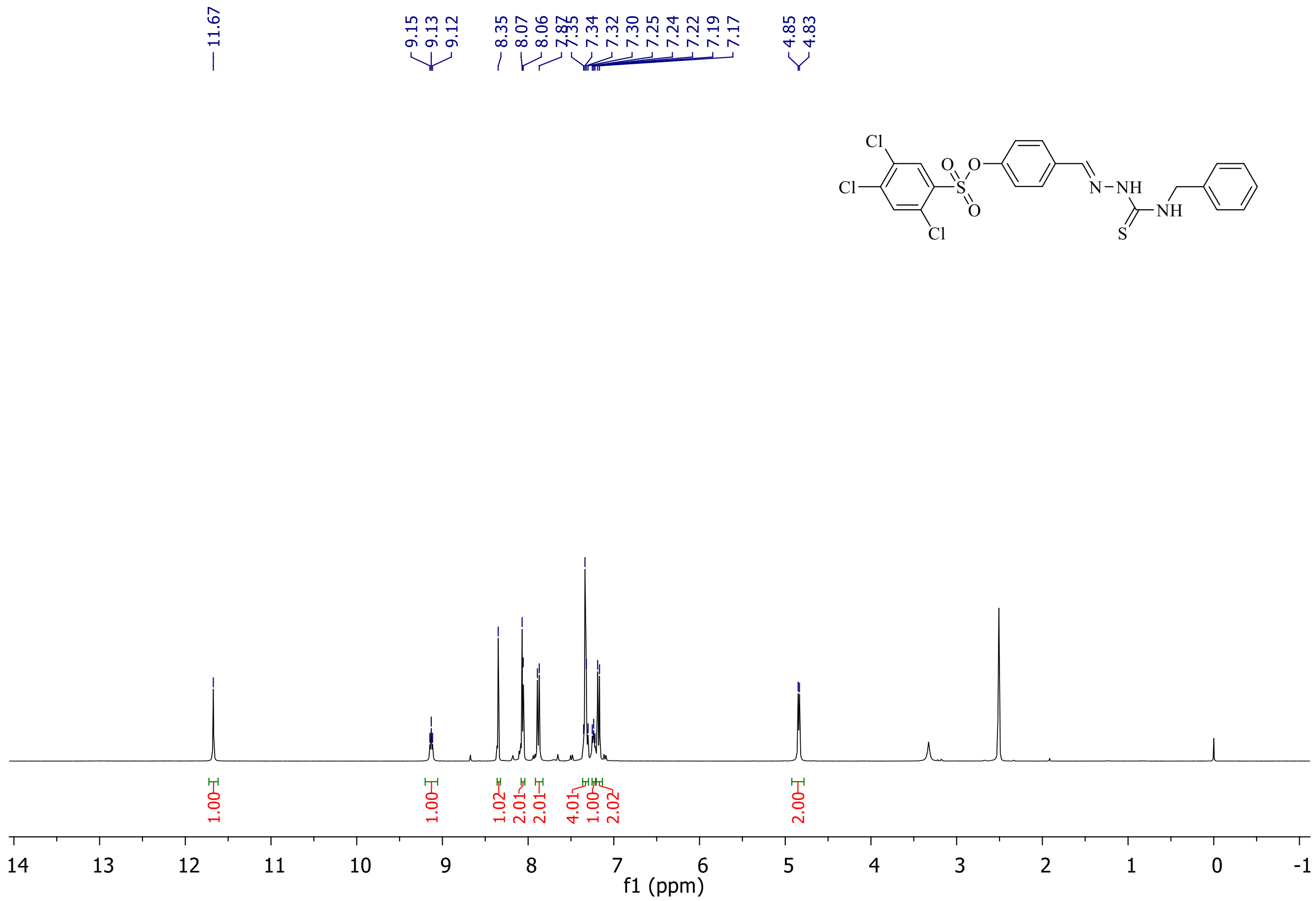

^{1}H NMR Spectrum of Compound 8

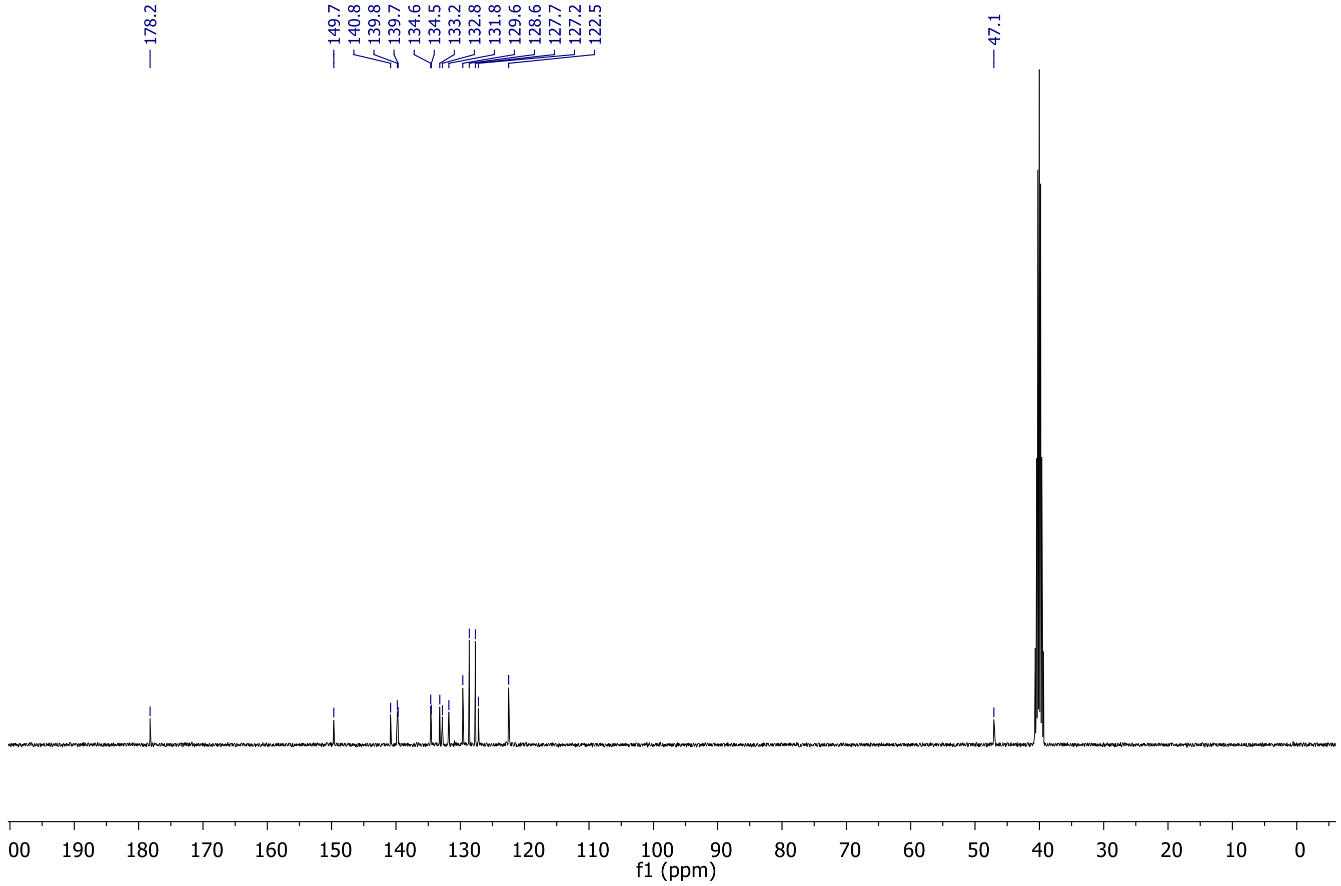

^{13}C NMR Spectrum of Compound 8

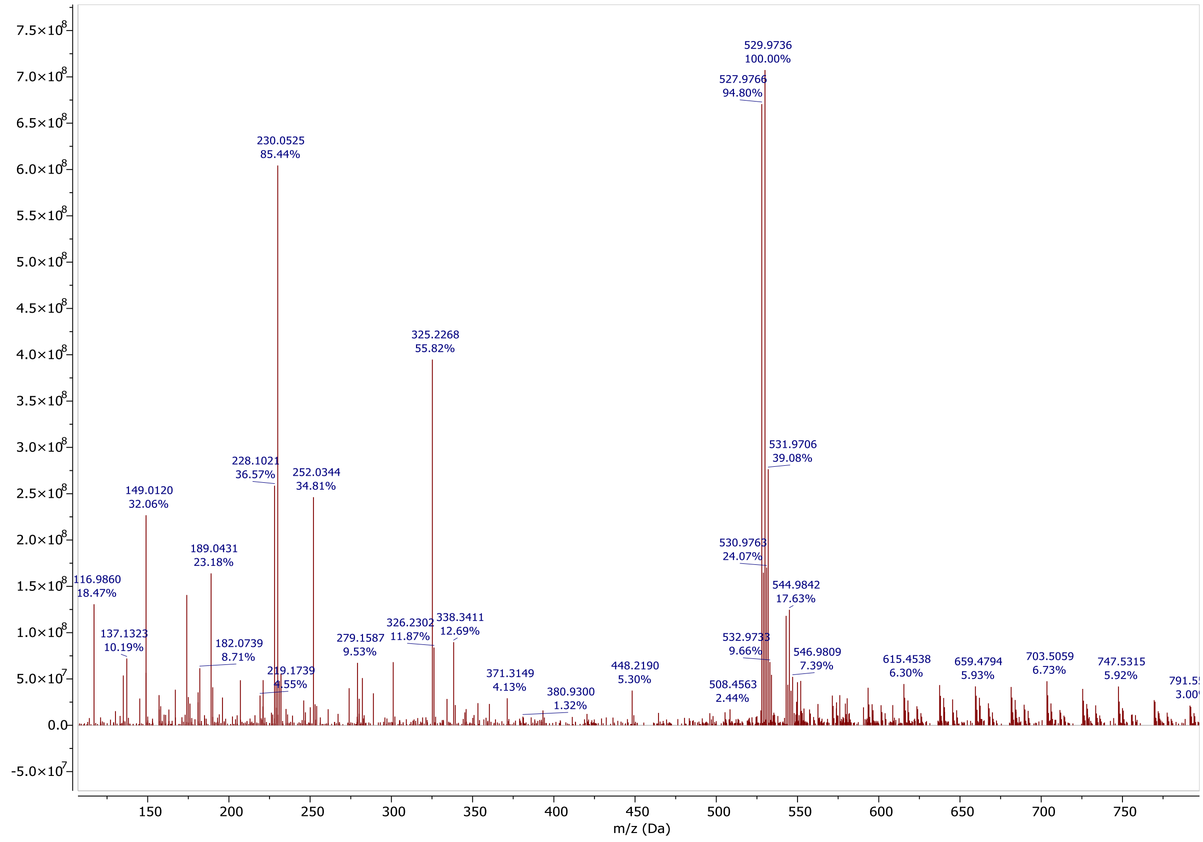

HRMS Spectrum of Compound 8

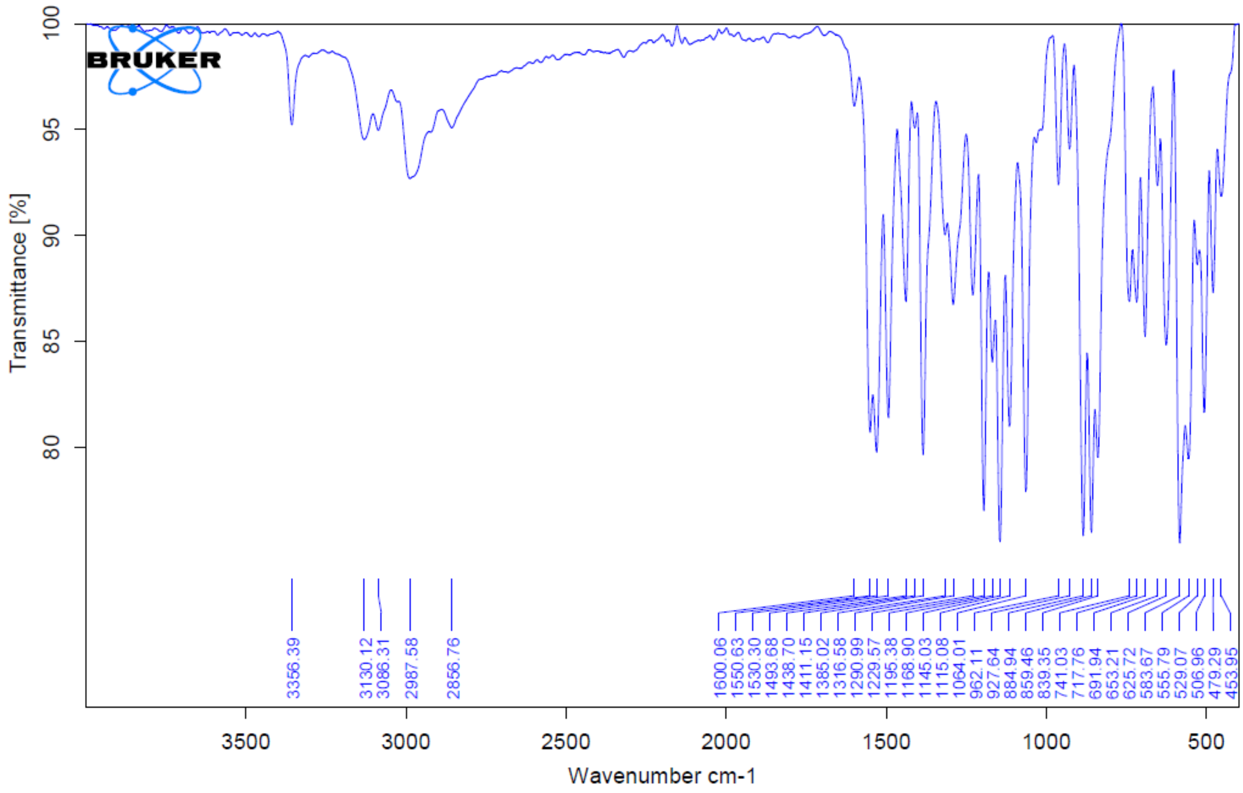

FTIR Spectrum of Compound 8

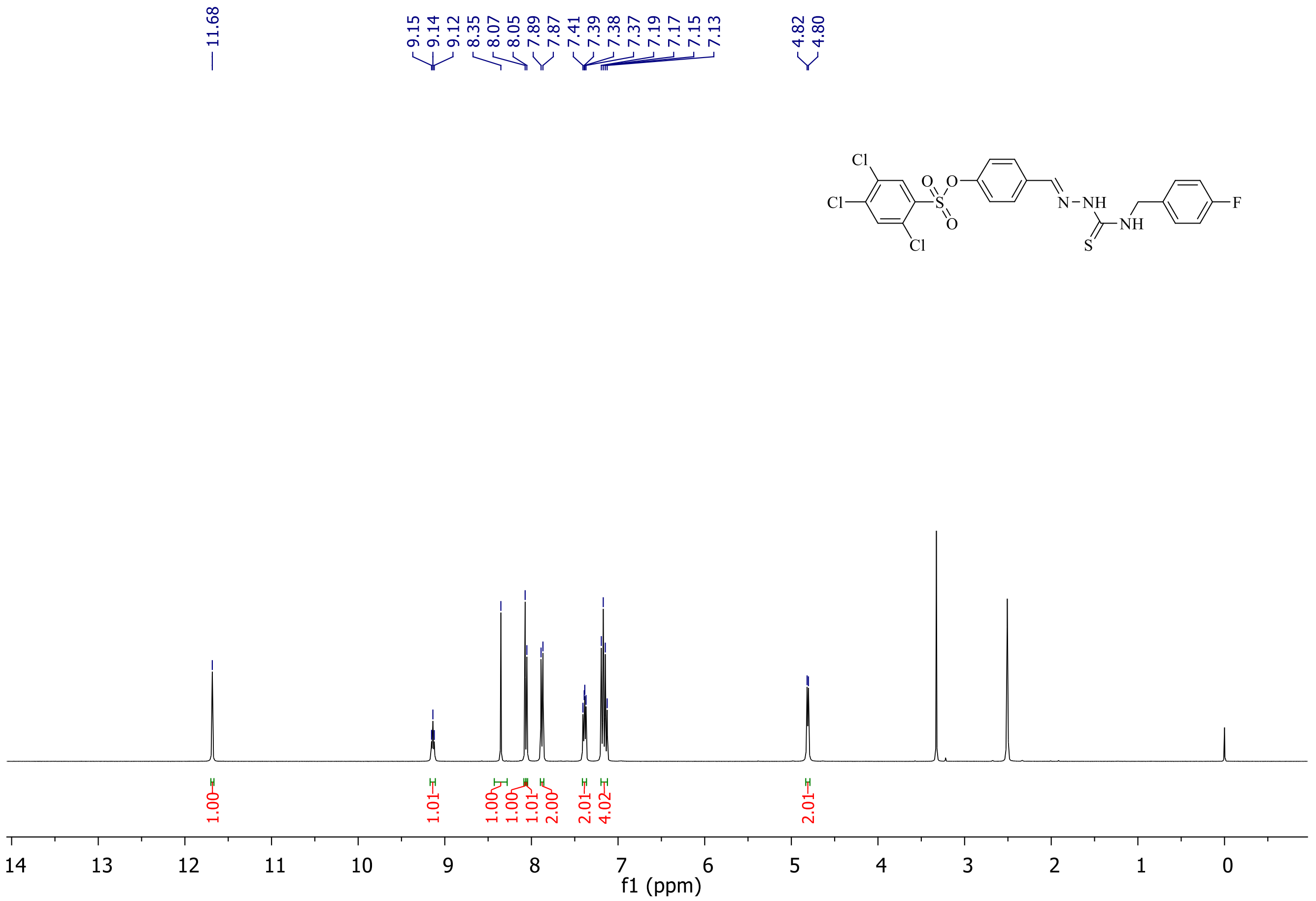

^{1}H NMR Spectrum of Compound 9

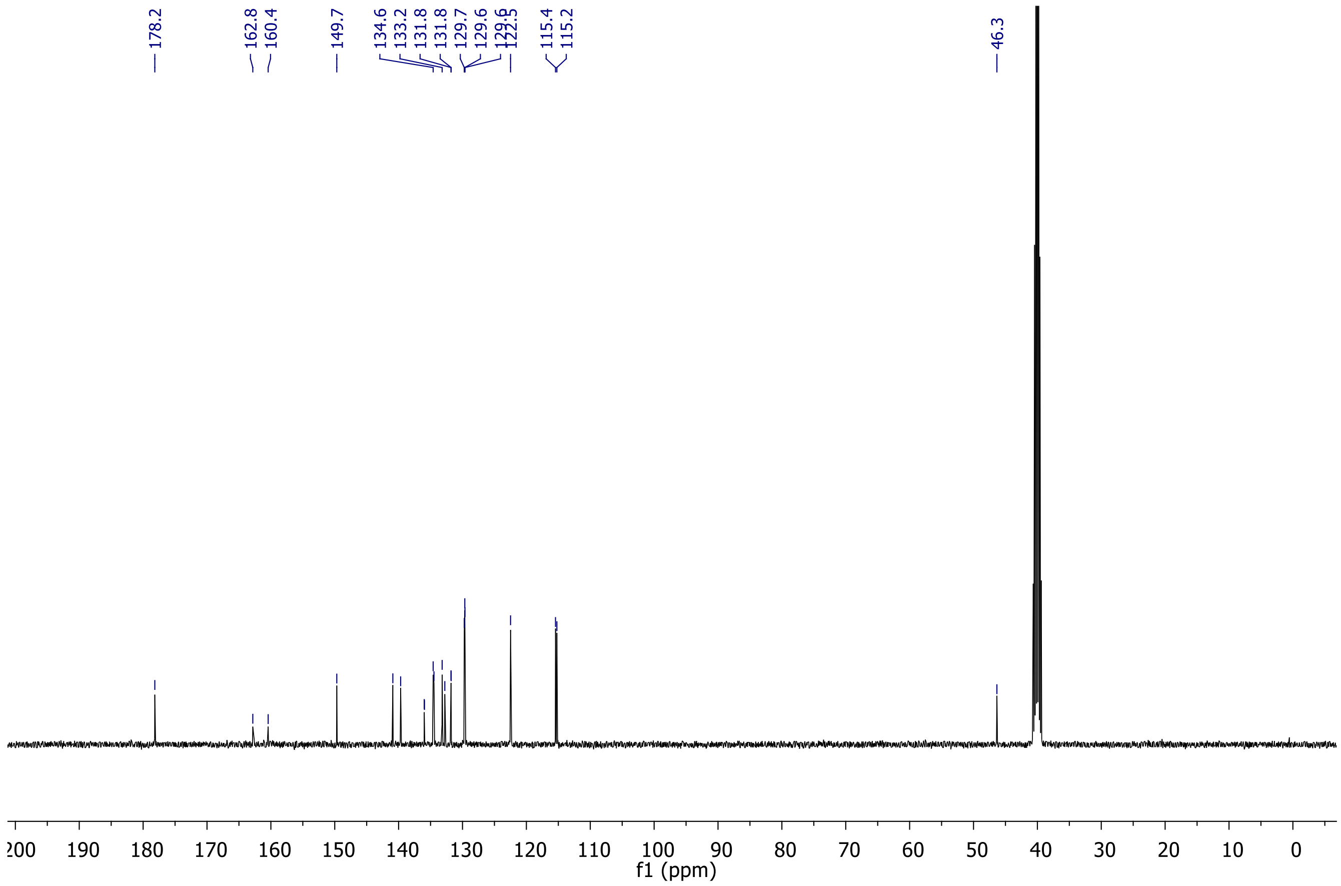

^{13}C NMR Spectrum of Compound 9

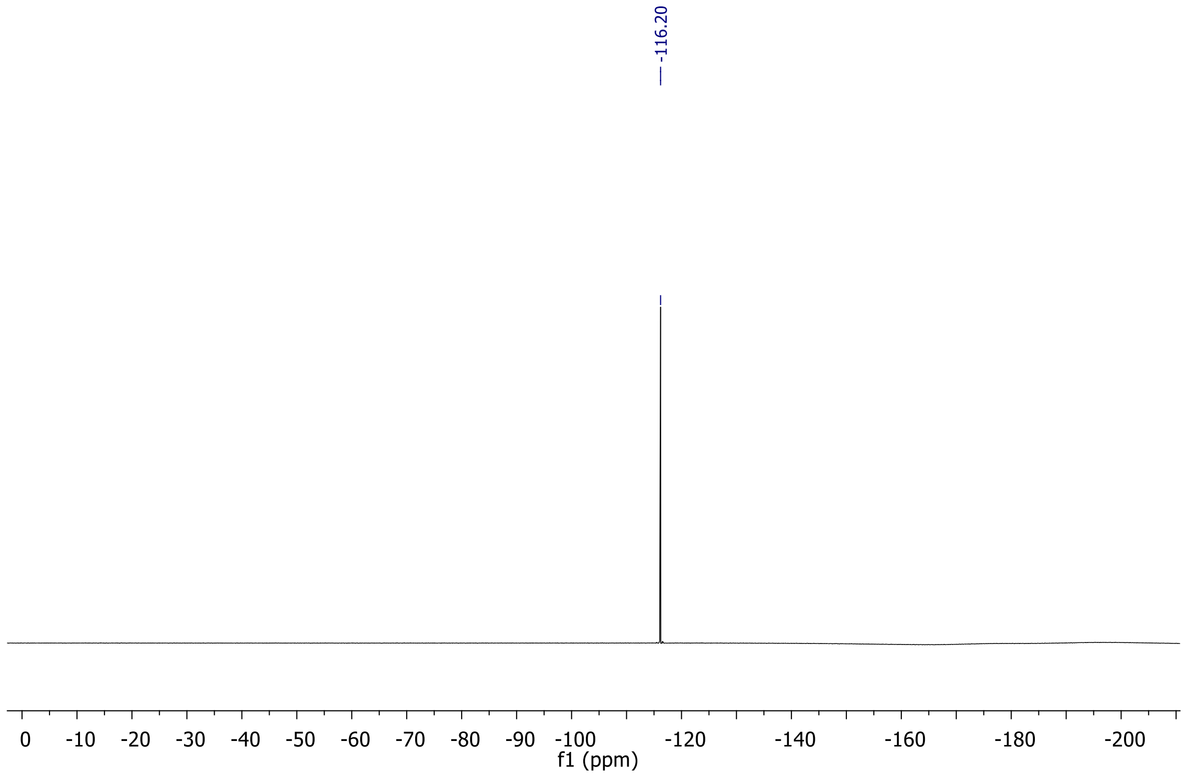

^{19}F NMR Spectrum of Compound 9

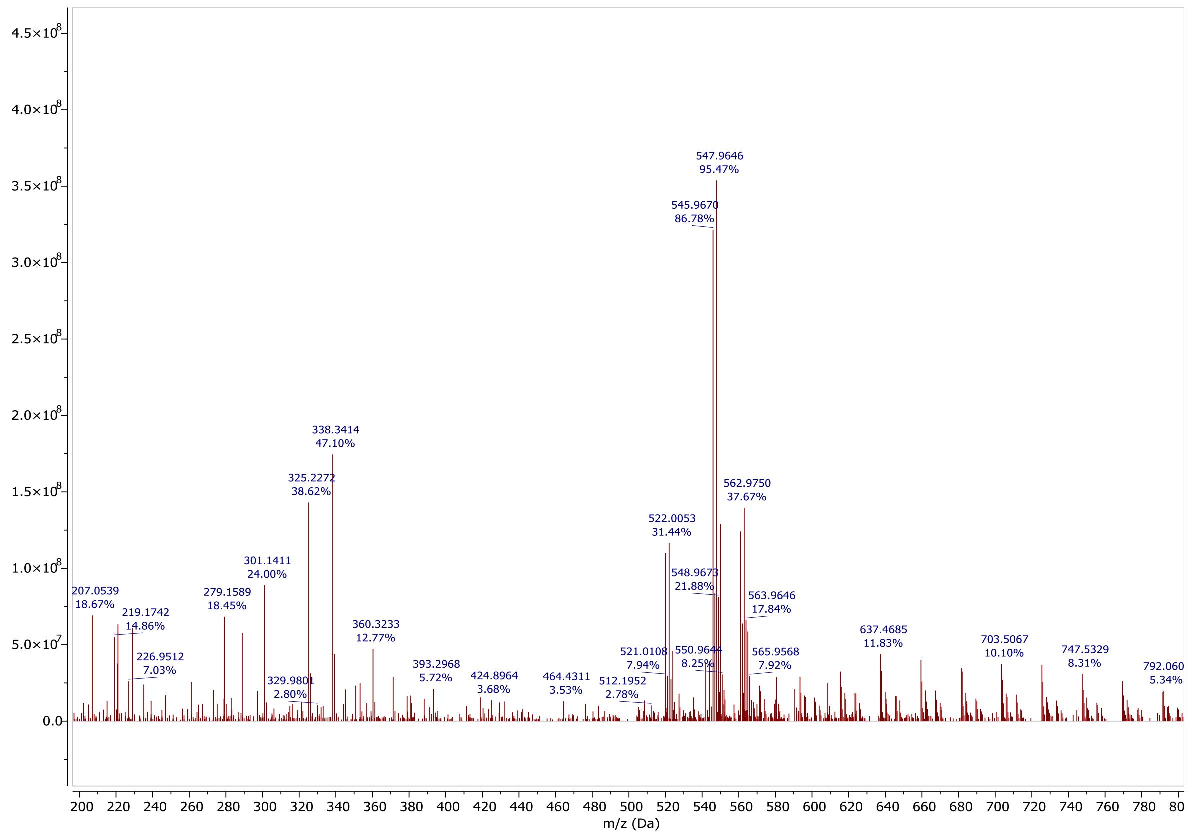

HRMS Spectrum of Compound 9

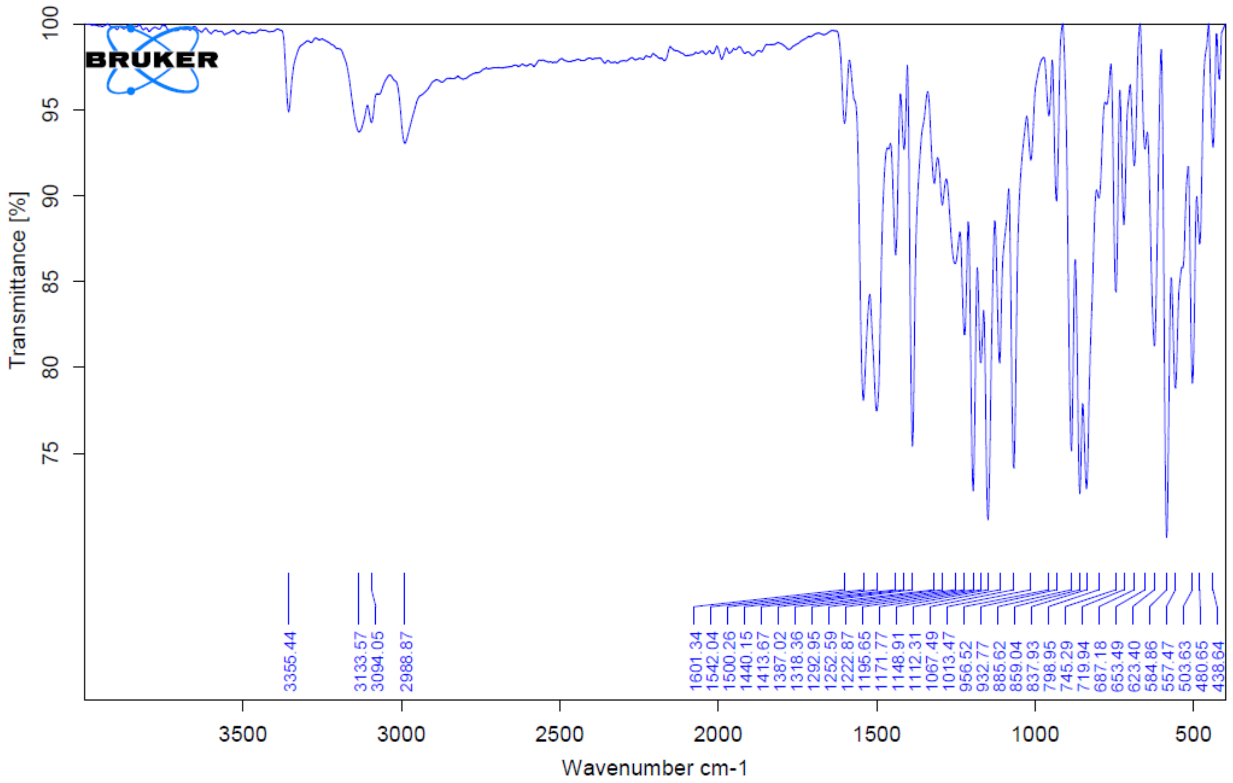

FTIR Spectrum of Compound 9

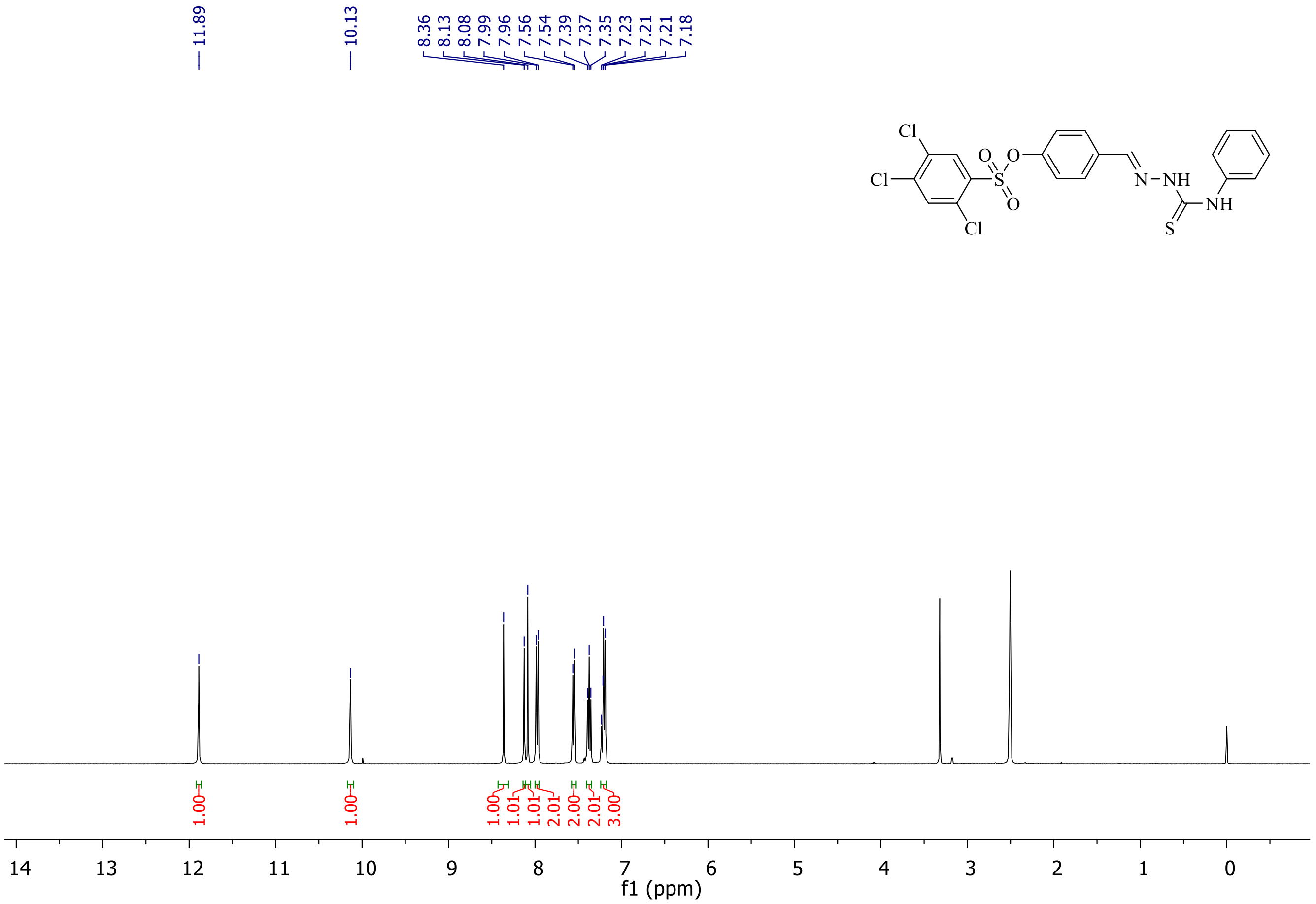

^{1}H NMR Spectrum of Compound 10

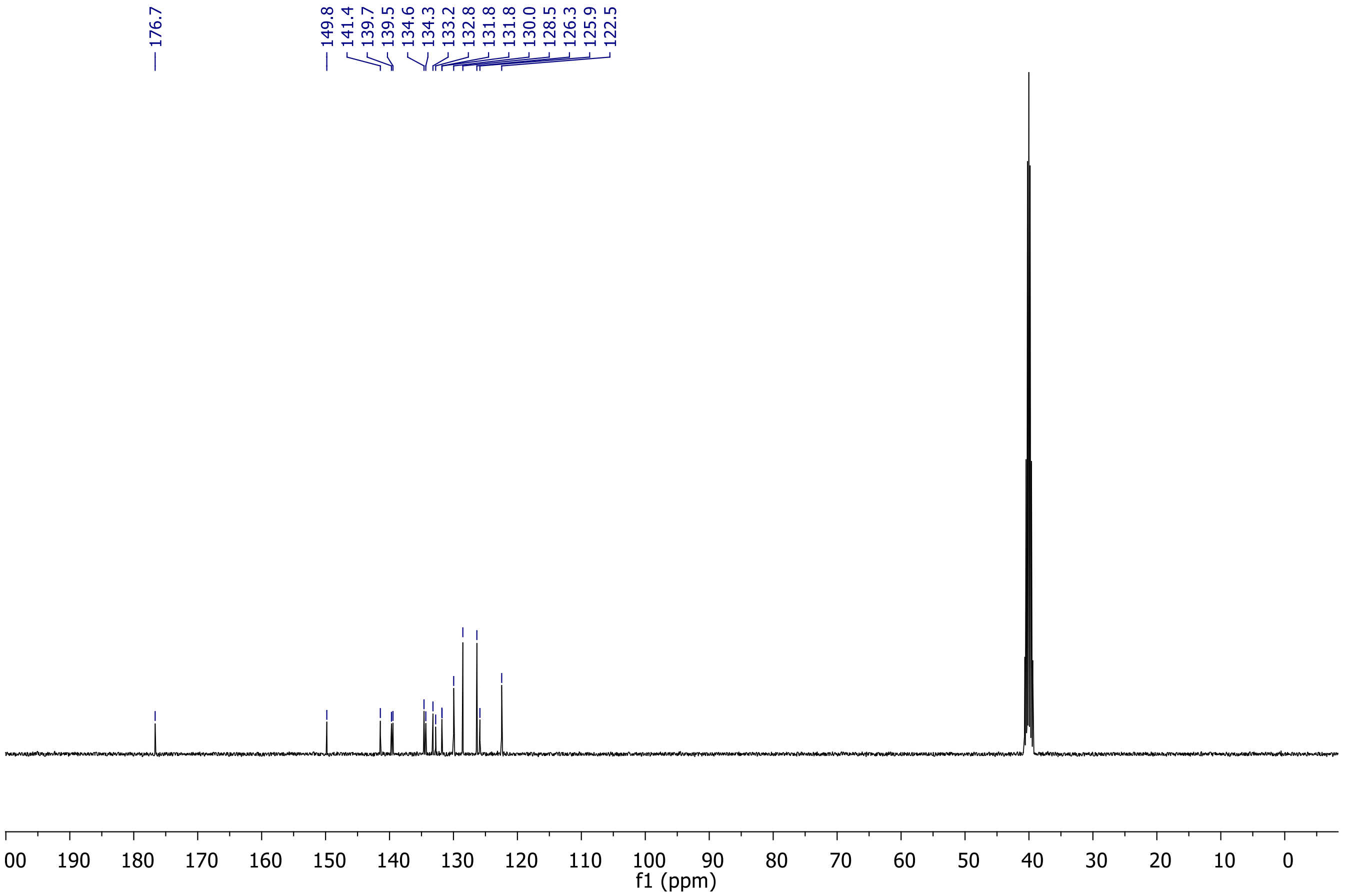

^{13}C NMR Spectrum of Compound 10

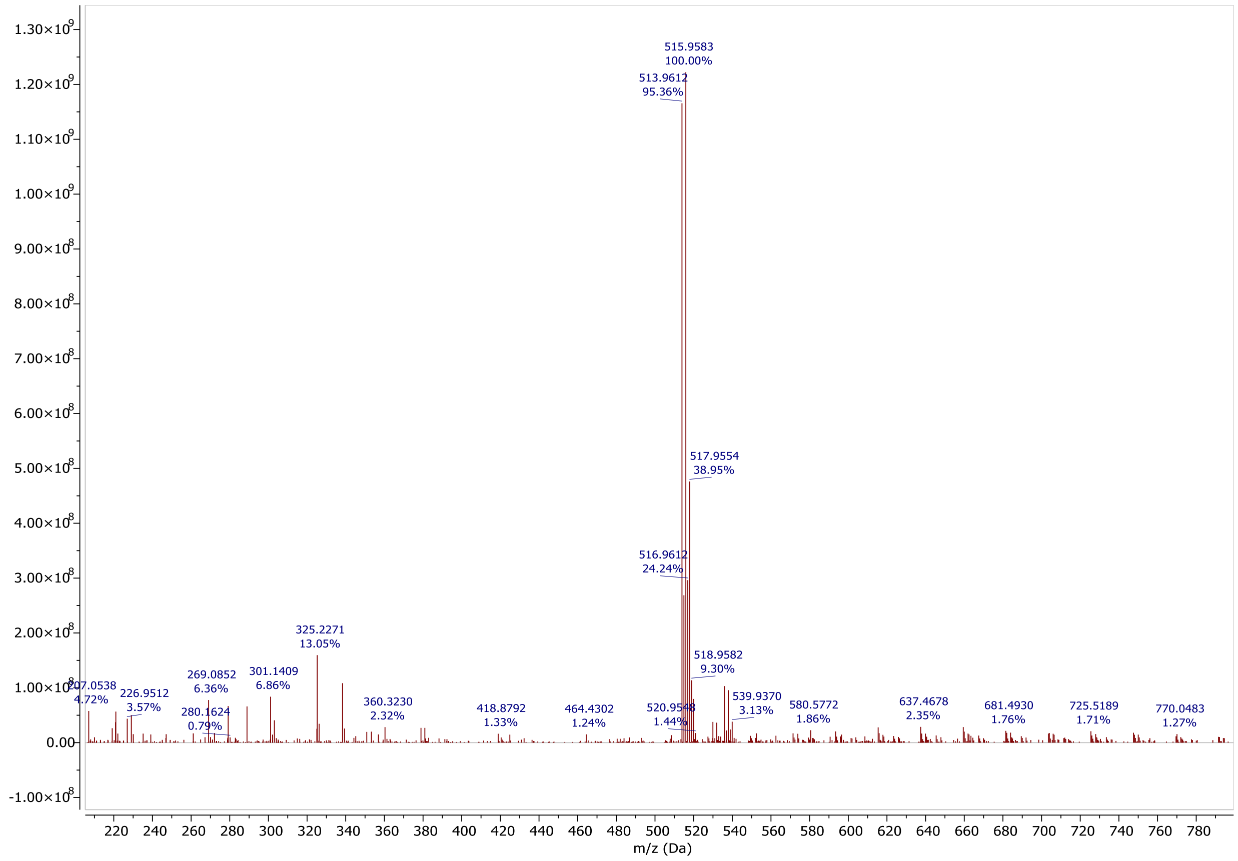

HRMS Spectrum of Compound 10

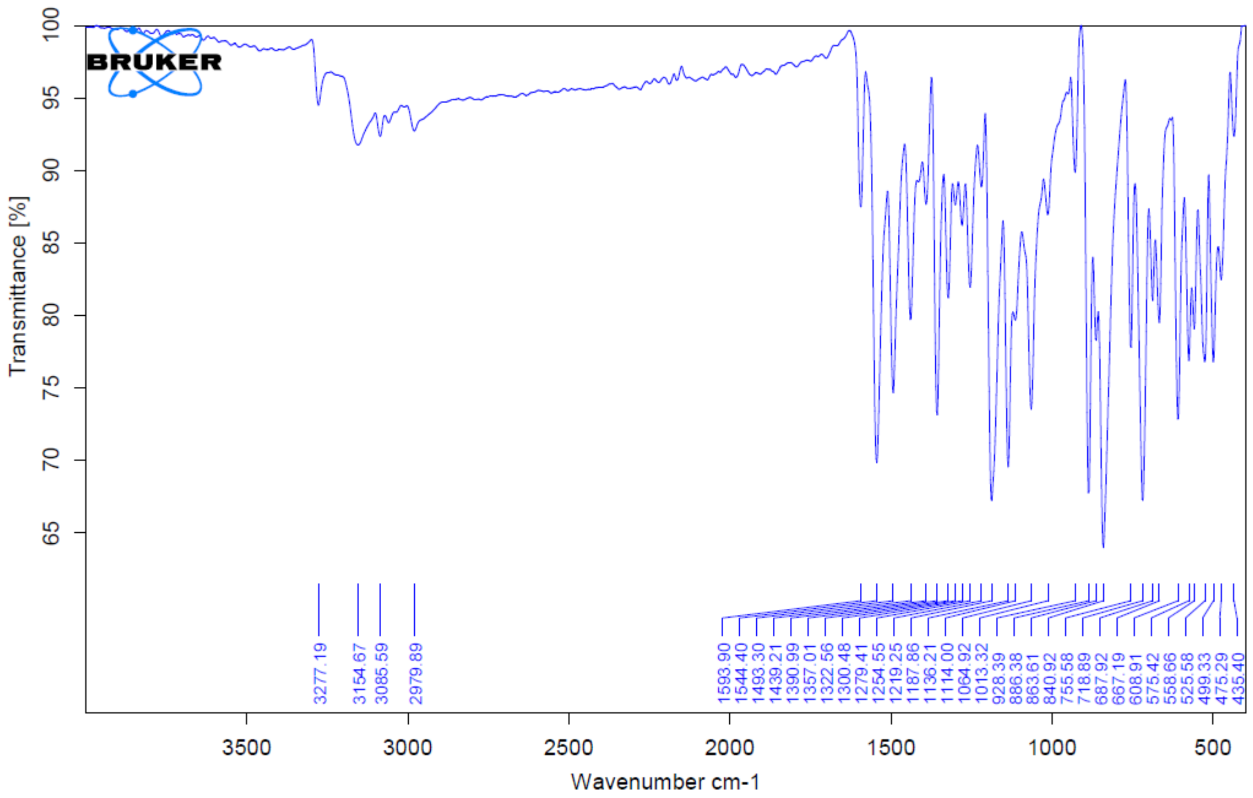

FTIR Spectrum of Compound 10

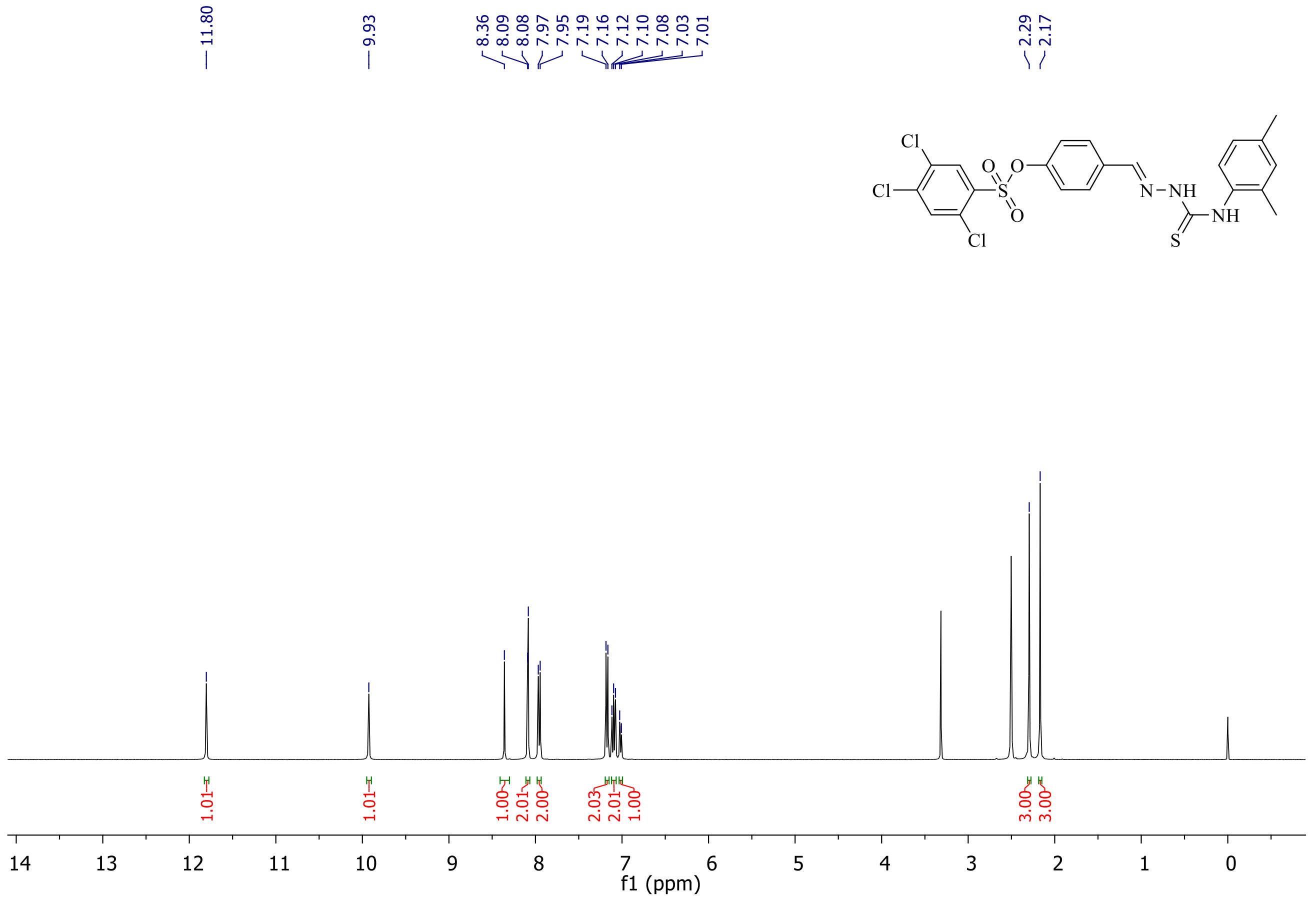

^{1}H NMR Spectrum of Compound 11

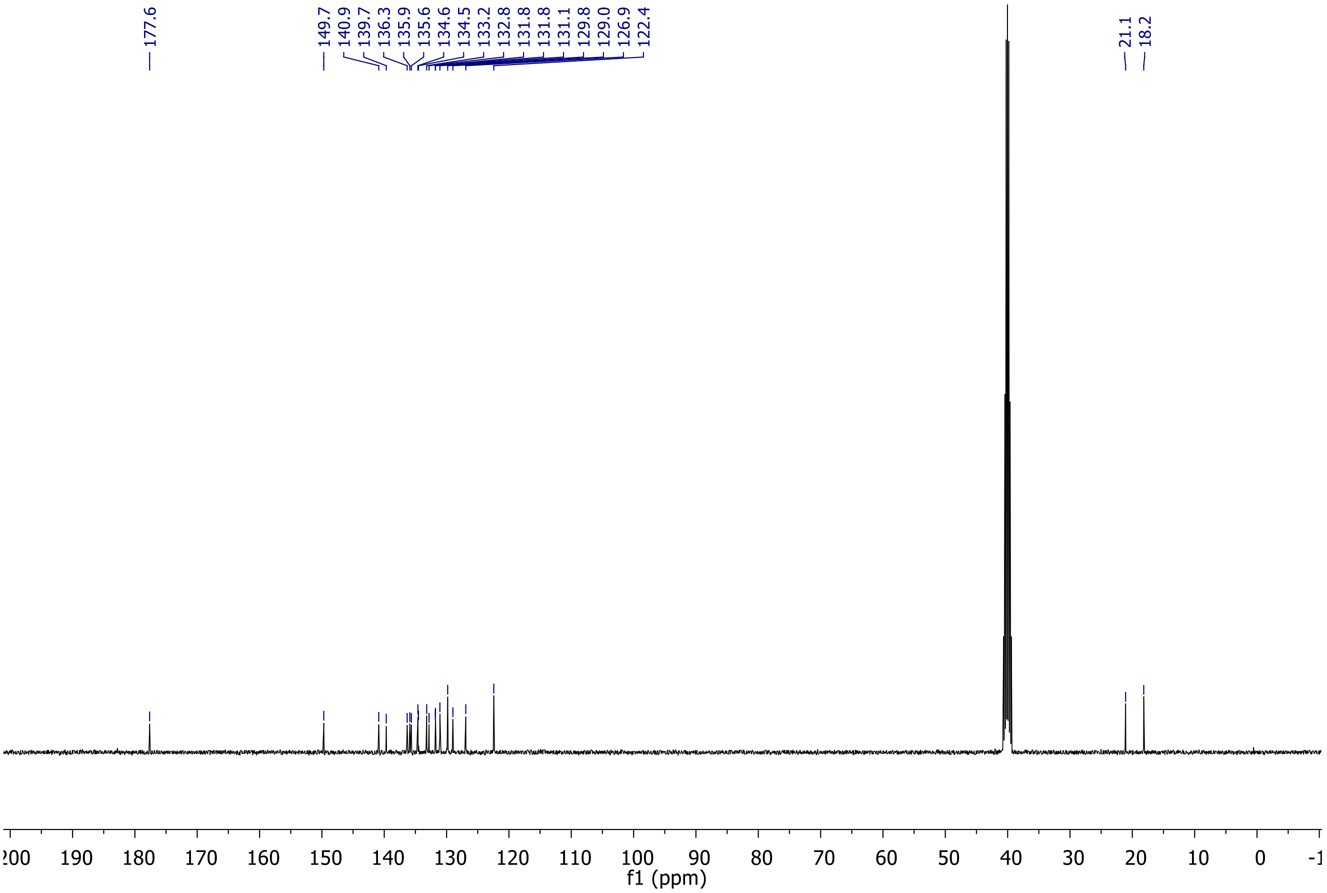

^{13}C NMR Spectrum of Compound 11

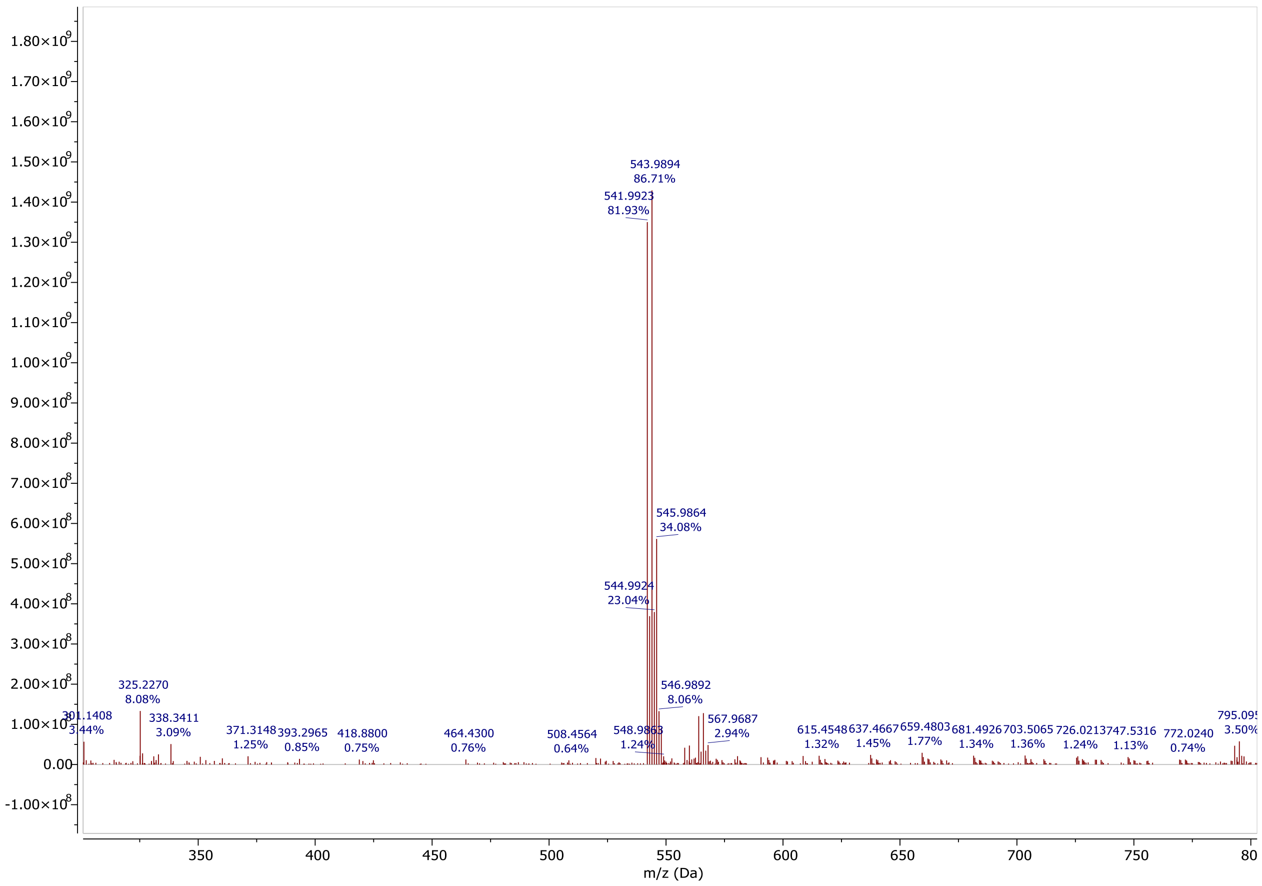

HRMS Spectrum of Compound 11

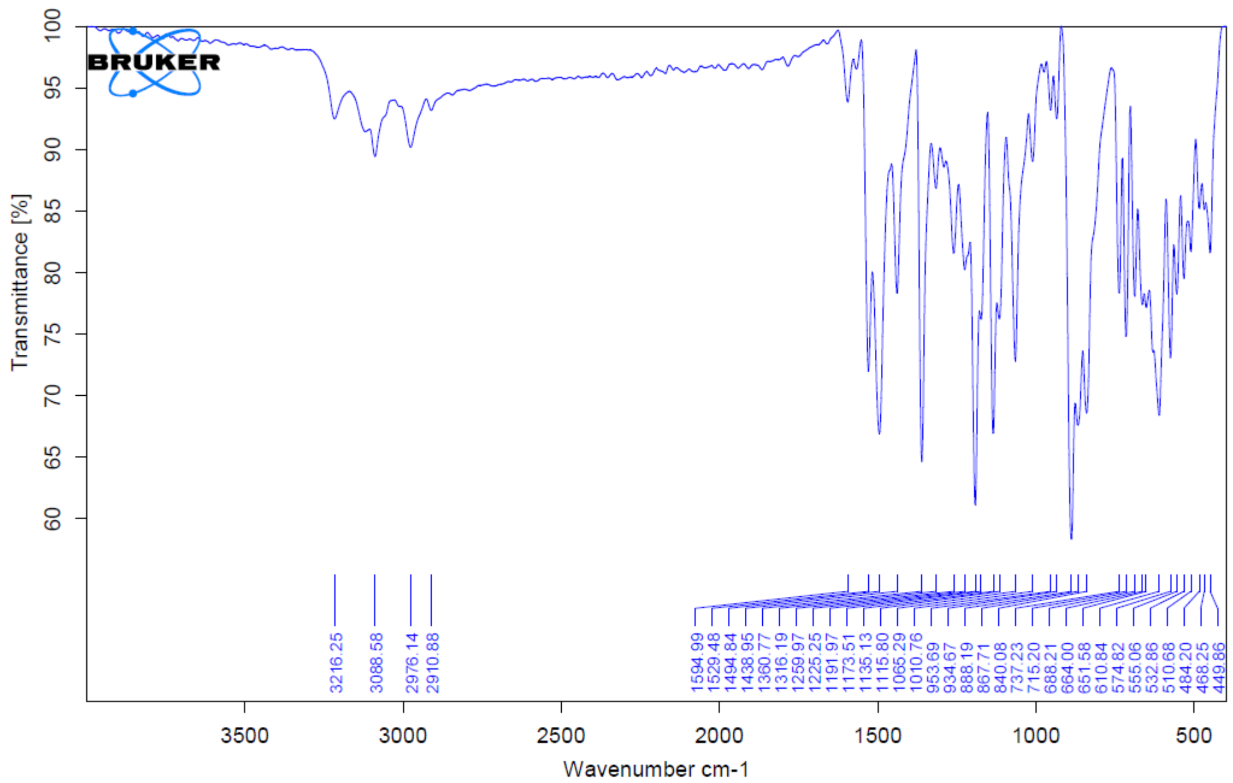

FTIR Spectrum of Compound 11

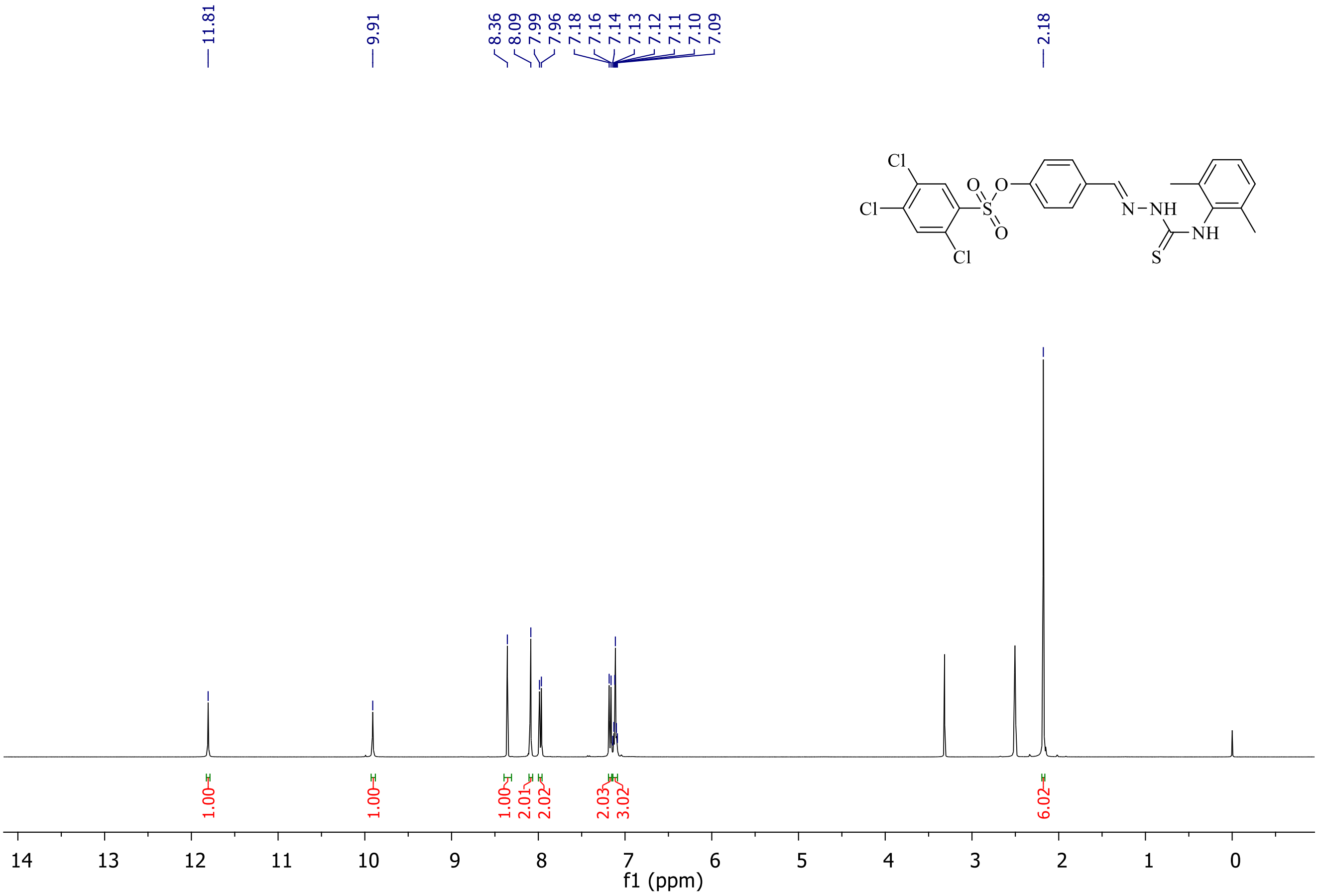

^{1}H NMR Spectrum of Compound 12

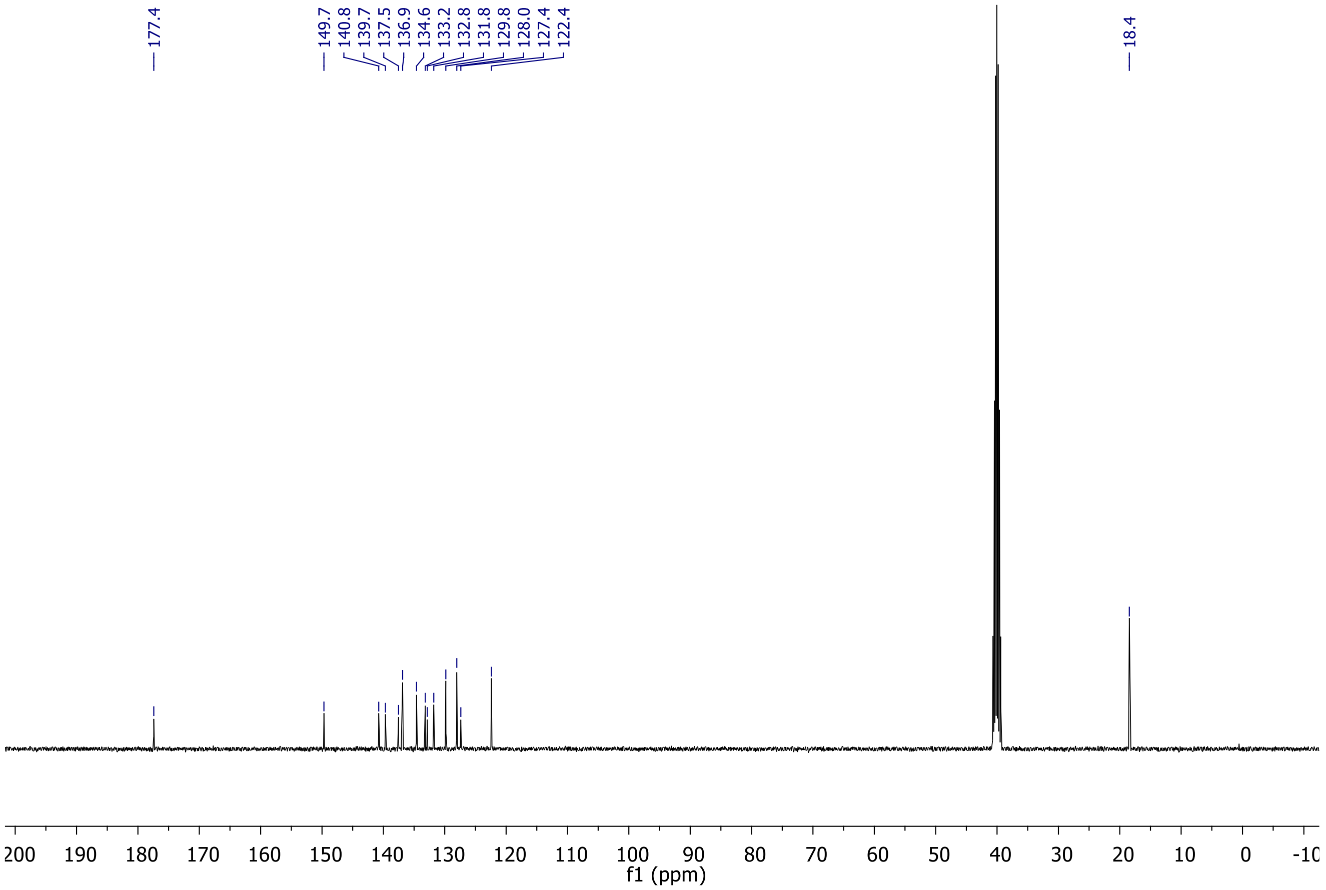

^{13}C NMR Spectrum of Compound 12

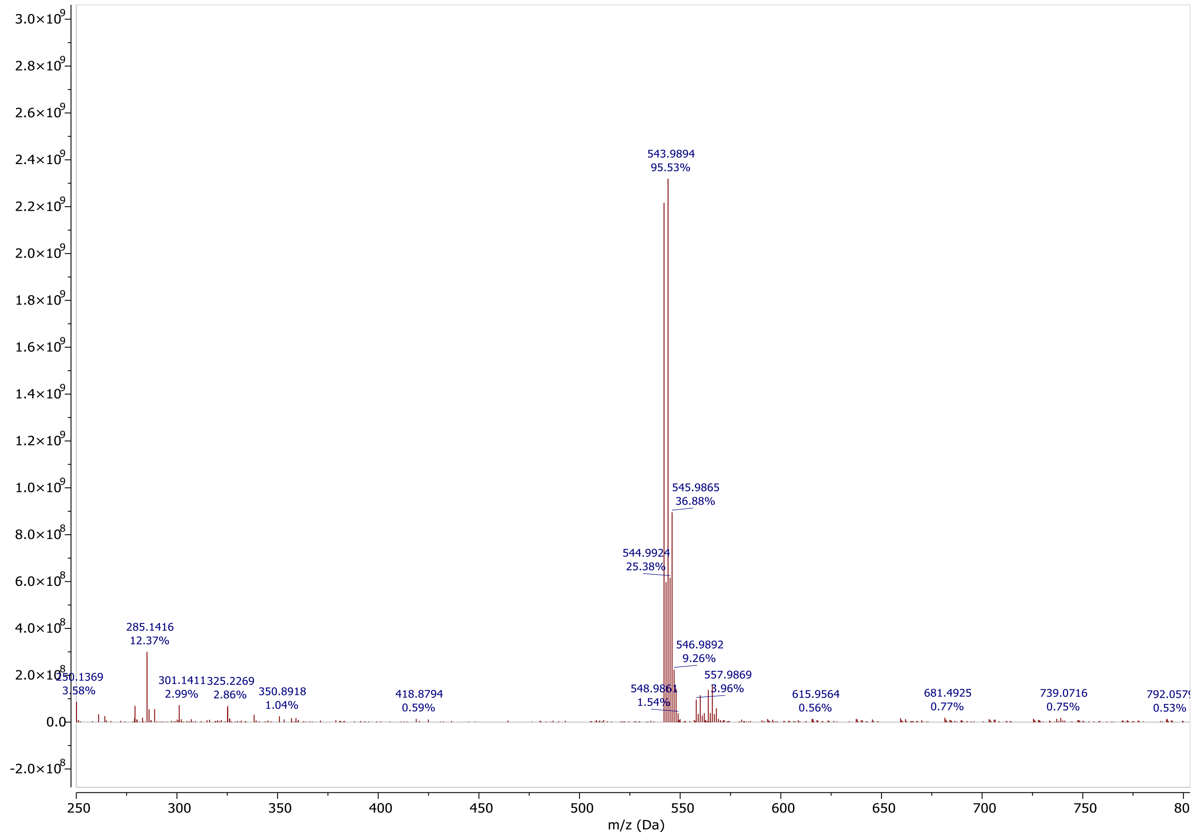

HRMS Spectrum of Compound 12

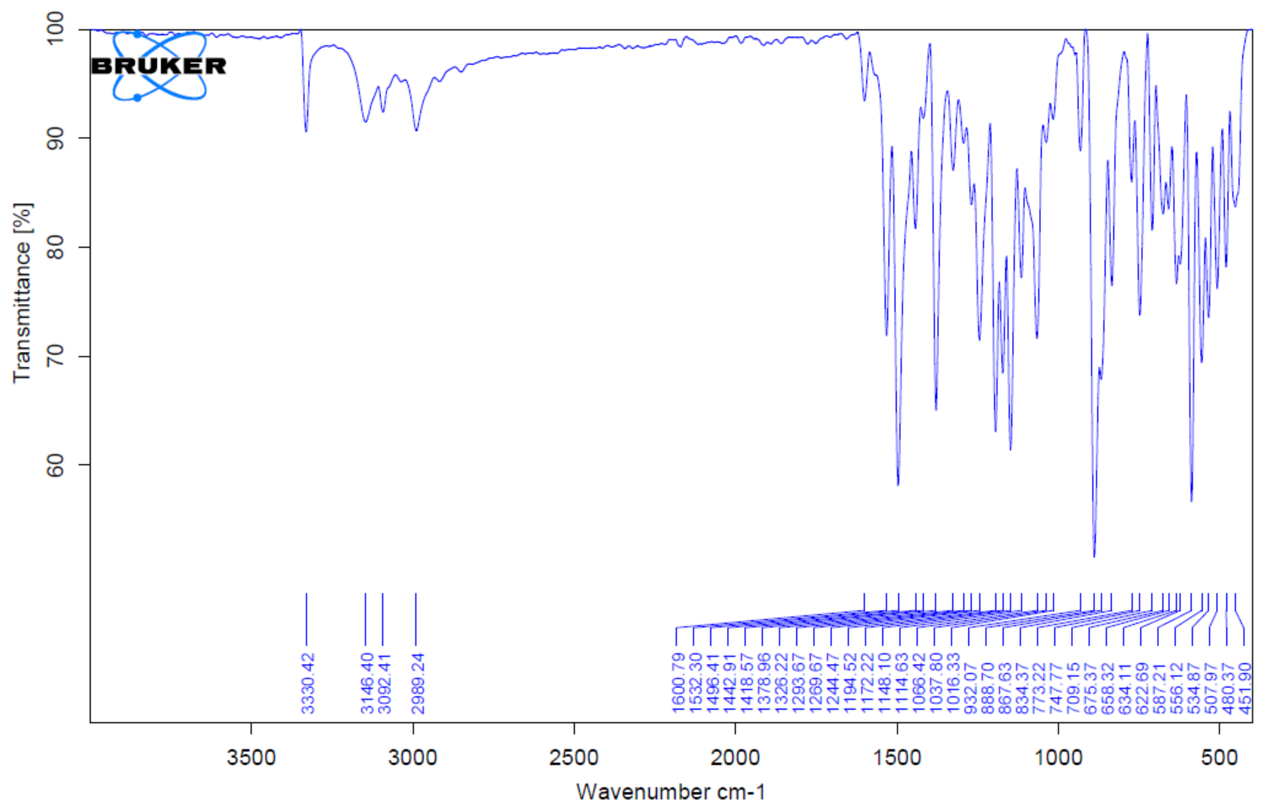

FTIR Spectrum of Compound 12

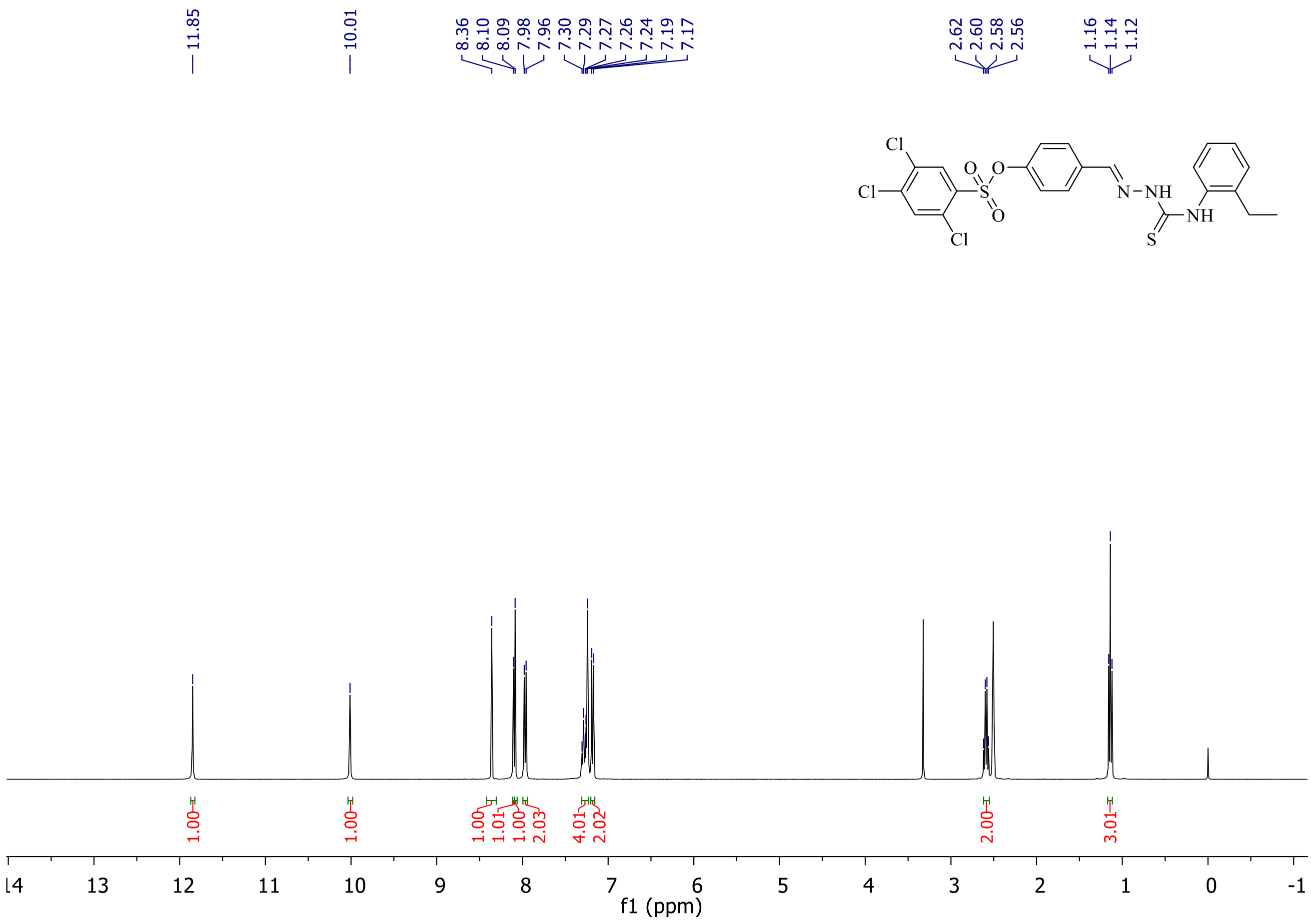

^{1}H NMR Spectrum of Compound 13

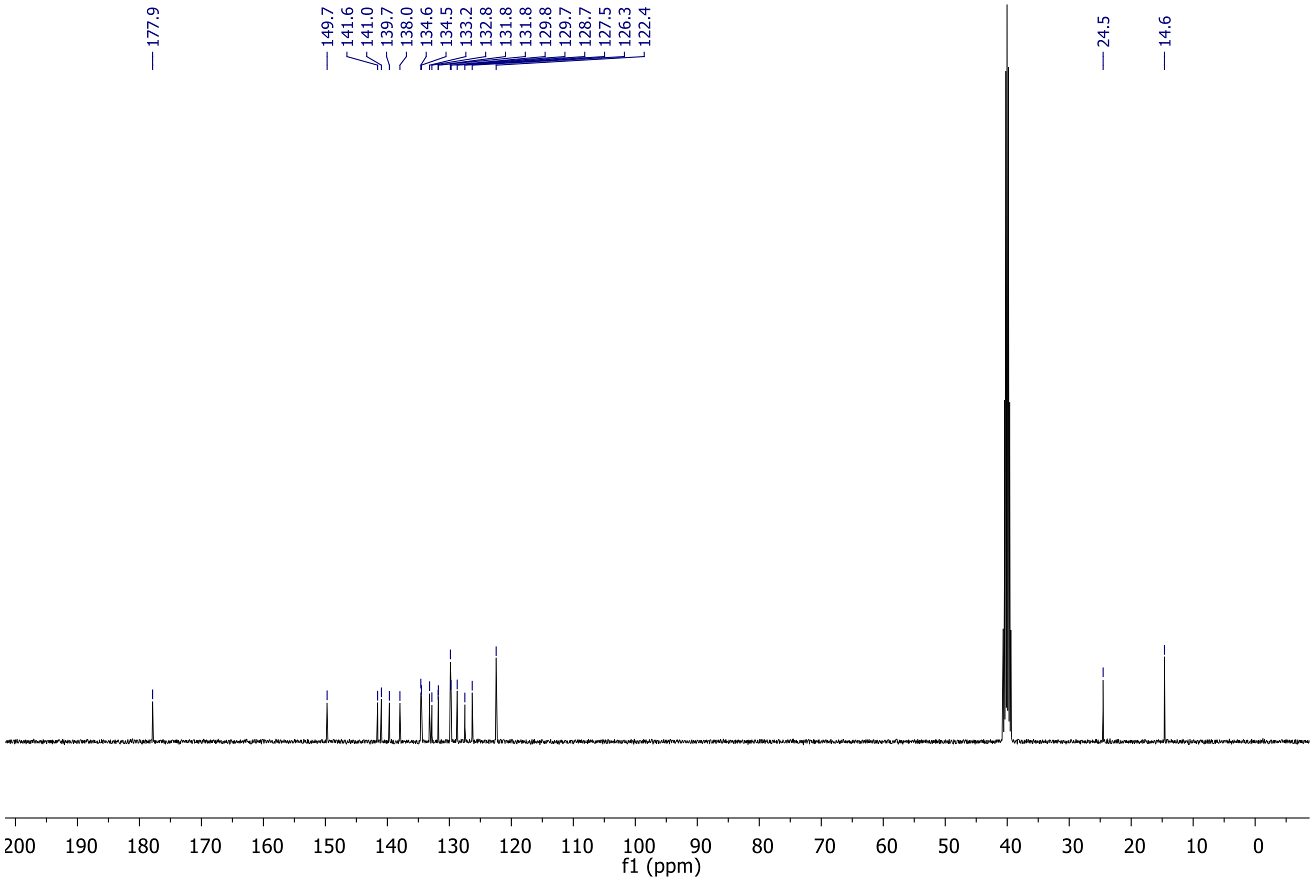

^{13}C NMR Spectrum of Compound 13

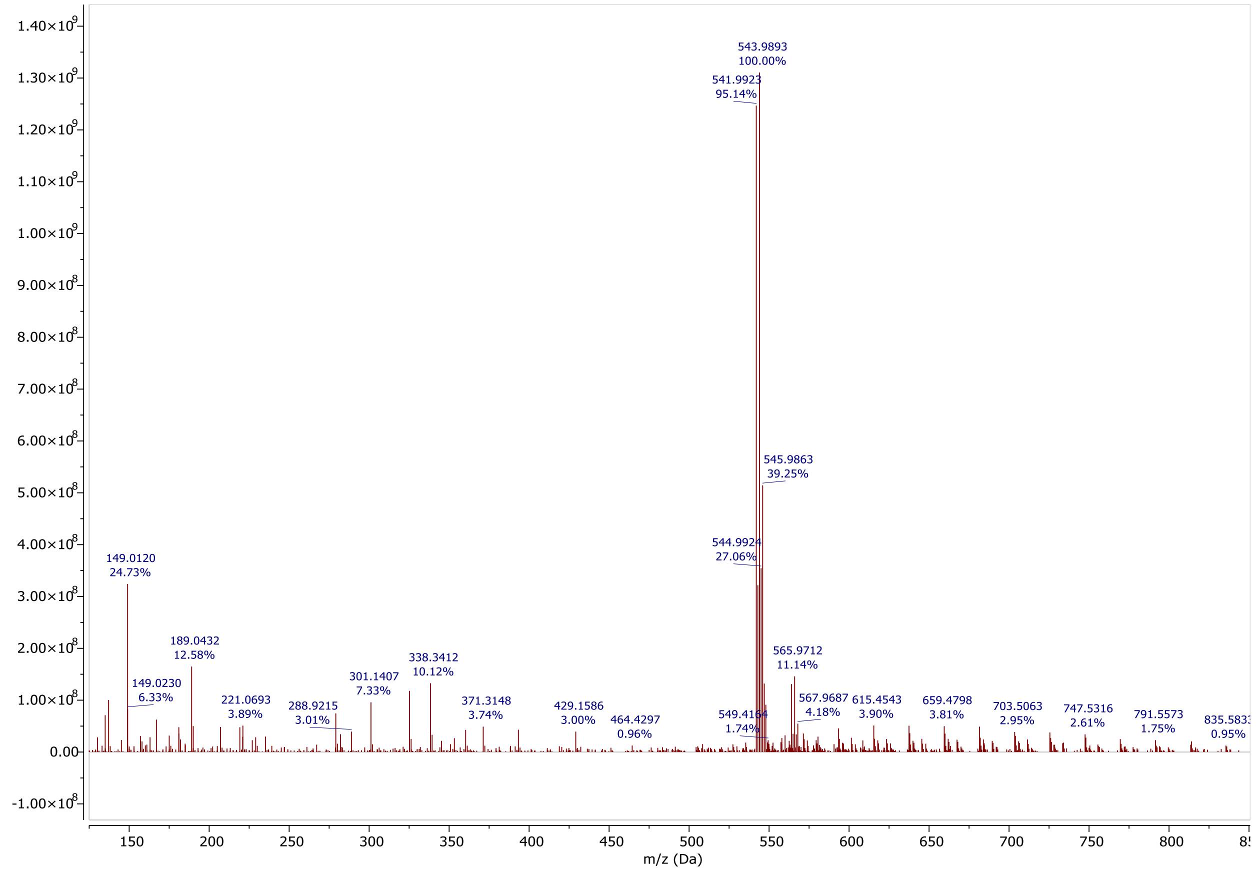

HRMS Spectrum of Compound 13

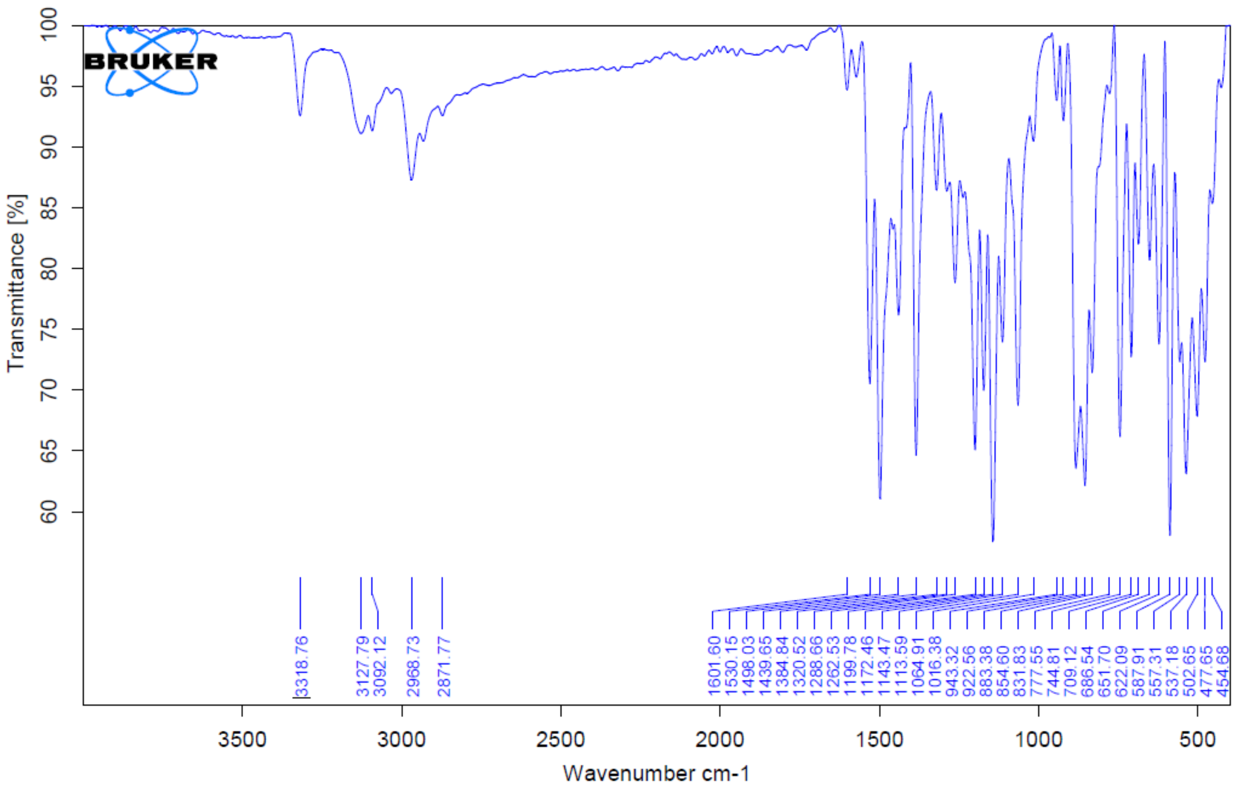

FTIR Spectrum of Compound 13

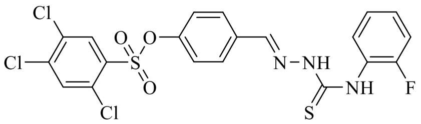

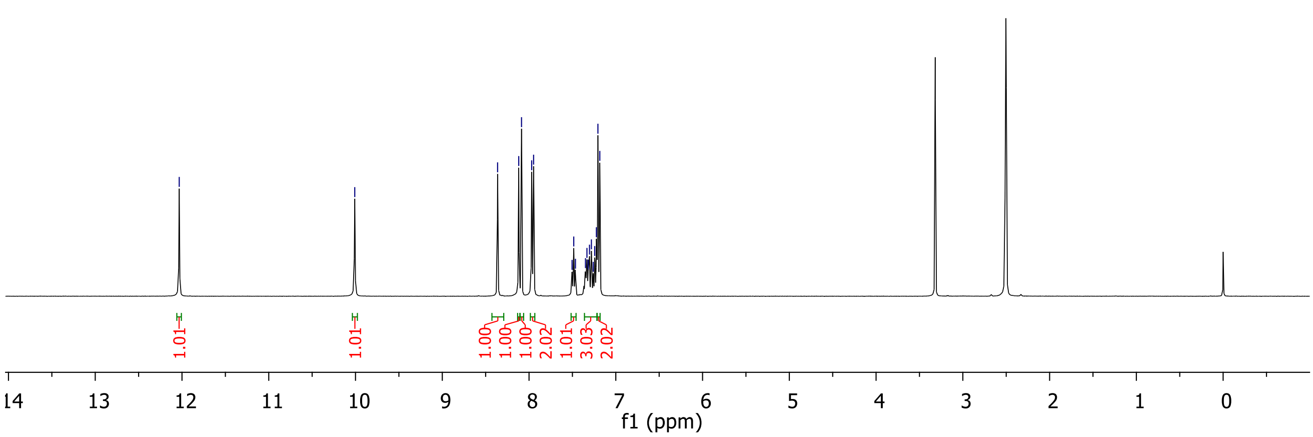

^{1}H NMR Spectrum of Compound 14

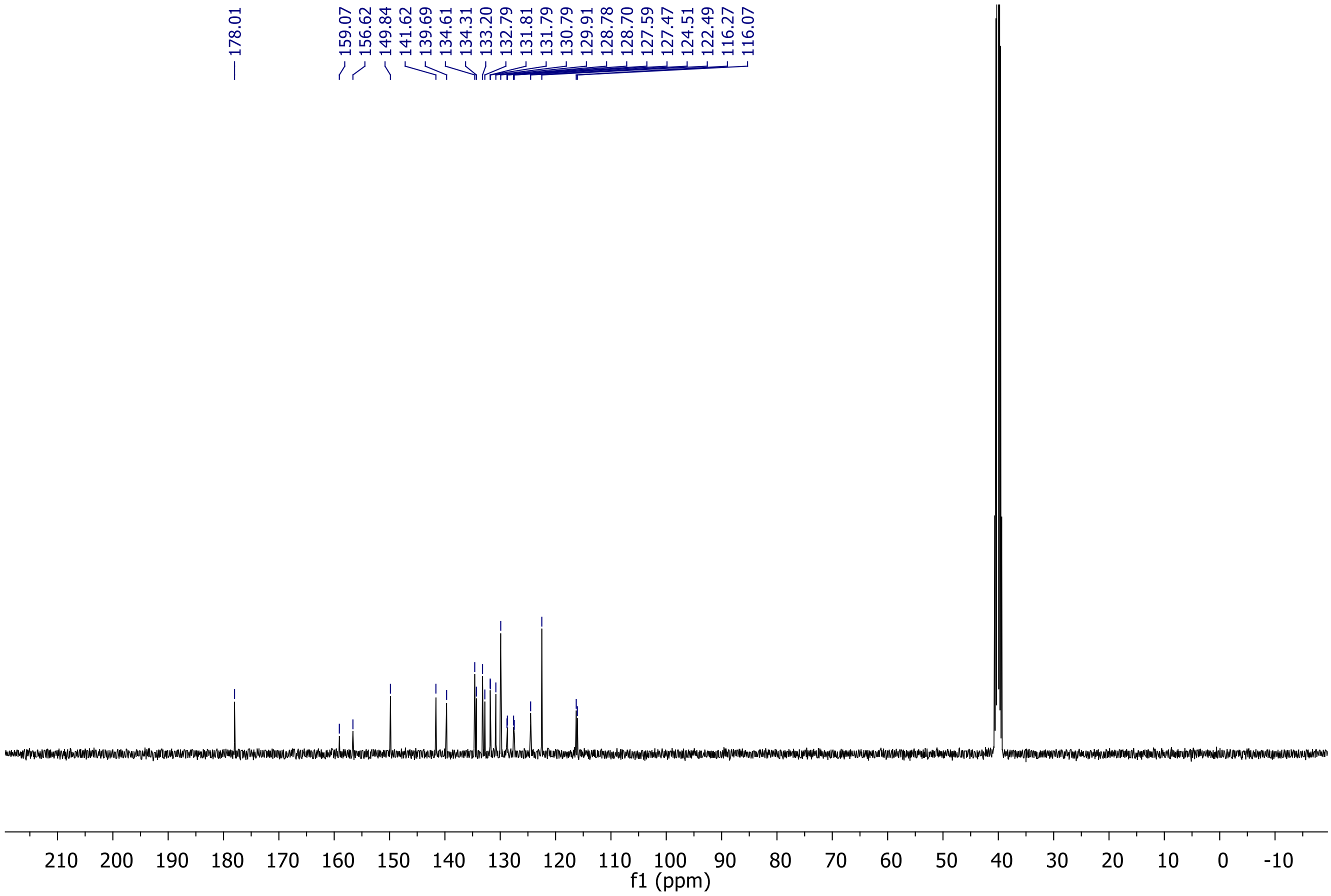

^{13}C NMR Spectrum of Compound 14

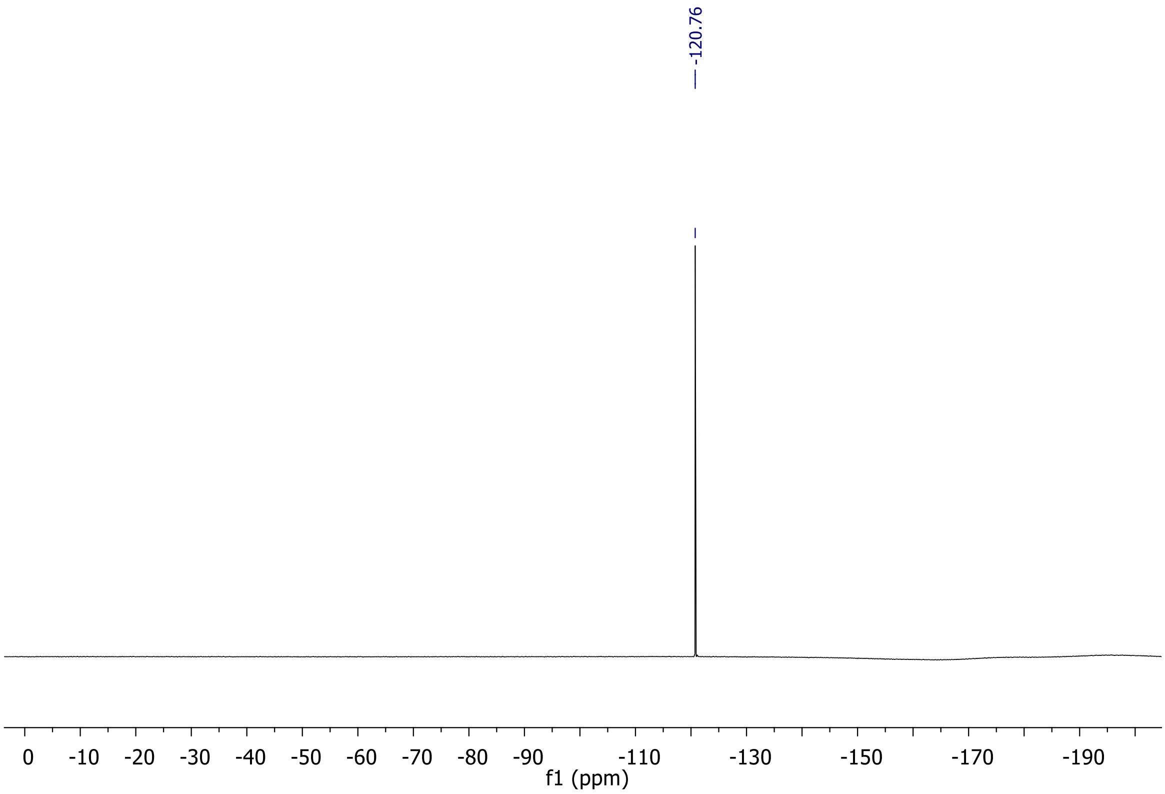

^{19}F NMR Spectrum of Compound 14

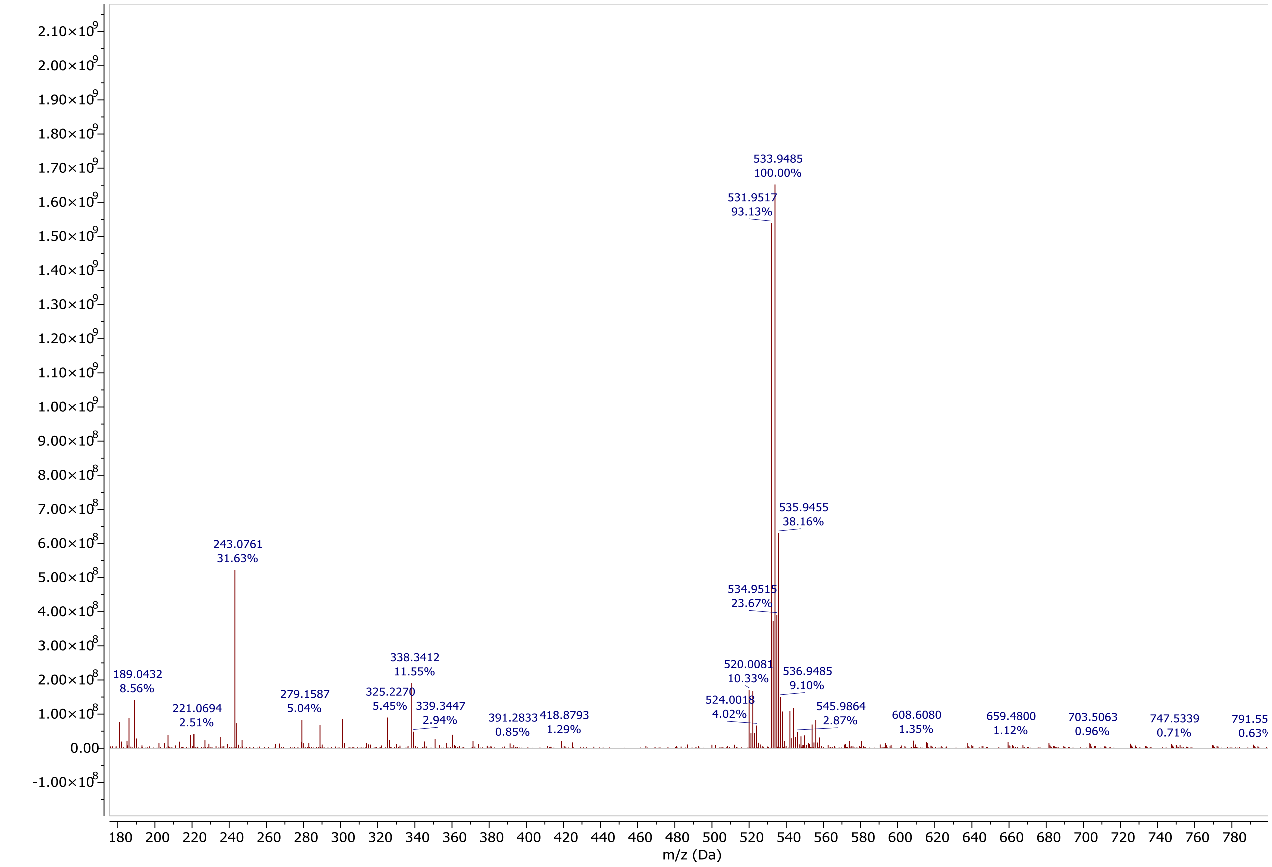

HRMS Spectrum of Compound 14

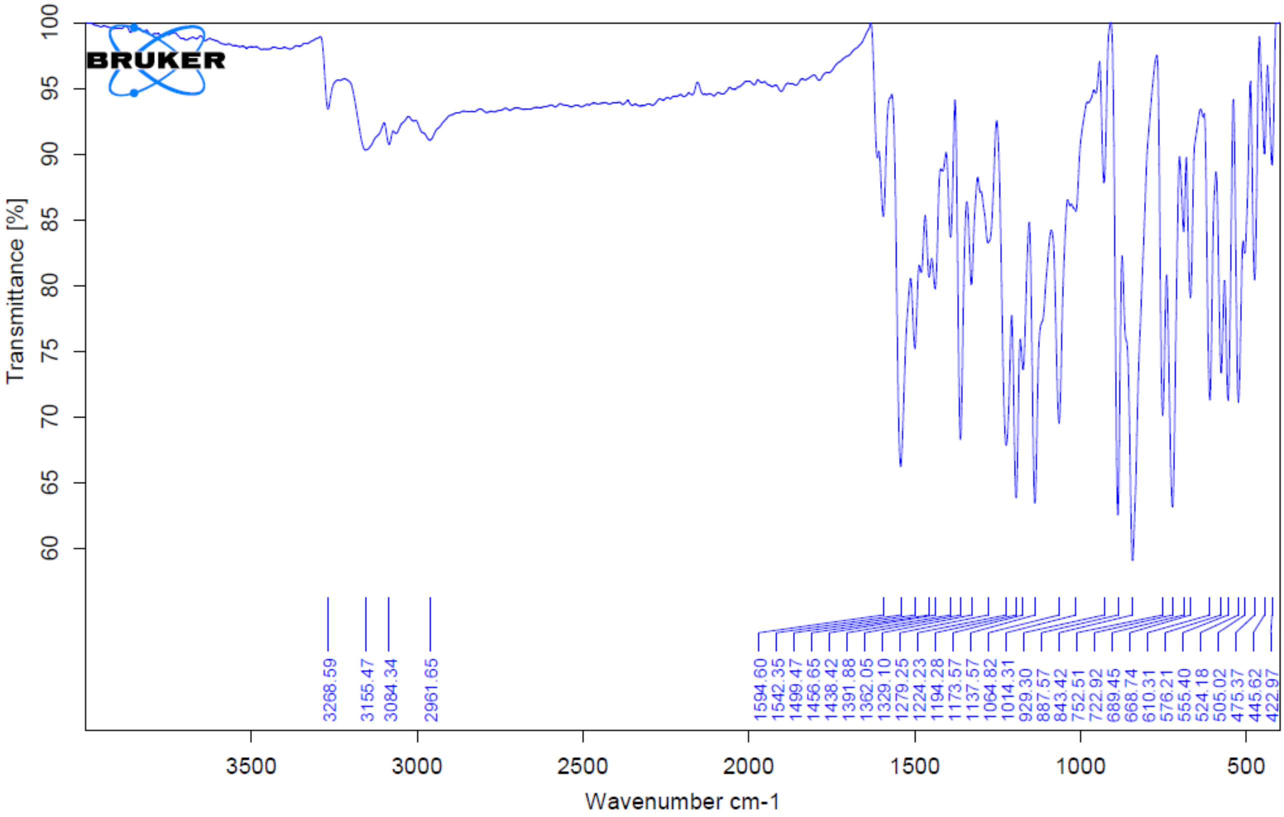

FTIR Spectrum of Compound 14

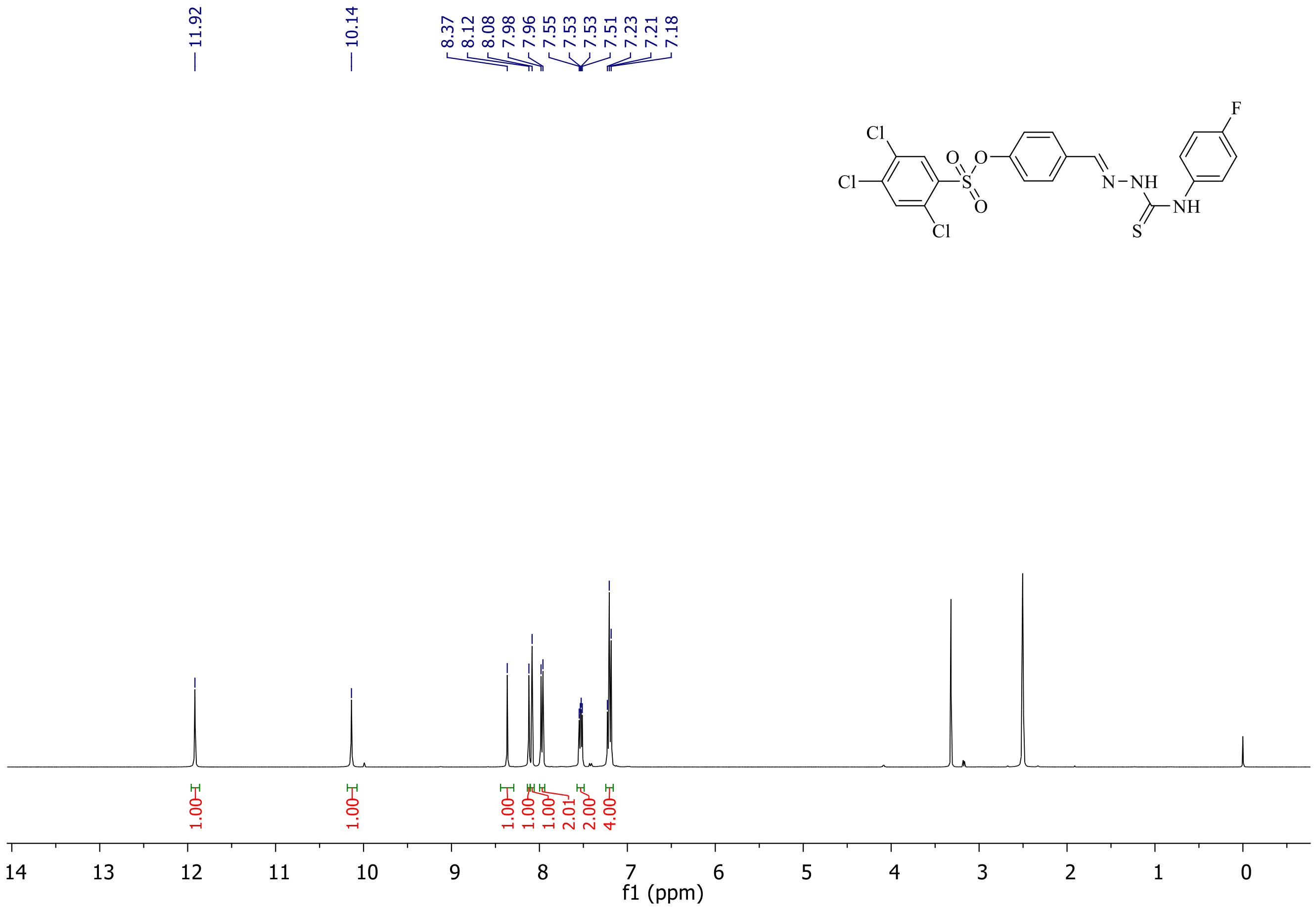

^{1}H NMR Spectrum of Compound 15

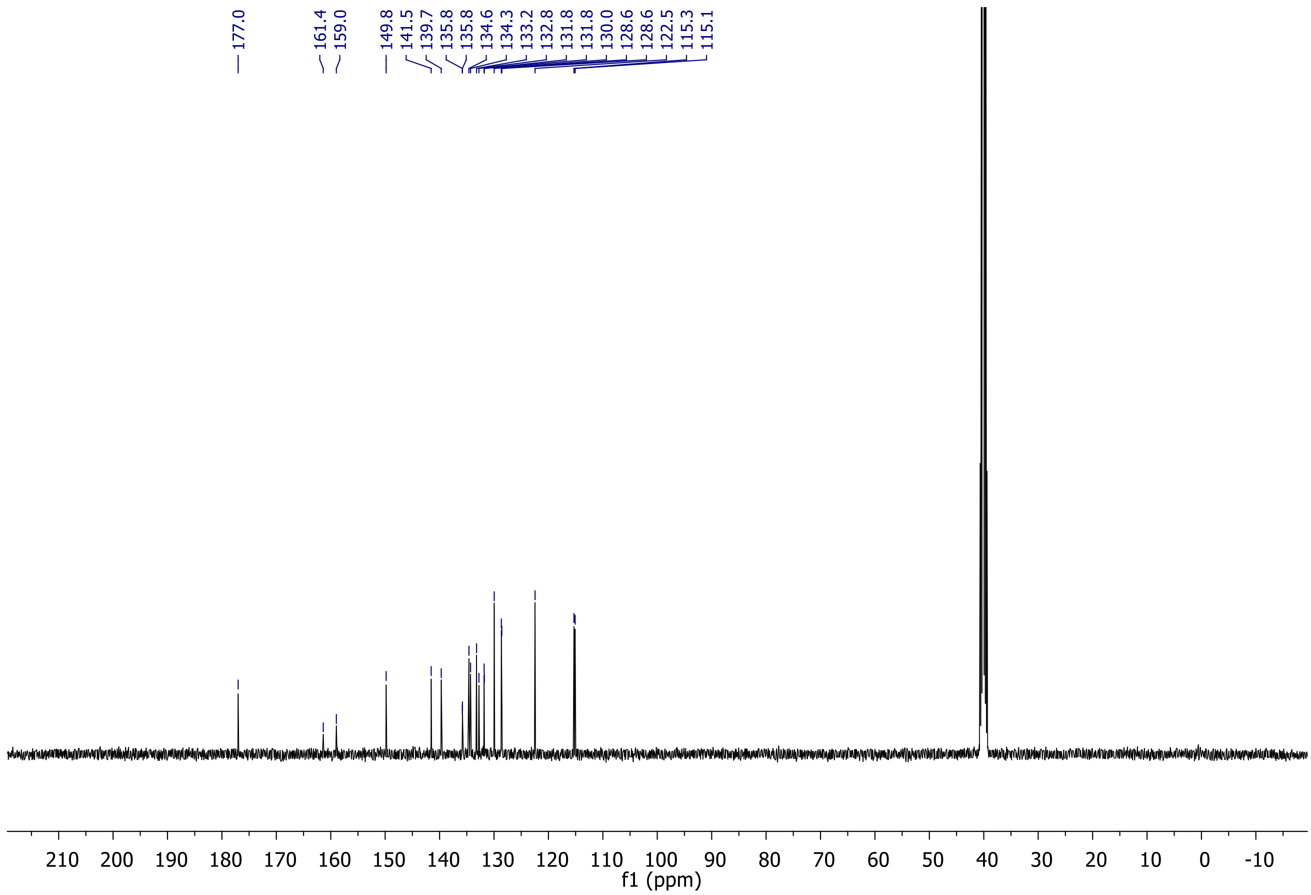

^{13}C NMR Spectrum of Compound 15

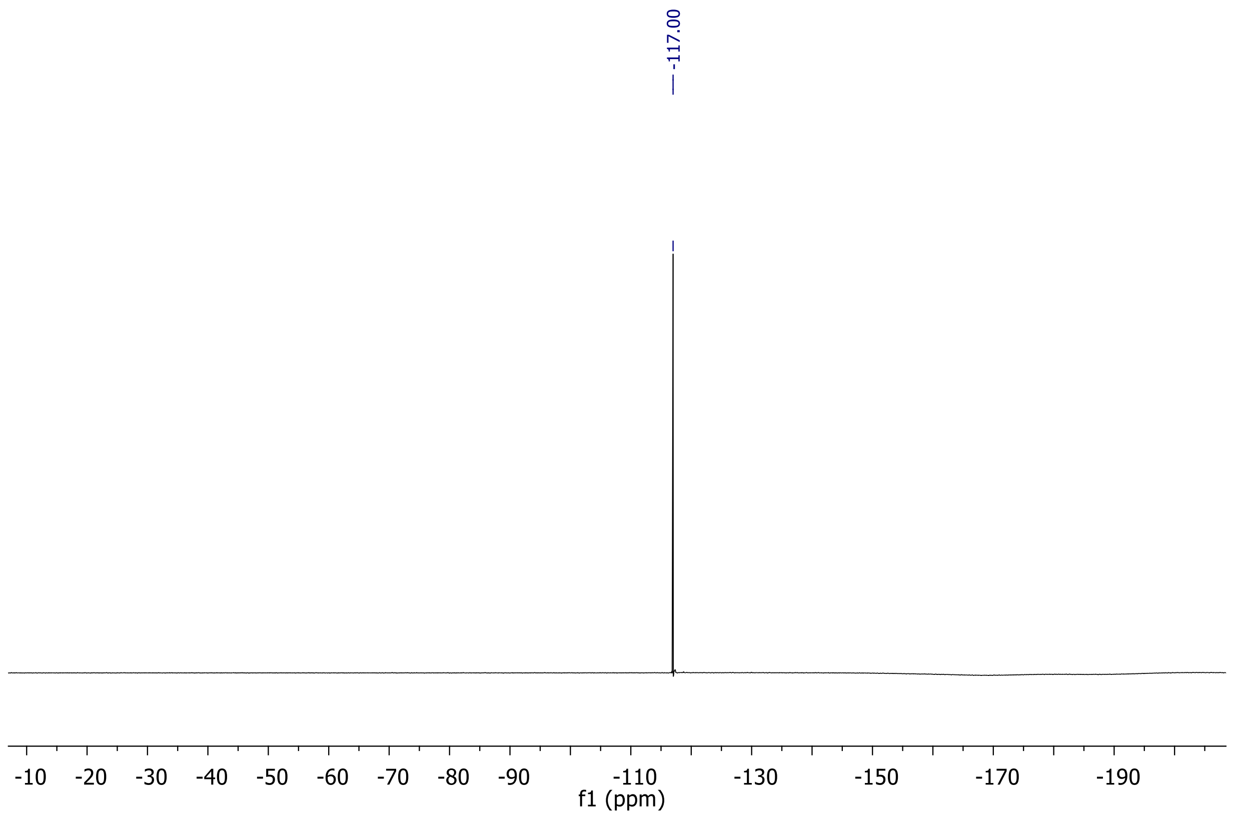

^{19}F NMR Spectrum of Compound 15

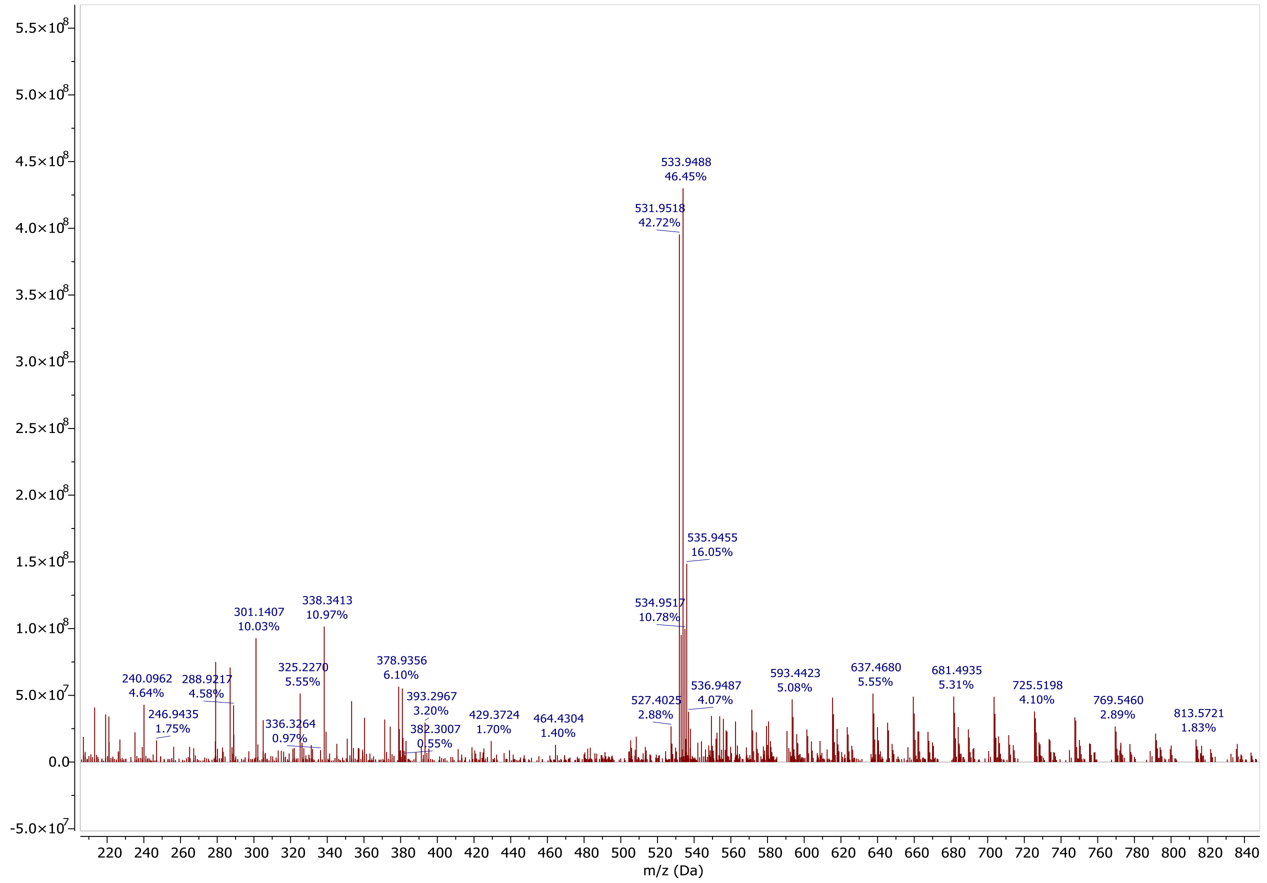

HRMS Spectrum of Compound 15

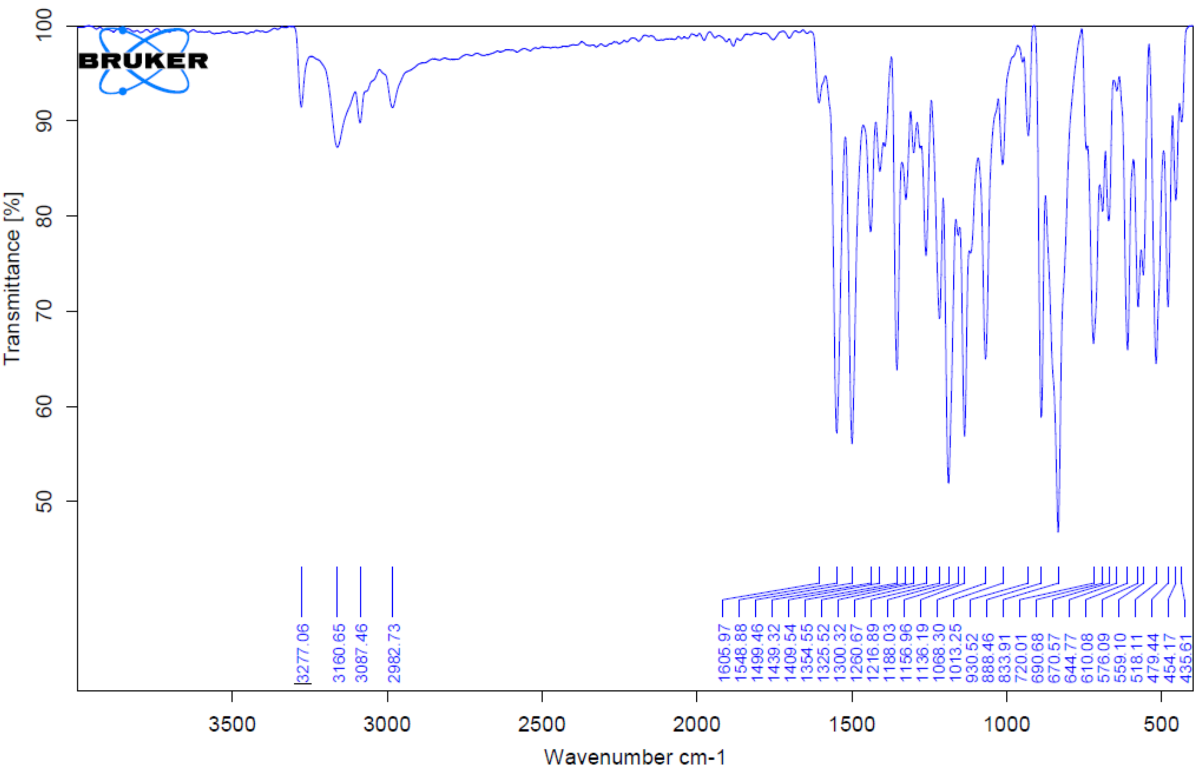

FTIR Spectrum of Compound 15

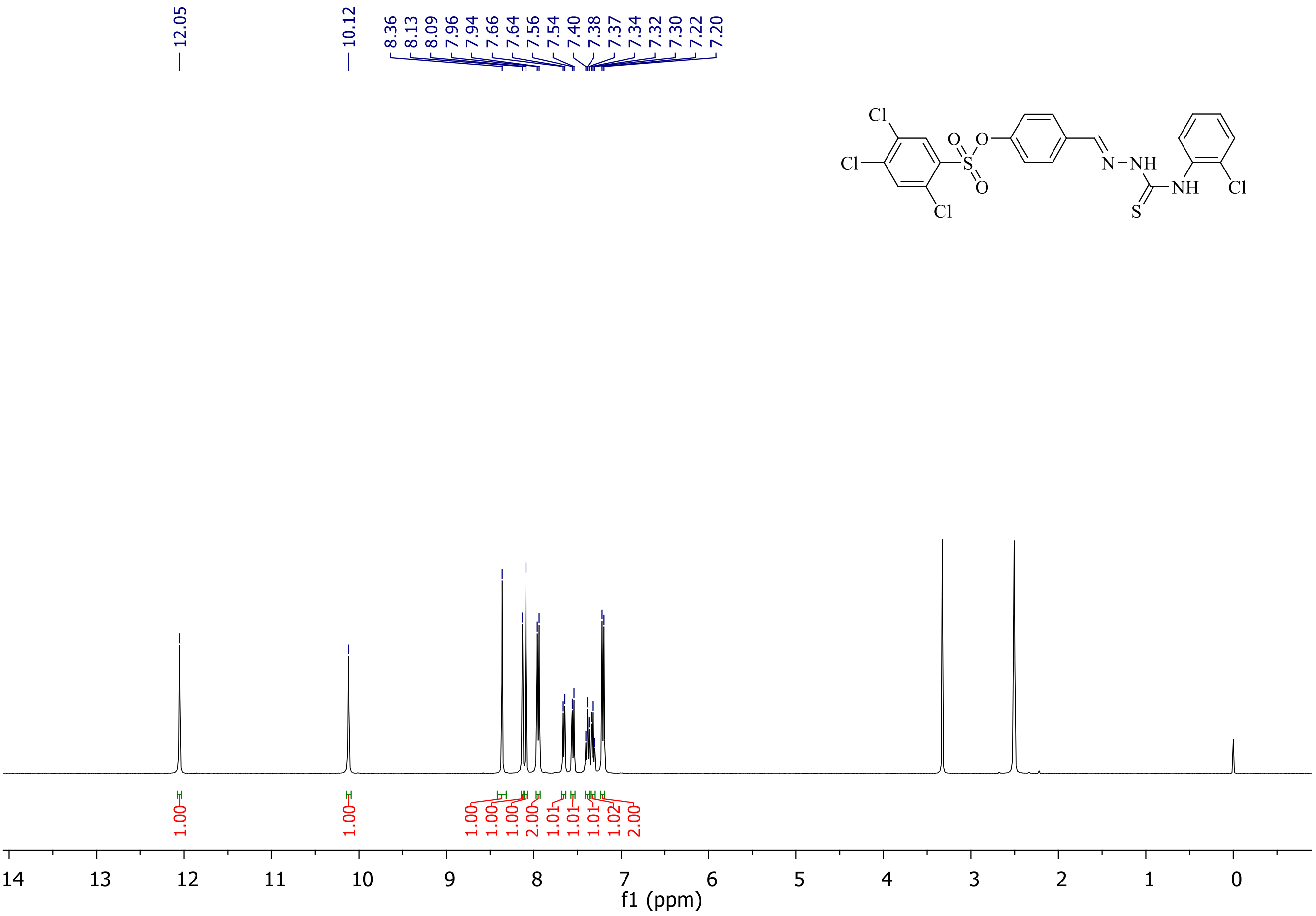

^{1}H NMR Spectrum of Compound 16

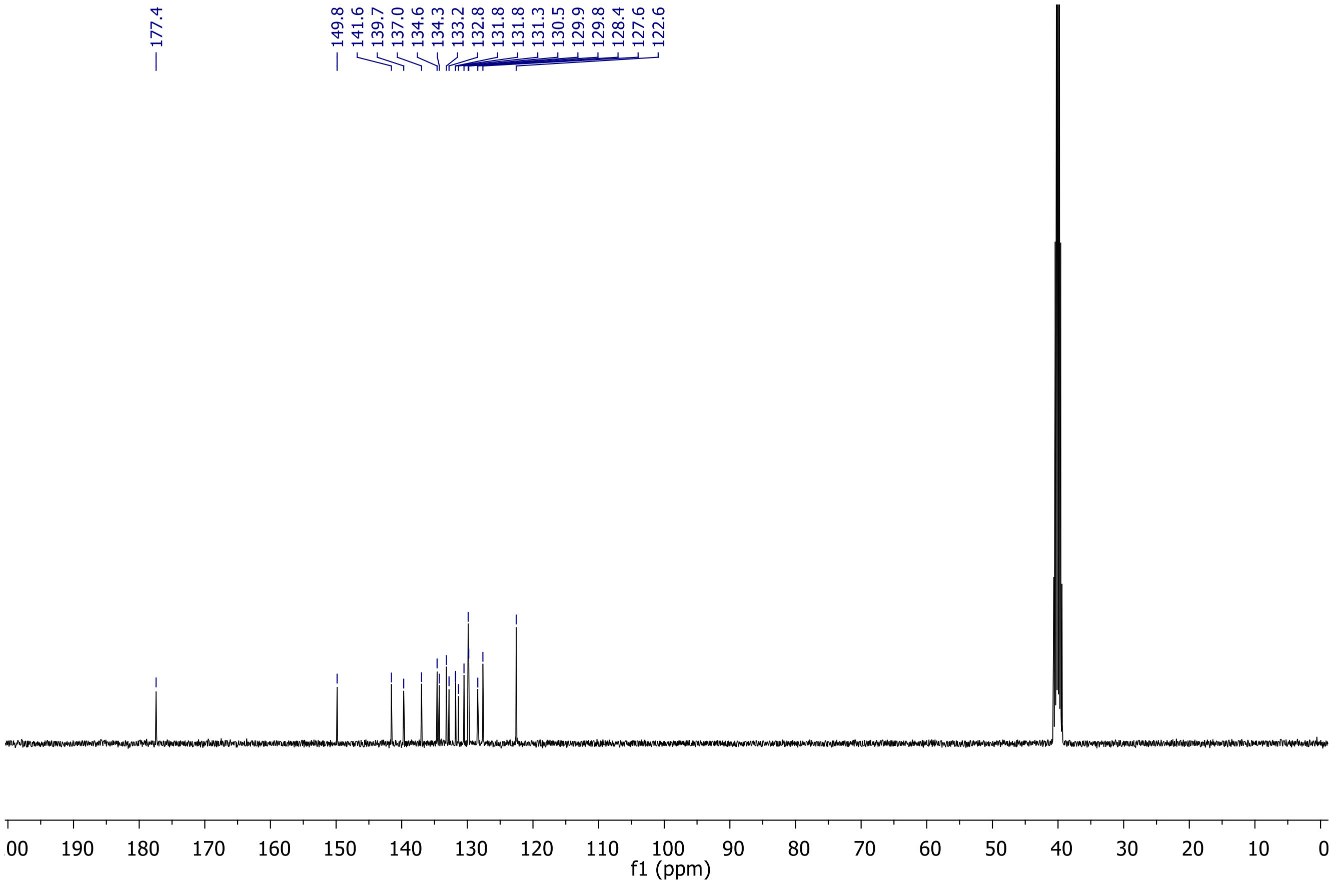

^{13}C NMR Spectrum of Compound 16

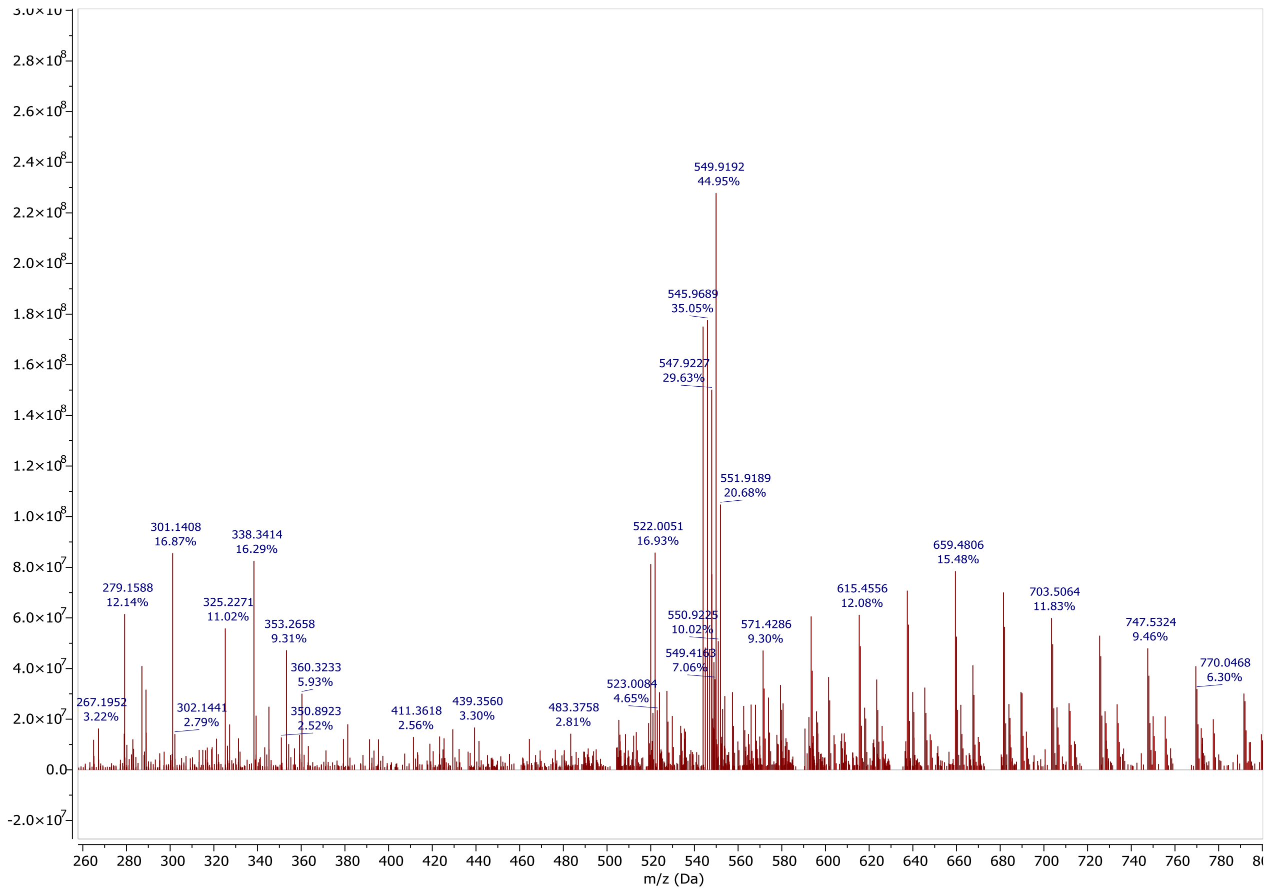

HRMS Spectrum of Compound 16

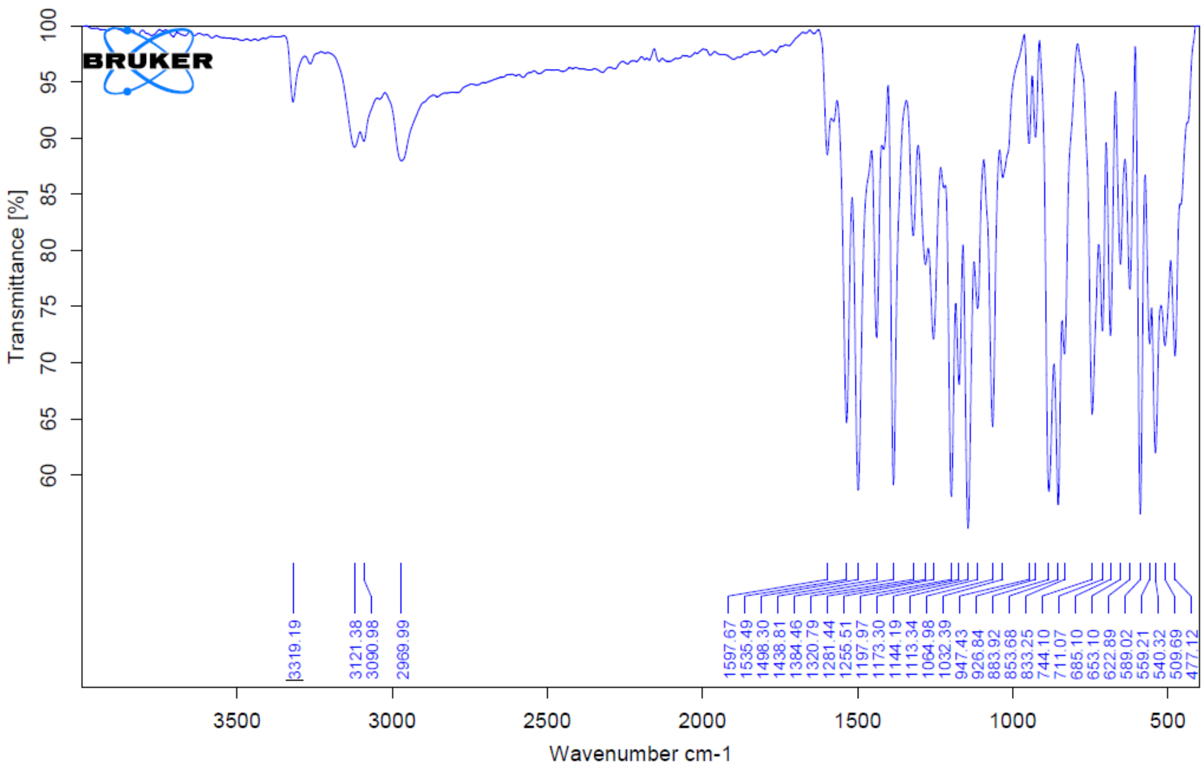

FTIR Spectrum of Compound 16

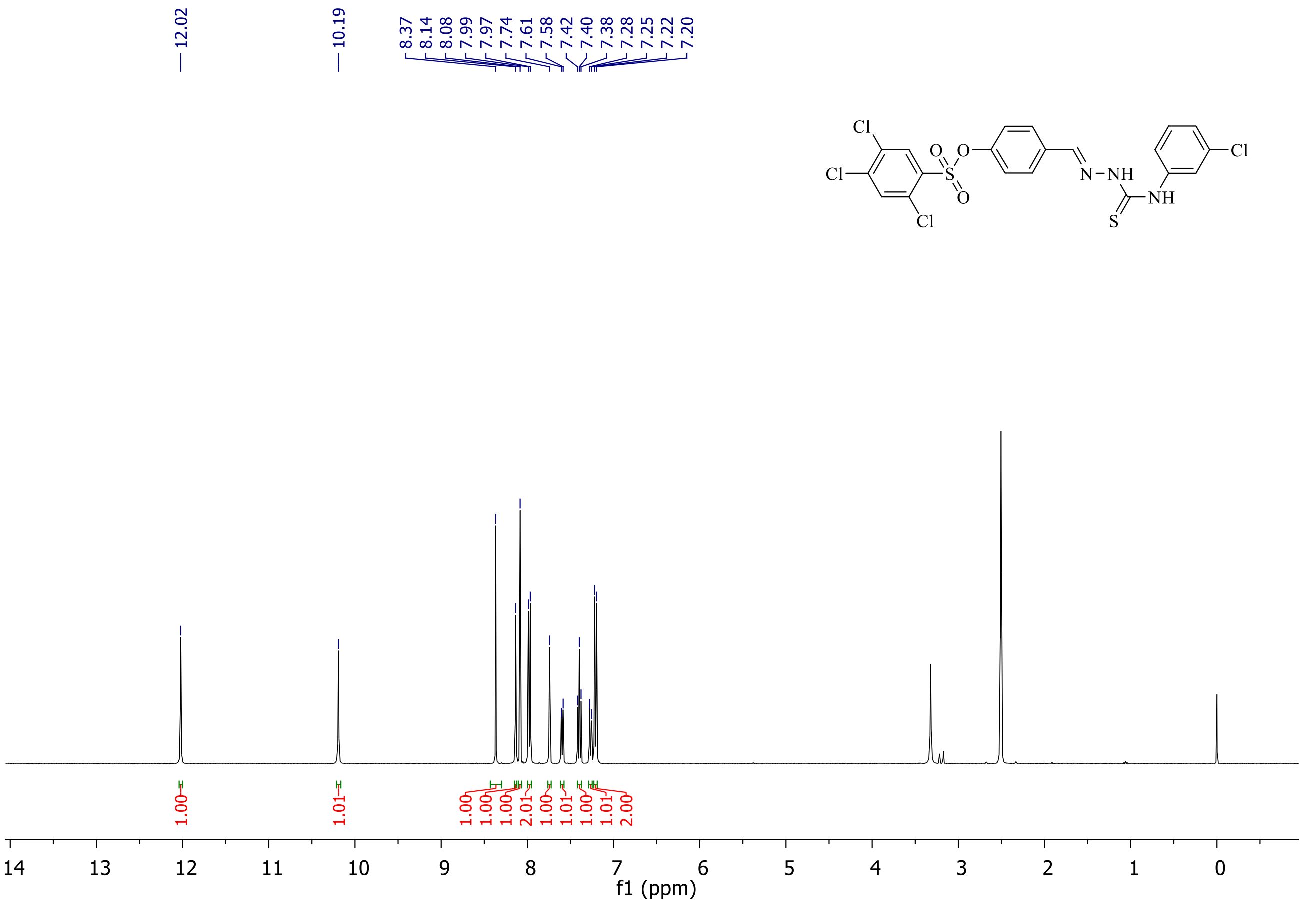

^{1}H NMR Spectrum of Compound 17

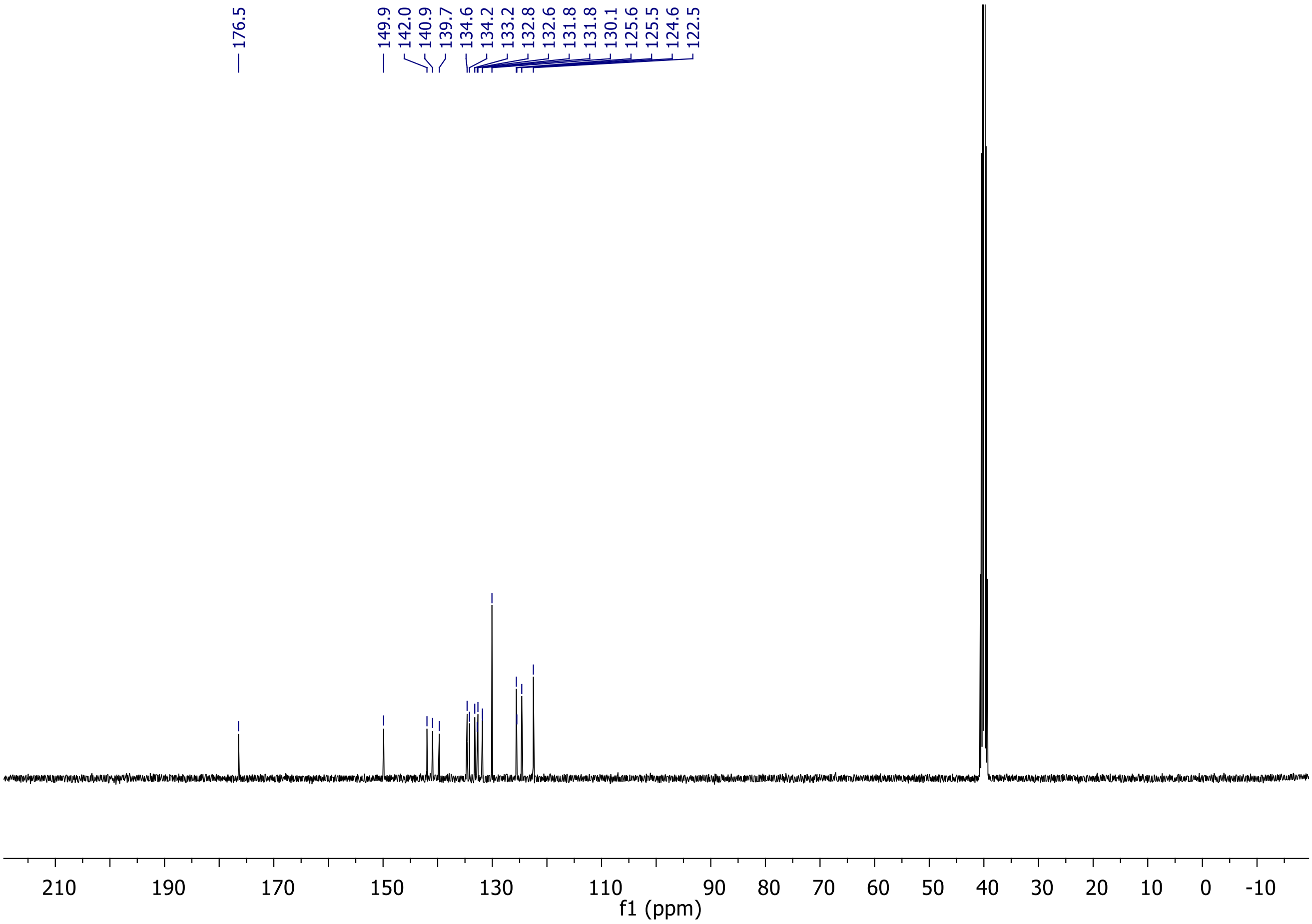

^{13}C NMR Spectrum of Compound 17

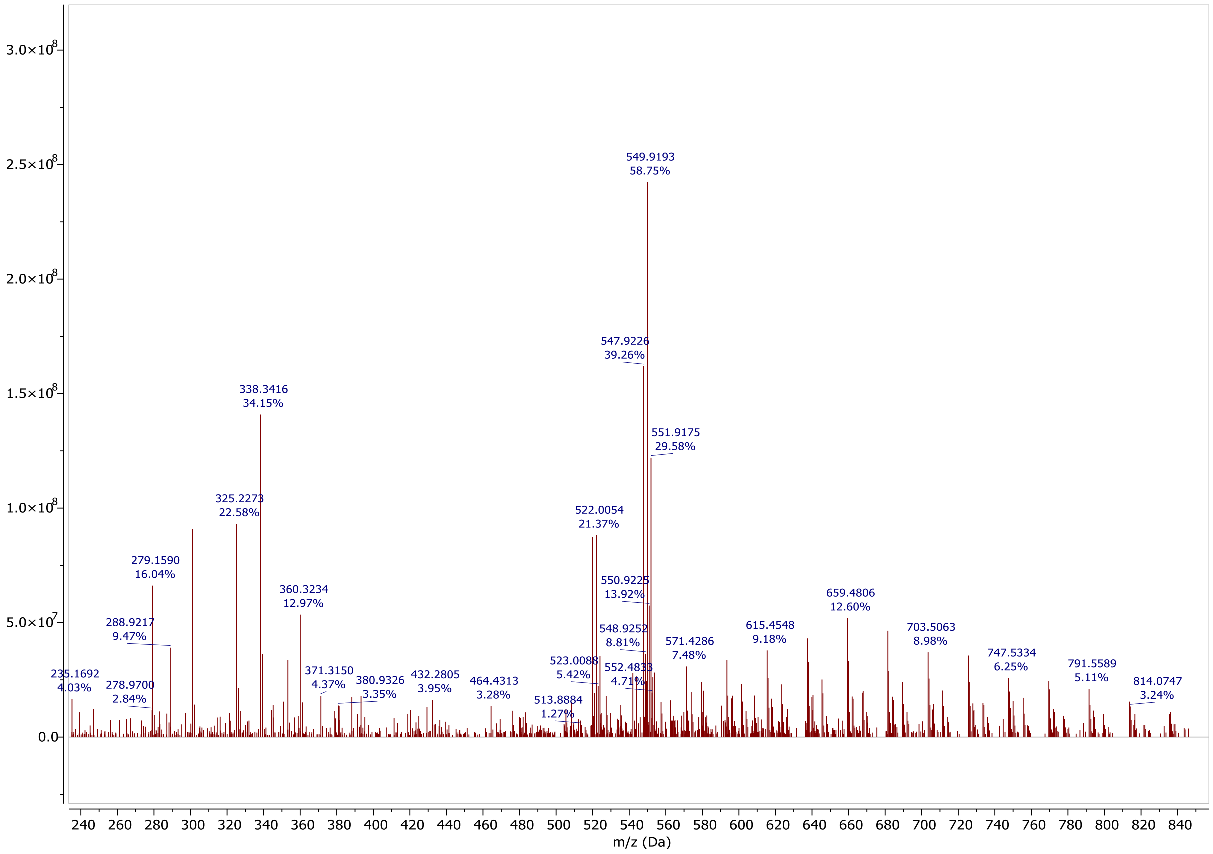

HRMS Spectrum of Compound 17

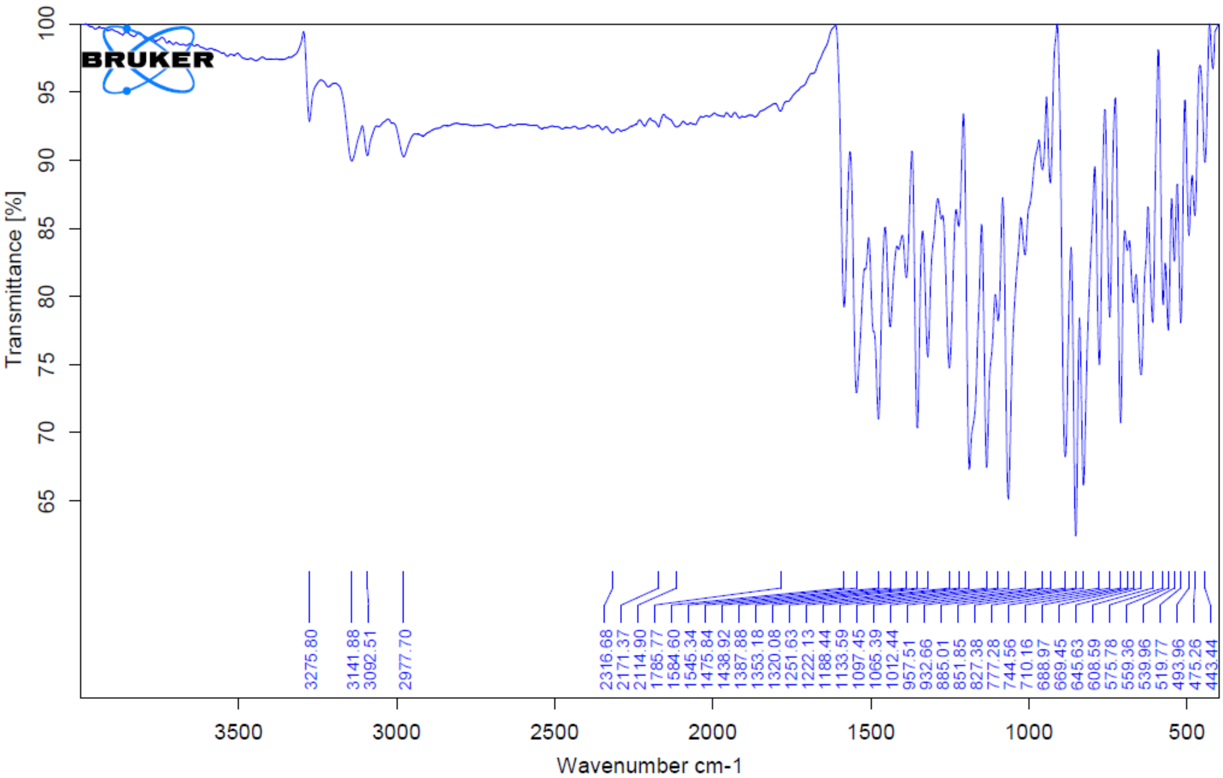

FTIR Spectrum of Compound 17

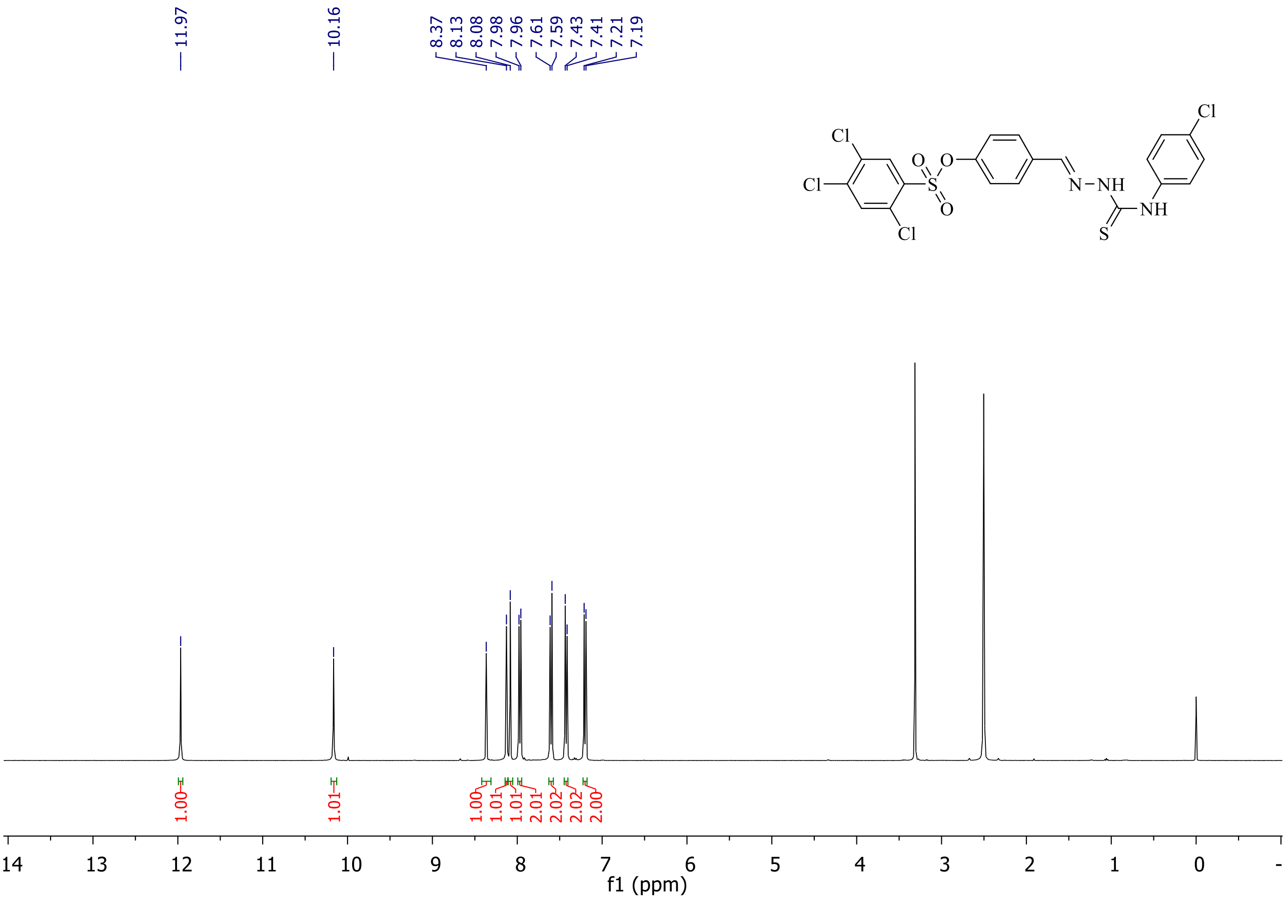

^{1}H NMR Spectrum of Compound 18

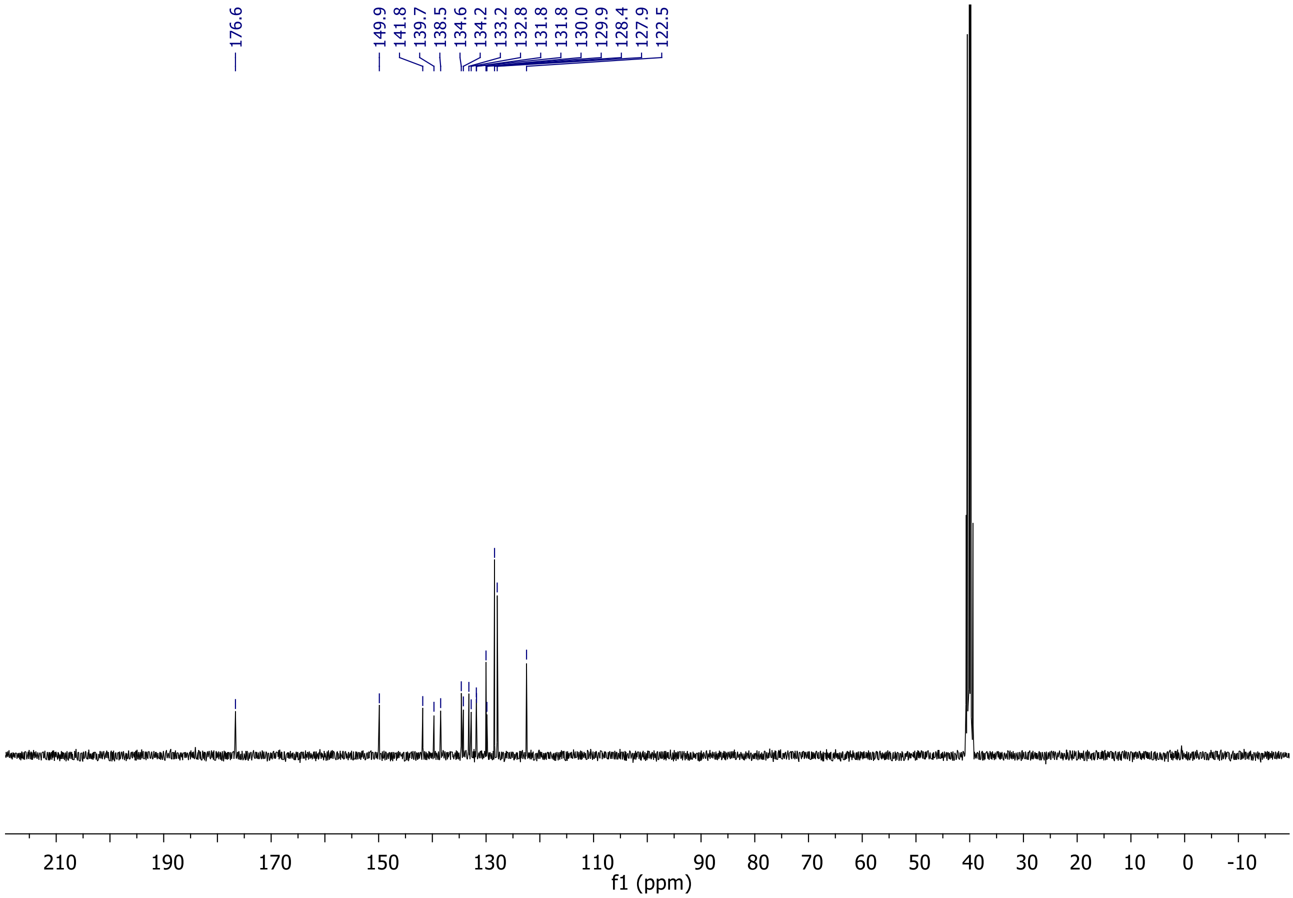

^{13}C NMR Spectrum of Compound 18

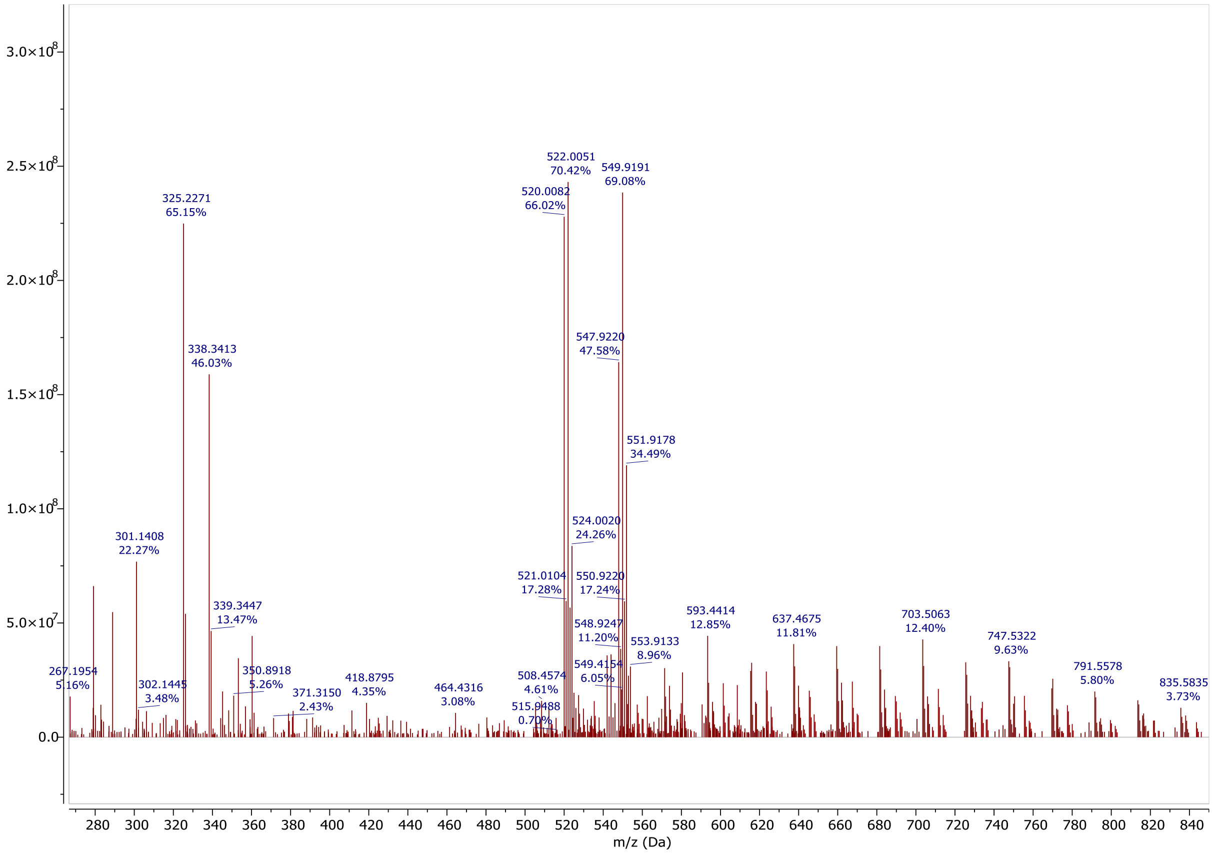

HRMS Spectrum of Compound 18

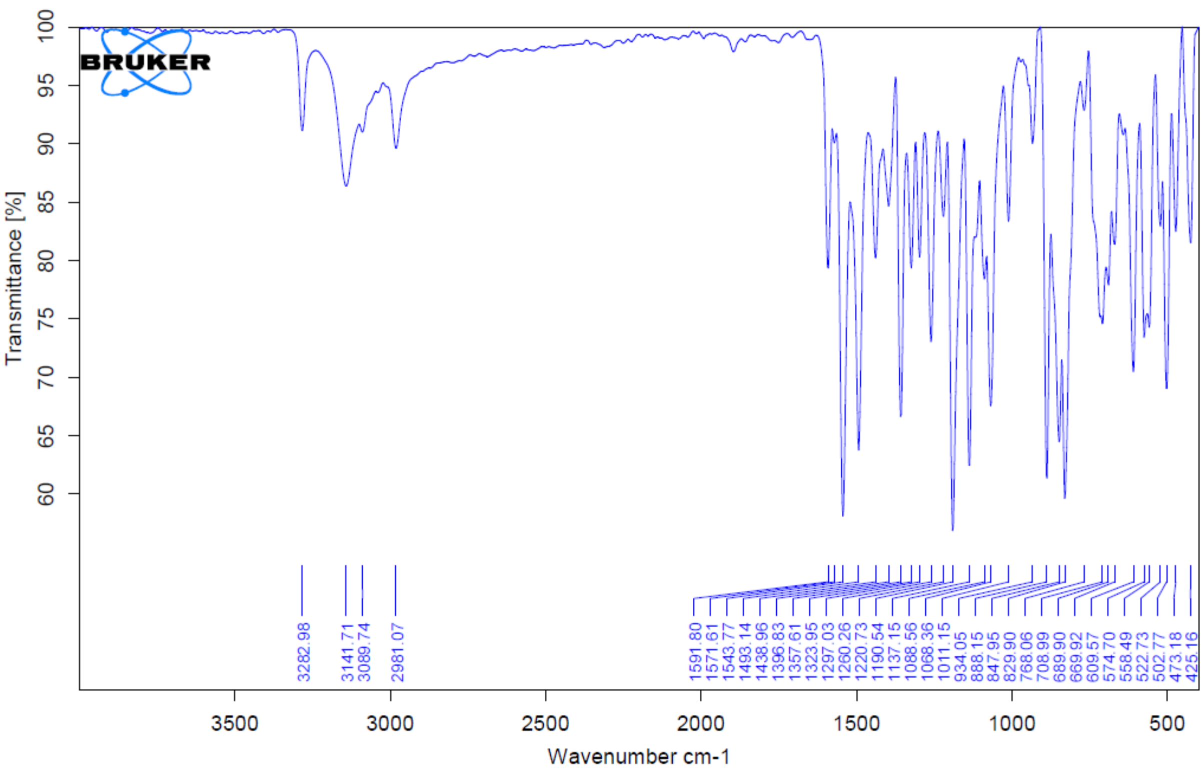

FTIR Spectrum of Compound 18

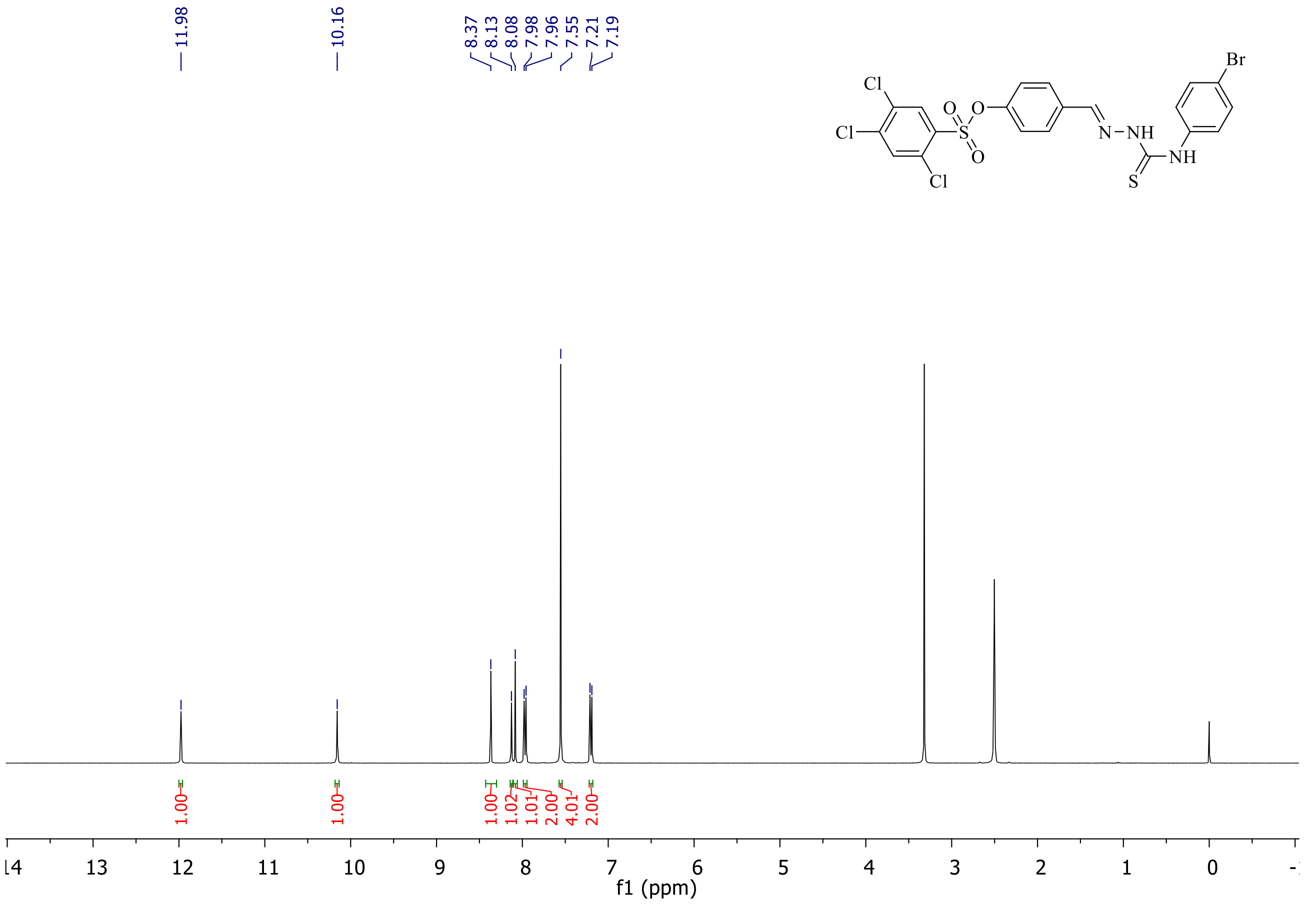

^{1}H NMR Spectrum of Compound 19

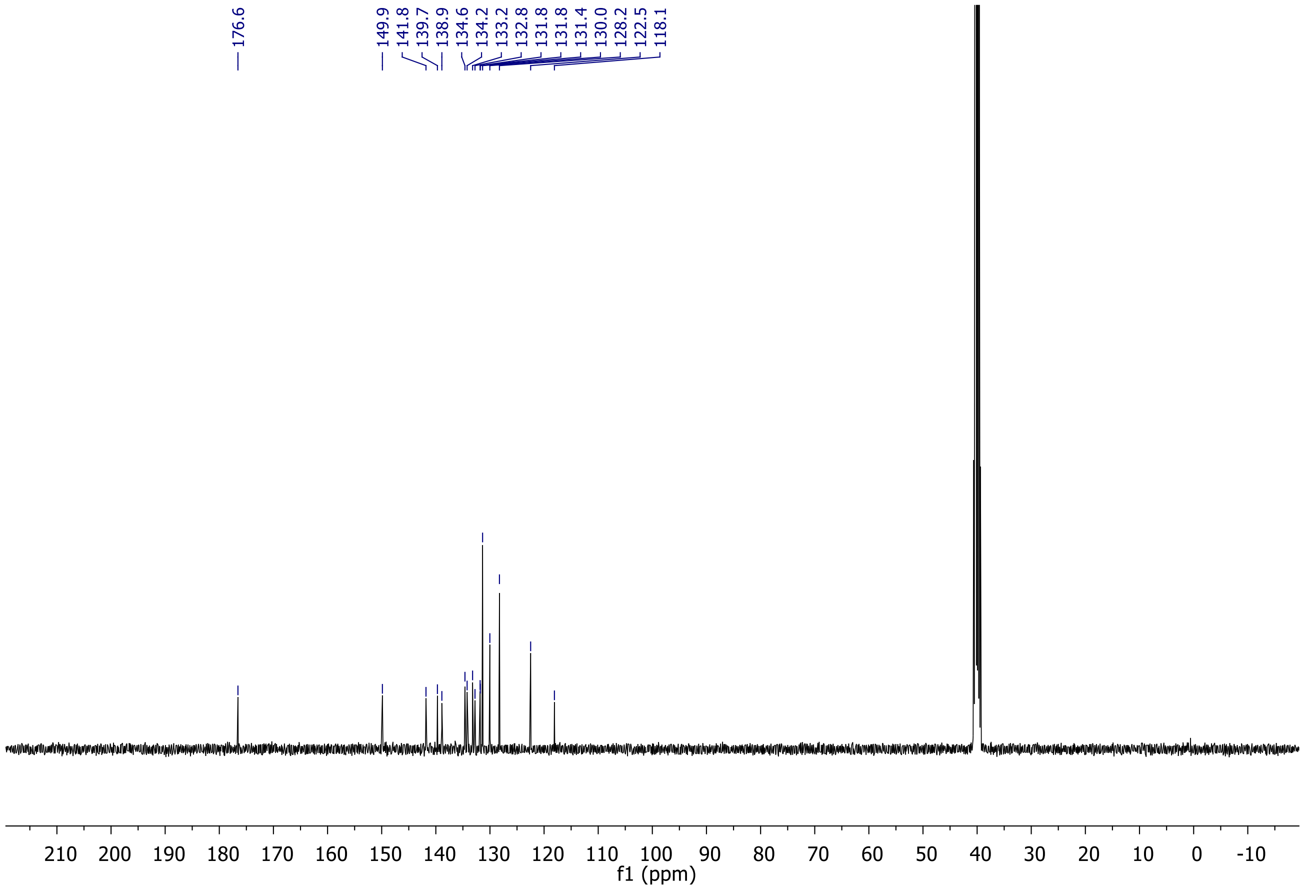

^{13}C NMR Spectrum of Compound 19

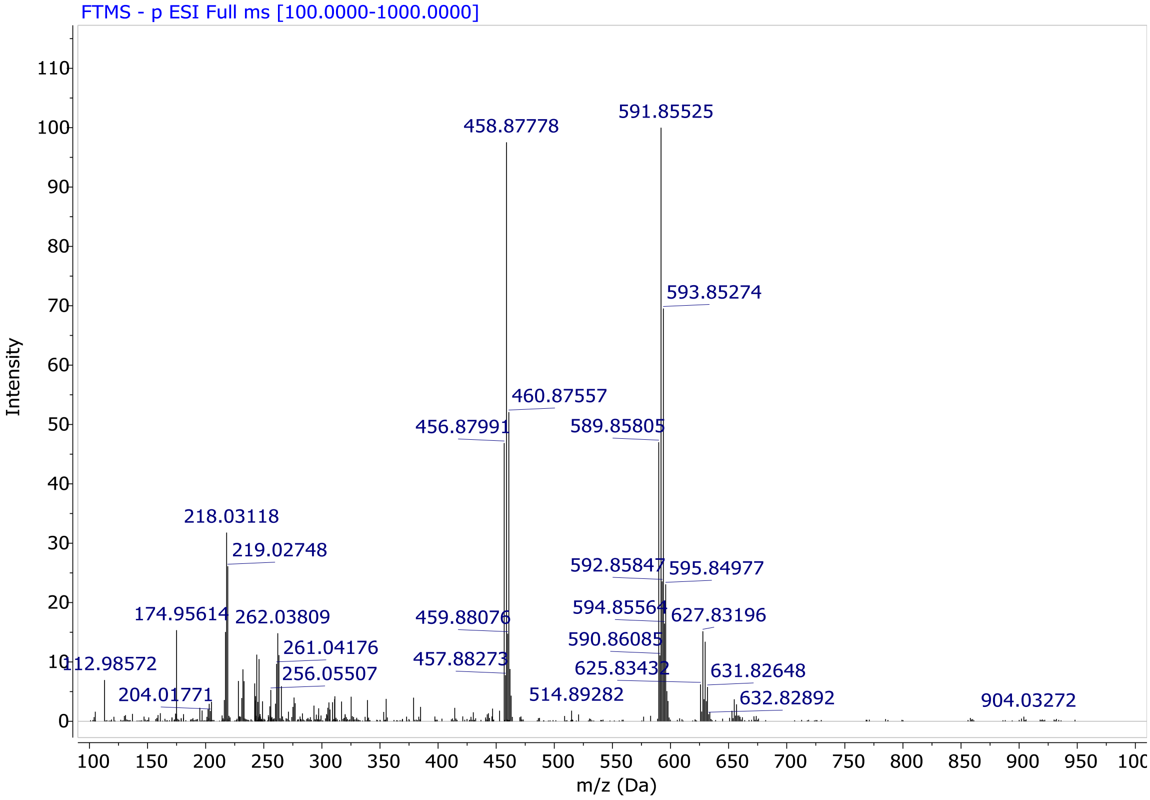

HRMS Spectrum of Compound 19

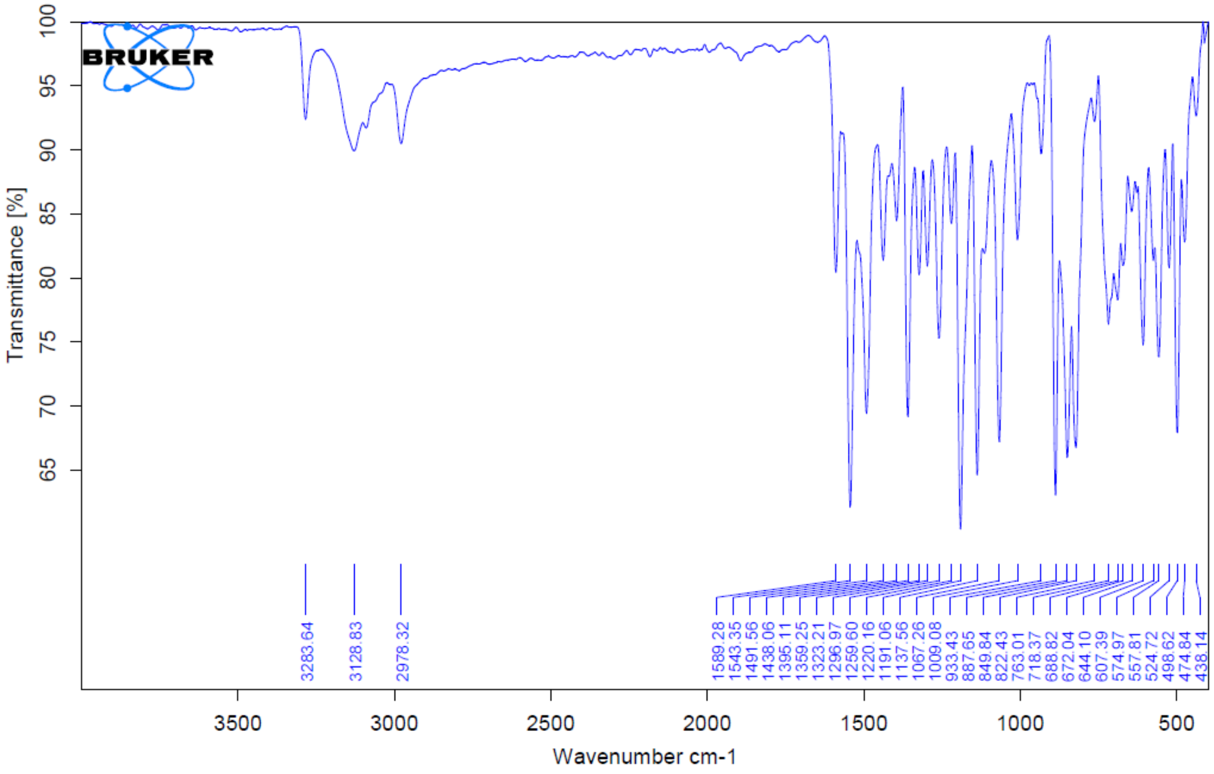

FTIR Spectrum of Compound 19

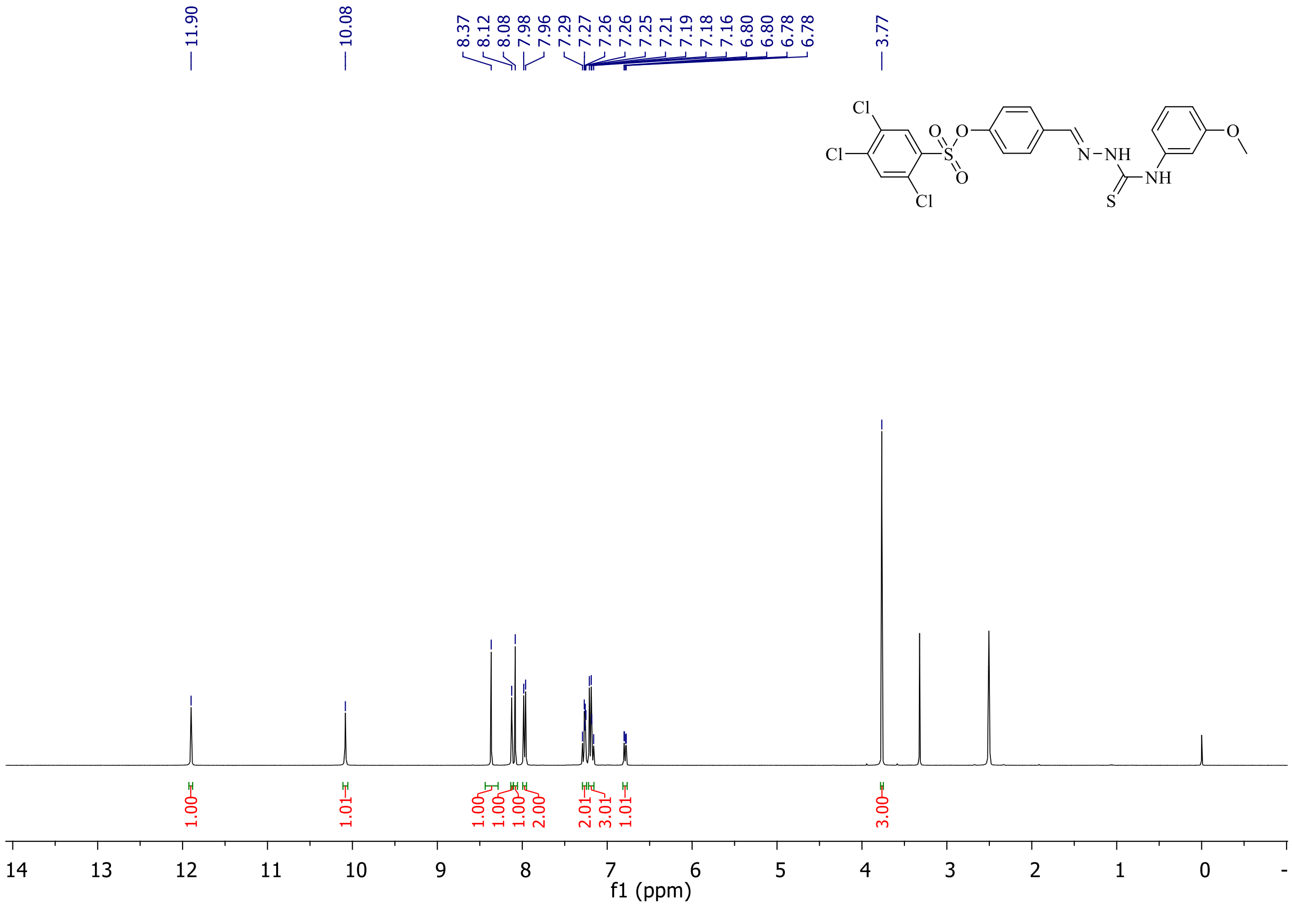

^{1}H NMR Spectrum of Compound 20

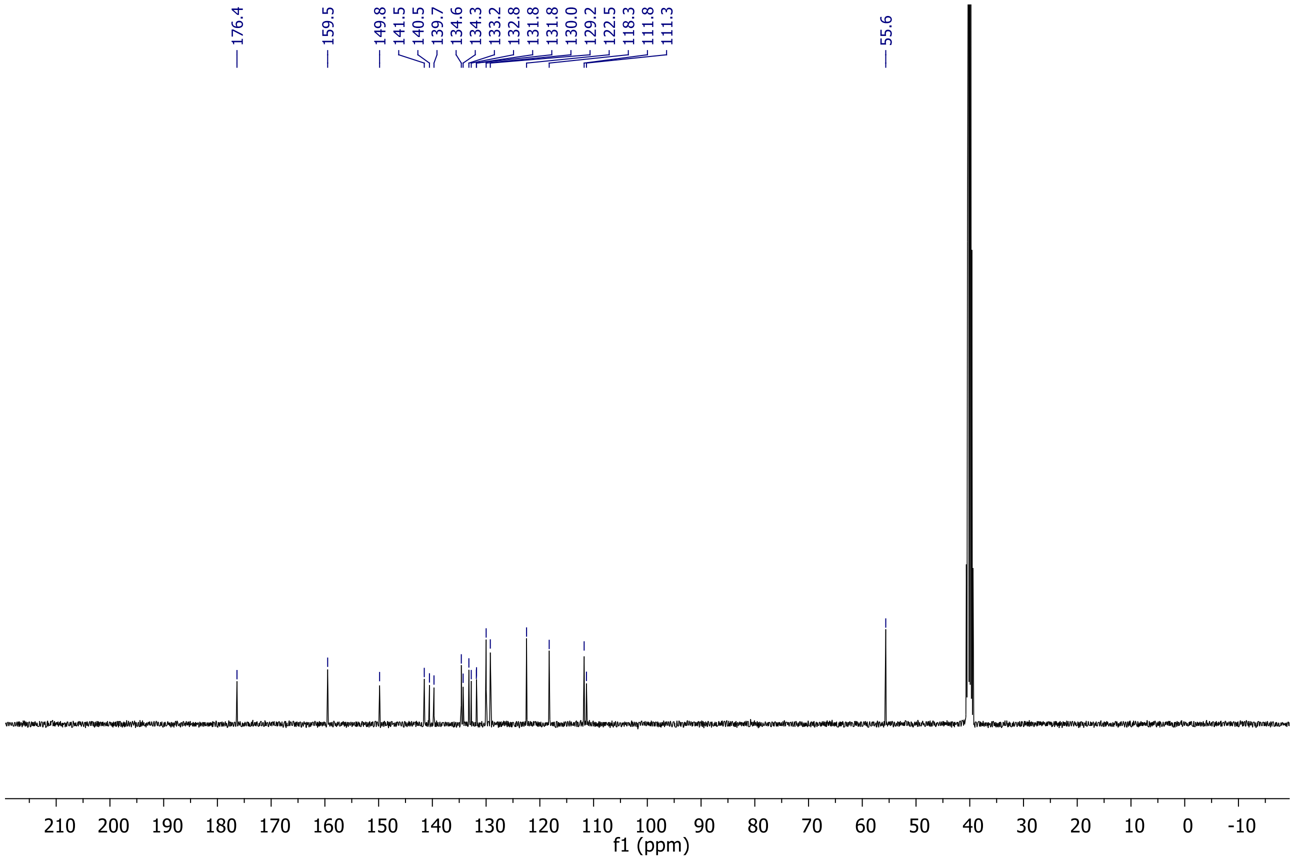

^{13}C NMR Spectrum of Compound 20

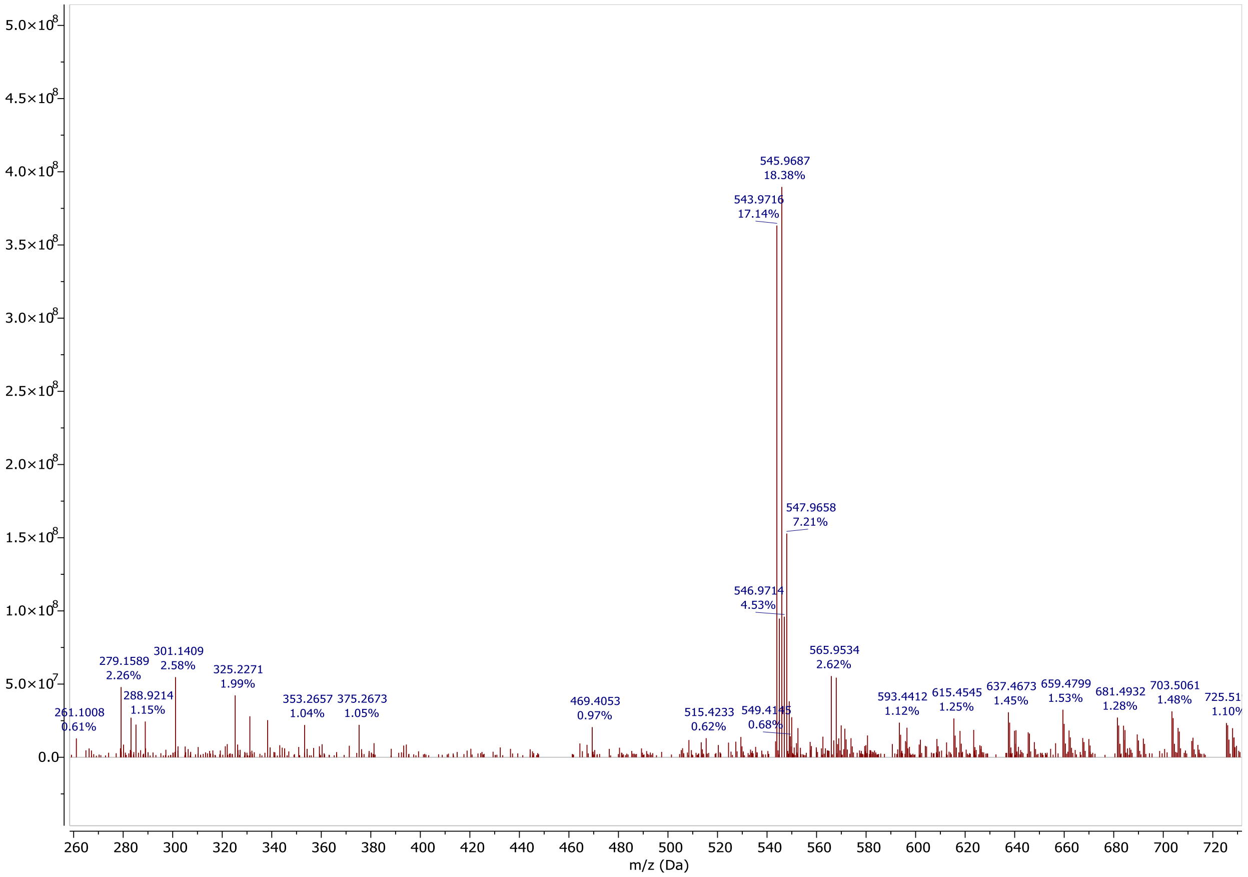

HRMS Spectrum of Compound 20

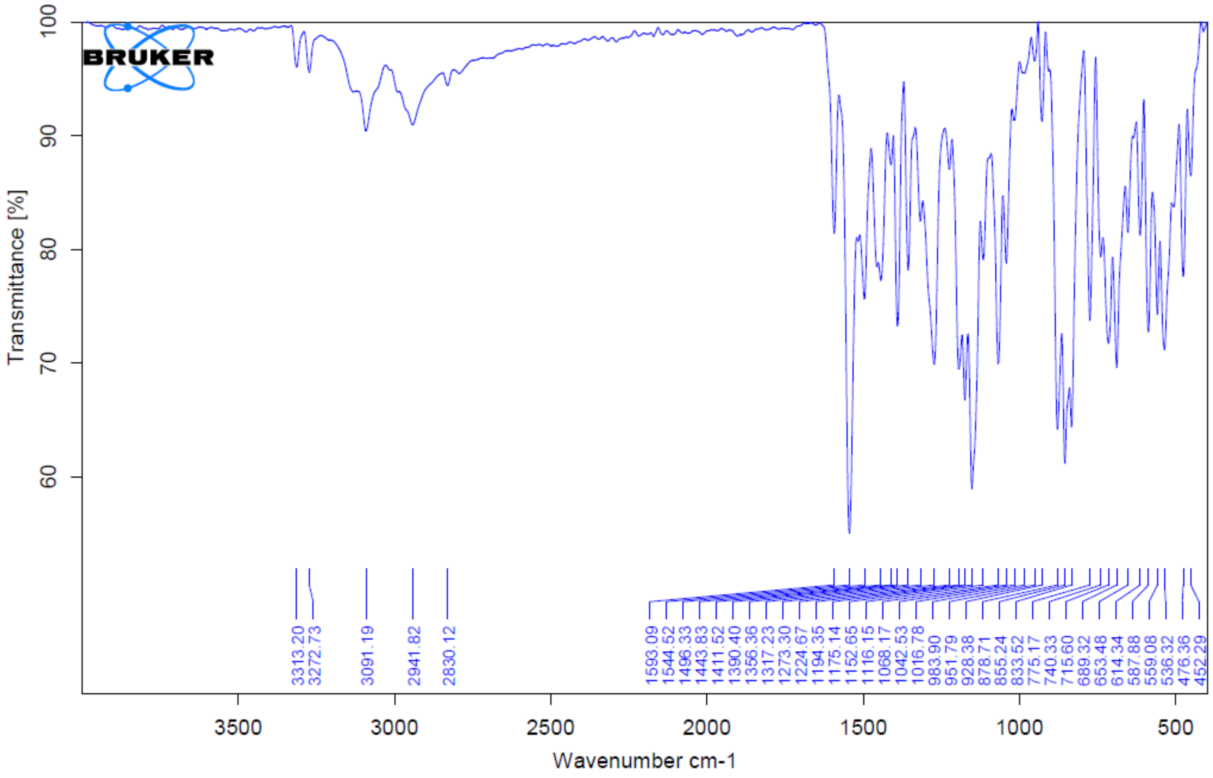

FTIR Spectrum of Compound 20

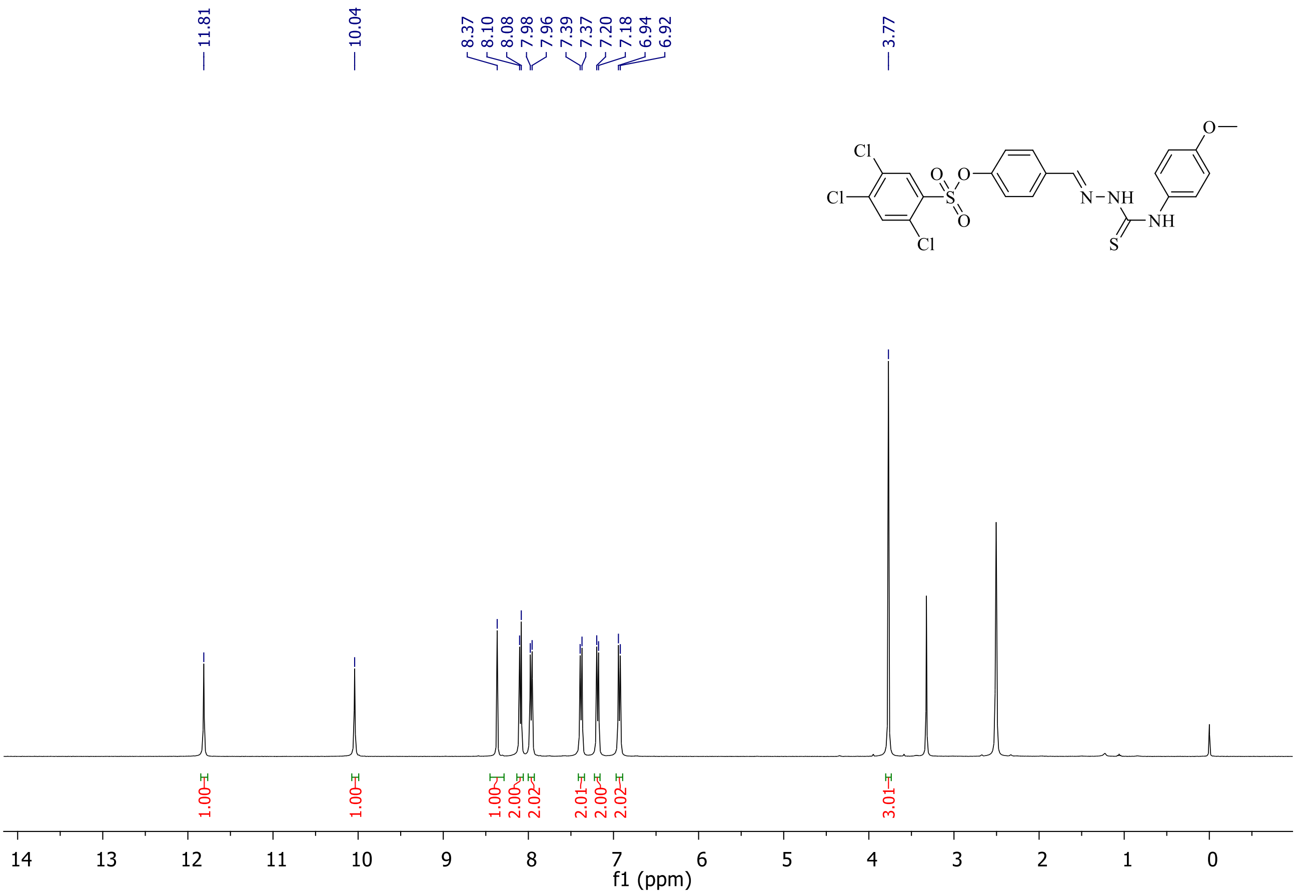

^{1}H NMR Spectrum of Compound 21

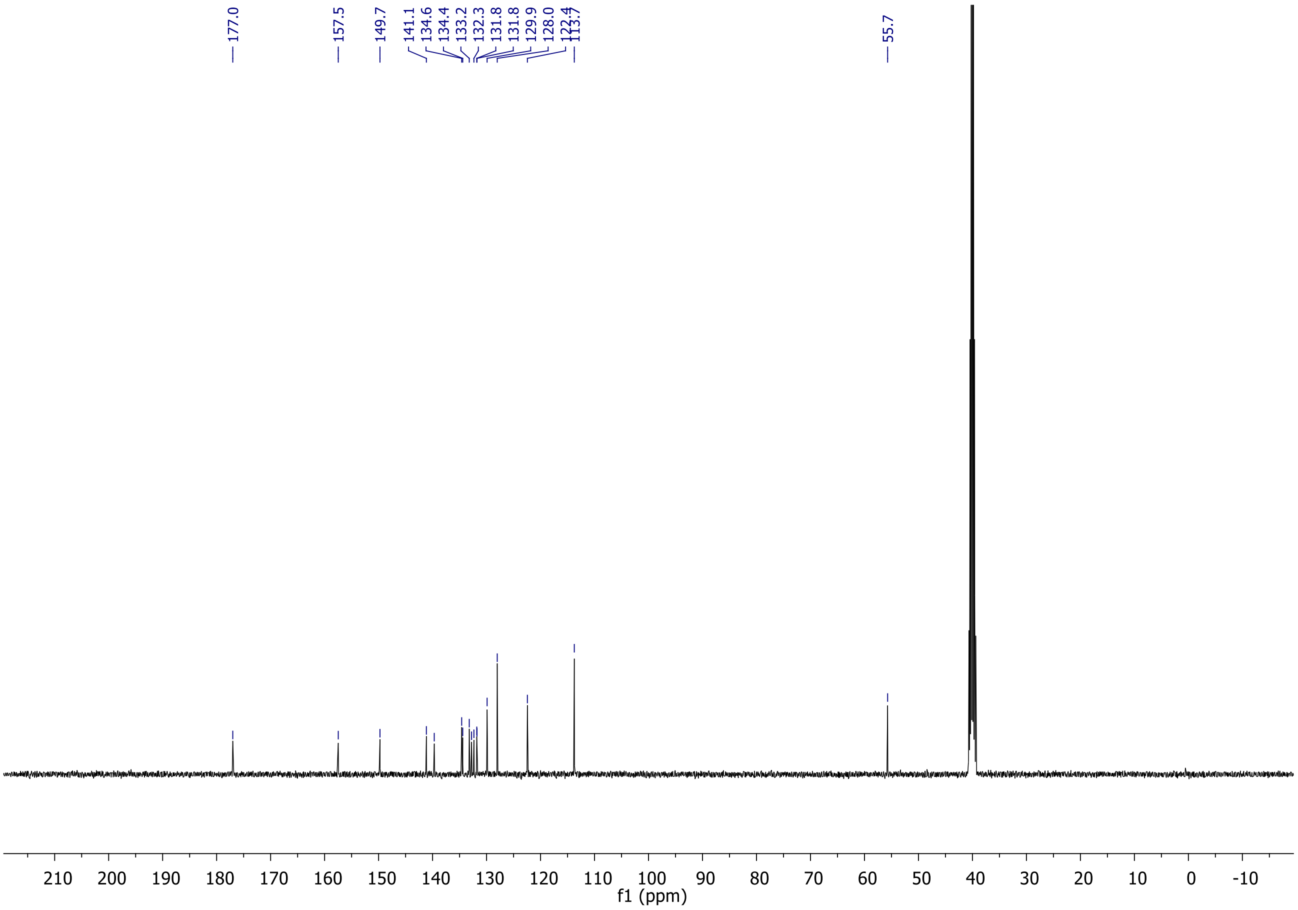

^{13}C NMR Spectrum of Compound 21

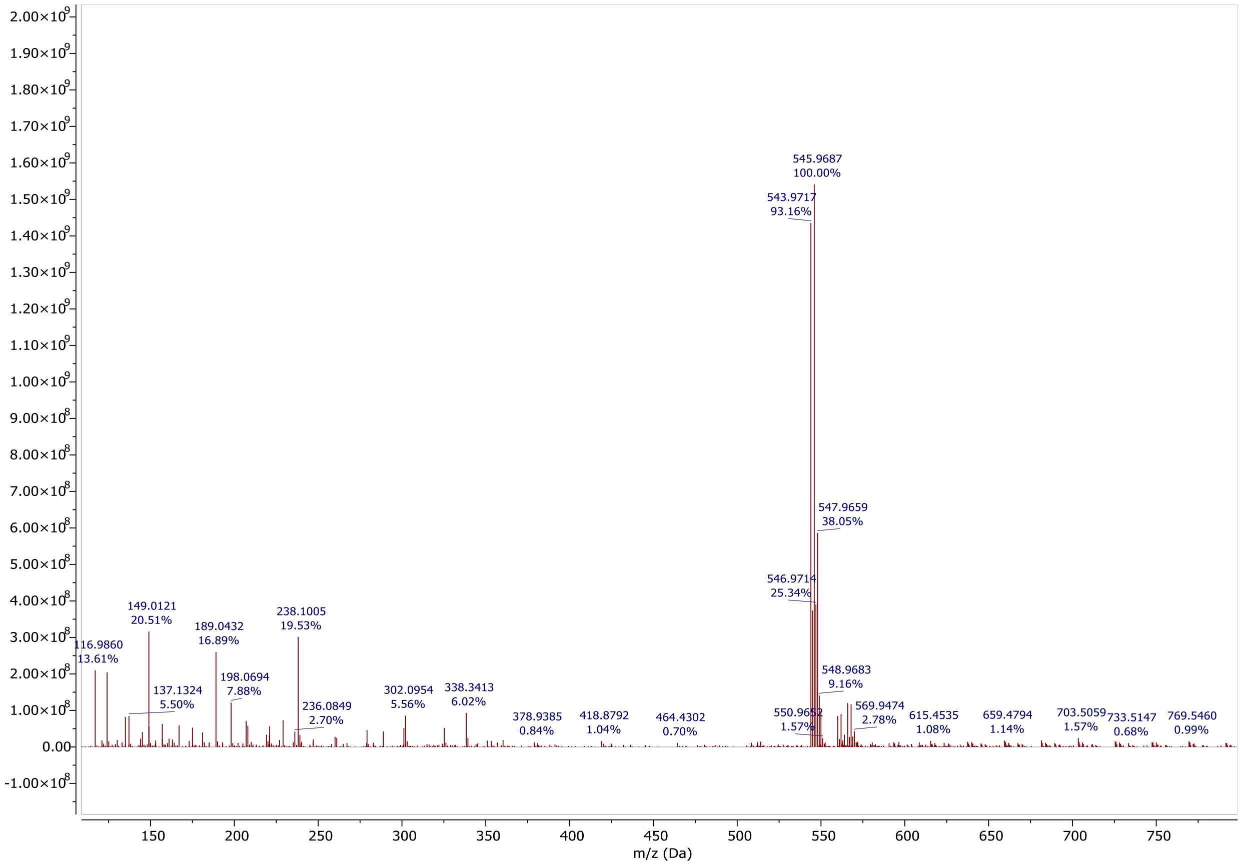

HRMS Spectrum of Compound 21

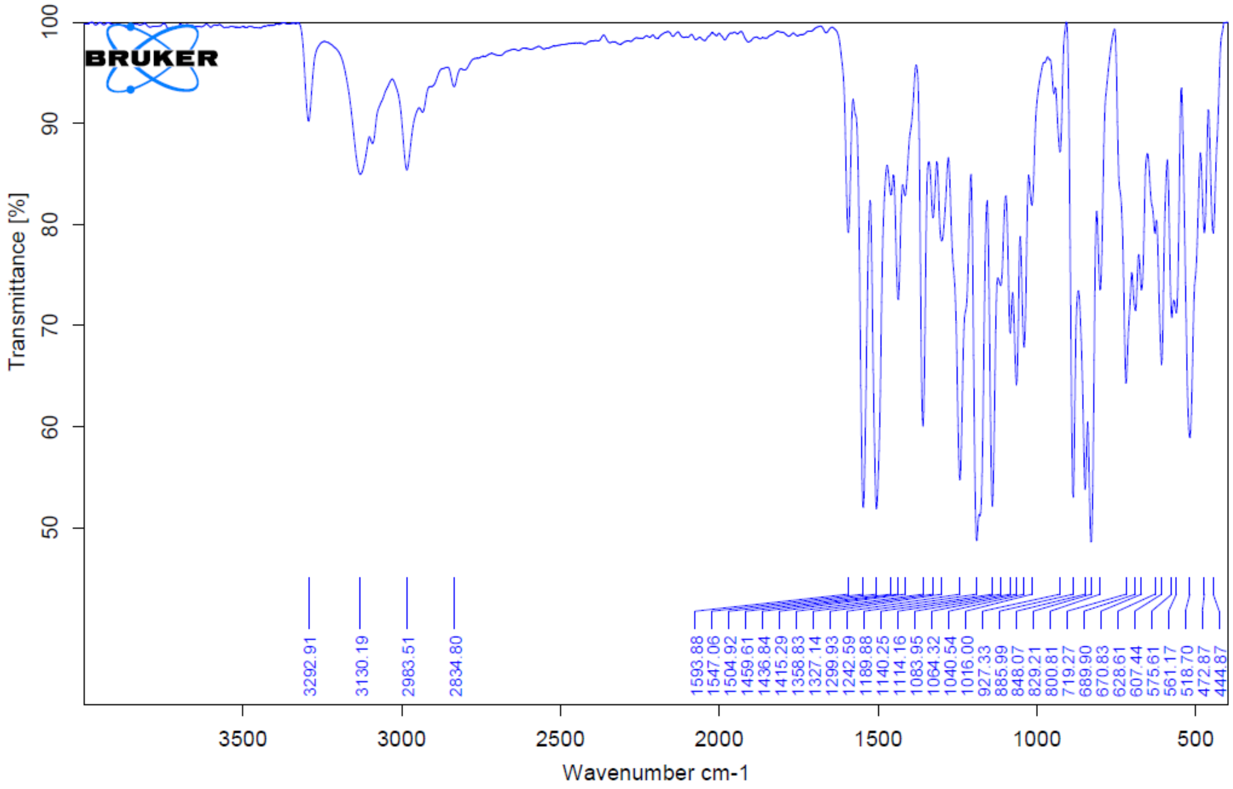

FTIR Spectrum of Compound 21

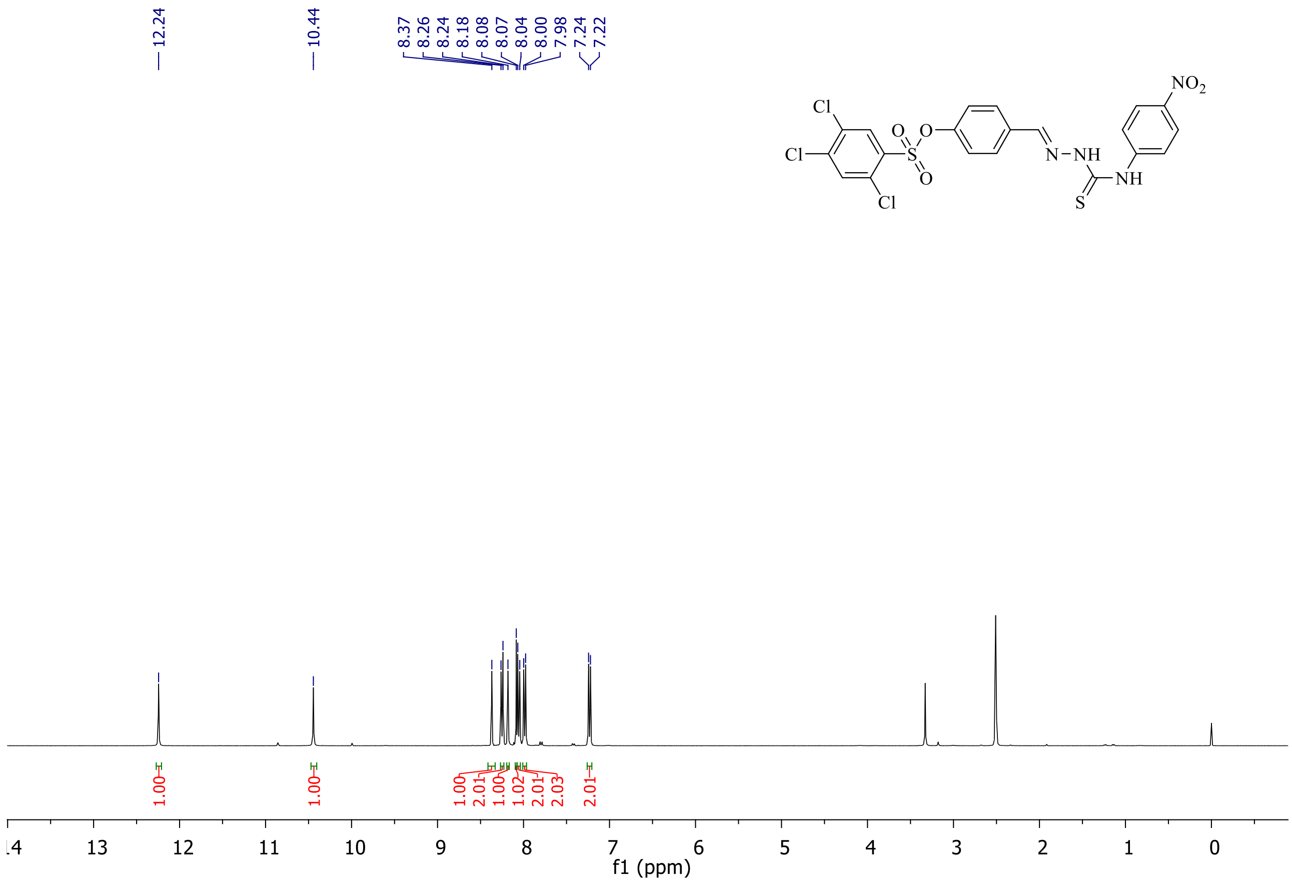

^{1}H NMR Spectrum of Compound 22

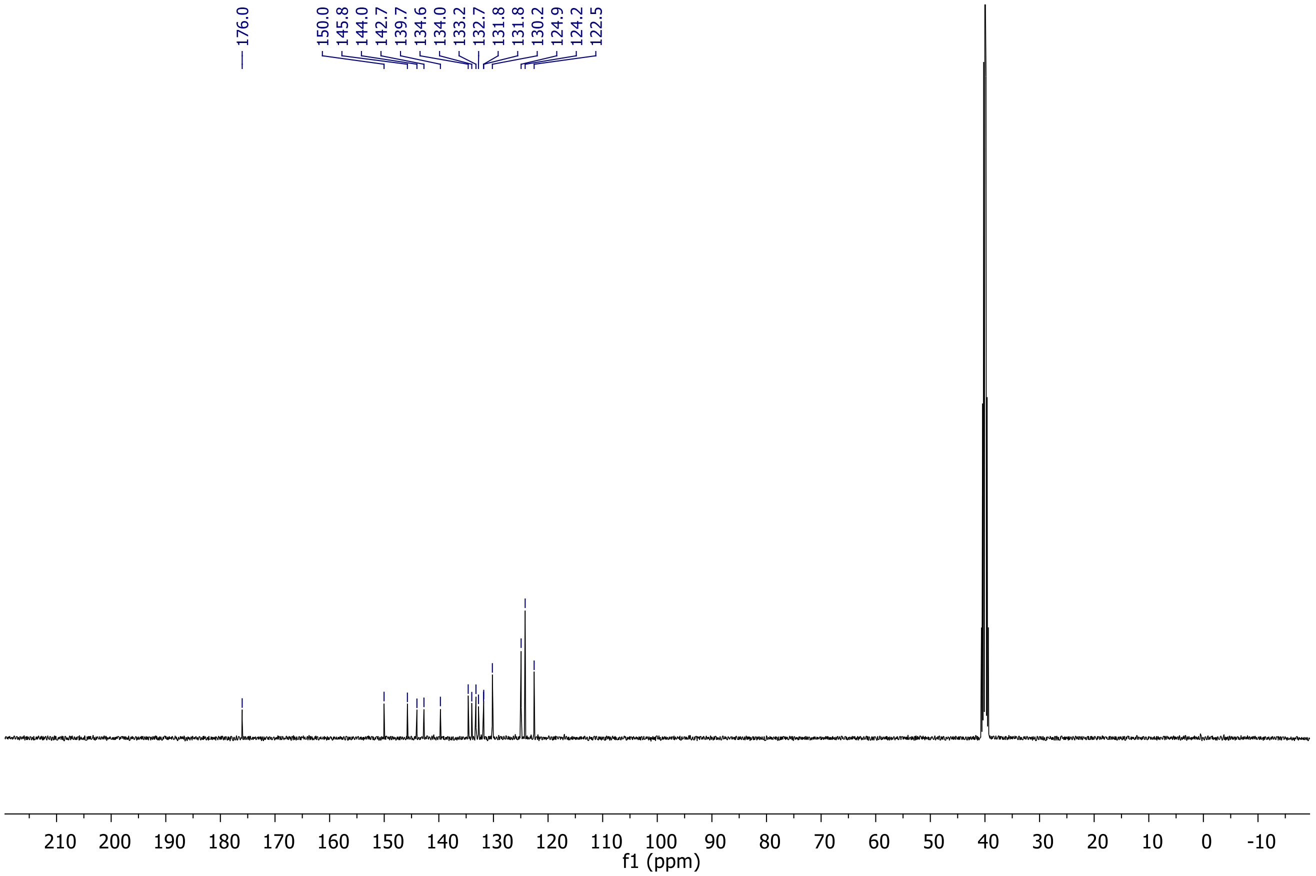

^{13}C NMR Spectrum of Compound 22

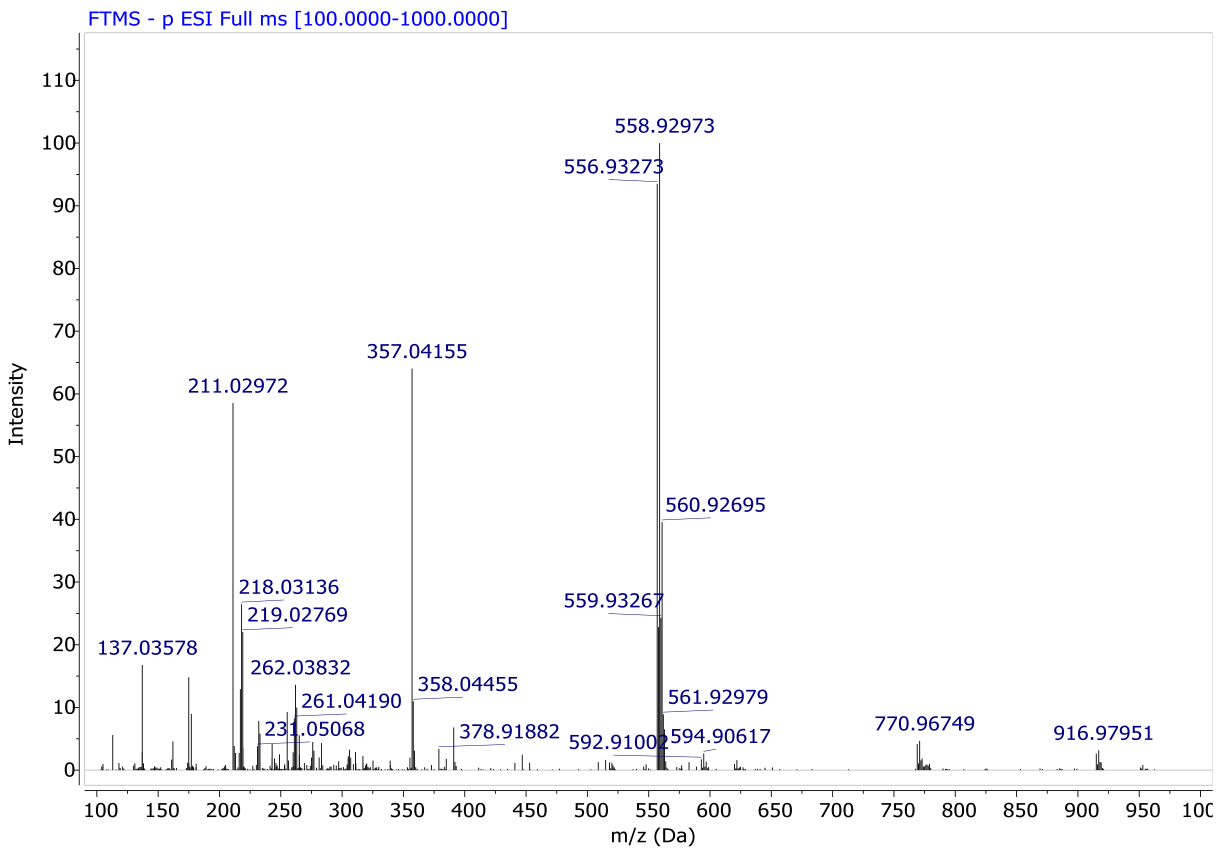

HRMS Spectrum of Compound 22

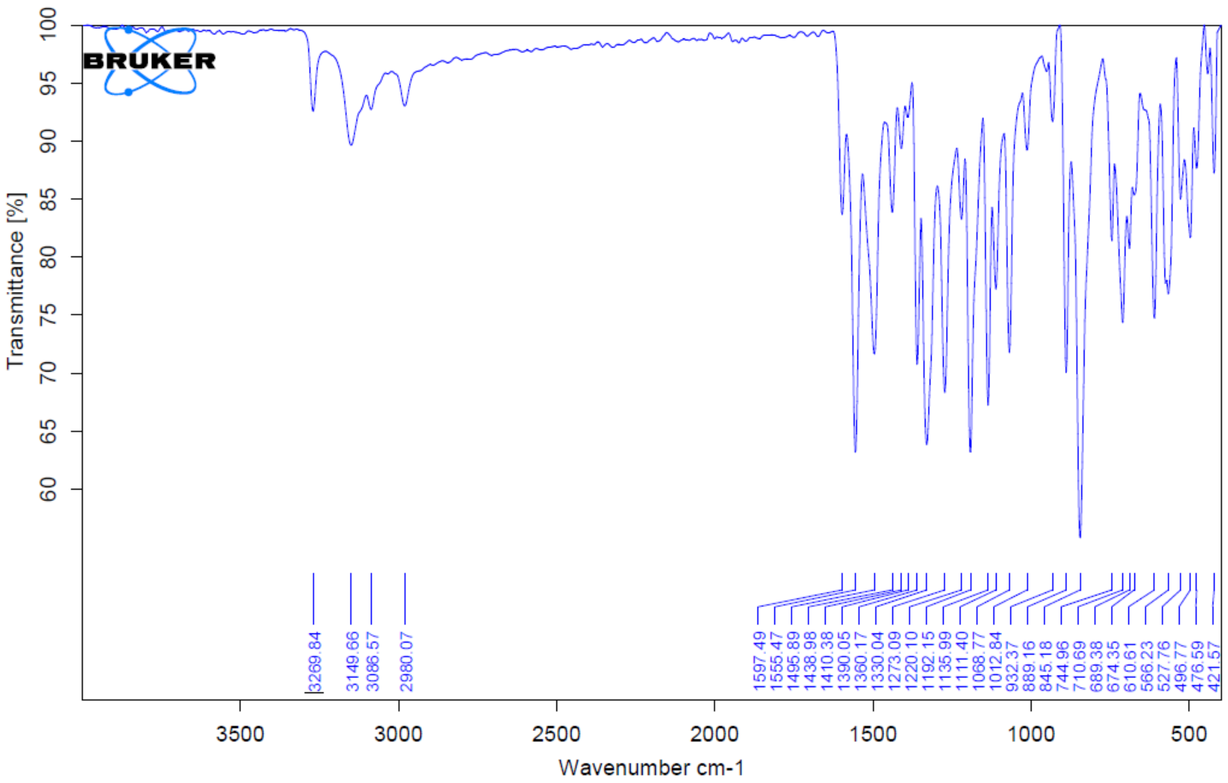

FTIR Spectrum of Compound 22

Lineweaver-Burk Graphs of Compounds 1-22 and Acarbose for α-Glu

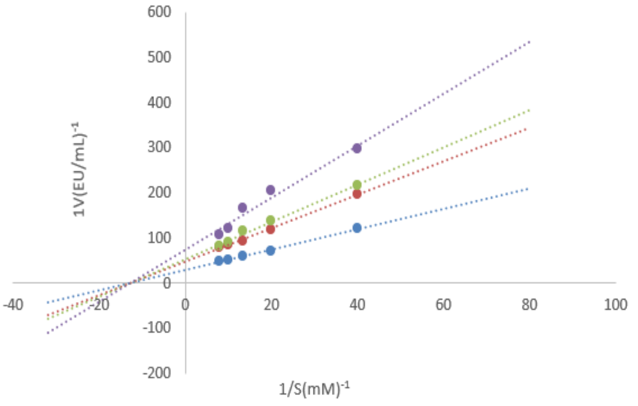

1

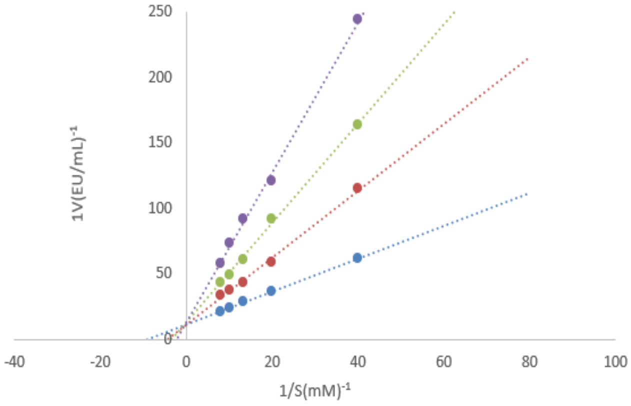

2

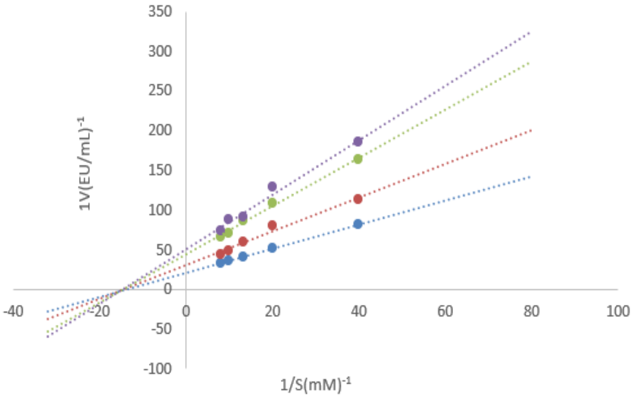

3

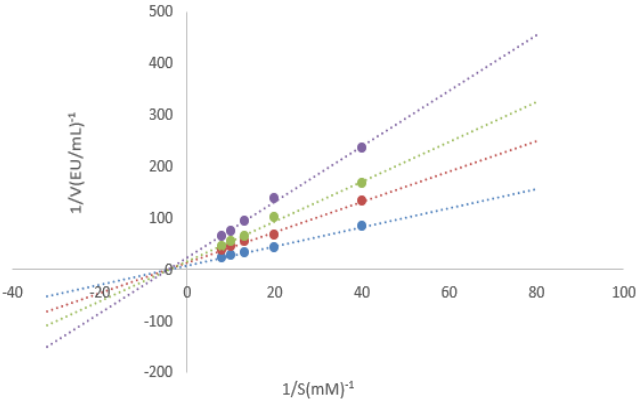

4

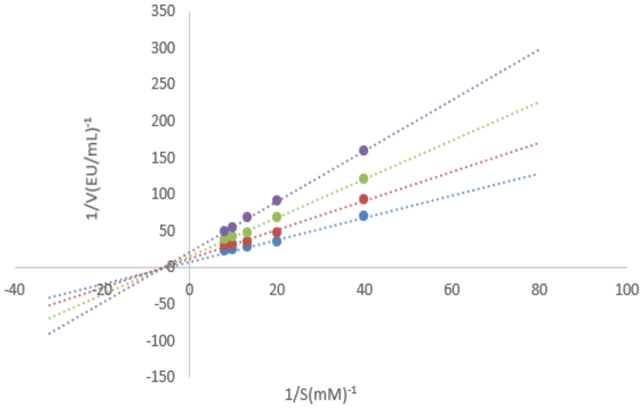

5

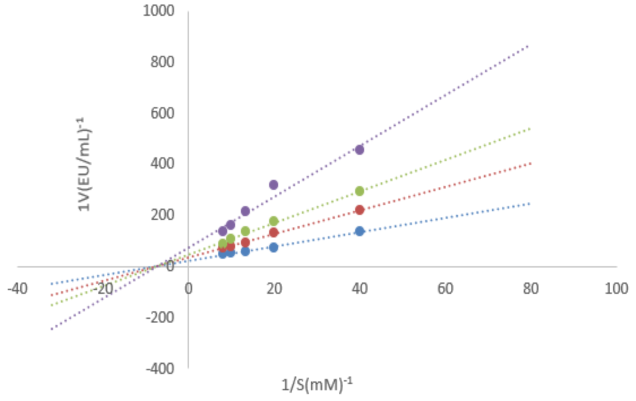

6

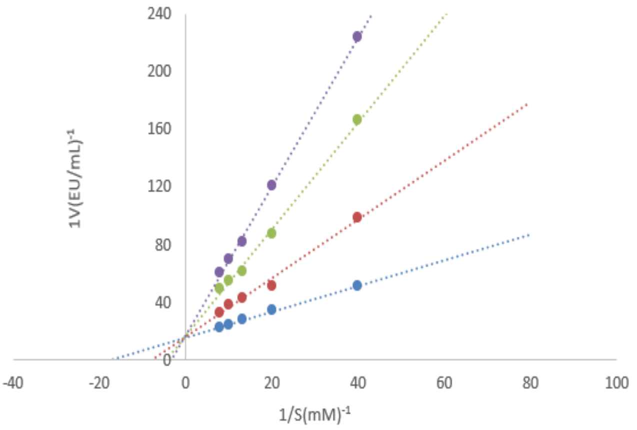

7

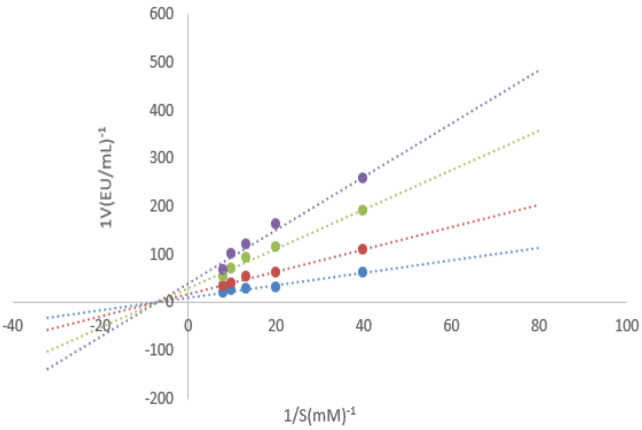

8

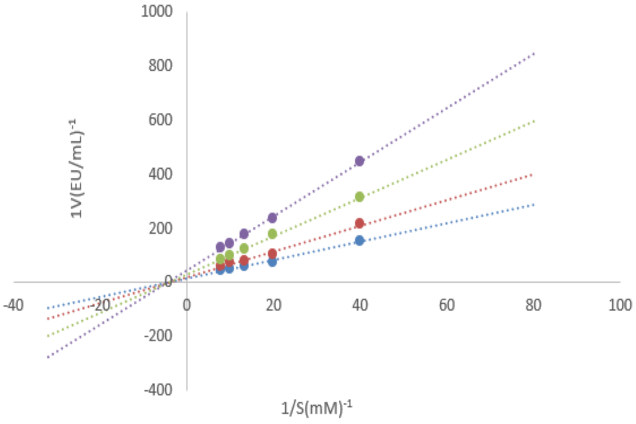

9

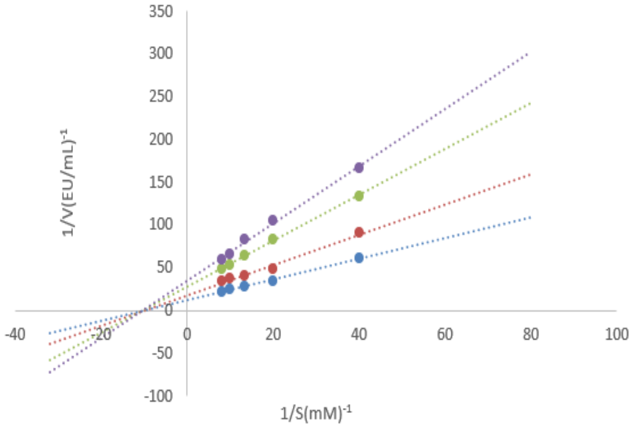

10

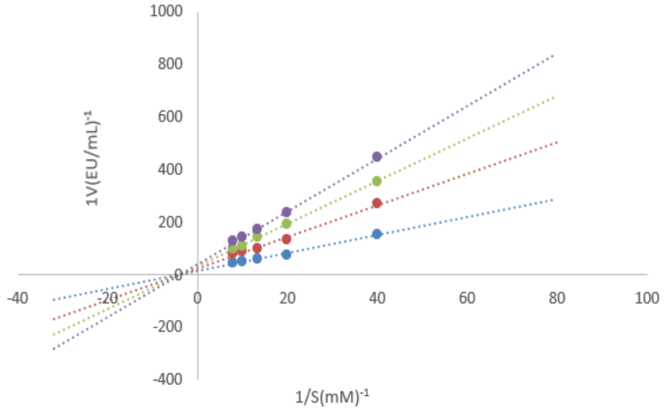

11

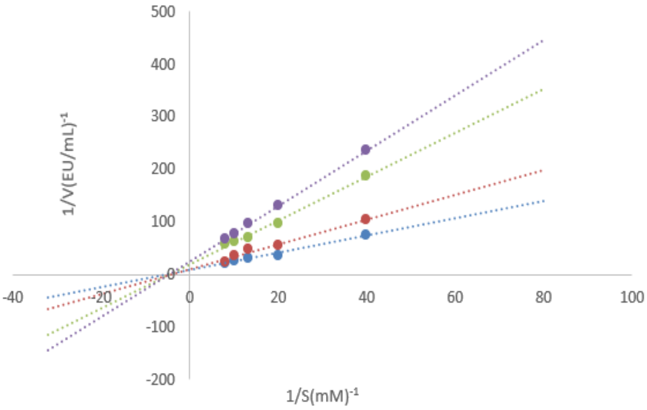

12

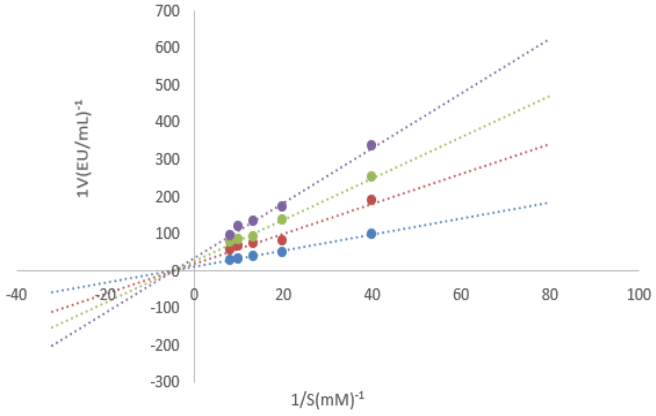

13

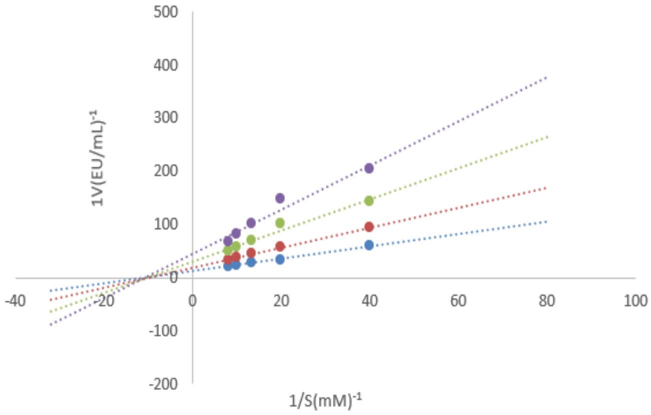

14

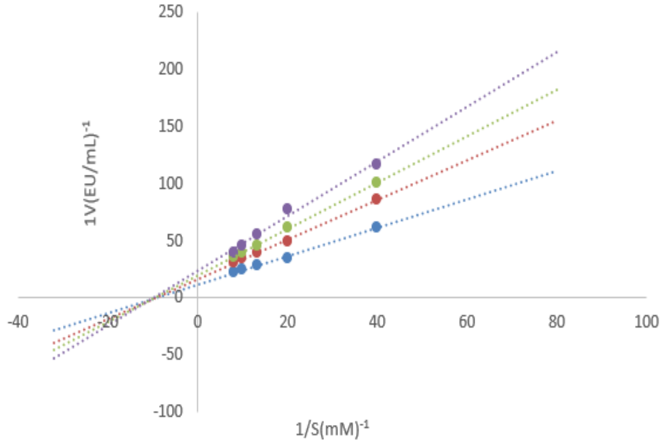

15

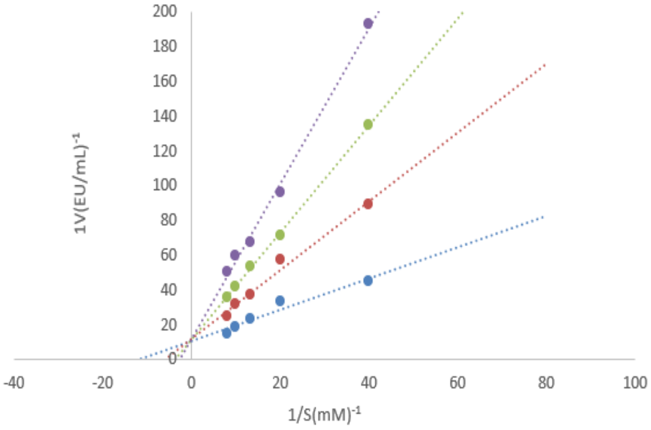

16

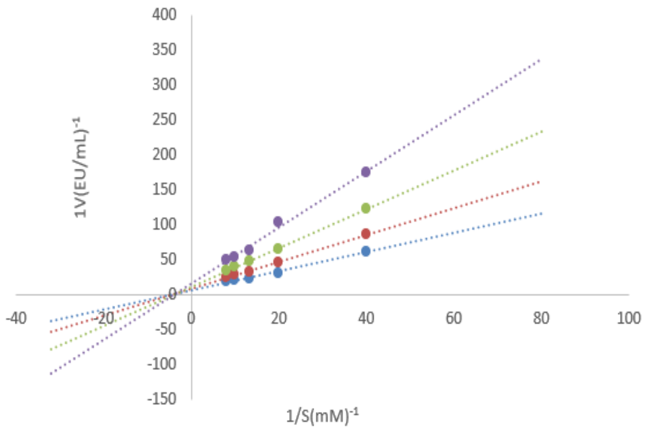

17

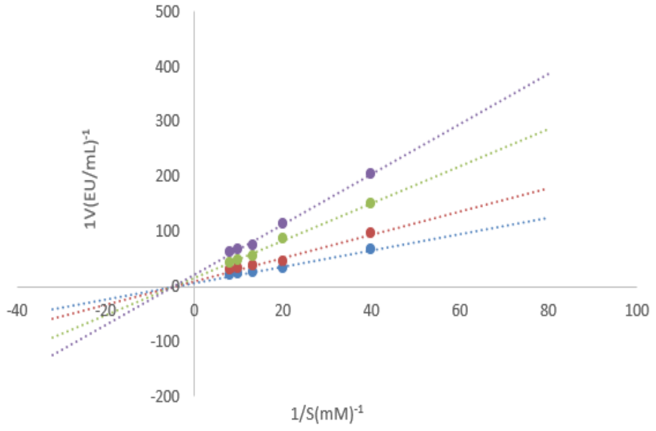

18

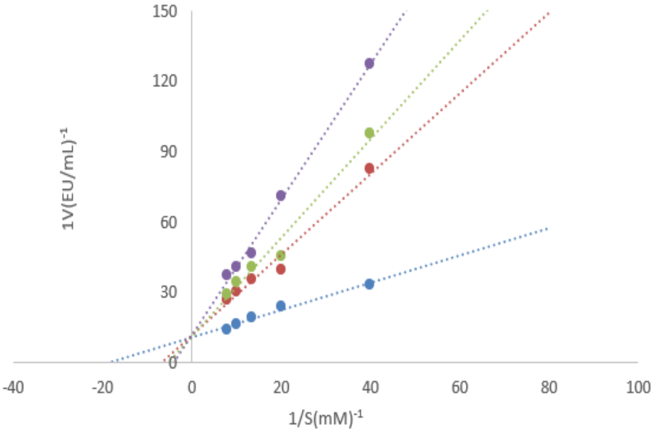

19

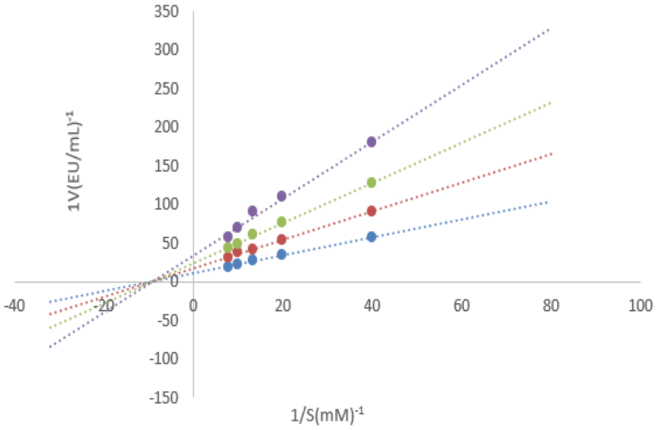

20

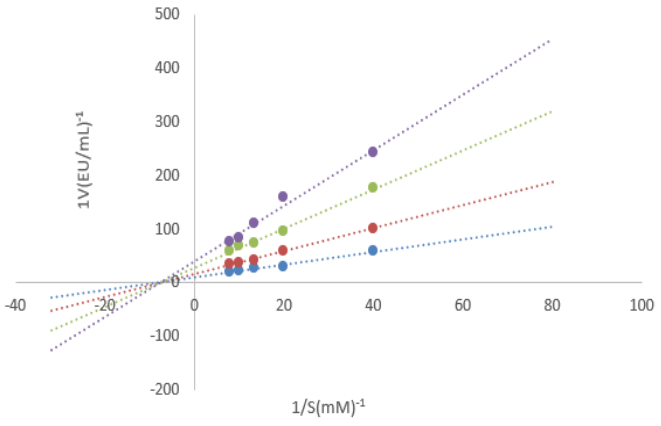

21

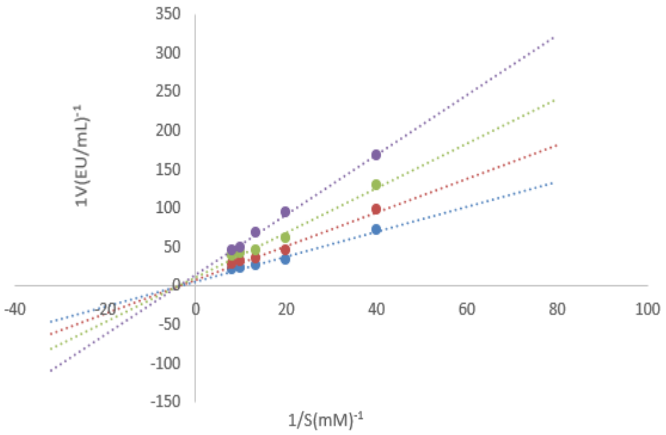

22

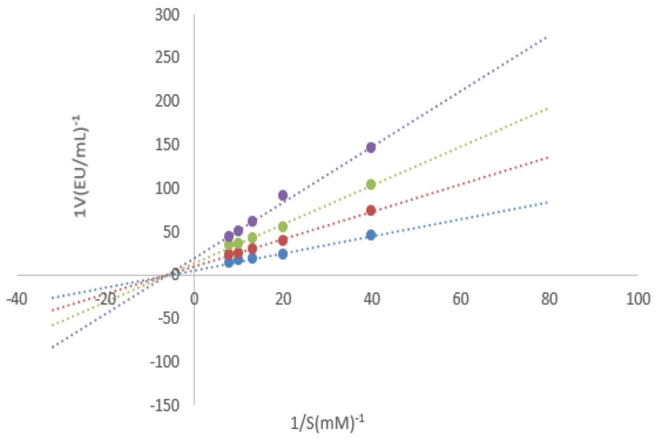

Acarbose

Table S1. The interactions of compound **16** with α-Glu and α-Amy

**Enzyme**	**Binding energy (kcal/mol)**	**Interacting residues**	**Type of interaction**	**Distance (Å)**
**α-Glu**	-69.67	Asn241	Hydrogen bond	1.90
		Glu276	Hydrogen bond	1.85 and 2.19
		Ash214	Hydrogen bond	2.26
		Phe157	π-π stacking	4.86
		Hie245	π-π stacking	5.00
		Tyr71	π-π stacking	3.76
**α-Amy**	-66.02	Glh233	Hydrogen bond	2.72
		Asp197	Hydrogen bond	2.08 and 2.28
		Lys200	Halogen bond	2.28 and 3.08
